# Mechanism-based heparanase inhibitors reduce cancer metastasis in vivo

**DOI:** 10.1073/pnas.2203167119

**Published:** 2022-07-26

**Authors:** Casper de Boer, Zachary Armstrong, Vincent A. J. Lit, Uri Barash, Gijs Ruijgrok, Ilanit Boyango, Merle M. Weitzenberg, Sybrin P. Schröder, Alexi J. C. Sarris, Nico J. Meeuwenoord, Pedro Bule, Yasmine Kayal, Neta Ilan, Jeroen D. C. Codée, Israel Vlodavsky, Herman S. Overkleeft, Gideon J. Davies, Liang Wu

**Affiliations:** ^a^Department of Bio-organic Synthesis, Leiden Institute of Chemistry, Leiden University, 2333 CC Leiden, The Netherlands;; ^b^Department of Chemistry, University of York, Heslington, York, YO10 5DD, UK;; ^c^Current address: Department of Bio-organic Synthesis, Leiden Institute of Chemistry, Leiden University, 2333 CC Leiden, The Netherlands;; ^d^Technion Integrated Cancer Center, The Bruce Rappaport Faculty of Medicine, Technion, Haifa 31096, Israel;; ^e^Current address: Centre for Interdisciplinary Research in Animal Health, Faculty of Veterinary Medicine, University of Lisbon, 1300-477 Lisbon, Portugal;; ^f^The Rosalind Franklin Institute, Harwell, OX11 0FA, UK

**Keywords:** heparanase, heparan sulfate, cancer, metastasis, covalent inhibition

## Abstract

Heparan sulfate proteoglycans (HSPGs) mediate essential interactions throughout the extracellular matrix (ECM), providing signals that regulate cellular growth and development. Altered HSPG composition during tumorigenesis strongly aids cancer progression. Heparanase (HPSE) is the principal enzyme responsible for extracellular heparan sulfate catabolism and is markedly up-regulated in aggressive cancers. HPSE overactivity degrades HSPGs within the ECM, facilitating metastatic dissemination and releasing mitogens that drive cellular proliferation. Reducing extracellular HPSE activity reduces cancer growth, but few effective inhibitors are known, and none are clinically approved. Inspired by the natural glycosidase inhibitor cyclophellitol, we developed nanomolar mechanism-based, irreversible HPSE inhibitors that are effective within physiological environments. Application of cyclophellitol-derived HPSE inhibitors reduces cancer aggression in cellulo and significantly ameliorates murine metastasis. Mechanism-based irreversible HPSE inhibition is an unexplored anticancer strategy. We demonstrate the feasibility of such compounds to control pathological HPSE-driven malignancies.

## Significance

Cancer growth is accompanied by changes to the extracellular environment of tumors, which aids the proliferation and spread of cancer cells. Cancer-associated extracellular matrix changes include excessive degradation of heparan sulfate carbohydrates, promoting metastatic spread by multiple mechanisms. Heparanase is the main human enzyme responsible for extracellular heparan sulfate breakdown and strongly drives metastasis when overexpressed. Few effective heparanase inhibitors are known, and the most effective molecules to date possess nondrug-like structures with multiple off-target effects. We have carried out structure-guided development of heparanase inhibitors, which covalently bind to the enzyme active site to cause irreversible inhibition. These inhibitors are heparanase specific and reduce metastasis in animal models with comparable efficacy to current “best-in-class” compounds.

Cancer progression is accompanied by extensive changes to the tumor microenvironment, which support the proliferation and dissemination of malignant cells. Extracellular matrix (ECM) remodeling by cancer and associated stromal cells utilizes a host of enzymes that act upon both proteins and carbohydrates within the ECM, causing profound compositional changes that can drive growth of the primary tumor, as well as prime distant sites for metastasis (1).

Heparan sulfate proteoglycans (HSPGs) are a fundamental class of ECM constituent, comprised of pericellular and extracellular core proteins conjugated to one or more chains of the glycosaminoglycan polysaccharide heparan sulfate (HS) (2). HSPGs mediate myriad biological processes, including signaling (3), developmental patterning (4), adhesion (5), barrier formation (6, 7), endocytosis (8), and viral entry (9–11). These processes largely depend upon the HS polysaccharides adorning the core protein (12), whose heterogeneous structures allow for interaction with multiple diverse partners.

**Fig. 1.**
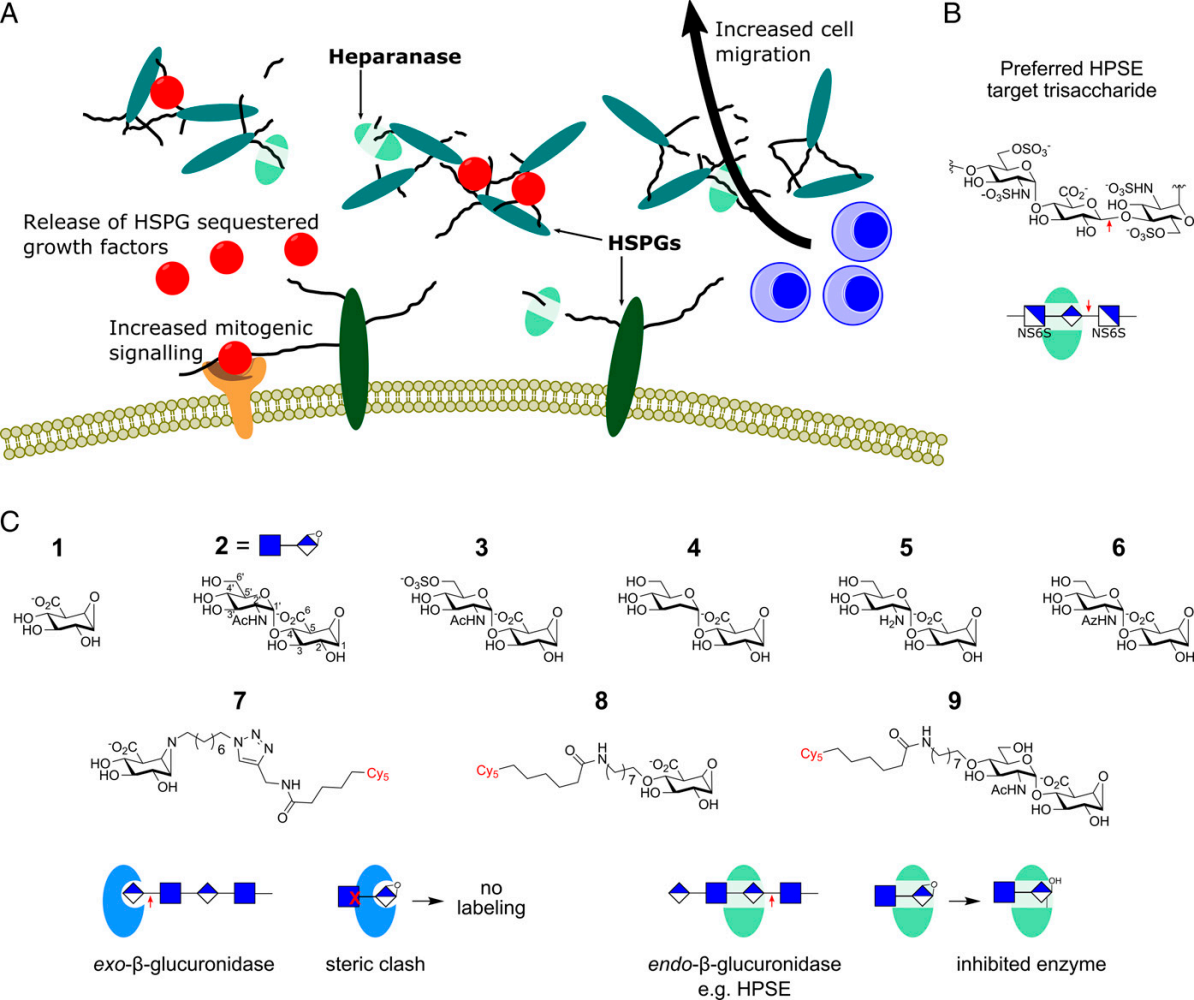
Design and development of HS-configured cyclophellitol pseudodisaccharides. (*A*) Biological effects of HPSE overexpression in the extracellular space. Excessive degradation of HSPG networks in basement membranes facilitates cell migration to and from the vasculature. Release of HSPG sequestered growth factors stimulates cell proliferation. (*B*) Preferred HPSE target sequence, comprising a GlcA residue flanked by two sulfated glucosamine residues. (*C*) Inhibitors and probes used in this study; atom reference positions are shown on inhibitor **2**. Full structures of **7**–**9**, including Cy5 linker, are shown in the *SI Appendix*. (*Bottom*) Principal of pseudodisaccharide HPSE selectivity via steric occlusion of exo-β-glucuronidase binding.

Given the importance of HSPGs within the ECM, tightly coordinated biosynthesis and breakdown mechanisms act to regulate their composition (13, 14). HSPG breakdown is mediated (in part) by endo- and exo-glycosidases, which hydrolytically cleave within or at the termini of HS polysaccharide chains, respectively (14). Heparanase (HPSE) is a mammalian endo-β-D-glucuronidase (hereafter, endo-β-glucuronidase) that plays a key role in HS polysaccharide degradation (15). Initially produced as a proenzyme (proHPSE), HPSE is matured within lysosomes by proteolysis of a linker peptide that obstructs its active site. Mature HPSE remains active within lysosomes, but can also be secreted into the extracellular space, where it aids ECM remodeling during tissue development and homeostasis. In contrast to its physiological role in ECM maintenance, pathological HPSE overexpression strongly drives the growth of aggressive metastatic cancers (16): excessive HSPG degradation within the ECM directly facilitates cancer cell migration to and from the vasculature (17, 18), while growth factors and cytokines liberated upon HSPG degradation stimulate proliferation and angiogenesis (19, 20) (Fig. 1*A*). HPSE overexpression can also increase the formation of tumor-derived exosomes, which circulate to distal tissues to establish premetastatic niche environments primed for colonization (21–23).

HPSE is the sole human enzyme responsible for extracellular HS polysaccharide degradation. Accordingly, there is intense interest in its inhibition as an anticancer strategy. A plethora of small-molecule (24–27), saccharide (28–31), neoproteoglycan (32, 33), and antibody (34) based HPSE inhibitors have been reported (35), although, to date, only four compounds have progressed to clinical trials (*SI Appendix*, Fig. S1) (28–31). Notably, these current “best-in-class” inhibitors are all polyanionic oligo-/polysaccharide derivatives that mimic the physicochemical properties of the natural HPSE inhibitor heparin, a glycosaminoglycan polysaccharide closely related to HS. The heparin-like properties of these best-in-class inhibitors, along with their structural heterogeneity, can produce unwanted pleiotropic effects, such as anticoagulation and growth factor binding, which complicate their clinical use (36).

Comparatively few small-molecule HPSE inhibitors with in vivo efficacy have been described (35). The active site of HPSE has proven challenging for small-molecule pharmacological intervention, given its extensive interaction surface evolved to bind large HS polysaccharides. Such challenging sites are often well targeted by mechanism-based covalent inhibitors, which may still react with an enzyme despite weak or transient initial binding. We previously reported that derivatives of the naturally occurring β-glucosidase inhibitor cyclophellitol yield highly active mechanism-based retaining glycosidase inhibitors and probes (37–40). Enzymatic attack by glycosidases upon the electrophilic epoxide “warhead” of cyclophellitol-derived molecules leads to alkylation of a catalytically essential nucleophile residue, resulting in covalent and irreversible inhibition (*SI Appendix*, Fig. S2). Tuning the functionality and stereochemistry of cyclophellitol-derived scaffolds alters target selectivity, in principle allowing inhibition of any retaining glycosidase enzyme (41).

Herein, we present HS-configured pseudodisaccharides, featuring a cyclitol epoxide designed to bind selectively to the HPSE active site and react with its essential catalytic nucleophile residue. We show that these HS-configured pseudodisaccharides are nanomolar, irreversible HPSE inhibitors in vitro and markedly reduce cancer aggression in in cellulo and in vivo cancer models. Covalent irreversible inhibition remains an underexploited avenue for therapeutic exploitation. Our results demonstrate that compounds acting by this mechanism can deliver effective treatments for addressing HPSE-driven cancer aggression.

## Results

### Design and construction of HPSE inhibitors.

Pharmacological HPSE inhibition effectively ameliorates aggression and metastasis across a wide range of cancer types (35). However, the most effective HPSE inhibitors to date are nondrug-like polyanionic heparin-mimetics, which are structurally complex and often heterogenous (*SI Appendix*, Fig. S1) (28–31). We sought to develop well-defined small molecules that selectively bind the HPSE active site and can covalently label the enzyme to produce potent and durable inhibition.

Structures of HPSE complexed with HS oligosaccharides show that the enzyme active site preferentially interacts with a sulfated disaccharide at its −1/−2 subsites (15) (Fig. 1*B*; see (42) for active site nomenclature). We hypothesized that elaboration of a previously reported glucuronyl-cyclophellitol **1** (38) with a nonreducing end (NRE) glucosamine sugar could enhance its interaction with the endo-acting binding cleft of HPSE, while simultaneously occluding interactions with the shallower binding pockets of exo-β-glucuronidases (Fig. 1*C*). Native HS-like α-1,4-GlcNAc and α-1,4-GlcNAc-6′-O-sulfate were appended to the NRE of **1** to create pseudodisaccharides **2** and **3,** respectively. We also examined the effect of 2′ modified deoxy-, amino- and GlcNAz- NRE sugar conjugates (43–). Finally, three Cy5 fluorescent probes were prepared: the previously reported glucuronyl-cyclophellitol aziridine **7** (38) and fluorescent pseudomonosaccharide **8** and pseudodisaccharide **9** (analogs of **1** and **2,** respectively).

### Mechanism-based HPSE inhibitors display nanomolar potency in vitro.

Initial tests of the inhibitory selectivity and potency of **1**–**6** were conducted against a panel of recombinant β-glucuronidase enzymes. Humans express two distinct and unrelated β-glucuronidases: endo-β-glucuronidase HPSE falls under the GH79 family of the sequence-based Carbohydrate Active enZymes (CAZy) classification (44), while exo-β-glucuronidase GUSB is a CAZy GH2 family enzyme involved in lysosomal mucopolysaccharide breakdown. We prepared recombinant human HPSE for inhibitor evaluation, along with its precursor proHPSE, whose active site is occluded by a linker peptide and should thus be unreactive to pseudodisaccharides. Due to the intractability of producing human GUSB, we turned to a GH2 ortholog, EcGUSB, from *Escherichia coli*, which shares 47.6% sequence identity with the human enzyme, including full conservation of active site residues (*SI Appendix*, Fig. S3). As further tests of inhibitor specificity, we also assessed GH79 orthologs BpHep from *Burkholderia pseudomallei* (45) and AcGH79 from *Acidobacterium capsulatum* (46), which share 20.1% and 20.8% sequence identity with HPSE, respectively (*SI Appendix*, Fig. S4).

**Fig. 2.**
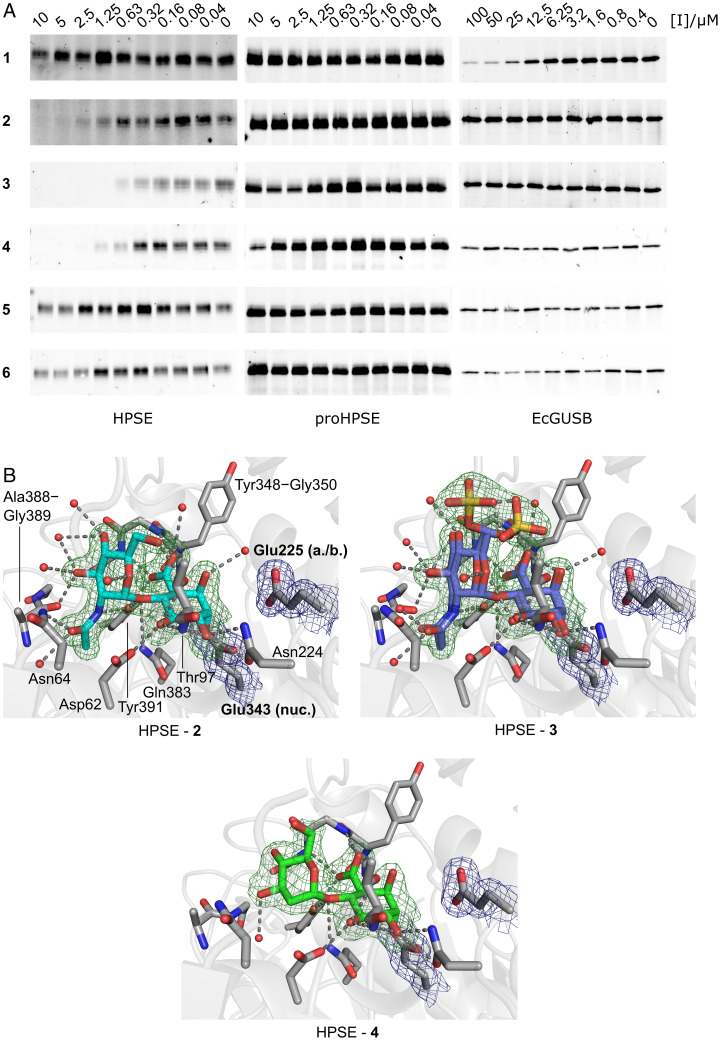
In vitro HPSE inhibition by HS cyclophellitol inhibitors. (*A*) cABPP gels for HPSE, proHPSE, and EcGUSB inhibition by **1–6**. Pseudodisaccharides **2–4** inhibit HPSE labeling with nanomolar potency. Only **1** inhibits EcGUSB. No inhibitors were effective against proHPSE. Full length gel images are available in Fig. S8. (*B*) Active site views from crystal structures of HPSE in complex with **2–4**, showing covalent labeling of the HPSE catalytic nucleophile. Electron density for sidechains is REFMAC σ_A_-weighted 2mFo-DFc, contoured to 1σ (0.23–0.26 e^−^.Å^−3^). Electron density for ligands is REFMAC σ_A_-weighted mFo-DFc, contoured to 3σ (0.25–0.27 e^−^.Å^−3^).

We first assessed enzyme inhibition using a competitive activity-based protein profiling (cABPP) assay, which measures the ability of inhibitors to abrogate enzyme labeling by the fluorescent pan-β-glucuronidase activity-based probe (ABP) **7** (38). Using cABPP, pseudomonosaccharide **1** proved to be an ineffective HPSE inhibitor up to 10 μM, while **2**, **3,** and **4** all displayed nanomolar HPSE inhibition potencies (Fig. 2*A*). Inhibitor **3** showed greater potency than **2** and **4**, likely due to electrostatic interactions from its 6′-O-sulfate. Effective HPSE inhibition by **4** clearly demonstrates some tolerance of nonnatural 2′ modifications in pseudodisaccharide inhibitors, although the weaker inhibition of **5** and **6** suggests that positive charge or steric bulk at 2′ are poorly tolerated. As expected, no compounds examined inhibited proHPSE, consistent with the steric requirement of an extended binding cleft for pseudodisaccharide binding. Similarly, **1**, but not **2**–**6,** inhibited the exo-β-glucuronidase EcGUSB. Inhibition of BpHep and AcGH79 followed similar patterns to those observed for HPSE, with all compounds apart from **5** showing activity against one or both enzymes (*SI Appendix*, Fig. S5).

We further analyzed the molecular basis of interaction between our inhibitors and their targets by X-ray crystallography, using ligand soaking to derivatize enzymes *in crystallo.* **2**, **3**, and **4** were all observed to specifically occupy the HPSE active site cleft, with covalent linkages between their pseudoanomeric (C1) carbons and the enzyme nucleophile Glu343. The 6′-O-sulfate of **3** adopted two equally populated configurations, both well placed to interact with basic residues around the HPSE substrate binding cleft (Lys158, Lys159, Lys161, Arg303). Noncovalent interactions for **2**–**4** within the HPSE cleft were generally similar to those previously characterized for HS substrates (15), albeit with a ∼17° rotation around C1′ for the NRE deoxy-sugar of **4** (compared to **2**), owing to its lack of 2′ substituent (Fig. 2*B*). Complexes of **2** and **3** with AcGH79 and BpHep showed similar interaction modes as found for HPSE (*SI Appendix*, Fig. S6), indicating a general GH79 reactivity mode for HS pseudodisaccharides. Of note, HPSE is the sole active GH79 enzyme present in humans. Consistent with its cABPP profile, we could only obtain an EcGUSB complex with **1**, which showed expected active site occupation and nucleophile labeling (*SI Appendix*, Fig. S6).

### Mechanism-based HPSE inhibitors are effective in physiological milieux.

Based on results from cABPP trials, we advanced a subset of inhibitors (47–) to evaluate HPSE inhibition in more biologically relevant milieux, including within complex cellular mixtures and against more physiologically relevant substrates.

**Fig. 3.**
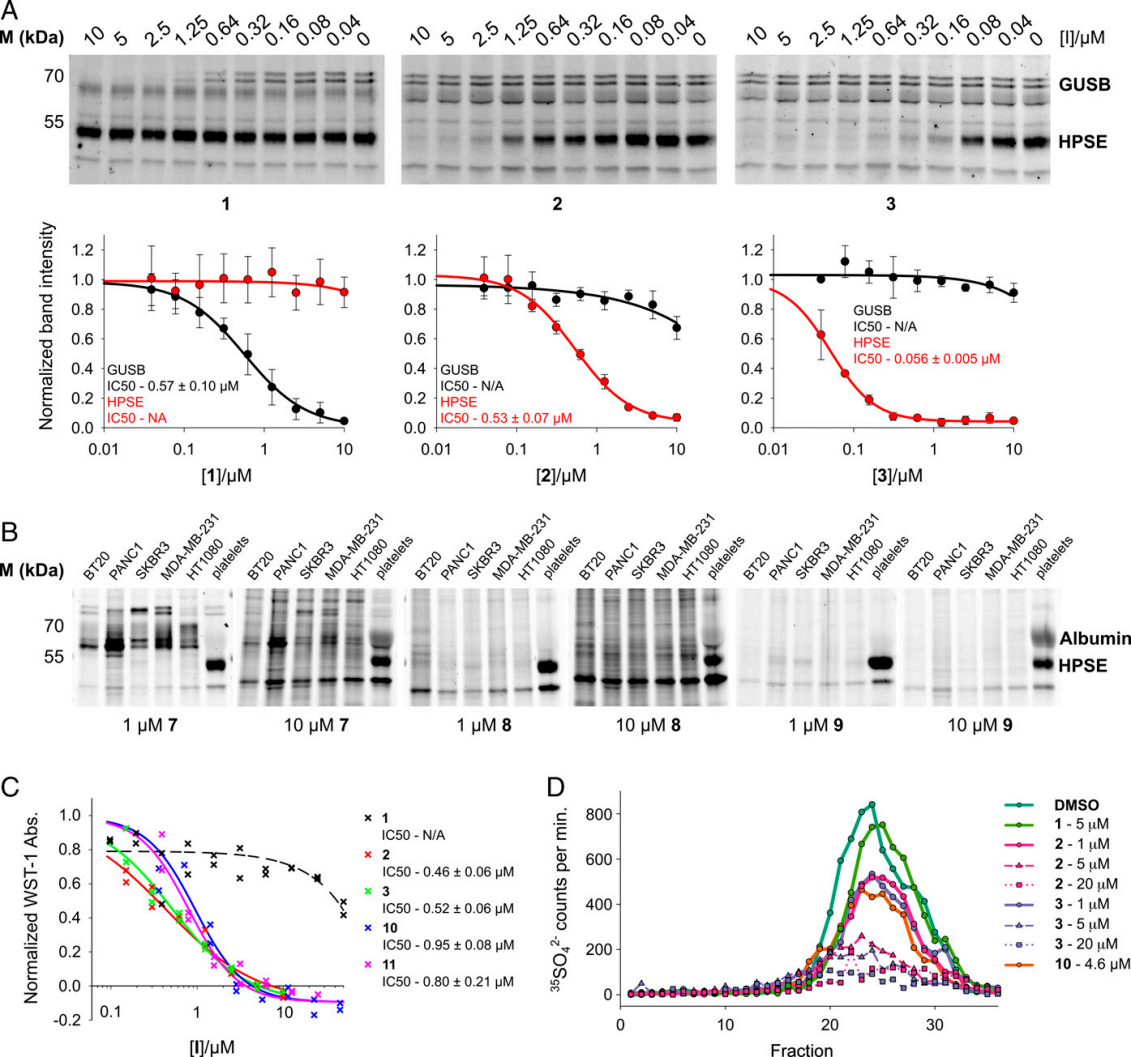
HPSE inhibition in physiologically representative scenarios. (*A*) Cyclophellitol pseudodisaccharides **2** and **3** selectively inhibit endogenous HPSE in human platelet lysates, which contain both GUSB and HPSE β-glucuronidase activities. Representative gels are shown, alongside graphs showing normalized quantitated band intensities vs. inhibitor concentration. Datapoints are mean ± SD (*n* = 3). (*B*) Cy5 fluorescent ABP **7**, **8** and **9** labeled cell lysates. Pseudodisaccharide **9** displays superior HPSE specificity compared to **7** and **8**. (*C*) **2** and **3** inhibit HPSE-mediated cleavage of the synthetic HS pentasaccharide fondaparinux, with greater potency than reference inhibitors **10** and **11**. Individual assay datapoints (*n* = 2) plotted. (*D*) **2** and **3** effectively inhibit HPSE-mediated liberation of H^35^S fragments from basement membrane H^35^SPGs.

Platelet lysates contain complex mixtures of overlapping enzyme activities, including both the endo- and exo-β-glucuronidases HPSE and GUSB, respectively; thus, they provide a challenging environment in which to examine specificity (48). cABPP of platelet lysates showed highly selective inhibition of human platelet HPSE by both **2** and **3** (IC50s = ∼0.53 μM and ∼0.06 μM, respectively), while **1** exclusively inhibited GUSB (IC50 = ∼0.75 μM) (Fig. 3*A*). To estimate the broader proteome-wide specificity of our inhibitors across concentrations, we applied fluorescent ABPs **7**, **8**, and **9** at 1 μM and 10 μM to lysates from a panel of human cancer cell lines: U87, PANC1, SKBR3, MDA-MB-231, and HT1080, as well as platelet lysates. Pseudodisaccharide **9** demonstrated markedly improved HPSE specificity over **7** and **8** in all lysates tested, with the most substantial off-target of **9** being serum albumin at 10 μM concentration (Fig. 3*B*).

We next investigated the ability of our inhibitors to abrogate HPSE processing of its native oligosaccharide substrates, using a colorimetric assay that detects cleavage of the synthetic HS pentasaccharide fondaparinux (49). Clear inhibition of HPSE-mediated fondaparinux cleavage was effected by both **2** (IC50 = ∼0.46 μM) and **3** (IC50 = ∼0.51 μM), which were both approximately twofold more potent than reference small-molecule HPSE inhibitors #98 (**10**; IC50 = ∼0.80 μM) and OGT2115 (24) (**11**; IC50 = ∼0.95 μM; *SI Appendix*, Fig. S1). In contrast to cABPP results, we observed no improved potency for **3** compared to **2** against HPSE-mediated fondaparinux cleavage. Little HPSE inhibition was produced by **1**, even at 50 μM (Fig. 3*C*).

Finally, we assessed our inhibitors against HPSE-mediated degradation of bona fide HSPGs in an ECM environment. Cultured bovine corneal endothelial cells secrete a subendothelial basement membrane material rich in proteoglycans, which can be radiolabeled by growth in the presence of ^35^SO_4_^2−^ (50). Solubilized H^35^S chains released from H^35^SPGs by HPSE digestion can be resolved chromatographically, with HPSE inhibition leading to a corresponding loss of signal. Both **2** and **3** produced robust, dose-dependent inhibition of recombinant HPSE-mediated H^35^SPG cleavage, with comparable levels of inhibition at 1 μM **2** or **3** compared to 4.6 μM **10**. As with the fondaparinux assay, no HPSE inhibition was observed for **1**, and we found no difference in potency between **2** and **3** (Fig. 3*D*). We also tested inhibition of endogenous cellular HPSE using this assay format. H^35^SPG digests by U87 glioma cell lysates produced similar H^35^S solubilization profiles whether inhibitor **2** was applied before or after lysis, indicating that this molecule can also penetrate cells to inhibit the intracellular HPSE pool (*SI Appendix*, Fig. S7).

### Mechanism-based HPSE inhibitors reduce cancer metastasis in vivo.

HPSE overexpression drives aggressive cancer growth and metastasis, leading to correspondingly worsened clinical outcomes (3). Given the in vitro efficacy of our pseudodisaccharide inhibitors, we sought to evaluate their efficacy against HPSE-driven malignancy in cellulo and in vivo.

**Fig. 4.**
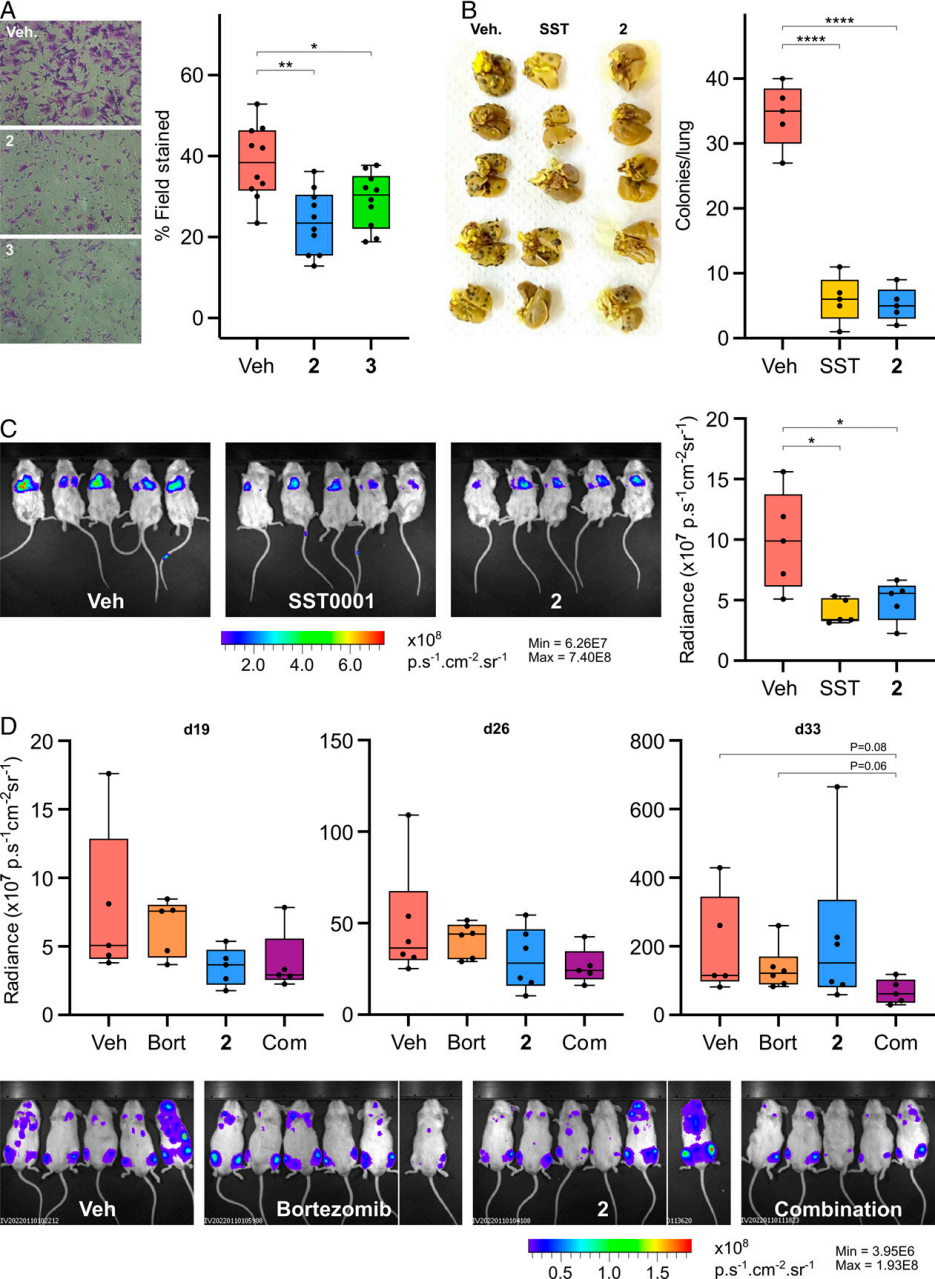
Cyclophellitol pseudodisaccharides reduce metastasis in cellulo and in vivo. (*A*) Inhibitors **2** and **3** reduce U87 cell invasion through a Matrigel-coated transwell. Representative fields of view of migrated cells (*Left*), alongside quantitation of invasion (% field stained) from 10 randomly selected fields of view (*Right*). Veh, Vehicle. (*B*) Inhibitor **2** reduces the formation of lung metastases by B16 melanoma cells, on par with HPSE inhibitor SST0001. Images of lungs showing metastatic B16 foci (*Left*), alongside quantitation of foci on lungs from five replicates (*Right*). (*C*) Inhibitor **2** reduces the formation of lung metastases formed by 4T1 breast cancer cells, with efficacy on par with SST0001. IVIS images of luciferase expressing 4T1 cell bioluminescence in lungs at the 14 d timepoint (*Left*) alongside quantitation of bioluminescent flux from five replicates (*Right*). (*D*) Inhibitor **2** reduces the formation of bone metastases formed by CAG myeloma cancer cells, with efficacy on par with bortezomib (Bort), and synergistic efficacy. IVIS images of luciferase expressing CAG cell bioluminescence in bones at the 33 d timepoint (*Bottom*) alongside quantitation of bioluminescent flux from five or six replicates (*Top*). All box and whisker plots show the mean, interquartile range, maxima and minima. Com, combination. Statistical comparisons are two-tailed Student’s *t* tests: *P < 0.05; **P < 0.01; ***P < 0.001; ****P < 0.0001.

Preliminary in cellulo evaluations of our inhibitors’ effects on cancer aggression were conducted using a Matrigel invasion assay, which measures the ability of cells to migrate through transwell inserts coated with the ECM material Matrigel. Matrigel invasion by cells requires a broad complement of proteases and glycosidases, thereby mimicking some aspects of metastasis in vivo (51). U87 glioblastoma cells, which show a strong HPSE-dependent metastatic phenotype (52), displayed significantly reduced Matrigel invasion upon application of **2** or **3**, indicating potential efficacy as antimetastatic agents (Fig. 4*A*).

Encouraged by the efficacy of our inhibitors against Matrigel invasion, we proceeded to murine experimental metastasis models to evaluate the efficacy of our compounds in vivo. Given the amounts of compound required, the similar efficacy of **2** and **3** against Matrigel invasion, and the more scalable synthesis of **2**, only this inhibitor was progressed toward in vivo experiments.

We initially tested a B16 murine melanoma metastasis model, measuring the formation of metastatic lung tumors by B16 melanoma cells after intravenous (i.v.) injection. The efficacy of covalent HPSE inhibition by **2** was compared to SST0001 (Roneparstat), a current best-in-class HPSE inhibitor and clinical trial candidate (*SI Appendix*, Fig. S1). Mice were inoculated intraperitoneally (i.p.) with 300 nmol of **2** (∼15 μmol/kg for a 20 g mouse), or 150 μg SST0001, followed by an i.v. injection of B16 cells alongside 2 nmol **2** or 10 μg SST0001. After 14 d, a marked reduction in lung growths was observed for mice treated with **2** compared to a phosphate buffered saline (PBS)-only control group (Fig. 4*B*). Remarkably, this reduction was also similar in magnitude to that elicited by SST0001, demonstrating potential in vivo efficacy for cyclophellitol-derived HPSE inhibitors.

Since HPSE drives metastasis in multiple cancer types, we tested **2** against another metastasis model—4T1 murine mammary carcinoma, which also migrates to the lungs. Here, we were aided by a constitutive luciferase expressing 4T1 line, enabling real-time tracking of cancer growth by chemiluminescent imaging. Similar to the B16 model, mice were dosed i.p. with 300 nmol of **2** or 150 μg SST0001, followed by i.v. injection of 4T1-Luc cells alongside 2 nmol **2** or 10 μg SST0001. Again, we observed a marked reduction in lung metastases following treatment, with comparable antimetastatic efficacy to that achieved by SST0001 (Fig. 4*C*).

Finally, we examined a CAG myeloma xenograft model, which faithfully recapitulates the invasion of multiple myeloma to the bone marrow. We compared the efficacy of **2** to bortezomib, a proteasome inhibitor and standard treatment for multiple myeloma and lymphomas, and also explored the possibility of synergistic proteasomal and HPSE inhibitor treatment. In contrast to the single initial dose regimens used for B16 and 4T1, a continuous dosing regimen was used in this assay to gauge the efficacy of **2** over longer treatment periods. Following injection of luciferase expressing CAG cells, mice were treated with a course of **2** (300 nmol/day i.p.), bortezomib (0.5 mg/kg twice weekly i.p.), or a combination of both compounds. Chemiluminescent imaging over time showed a moderate reduction in metastatic burden for mice treated with **2**, similar in magnitude to that achieved by bortezomib, albeit with somewhat reduced efficacy following prolonged treatment (33 d timepoint). This reduction in antimetastatic efficacy at the longer timepoint may reflect some metabolic instability in the structure of **2** (vide infra) or possible compensatory resistance. In contrast, the combined treatment regimen of **2**+bortezomib showed improved efficacy after 33 d compared to both monotherapies, highlighting the potential for combined proteasomal inhibition and covalent HPSE inhibition as an antimetastatic strategy (Fig. 4*D*).

## Discussion

Despite intense interest in the development of therapeutic HPSE inhibitors over several decades, no compounds have yet been approved for clinical use. This unmet need likely relates to the complex active site cleft of the HPSE enzyme, which makes multiple interactions with structurally diverse HS polysaccharide substrates. Such a binding surface presents a challenging target for pharmacological intervention; thus, the only HPSE inhibitors effective enough to have undergone clinical trials to date are molecules possessing a polyanionic saccharide structure similar to that of HS itself. These highly charged and often heterogeneous compounds interact with multiple proteins in vivo, producing pleiotropic effects that may complicate their clinical use.

Mechanism-based covalent inhibition is an unexplored paradigm for targeting HPSE. We have demonstrated here that pseudodisaccharides derived from the naturally occurring molecule cyclophellitol can irreversibly inhibit HPSE with nanomolar potency and high selectivity. By elaboration of a cyclophellitol-derived warhead with a α-1,4 glucosaminyl moiety, we effectively blocked inhibitor reactivity with exo-acting β-glucuronidases such as GUSB, while simultaneously enhancing potency for HPSE itself. Cyclophellitol-derived pseudodisaccharides are effective within complex physiological milieux and reduce cancer aggression in both in vitro and in vivo metastasis models. Further dose-ranging studies will be required to establish the minimal amounts of inhibitor required for therapeutic efficacy.

Covalency has historically been avoided during pharmacological development for fears of off-target toxicity. However, this inhibition modality offers several advantages compared to traditional competitive inhibition (53). Covalent inhibitors can effectively target challenging protein sites by virtue of their ability to label despite poor or transient initial enzyme binding. Improved potency is also readily achieved by time-dependent accumulation of inhibition, an effect that likely contributes to the nanomolar HPSE inhibition potencies observed for **2**–**4**. Noteworthy, HPSE has a long cellular lifetime of >72 h (t_1/2_ ∼30 h)[56]; thus, it may be especially well suited for targeting by irreversible inhibitors, whose effect will persist until the protein molecule is degraded. Potential off-target effects of our inhibitors are minimized by their mechanism-based nature of activation: efficient inhibition requires binding to a protein site that also contains the biochemical machinery necessary to process the electrophilic cyclophellitol warhead. These criteria are likely only well satisfied by HPSE in vivo.

Another major advantage of cyclophellitol-derived inhibitors is that rational improvements are readily designed and synthetically accessible. We have demonstrated effects on in vitro HPSE inhibition arising from modifications to the 6′ position (**3** vs. **2**) as well as the 2′ position (**4**–**6** vs. **2**) of our molecules, indicating that alterations to the NRE sugar of pseudodisaccharides can be well tolerated. Exploring the effect of increasingly nonnatural NRE substituents on inhibitor efficacy is warranted, especially with a view toward developing molecules with increased metabolic stability. HPSE-specific ABPs such as **9**, which incorporate a NRE fluorophore, will also enable facile detection of HPSE activity in cell and tissue samples. The development of matched ABPs and inhibitors, both derived from a common cyclophellitol scaffold, enables a pathway toward HPSE-directed theranostics for coupled diagnosis and treatment of HPSE-driven pathologies.

Metabolic stability is an important consideration for in vivo compounds. Two structural motifs on our inhibitors warrant comment in this regard: the cyclophellitol-derived epoxide warhead and the α-1,4 glycosidic bond. Concerning the former, proteome-wide profiling suggests that relatively few targets are labeled by simple epoxide electrophiles within biological milieux, with labeling driven primarily by enzyme recognition. Indeed, several pharmaceutical and natural product inhibitors (both reversible and irreversible) contain epoxide motifs, e.g., fosfomycin, scopolamine, and cyclophellitol itself, indicating this functionality can be stable and well tolerated in vivo. In contrast, the α-1,4 glycosidic bond of our inhibitors presents a potential metabolic vulnerability that may be targeted by α-glucosaminidases, such as the lysosomal enzyme NAGLU, effectively converting HS pseudodisaccharides to **1**. The design of next-generation inhibitors incorporating nonnatural NRE substituents, which inhibit HPSE but are not recognized by unrelated enzymes, should provide a solution to metabolic processing of the α-1,4 glycosidic bond. Incorporation of nonnatural NRE substituents also opens routes to alter hydrophobicity and other physicochemical properties, enabling renal excretion, albumin binding, and other pharmacokinetic parameters to be tuned.

In conclusion, we have presented a set of HPSE-specific mechanism-based inhibitors for biochemical studies and potential therapeutic applications. These compounds were rationally developed based on structural consideration of HPSE–HS interactions and further optimized using similar structure-based design principles. Ultimately, we envision that the development of improved inhibitors containing a highly specific warhead, coupled to metabolically stable glycoside-mimicking linkages, may enable the production of designer inhibitors for HPSE and other therapeutically relevant glycosidases.

## Methods

Detailed methods for gene expression, protein purification, biochemical assays, structural biology, cell invasion, animal metastasis experiments, and complete synthetic protocols can be found in the *SI Appendix*.

## Supplementary Material

Supplementary File

## Data Availability

Crystal structures have been deposited in the Protein Data Bank under the accession codes: 7PR7 (54) (HPSE–**2**), 7PR8 (55) (HPSE–**3**), 7PRT (56) (HPSE–**4**), 7PR9 (57) (BpHep–**2**), 7PRB (58) (BpHep–**3**), 7PSH (59) (*Apo* AcGH79), 7PSI (60) (AcGH79–**1**), 7PSJ (61) (AcGH79–**2**), 7PSK (62) (AcGH79–**3**), and 7PR6 (63) (EcGUSB–**1**).
