# Supplementary material for: Mechanism-based heparanase inhibitors reduce cancer metastasis in vivo
Source: Proc Natl Acad Sci U S A. 2022 Jul 26;119(31):e2203167119. doi: 10.1073/pnas.2203167119 (PMC9351465; doi:10.1073/pnas.2203167119)
Supplement: Supplementary File [file pnas.2203167119.sapp.pdf]

Supporting Information for

# Mechanism based heparanase inhibitors reduce cancer metastasis in vivo

Casper de Boer*[1], Zachary Armstrong*^[2], Vincent A. J. Lit*[1], Uri Barash[3], Gijs Ruijgrok[1], Ilanit Boyango[3], Merle M. Weitzenberg[1], Sybrin P. Schröder,[1] Alexi J. C. Sarris[1], Nico J. Meeuwenoord[1], Pedro Bule[&2], Yasmine Kayal[3], Neta Ilan[3], Jeroen D. C. Codée[1], Israel Vlodavsky[3], Herman S. Overkleeft[#1], Gideon J. Davies[#2], Liang Wu[#2,4]

1 - Department of Bio-organic Synthesis, Leiden Institute of Chemistry, Leiden University, Einsteinweg 55, 2333 CC Leiden, The Netherlands.

2 - Department of Chemistry, University of York, Heslington, York, YO10 5DD, U.K.

3 - Technion Integrated Cancer Center (TICC), The Bruce Rappaport Faculty of Medicine, Technion, Haifa 31096, Israel.

4 - The Rosalind Franklin Institute, Harwell, OX11 0FA, U.K.

*Contributed equally

# Correspondence to h.s.overkleeft@lic.leidenuniv.nl, gideon.davies@york.ac.uk, liang.wu@rfi.ac.uk

& Current address - Centre for Interdisciplinary Research in Animal Health, Faculty of Veterinary Medicine, University of Lisbon, Avenida da Universidade Técnica, 1300-477 Lisbon, Portugal

^ Current address - Department of Bio-organic Synthesis, Leiden Institute of Chemistry, Leiden University, Einsteinweg 55, 2333 CC Leiden, The Netherlands.

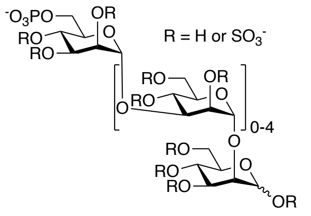


PI-88 (Phase III - terminated)

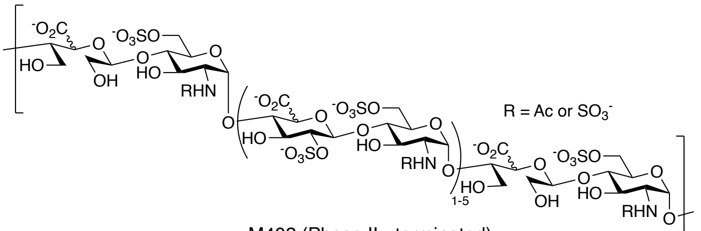


M402 (Phase II - terminated)

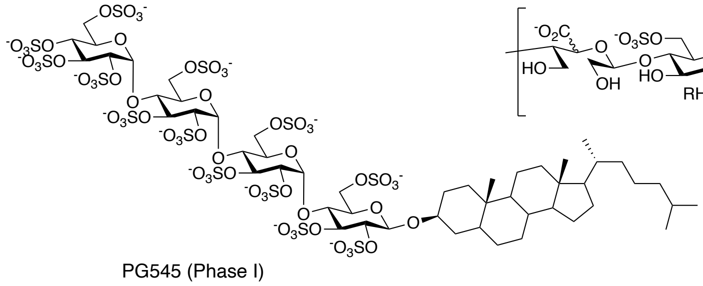

PG545 (Phase I)

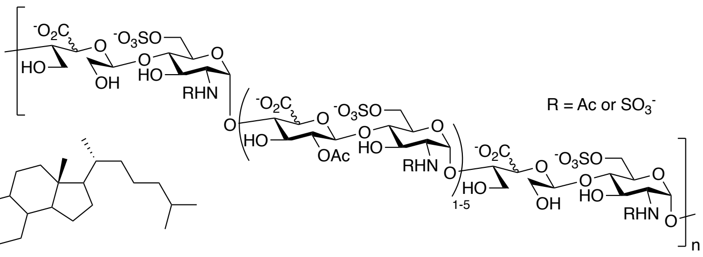


SST0001 (Phase I)

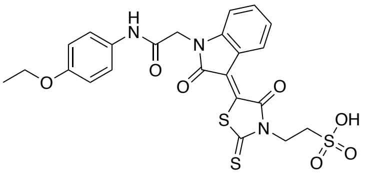

**10**
HPSE inhibitor #98

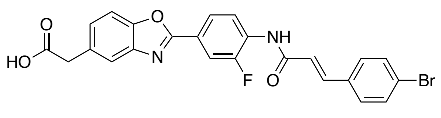

**11**
HPSE inhibitor OGT2115

**Figure S1** Top – HPSE inhibitors that have been tested in clinical trials to date. Bottom – additional HPSE inhibitors used in this study.

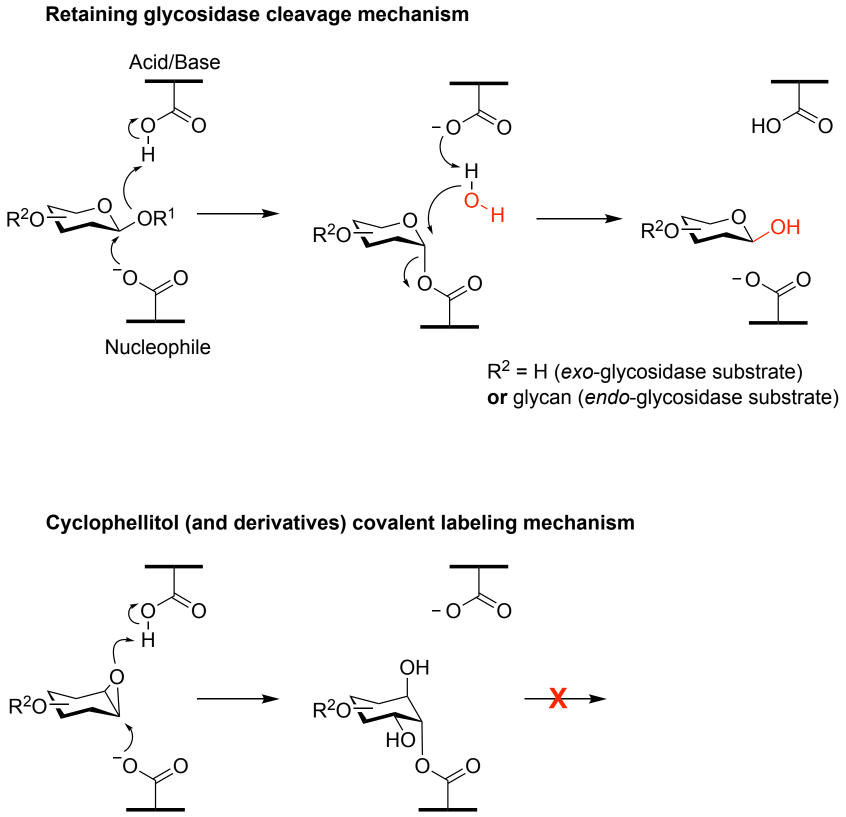


**Figure S2** Top: Double displacement mechanism used by typical retaining glycoside hydrolases during substrate cleavage. Bottom: Mechanism-based covalent labeling of retaining glycoside hydrolases by cyclophellitol and derivatives.

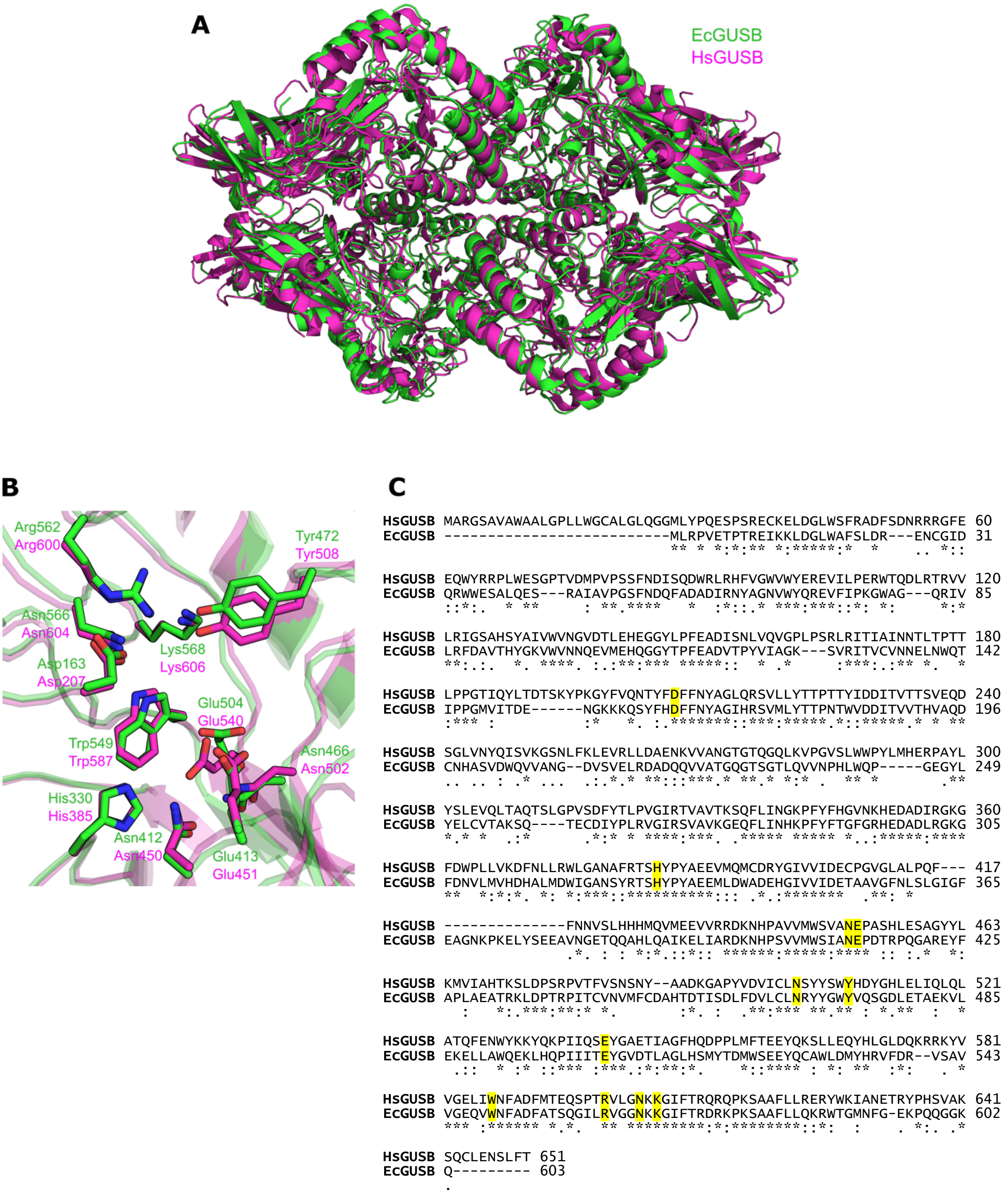


**Figure S3** Homology between CAZy GH2(1) enzymes HsGUSB and EcGUSB. **A**) Superposed EcGUSB and HsGUSB tertiary/quaternary structures, showing the close similarity between these two enzymes. **B**) Overlay of active sites of EcGUSB and HsGUSB, showing full conservation of active site residues. **C**) Sequence homlogy between HsGUSB and EcGUSB. Active site residues are highlighted in yellow.

**A**

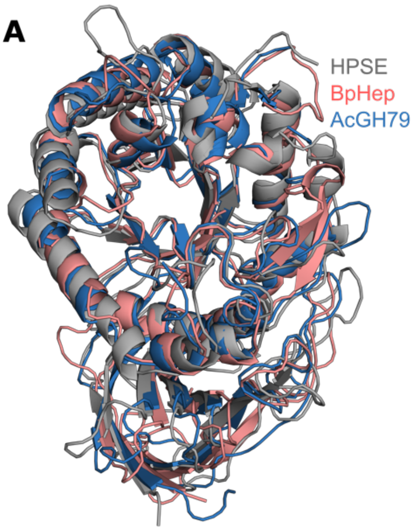


**B**

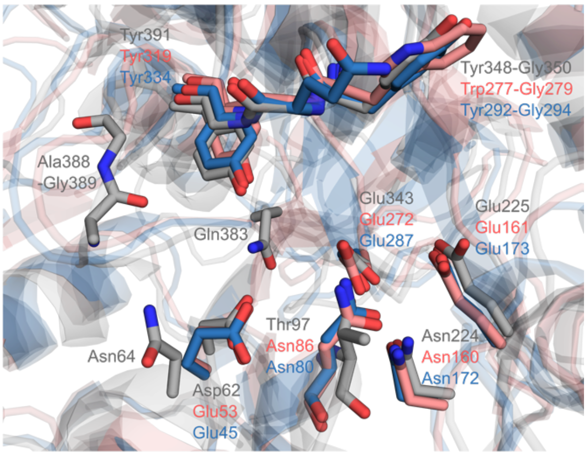


**C**

```
HPSE      MLLRSKPALPPPLMLLLLGPLGPLS--PGA--LPRPAQAQDVVDLDFFTQEPLHLVSPSF 56
BpHep     -------------MLAACGGGGGADAAAAAPQQPPPAGPSSSANVAMTLPADAPRIARDF 47
AcGH79    ---------------MAFARGGLAQTASQTTSSPVRVG------LSVDASALGHTIPPDY 39
                            .  *  .    :   *  .       : .        :  .:

HPSE      LSVTIDANLATDPRFLILLGSPKLRTLARGLSPAYLRFGGTKTDFLIFDPKKESTFEERS 116
BpHep     AGLSIEKAALSYP-LLSGENGNMVGLFNRLG-AGVLRIGGNSSDAS-------------- 91
AcGH79    TGLSYEQAQMANPNYFSGANTQLAGFLRTLGRQGVLRIGGNTSEYT-------------- 85
           .:: :    : *  :   .      :      . **:**..::

HPSE      YWQSQVNQDICKYGSIPPDVEEKLRLEWPYQEQLLLREHYQKKFKNSTYSRSSVDVLYTF 176
BpHep     GWQRTGPD-----------------------------------ETSGVITPAAVDRLASF 116
AcGH79    FWNRHAKPTAAD---------EHLAAG-P--------DKGHHAAAREVITPEAVNNLSEF 127
           *:                                            . :  :*: *  *

HPSE      ANCSGLDLIFGLNALLRTADLQWNSSNAQLLLDYCSSKGYNISWELGNEPNSFLKKADIF 236
BpHep     VQACRWRVIYGLNFVGNDPATIADE--AAY-AAQALGV-QLAGFEIGNEPDLYAQHGLAP 172
AcGH79    LDKTGWKLIYGLNLGKGTPENAADE--AAY-VMETIGADRLLAFQLGNEPDLFYRNGIRP 184
           :     :*:***          :.  *        .     .:::****: : ::.

HPSE      ING--SQLGEDFIQLHKLLRKSTFKNAKLYGPDVGQPRRKTAKMLKSFLKAGGEVIDSVT 294
BpHep     NANTYPGFVSRWTTFANAIRA-AVPDAVFTGPATAWNYQ---RYTVPFASDAAGLVSLL- 227
AcGH79    ASYDFAAYAGDWQRFFTAIRK-RVPNAPFAGPDTAYNT----KWLVPFADKFKHDVKFI- 238
                     :  : . :*   . :* : ** ..       :    * .     :. :

HPSE      WHHYYLNGRT----ATKEDFLNPDVLDIFISSVQKVFQVVESTRPGKKVWLGETSSAYGG 350
BpHep     TQHHYRN----PDSATIEAMLSPDPSLA--PMLQ-ALQGA-ASARGIGFRLAETNSYWGG 279
AcGH79    SSHYYAEGPPTDPSMTIERLMKPNPRLL--GETA-GLKQV-EADTGLPFRLTETNSCYQG 294
            *:* :        * * ::.*:            :: .  :  *  . * **.* : *

HPSE      GAPLLSDTFAAGFMWLDKLGLSARMGIEVVMRQVFFGAGNYHLVDEN----F--DPLPDY 404
BpHep     GKPGVSDAHASALWVINFLFAVAQGGASG-VNLHTGGGASYSAIKTNKTAGTVAAIGPEY 338
AcGH79    GKQGVSDTFAAALWAGDLMYQQAAAGSTG-INFHGGGYGWYTPVAGTPEDGF--IARPEY 351
          *   :**:.*:.:   : :   *  *    :.    * . *  :  .          *:*

HPSE      WLSLLFKKLVGTKVLMASVQGSKRRKLRVYLHCTNTDNPRYKEGDLTLYAINLHNVTKYL 464
BpHep     YGIYLFNQAAGGRLMQTRVDSAGTTL---FAHAVAAD-----GGGVRLILVNTDANSGYD 390
AcGH79    YGMLLFAQAGAGQLLGAKLTDNSAAPL-LTAYALRGT-----DGRTRIALFNKNLDADVE 405
          :   ** :  . ::: : : .          :.          *   :  .* .  :

HPSE      -RLPYPFSNKQVDKYLLRPLGPHGLLSKSVQLNGLTLKMVDDQTLPPLMEKPLRPGSSLG 523
BpHep     VAVDCSSVPNARAGIVTTLGGPSLGSLTGTQIDGATFALD--GSGAPQGGRPVAC----- 443
AcGH79    VAISGVASP---SGTVLRLEAPRADDTTDVTFGGAPVGAS--GSWSPLVQEYVPG----- 455
                 :             :   .*     ... :.*  .      :  *   . :

HPSE      LPAFSYSFFVIRNAKVAACI---- 543
BpHep     ---VN-GVLGVHVASASALLVDFA 463
AcGH79    ---HS-GQFVLHMRKASGALLEFA 475
              . . : ::  ..:. :
```

**Figure S4** Homology between CAZy GH79 (1) enzymes HPSE, BpHep and AcGH79. **A**) Superposed enzyme tertiary structures. **B**) Overlay of active site residues between HPSE, BpHep and AcGH79. Some residues involved in substrate interaction are unique to HPSE and have no clear structural homology in the other enzymes. **C**) Sequence homlogy between HPSE, BpHep and AcGH79. Conserved active site residues are highlighted in yellow. HPSE unique active site residues are highlighted in green. The 6-kDa linker peptide of HPSE is highlighted in grey.

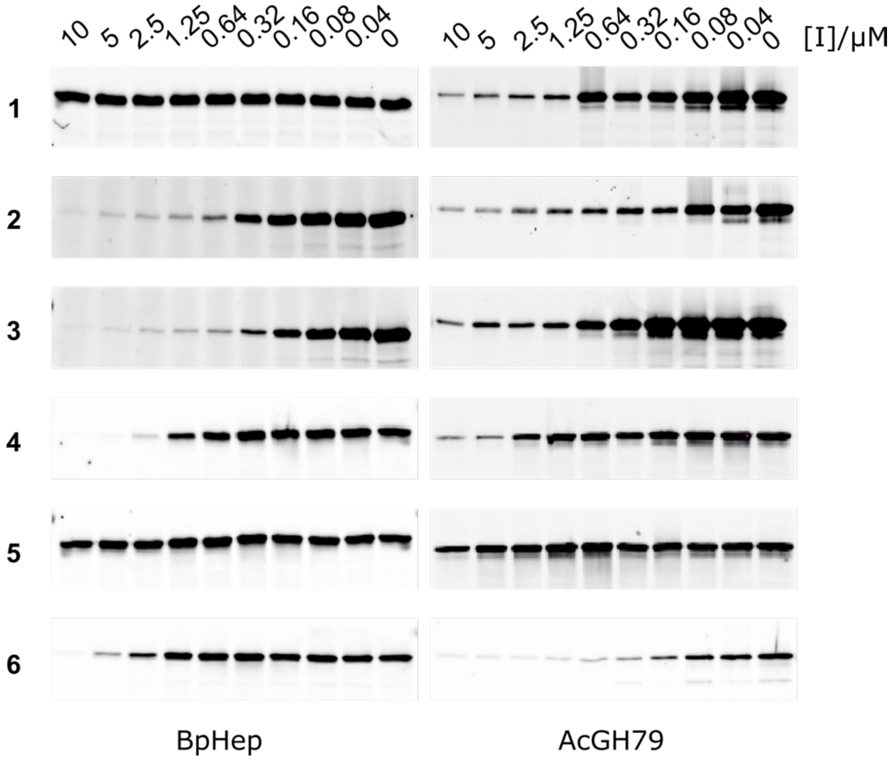


**Figure S5** cABPP gels for BpHep and AcGH79 inhibition by **1**-**6**. Full length gels are shown in **Figure S8**.

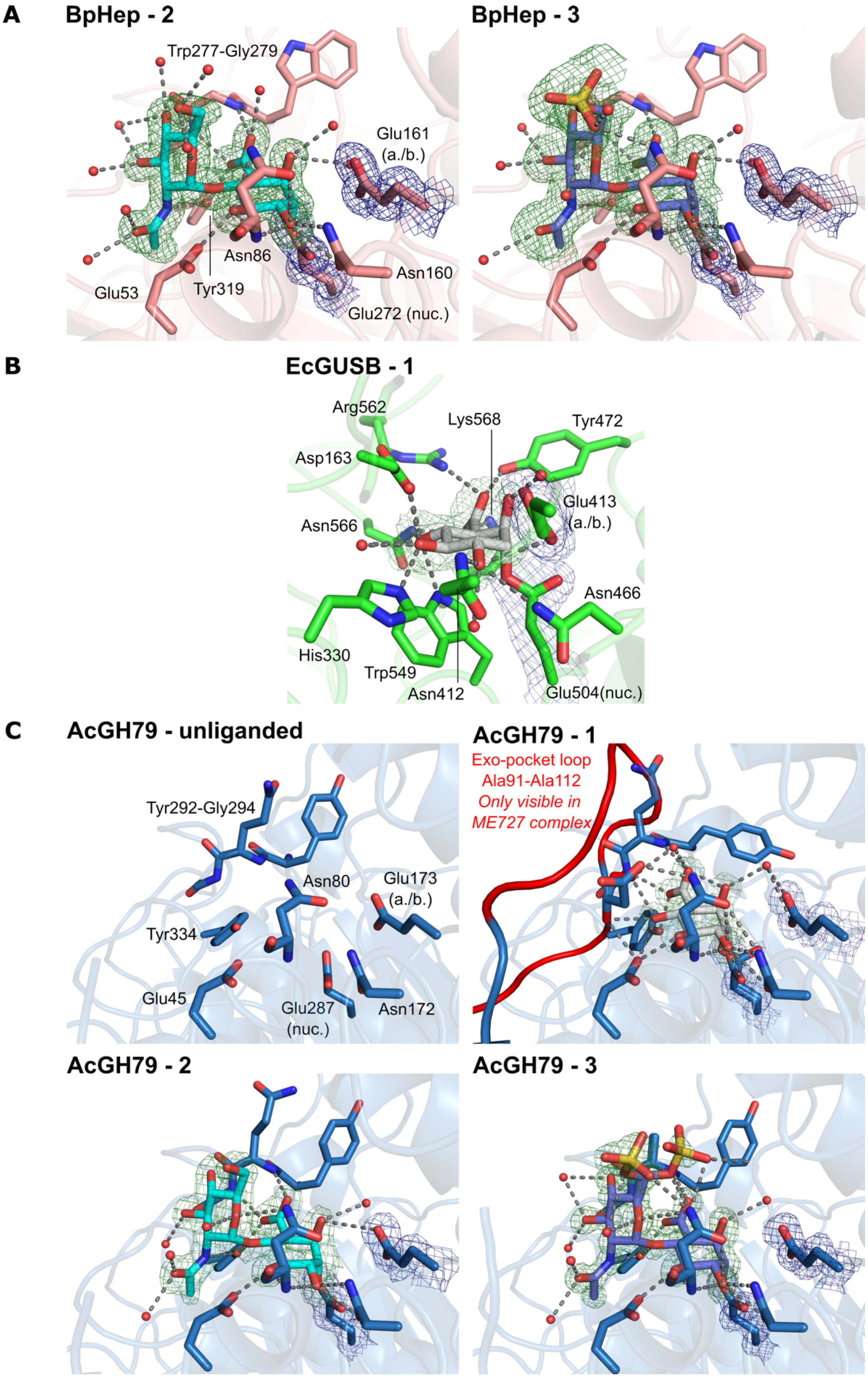


**Figure S6** Active-site views of BpHep (**A**), EcGUSB (**B**), and AcGH79 (**C**) in complex with cyclophellitol-derived inhibitors. Electron density for sidechains is REFMAC $\sigma_A$-weighted 2mFo-DFc, contoured to 1$\sigma$ (0.21–0.51 $e^{-}.Å^{-3}$). Electron density for ligands is REFMAC $\sigma_A$-weighted mFo-DFc, contoured to 3$\sigma$ (0.2–0.54 $e^{-}.Å^{-3}$). An otherwise unstructured loop in the AcGH79 active site is only visible in the complex with pseudo-monosaccharide **1**.

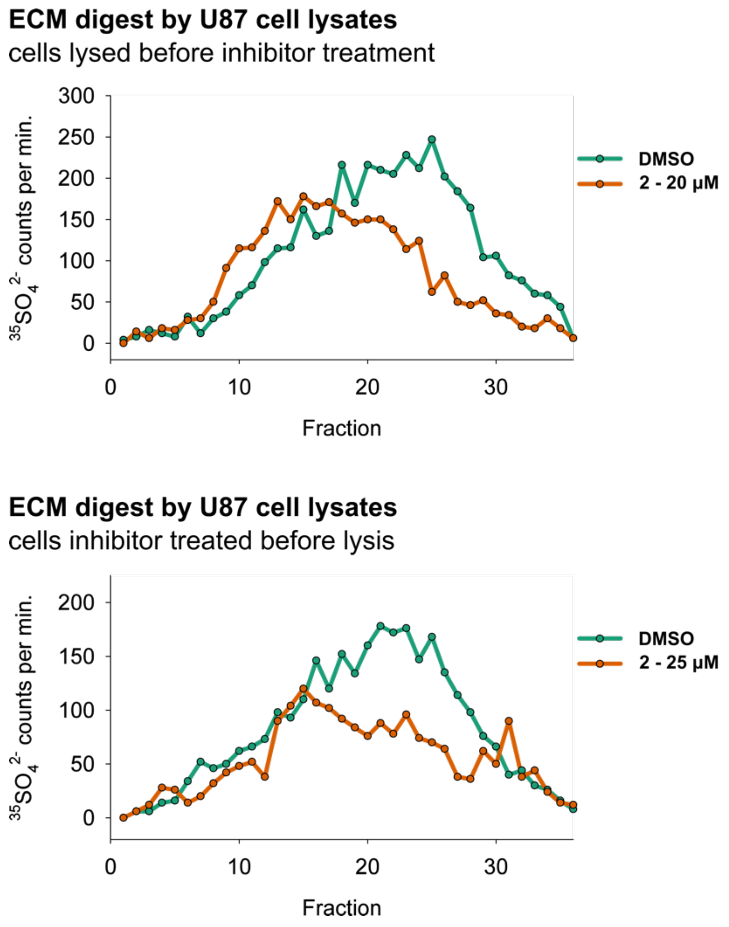


**Figure S7** Radiolabelled $H^{35}SPG$ basement membrane digests by HPSE expressing U87 cell lysates show comparable profiles of $H^{35}S$ solubilization whether inhibitor **2** is applied before or after cell lysis. These data indicate that **2** can penetrate cells to inhibit the intracellular HPSE pool.

Figure 2A, **1**-HPSE

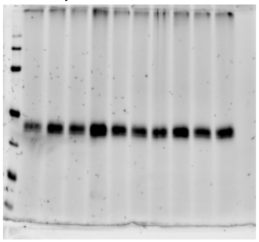

Figure 2A, **2**-HPSE

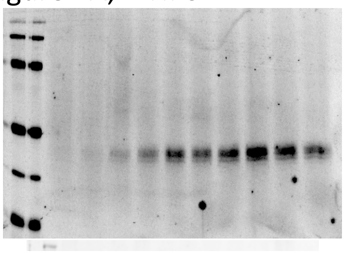

Figure 2A, **3**-HPSE

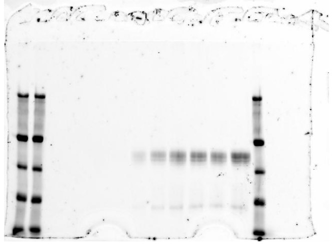

Figure 2A, **4**-HPSE

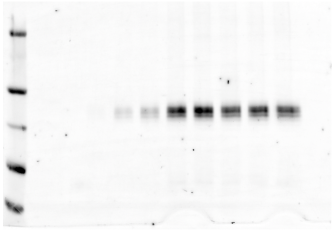

Figure 2A, **5**-HPSE

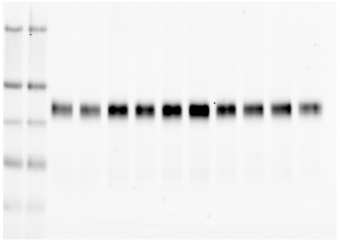

Figure 2A, **6**-HPSE

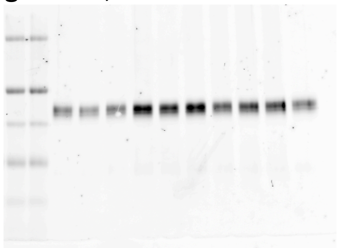

Figure 2A, **1**-proHPSE

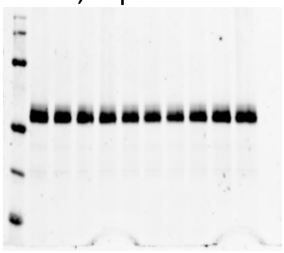

Figure 2A, **2**-proHPSE

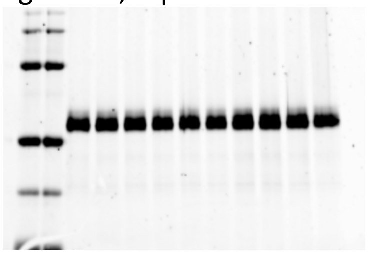

Figure 2A, **3**-proHPSE

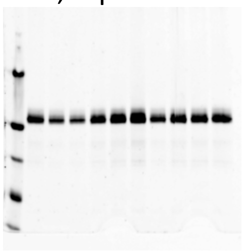

Figure 2A, **4**-proHPSE

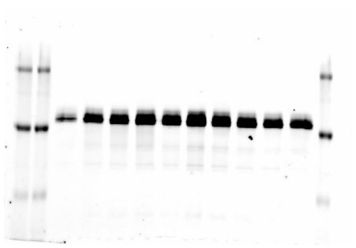

Figure 2A, **5**-proHPSE

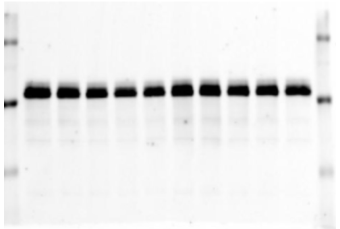

Figure 2A, **6**-proHPSE

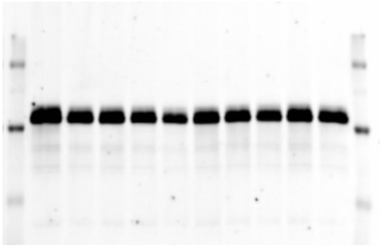

Figure 2A, **1**-EcGUSB

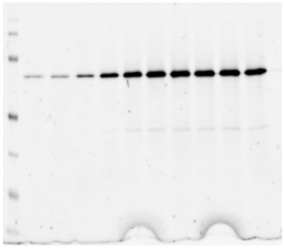

Figure 2A, **2**-EcGUSB

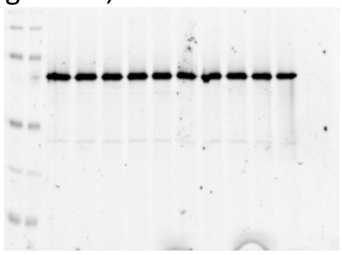

Figure 2A, **3**-EcGUSB

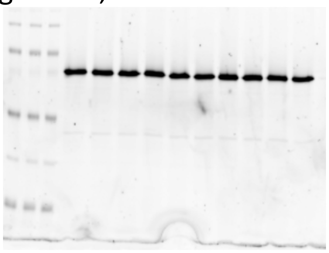

Continued…

Figure 2A, **4**-EcGUSB

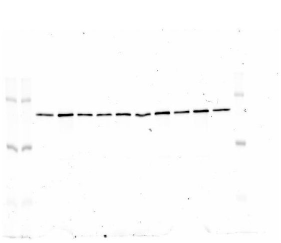

Figure 2A, **5**-EcGUSB

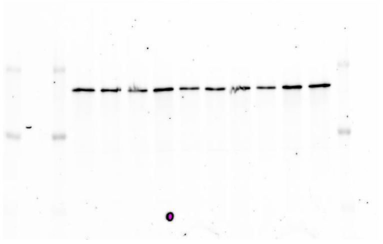

Figure 2A, **6**-EcGUSB

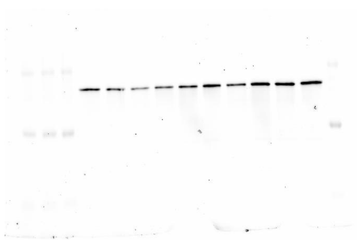

Figure 3A, **1**

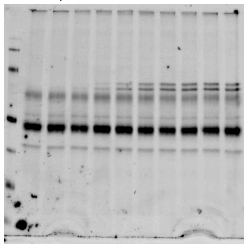

Figure 3A, **2**

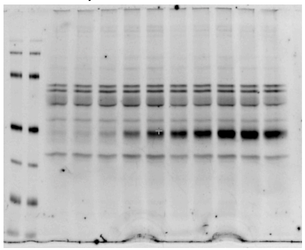

Figure 3A, **3**

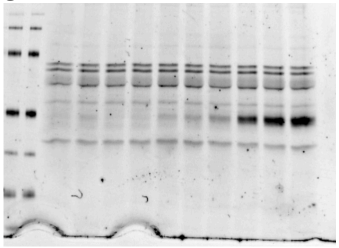

Figure 3B, **7** (1 μM)

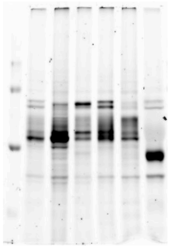

Figure 3B, **7** (10 μM)

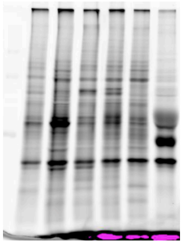

Figure 3B, **8** (1 μM)

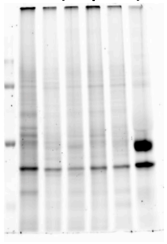

Figure 3B, **8** (10 μM)

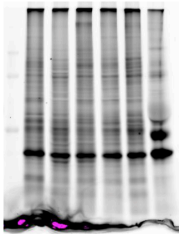

Figure 3B, **9** (1 μM)

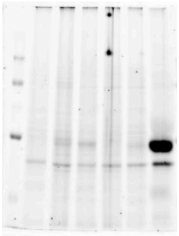

Figure 3B, **9** (10 μM)

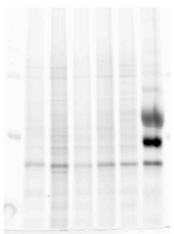

Fig. S4, **1**-BpHep

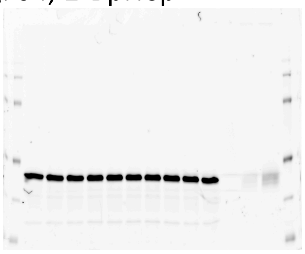

Fig. S4, **2**-BpHep

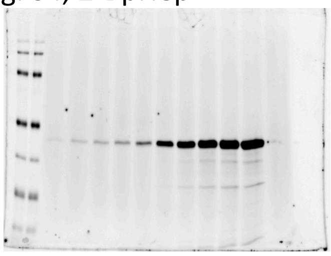

Fig. S4, **3**-BpHep

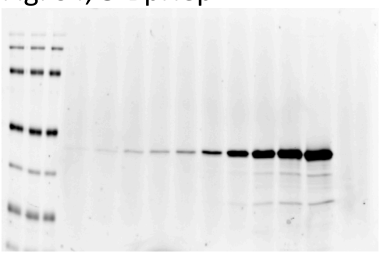

Continued…

Fig. S4, **4**-BpHep

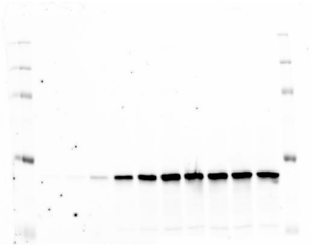

Fig. S4, **5**-BpHep

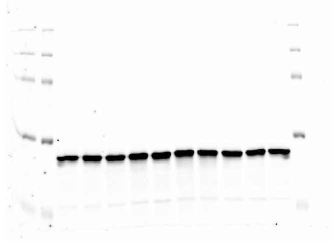

Fig. S4, **6**-BpHep

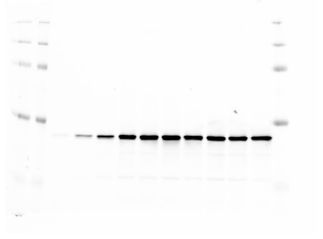

Fig. S4, **1**-AcGH79

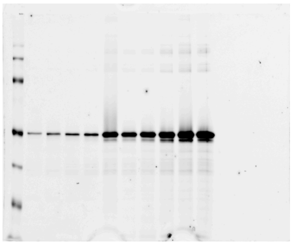

Fig. S4, **2**-AcGH79

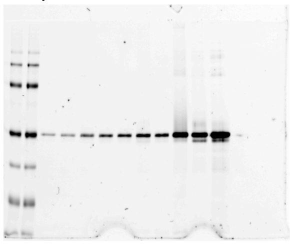

Fig. S4, **3**-AcGH79

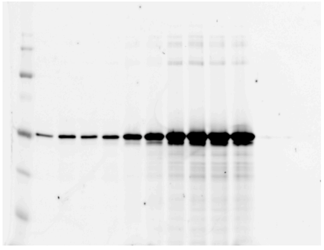

Fig. S4, **4**-AcGH79

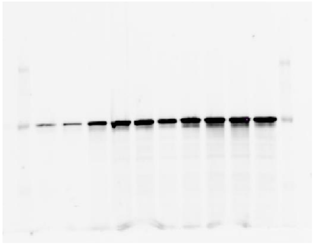

Fig. S4, **5**-AcGH79

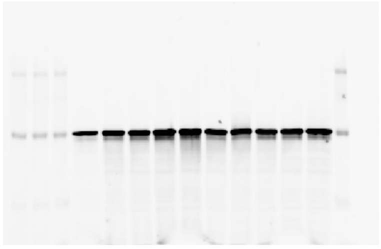

Fig. S4, **6**-AcGH79

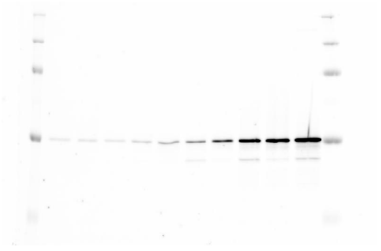

**Figure S8** Full length images of ABP gels shown in this study.

**Table S1**. Data collection and refinement statistics

| | HPSE–**2** (PDB 7PR7) | HPSE–**3** (PDB 7PR8) | HPSE–**4** (PDB 7PRT) | BpHep–**2** (PDB 7PR9) | BpHep–**3** (PDB 7PRB) |
|---|---|---|---|---|---|
| **Data collection** | | | | | |
| Space group | $P2_1$ | $P2_1$ | $P2_1$ | $P2_12_12_1$ | $P2_12_12_1$ |
| *a*, *b*, *c* (Å) | 46.4, 71.0, 79.2 | 46.5, 71.3, 79.1 | 46.8, 71.6, 78.7 | 79.9, 104.1, 114.0 | 81.2, 101.1, 113.6 |
| α, β, γ (°) | 90, 95.1, 90 | 90, 95.2, 90 | 90, 94.9, 90 | 90, 90, 90 | 90, 90, 90 |
| Resolution (Å) | 52.78–1.52 (1.56–1.52) | 39.37–1.66 (1.70–1.66) | 52.89-1.70 (1.73-1.70) | 79.89–1.34 (1.37–1.34) | 66.06–1.31 (1.34–1.31) |
| $R_{meas}$ | 0.06 (1.54) | 0.06 (1.63) | 0.060 (1.07) | 0.06 (1.37) | 0.05 (1.59) |
| $R_{pim}$ | 0.03 (0.74) | 0.03 (0.79) | 0.04 (0.70) | 0.02 (0.61) | 0.02 (0.53) |
| $I / \sigma I$ | 12.3 (1.1) | 12.5 (1.0) | 11.0 (1.1) | 13.4 (1.1) | 15.7 (1.2) |
| Completeness (%) | 99.9 (100.0) | 98.6 (97.9) | 99.8 (99.7) | 98.9 (92.5) | 98.5 (96.5) |
| Redundancy | 4.1 (4.1) | 4.2 (4.2) | 4.1 (3.7) | 7.6 (4.7) | 8.3 (8.6) |
| $CC_{1/2}$ | 0.998 (0.479) | 0.999 (0.532) | 0.999 (0.748) | 0.999 (0.565) | 0.999 (0.682) |
| **Refinement** | | | | | |
| Unique reflections | 74697 | 56894 | 56984 | 199376 | 209063 |
| $R_{work}$ / $R_{free}$ | 0.17/0.19 | 0.17/0.19 | 0.19 / 0.23 | 0.18/0.20 | 0.17/0.19 |
| No. atoms | | | | | |
| Protein | 3682 | 3682 | 3681 | 6495 | 6465 |
| Ligand/ion | 122 | 153 | 115/2 | 76 | 121 |
| Water | 324 | 308 | 91 | 992 | 991 |
| *B*-factors | | | | | |
| Protein | 34.2 | 37.65 | 40.79 | 22.2 | 23.5 |
| Ligand/ion | 53.1 | 51.1 | 65.95/43.19 | 24.7 | 26.1 |
| Water | 43 | 45.9 | 36.36 | 33.5 | 36.1 |
| R.m.s. deviations | | | | | |
| Bond lengths (Å) | 0.012 | 0.011 | 0.009 | 0.014 | 0.014 |
| Bond angles (°) | 1.79 | 1.69 | 1.60 | 1.81 | 1.90 |

*Values in parentheses are for highest-resolution shell.

**Table S1 continued**. Data collection and refinement statistics

| | AcGH79 (Apo) (PDB 7PSH) | AcGH79–**1** (PDB 7PSI) | AcGH79–**2** (PDB 7PSJ) | AcGH79–**3** (PDB 7PSK) | EcGUSB-**1** (PDB 7PR6) |
|---|---|---|---|---|---|
| **Data collection** | | | | | |
| Space group | $I2_1$ | $I2_1$ | $I2_1$ | $I2_1$ | C2 |
| *a*, *b*, *c* (Å) | 82.9, 44.1, 136.3 | 82.6, 44.5, 136.7 | 83.4, 44.1, 136.7 | 82.8, 44.6, 136.9 | 167.8, 76.6, 125.7 |
| $\alpha$, $\beta$, $\gamma$ (°) | 90.0, 97.6, 90.0 | 90.0, 97.6, 90.0 | 90.0, 97.6, 90.0 | 90.0, 97.6, 90.0 | 90, 125.01, 90 |
| Resolution (Å) | 74.69-1.24 (1.26-1.24) | 74.69-1.25 (1.27-1.25) | 75.11-1.55 (1.58-1.55) | 67.85-1.09 (1.11-1.09) | 47.76–1.99 (2.04–1.99) |
| $R_{meas}$ | 0.07 (0.67) | 0.10 (0.69) | 0.10 (0.96) | 0.09 (0.51) | 0.16 (1.97) |
| $R_{pim}$ | 0.04 (0.40) | 0.07 (0.46) | 0.06 (0.63) | 0.06 (0.33) | 0.08 (0.99) |
| $I$ / $\sigma I$ | 11.6 (2.2) | 6.6 (1.1) | 7.1 (1.0) | 7.9 (2.0) | 6.5 (0.8) |
| Completeness (%) | 97.2 (91.1) | 98.4 (90.7) | 99.9 (99.9) | 97.6 (89.9) | 97.3 (98.7) |
| Redundancy | 6.1 (4.5) | 3.7 (2.8) | 3.9 (3.7) | 3.8 (2.8) | 3.9 (3.9) |
| $CC_{1/2}$ | 0.998 (0.794) | 0.993 (0.680) | 0.997 (0.793) | 0.994 (0.803) | 0.983 (0.334) |
| **Refinement** | | | | | (TLS refinement) |
| Unique reflections | 134306 | 133776 | 71878 | 201068 | 82582 |
| $R_{work}$ / $R_{free}$ | 0.17/0.19 | 0.17/0.18 | 0.19/0.22 | 0.17/0.18 | 0.19/0.24 |
| No. atoms | | | | | |
| Protein | 3430 | 3491 | 3331 | 3418 | 9580 |
| Ligand/ion | - | 18 | 27 | 38 | 26 |
| Water | 411 | 466 | 360 | 563 | 469 |
| *B*-factors | | | | | |
| Protein | 17.2 | 14.3 | 23.1 | 11.2 | 31.5 |
| Ligand/ion | - | 15.8 | 19.3 | 11.2 | 26.6 |
| Water | 26.0 | 23.6 | 30.6 | 22.5 | 44.6 |
| R.m.s. deviations | | | | | |
| Bond lengths (Å) | 0.017 | 0.016 | 0.011 | 0.020 | 0.012 |
| Bond angles (°) | 1.96 | 1.80 | 0.16 | 1.99 | 1.73 |

*Values in parentheses are for highest-resolution shell.

## Methods

### Recombinant protein production and purification

*HPSE* – Human HPSE was expressed and purified according to previously reported procedures (2).

*proHPSE* – Human proHPSE was expressed and purified according to previously reported procedures (3).

*AcGH79* – AcGH79 was expressed and purified according to previously reported procedures (3).

*BpHep* – The coding sequence of BpHep was cloned into the pET28a vector (Novagen), behind an N-terminal 6xHis tag and thrombin cleavage site, and used to transform *E. coli* strain BL21 Gold (DE3) (Agilent). Transformants were grown in TB media supplemented with 50 μg/mL kanamycin at 37 °C until cultures reached an OD600 of 0.8-1.0, whereupon expression was induced by the addition of 0.5 mM isopropyl β-D-1-thiogalactopyranoside (IPTG; Sigma). Induced cultures were grown at 16 °C overnight, then harvested by 4,000 g centrifugation at 4 °C for 15 min.

Harvested cells were resuspended in ~50 mL Histrap buffer A (20 mM Tris pH 8.0, 500 mM NaCl, 20 mM imidazole, 1 mM DTT), supplemented with DNAse I (Sigma; bovine pancrease), and cOmplete™ EDTA protease inhibitors (Roche). Cells were lysed using a cell disruptor (Constant systems) at 40 kPSI operating pressure, and lysate clarified by centrifugation at 40,000 g at 4 °C for 30 min. Clarified supernatant was loaded onto a 5 mL HisTrap FF crude column (Cytiva) pre-equilibrated with HisTrap buffer A, washed with 10 column volumes (CV) of HisTrap buffer A, before eluting with HisTrap buffer B (20 mM Tris pH 8.0, 500 mM NaCl, 1000 mM imidazole, 1 mM DTT) over a 20 CV linear gradient. BpHep containing fractions were pooled, and buffer exchanged into 20 mM HEPES pH 7.4, 100 mM NaCl by at least 3 rounds

of sequential concentration/dilution using a 30 kDa molecular weight cut-off (MWCO) Vivaspin centrifugal concentrator (Cytiva). Buffer exchanged BpHep was digested overnight at ambient temperature with thrombin (Sigma; bovine plasma) at 1:100 mass ratio thrombin:BpHep.

Digested BpHep was rerun over a 5 mL HisTrap FF crude column pre-equilibrated with HisTrap buffer A, which was further washed with 3 CV of Histrap buffer A. Combined flowthrough and wash fractions were concentrated to ~2 mL volume using a 30 kDa MWCO Vivaspin centrifugal concentrator, then loaded onto a Superdex S75 16/600 pg size exclusion chromatography (SEC) column (Cytiva) pre-equilibrated in SEC buffer (20 mM HEPES pH 7.4, 200 mM NaCl, 1 mM DTT). BpHep containing fractions were pooled and concentrated using a 30 kDa MWCO Vivaspin centrifugal concentrator to a final concentration of ~20 mg/ml. Purified protein was flash frozen in liquid nitrogen (LN2) and stored at –80 °C for use in downstream experiments.

*EcGUSB* - The coding sequence of EcGUSB was cloned into the pET28a vector, behind an N-terminal 6xHis tag, and used to transform *E. coli* strain BL21 Gold (DE3). Transformants were grown in TB media supplemented with 50 μg/mL kanamycin at 37 °C until cultures reached an OD600 of 0.8-1.0, whereupon gene expression was induced by the addition of 0.5 mM IPTG. Induced cultures were grown at 16 °C overnight, then harvested by 4,000 g centrifugation at 4 °C for 15 min.

Harvested cells were resuspended in ~50 mL Histrap buffer (20 mM Tris pH 8.0, 500 mM NaCl, 20 mM imidazole, 1 mM DTT), supplemented with DNAse I, and cOmplete™ EDTA protease inhibitors. Cells were lysed using a cell disruptor at 40 kPSI operating pressure, then lysate clarified by centrifugation at 40,000 g at 4 °C for 30 min. Clarified supernatant was loaded onto a 5 mL HisTrap FF crude column pre-equilibrated with HisTrap buffer A, washed with 10 CV of HisTrap buffer A, before eluting with HisTrap buffer B (20 mM Tris pH 8.0, 500 mM NaCl,

1000 mM imidazole, 1 mM DTT) over a 20 CV linear gradient. BpHep containing fractions were pooled, and concentrated using a 30 kDa MWCO Vivaspin centrifugal concentrator to a volume of ~ 2 mL. Concentrated protein was loaded onto a Superdex S200 16/600 pg SEC column pre-equilibrated in SEC buffer (20 mM HEPES pH 7.4, 200 mM NaCl, 1 mM DTT). EcGUSB containing fractions were pooled and concentrated using a 30 kDa MWCO Vivaspin centrifugal concentrator to a final concentration of ~24.5 mg/ml. Purified protein was flash frozen in LN2 and stored at -80 °C for use in downstream experiments.

**Cell samples**

Platelets were isolated from whole blood as described previously (3), or purchased from Innovative Research (Novi, MI, USA). Approval for tissue collection was obtained from the University of York medical ethics committee.

Cell lines (BT20, PANC1, SKBR3, MDA-MB-453, HT-1080) were obtained from the American type culture collection (ATCC), and cultured at 37°C, 5% $CO_2$ according to the given protocols. All cells were confirmed negative for mycoplasma before use in experiments.

Cell lysates were prepared as described previously (3), and total protein concentrations determined by the Bradford assay using bovine serum albumin (BSA) as a standard. Aliquots of lysates were flash frozen in LN2, and stored at –80 °C until use.

**ABP Labelling experiments**

ABP labelling reactions were carried out in McIlvaine buffer (pH 5.5), 300 mM NaCl. For recombinant proteins 200 fmol of EcGH2, BpHep, AcGH79, HPSE or proHPSE was used (20 nM in 10 µl final reaction volume). For labeling cell lysates, 4–20 µg total protein was used in a 10 µL final reaction volume. Labeling reactions were initiated by the addition of ABP **7**, **8** or **9** to a final concentration of 100 nM (unless otherwise specified), before incubation for 30 min at 37°C with shaking. Following labeling, samples were denatured by boiling with Laemmli buffer

for 5 min and resolved by SDS-PAGE. Gels were scanned using Typhoon-5 laser scanner platform (Cytiva), using the $\lambda_{EX}$ 635 nm laser and 670BP30 emission filter.

For competitive ABPP (cABPP) experiments, protein mixtures were preincubated with inhibitor (or buffer only control) for 1 h at 37°C with shaking, before the addition of **7** to a final concentration of 100 nM, and subsequent incubation for 30 min at 37°C with shaking. SDS-PAGE and analysis steps were carried out as above. For quantitative measurements, fluorescently labeled band intensities were calculated using Imagequant (Cytiva), and normalized to the corresponding band in the inhibitor free control lane. cABPP plots were prepared and analyzed using SigmaPlot 14.0 (Systat)

Full-length images of all fluorescent gels used in this study can be found in **Figure S8**.

**Protein Crystallization**

*HPSE* – Well diffracting crystals of HPSE were obtained by the sitting-drop vapor-diffusion method at 20 °C using a well solution containing 0.1 M MES pH 5.5, 0.1 M $MgCl_2$, 17 % (w/v) PEG 3350, and a protein to well solution ratio of 200 nl: 500 nl. Crystals typically appeared after 1 week.

Inhibitor ligand complexes were obtained by transferring HPSE crystals to drops of well solution supplemented with 25 % (v/v) ethylene glycol and 1–5 mM inhibitor. Crystals were incubated with ligand for ~2–4 h, then directly harvested and flash-cooled in LN2 for data collection.

*AcGH79* – Well diffracting crystals of AcGH79 were obtained by the sitting-drop vapor-diffusion method at 20 °C using a well solution containing 0.5 M ammonium sulfate, 1 M lithium sulfate, 0.1 M trisodium citrate, and a protein to well solution ratio of 500 nl: 500 nl. Crystals typically appeared after 1 week.

Inhibitor complexes were obtained by adding inhibitor at 1 mM concentration directly to crystallization droplets of pre-formed crystals. Crystals were incubated for 24 hours with the inhibitors, then cryo-protected using 2 M lithium sulfate, harvested and flash-cooled in LN2 for data collection.

*BpHep* – Well diffracting crystals of BpHep were obtained by the sitting-drop vapor-diffusion method at 20 °C using a well solution containing 0.1 M sodium citrate pH 5.0, 14% (w/v) PEG 6000, and a protein to well solution ratio of 300 nl: 500nl. Crystals typically appeared after 3 days.

Inhibitor ligand complexes were obtained by transferring BpHep crystals to drops of well solution supplemented with 25 % (v/v) ethylene glycol and 1–5 mM inhibitor. Crystals were incubated with ligand for ~2–4 h, then directly harvested and flash-cooled in LN2 for data collection.

*EcGUSB* – Initial crystals of EcGUSB were obtained by the sitting-drop vapor-diffusion method at 20 °C using a well solution containing 0.1 M Bis-Tris propane pH 7.5, 20% (w/v) PEG 3350, 0.2 M $NaNO_3$. These initial crystals were used to prepare a microseed stock using Seed Beads (Hampton), which was then used to seed optimized crystals of EcGUSB in the same well conditions, at a protein to seed to well solution ratio of 500 nl: 200 nl: 1000 nL.

Inhibitor ligand complexes were obtained by transferring EcGUSB crystals to drops of well solution supplemented with 25 % (v/v) ethylene glycol and 1–5 mM inhibitor. Crystals were incubated with ligand for ~2–4 h, then directly harvested and flash-cooled in LN2 for data collection.

**X-ray data collection and structure solution**

Xray diffraction data were collected at 100 K at beamlines i03 (HPSE-**2**, HPSE-**3**, HPSE-**4**, EcGUSB-**1**, BpHep-**3**), i04 (AcGH79-**2**, BpHep-**2**) and i04-1 (Apo-AcGH79, AcGH79-**1**, AcGH79-

**3**) of the Diamond Light Source UK. Reflections were autoprocessed using the XIA2 pipeline (4). Complexes were solved by directly refining against their unliganded structures where isomorphous, or else solved by molecular replacement with PHASER (5) (search model PDB accessions: 5E98 (HPSE), 3VNY (AcGH79), 3K46 (EcGUSB), 5BWI (BpHep)). Solved structures were iteratively improved by alternating rounds of manual model building and maximum-likelihood refinement using COOT (6) and REFMAC5 (7) respectively. Ligand coordinates were built using jLigand (8). Diagrams were generated using PyMOL.

**Fondaparinux assay**

Fondaparinux cleavage was performed essentially as previously described (9). Briefly, assays were carried out in 96 well plates (Nunc) pretreated with 4% BSA in TBS-T (20 mM Tris pH 7.4, 150 mM NaCl, 0.1% (v/v) Tween 20).

100 μL reactions were set up in 40 mM NaOAc pH 5.0 buffer, containing final concentrations of 200 ng/mL HPSE, 100 μM Fondaparinux (Arixtra) and specified concentrations of inhibitors. Reactions were incubated at 37°C for 18 h, then quenched with 100 μL 0.1 M NaOH supplemented with 1.7 mM WST-1 tetrazolium salt, and incubated at 60°C for 1 h. Absorbance at 584 nm was read using an ELISA plate reader (BIO-TEK Instruments). All reactions were carried out in technical duplicate. Data were plotted and analyzed using Sigmaplot (Systat).

**HSPG digest assay**

ECM coated 35 mm culture dishes containing $^{35}S$ labelled HS were prepared as previously described (10). Briefly, bovine corneal endothelial cells were plated at an initial density of $2x10^5$ cells/ml, with 4% dextran T-40 (Sigma) included in the growth medium. $Na_2{}^{35}SO_4$ (25 μCi/ml) was added to the incubation medium on days 2 and 5 after seeding. On day 12, subendothelial ECM is exposed by dissolving the cells with PBS containing 0.5% Triton X-100 and 20 mM $NH_4OH$, followed by four washes with PBS.

For HS-degradation assays, ECM coated dishes were incubated with 200 ng recombinant HPSE in 1 mL reaction mixture (10 mM buffer Phosphate-Citrate pH 6.0, 50 mM NaCl, 1 mM DTT, 1 mM $CaCl_2$) containing HPSE inhibitors or DMSO control. Plates were incubated at 37°C for 5 h, then assay medium collected and applied to a 0.9×30 cm Sepharose 6B size exclusion column. The Sepharose 6B column was eluted with PBS, and $^{35}S$ radioactivity of 0.2 mL fractions quantitated using a liquid scintillation analyzer (Packard BioScience) (11). Digest assays with U87 cell lysates were carried out in a similar fashion, except plates were incubated for 18 h with cell extracts prepared from $1x10^6$ U87 glioma cells lysed by 3x freeze/thaw cycles.

**Matrigel invasion assay**

$1x10^5$ U87 cells in 200 μL DMEM media (without serum) were seeded onto the top chamber of a Matrigel-coated Boyden transwell filter, alongside 20 μM HPSE inhibitor (or PBS control). Cells in the upper chamber were separated by Matrigel from a lower chamber containing 600 μL DMEM, supplemented with 10% FBS. Plates were incubated at 37 °C for 4 h, before fixing cells in 4% (v/v) paraformaldehyde, and staining with 0.5% (w/v) crystal violet. Matrigel and non-migrating cells in the upper side of the Boyden filter were removed by swabbing, then invading cells on the bottom side of the transwell filter imaged for analysis. 10 randomly selected microscopic fields were recorded, and cell invasion calculated using ImageJ, by quantitating the percentage of the field stained by crystal violet. Graphs were plotted using RStudio.

**Metastasis experiments**

*B16 melanoma* - Twenty minutes before tumor cell inoculation, C57BL/J6 mice (n=5) were injected intraperitoneally with vehicle alone (PBS), compound **2** (300 nmol/mouse), or SST0001 (150 μg/mouse). Mice were subsequently intravenously inoculated with $1.5x10^5$

B16-BL6 melanoma cells/mouse in a volume of 100 μL, alongside 20 μM **2** or 10 μg SST0001. Mice were sacrificed after 14 days and the lungs were dissected, photographed and evaluated for the number of metastatic foci/lung. Plots and statistical analyses were carried out in Prism 9 (Graphpad software).

*4T1 breast cancer* - Twenty minutes before tumor cell inoculation, Balb/c mice (n=5) were injected intraperitoneally with vehicle alone (PBS), compound **2** (300 nmol/mouse), or SST0001 (150 μg/mouse). Mice were subsequently intravenously inoculated with $1.5 \times 10^5$ luciferase tagged 4T1 mouse mammary carcinoma cells/mouse in a volume of 100 μL, alongside 20 μM **2** or 10 μg SST0001.

Bioluminescence imaging of lung metastases produced by luciferase-expressing 4T1 cells was carried out using a highly sensitive, cooled charge-coupled device camera assembled in a specimen box (IVIS; Xenogen Corp, Hopkinton, MA). Tumor bearing mice were intraperitoneally administered with a luciferase substrate D-luciferin at a dose of 150 mg/kg body weight. The mice were anesthetized and kept in a camera box followed by continuous exposure to isoflurane (EZAnesthesia, Palmer, PA, USA). The tumor burden was quantified using Living Image software (Xenogen) (12, 13). Plots and statistical analyses were carried out in Prism 9 (Graphpad software).

*CAG myeloma* - Luciferase-labeled CAG human myeloma cells ($3 \times 10^6$) were injected into the tail vein of NOD/SCID mice. 3-5 days after cell inoculation, mice were randomly assigned to 4 cohorts (6 mice each) receiving: (a) vehicle, (b) compound **2** (i.p. 300 nmol/day), (c) bortezomib (0.5 mg/kg twice weekly) or (d) **2** plus bortezomib (dosing as for individual compounds). Tumor development was inspected (every 7 days) by IVIS imaging as detailed above. Tumor burden was quantified using Living Image software. Plots and statistical analyses were carried out in Prism 9 (Graphpad software).

# Detailed Synthesis Procedures

## Schemes for synthesis of inhibitors and probes

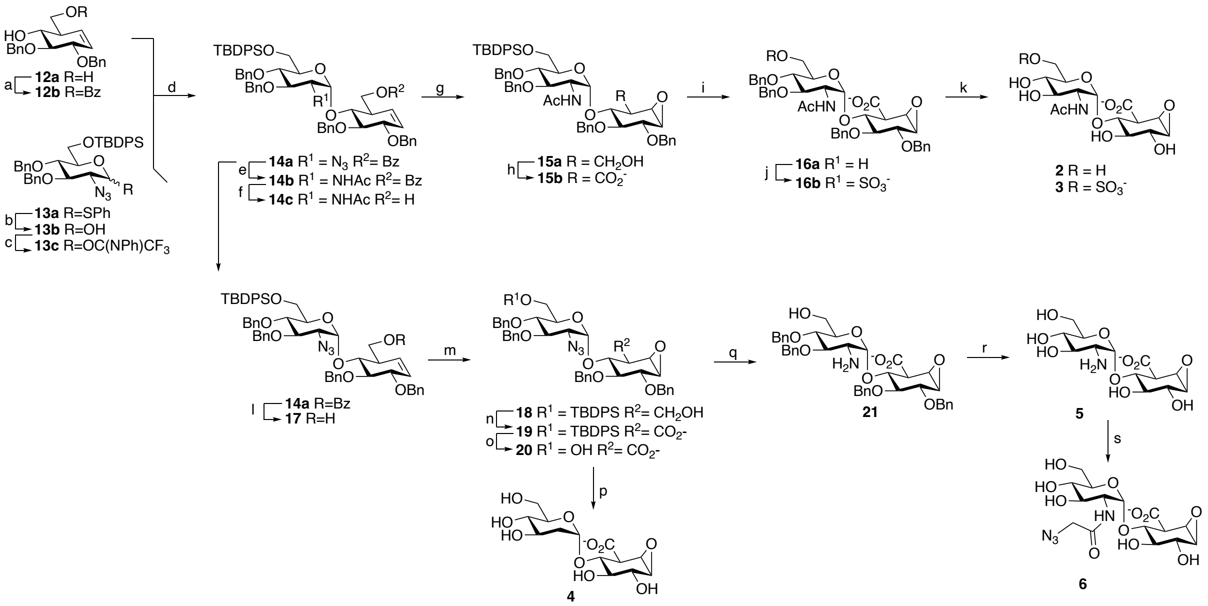

**Scheme 1** a) BzCl, $Et_3N$, DCM, 0°C, 82%; b) i. NIS, acetone/$H_2O$, 68%; c) N-phenyltrifluoroacetimidoylchloride, $Cs_2CO_3$, DCM, 90%; d) TfOH, DCM, -78°C → -30°C, 83%; e) i. $PPh_3$, $H_2O$, THF, 50°C; ii. $Ac_2O$, pyridine, 89%; f) NaOMe, MeOH, DCM, 97%; g) i. $Boc_2O$, DMAP, THF, 76%; ii. NIS, AcOH, 91%; iii. NaOMe, MeOH, DCM, quant.; h) TEMPO, BAIB, DCM, t-BuOH, $H_2O$, 80%; i) $3HF{\cdot}Et_3N$, $Et_3N$, THF, 87%; j) $SO_3{\cdot}Et_3N$, DMF. k) Na(s), $NH_3$, t-BuOH, THF, 91% for **2**, 87% for **3**; l) NaOMe, MeOH, DCM, quant.; m) i. $Boc_2O$, DMAP, THF, 82%; ii. NIS, AcOH, DCM, 71%; iii. NaOMe, MeOH, DCM, 94%; n) TEMPO, BAIB, DCM, t-BuOH, $H_2O$, quant.; o) $3HF{\cdot}Et_3N$, THF, 86%; p) Na(s), $NH_3$, t-BuOH, THF, quant. for **4;** q) Zn(s), $NH_4Cl$, MeOH, toluene; r) Na(s), $NH_3$, t-BuOH, THF, 51% over 2 steps for **5**; s) $N_3AcOH$, DIC, $Et_3N$, 2,3,4,5,6-pentafluorophenol, DMF, 12%.

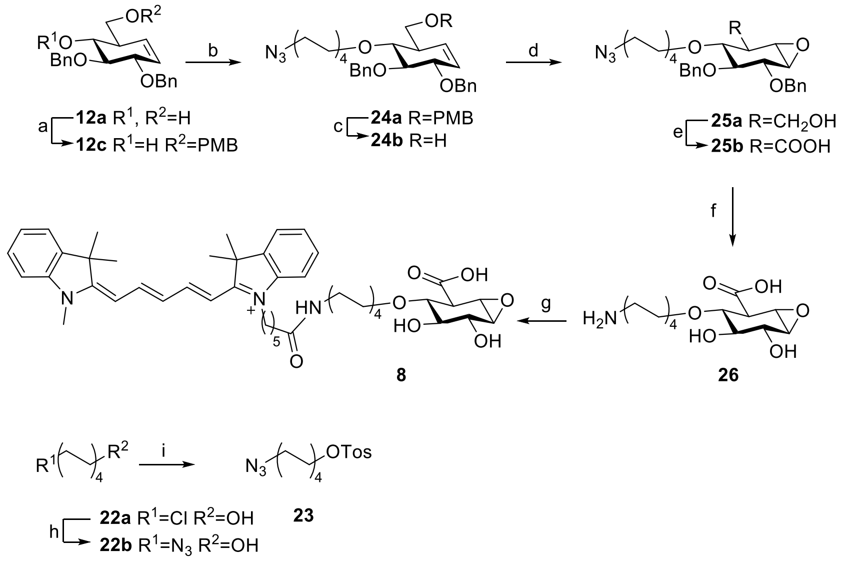


**Scheme 2** a) PMB-Cl, KI, $K_2CO_3$, ACN, 60°C, 73%. b) **23**, KHMDS, THF, 71%. c) DDQ, DCM, $H_2O$, 85%. d) mCPBA, DCM, 87%. e) TEMPO, BAIB, *t*-BuOH, DCM, $H_2O$, 90%. f) Na(s), $NH_3$, *t*-BuOH, THF, 28%. g) Cy5-NHS, DIPEA, DMF, 14%; h) $NaN_3$, DMSO, crude. g) **22b**, TsCl, $Et_3N$, DCM, 92% over 2 steps.

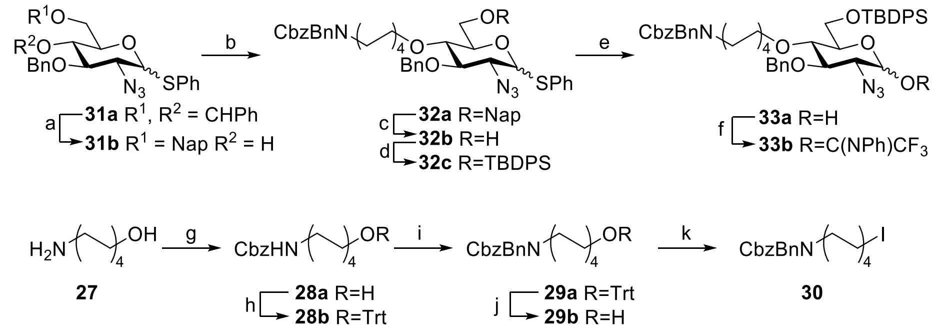


**Scheme 3** a) i. CSA, MeOH, DCE, 50°C; ii. NapBr, 2-aminoethyl diphenyl borinate, MeCN, 60°C, 93%. b) **30**, NaH, DMF, 88%. c) DDQ, DCM, MeOH, 72%. d) TBDPSCl, imidazole, DMF. e) NIS, acetone/$H_2O$, DCM, 79%. f) *N*-phenyl-trifluoroacetimidoylchloride, $Cs_2CO_3$, DCM, 94%. g) CbzCl, $NaHCO_3$, acetone/$H_2O$, 88%. h) TrtCl, $Et_3N$, DMF. **i**) BnBr, NaH, TBAI, DMF, 76% over 2 steps. j) 3% TFA/$H_2O$, DCM/MeOH, 84%; k) $PPh_3$, $I_2$, imidazole, DCM, 97%.

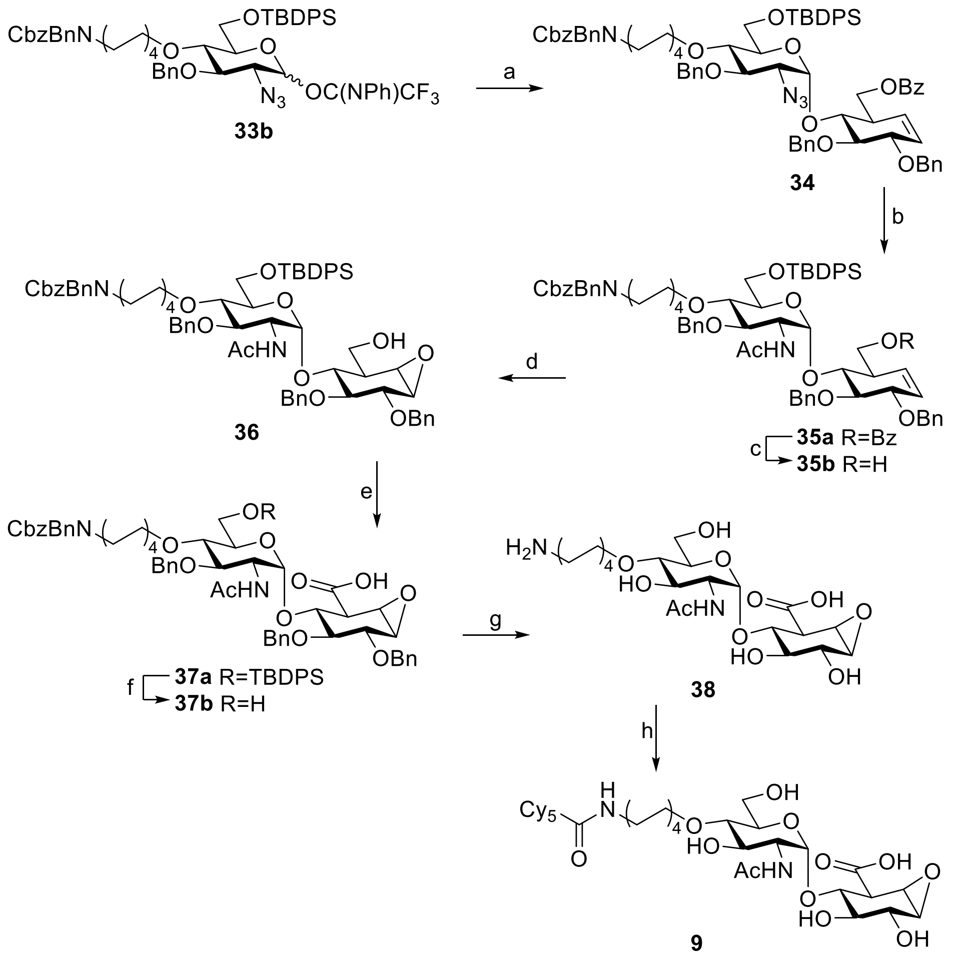


**Scheme 4** a) **12b**, TfOH, DCM, -78°C -> -30°C 97%. b) i. $PPh_3$, $H_2O$, pyridine, THF; ii. $Ac_2O$, pyr, DCM 98%. c) NaOMe, MeOH, DCM, 97%. d) i. $Boc_2O$, DMAP, THF, 82%; ii. NIS, AcOH, DCM, 68%; iii. NaOMe, MeOH, DCM, 88%. e) TEMPO, BAIB, *t*-BuOH, DCM, $H_2O$, 67%. f) $Et_3N{\cdot}3HF$, THF, quant. g) $H_2$, Pd/C, $H_2O$, dioxane, 41%; h) Cy5COOH, pentafluorophenyl trifluoroacetate, DIPEA, DMF, 13%.

## General

Chemicals were purchased from Acros, Sigma Aldrich, Biosolve, VWR, Fluka, Merck, Carbosynth and Fisher Scientific and used as received unless stated otherwise. Tetrahydrofuran (THF), N,N-dimethylformamide (DMF) and toluene were stored over molecular sieves before use. Traces of water from reagents were removed by co-evaporation with toluene in reactions that required anhydrous conditions. All reactions were performed under a nitrogen atmosphere unless stated otherwise. TLC analysis was conducted using Merck aluminum sheets (Silica gel 60 $F_{254}$) with detection by UV absorption (254 nm), by spraying with a solution of $(NH_4)_6Mo_7O_{24}\cdot 4H_2O$ (25 g/L) and $(NH_4)_4Ce(SO_4)_4\cdot 2H_2O$ (10 g/L) in 10% sulfuric acid or a solution of $KMnO_4$ (20 g/L) and $K_2CO_3$ (10 g/L) in water, followed by charring at ~150 ˚C. Column chromatography was performed using Screening Device b.v. silica gel (particle size of 40 – 63 µm, pore diameter of 60 Å) with the indicated eluents. For reversed-phase HPLC purifications an Agilent Technologies 1200 series instrument equipped with a semi-preparative column (Gemini C18, 250 x 10 mm, 5 µm particle size, Phenomenex) was used. LC/MS analysis was performed on a Surveyor HPLC system (Thermo Finnigan) equipped with a $C_{18}$ column (Gemini, 4.6 mm x 50 mm, 5 µm particle size, Phenomenex), coupled to a LCQ Adventage Max (Thermo Finnigan) ion-trap spectrometer ($ESI^+$). The applied buffers were $H_2O$, MeCN and 1% aqueous TFA. $^1$H NMR and $^{13}$C NMR spectra were recorded on a Bruker DPX-300 (300 and 75 MHz respectively), Brüker AV-400 (400 and 101 MHz respectively), a Bruker AV-I-500 (500 and 126 MHz respectively) spectrometer in the given solvent. Chemical shifts are given in ppm (δ) relative to the residual solvent peak or tetramethylsilane (0 ppm) as internal standard. Coupling constants are given in Hz. Assignments of peaks are based upon COSY and HSQC, for numbering see figure 1. High-resolution mass spectrometry (HRMS) analysis was performed with a LTQ Orbitrap mass spectrometer (Thermo Finnigan), equipped with an electronspray ion source in positive mode (source voltage 3.5 kV, sheath gas flow 10 mL/min, capillary temperature 250 °C) with resolution R = 60000 at m/z 400 (mass range m/z = 150 – 2000) and dioctyl phthalate (m/z = 391.28428) as a "lock mass". The high-resolution mass spectrometer was calibrated prior to measurements with a calibration mixture (Thermo Finnigan).

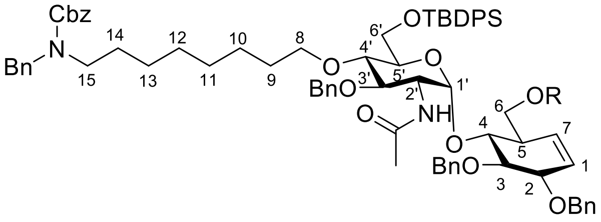


**Figure 1** Numbering used for assignment of NMR data.

### 2-azido-2-deoxy-3,4-di-*O*-benzyl-6-*O*-tert-butyldiphenylsilyl-D-glucopyranoside (13b)

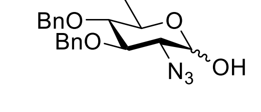


Thioglycoside **13a** (14) (3.58 g, 5.00 mmol) was dissolved in acetone/$H_2O$ (50 ml, 9/1, v/v). The mixture was cooled to 0 °C and NIS (2.25 g, 10.0 mmol) was added. The reaction was stirred for 4 hours. Upon completion $Na_2S_2O_3$ (aq. sat.) was added and the reaction turned colorless. The mixture was concentrated under reduced pressure, the residue was dissolved in EtOAc, washed with $NaHCO_3$ (aq. sat.) and brine, dried over $MgSO_4$, filtered and concentrated under reduced pressure. The product was obtained after chromatography ($Et_2O$/pentane, 1/19 -> 2/8, v/v) as a colorless oil (2.13 g, 3.41 mmol, 68%).

$^1$H NMR (400 MHz, $CDCl_3$) δ 7.79 – 7.59 (m, 7H), 7.42 – 7.24 (m, 23H), 7.23 – 7.12 (m, 3H), 5.23 (t, *J* = 3.4 Hz, 1H), 4.99 – 4.78 (m, 5H), 4.74 (d, *J* = 10.9 Hz, 1H), 4.66 (d, *J* = 10.8 Hz, 1H), 4.43 (dd, *J* = 7.8, 5.1 Hz, 1H), 4.15 – 3.78 (m, 7H), 3.78 – 3.66 (m, 1H), 3.50 – 3.26 (m, 4H), 1.06 (d, *J* = 1.6 Hz, 14H). $^{13}$C NMR (101 MHz, $CDCl_3$) δ 138.1, 137.9, 137.9, 137.8, 136.0, 135.9, 135.7, 135.7, 133.6, 133.6, 133.1, 133.0, 129.8, 129.8, 129.8, 128.6, 128.6, 128.5, 128.4, 128.3, 128.1, 127.9, 127.9, 127.8, 127.8, 127.7, 127.7, 127.7, 127.7, 96.1, 92.1, 83.1, 80.2, 78.3, 77.5, 77.2, 76.8, 76.1, 75.8, 75.8, 75.2, 71.9, 67.6, 64.2, 62.8,

62.5, 60.6, 26.9, 19.4, 19.4. HRMS (ESI) m/z: $[M+Na]^+$ calculated for $C_{36}H_{41}N_3O_5Na$ 646.2708, found 646.2702.

**2-azido-2-deoxy-3,4-di-*O*-benzyl-6-*O*-tert-butyldiphenylsilyl-D-glucopyranosyl-N-phenyltrifluoroacetimidate (13c)**

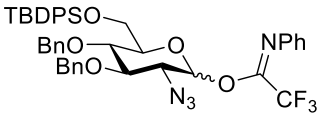


Lactol **13b** (2.10 g, 3.37 mmol) was dissolved in DCM (16.8 ml). 2,2,2-trifluoro-*N*-phenyltrifluoroacetimidoyl chloride (0.15 ml, 0.92 mmol, 1.5 eq) and $Cs_2CO_3$ (1.32 g, 4.04 mmol) were added and the reaction was stirred overnight at room temperature. The reaction was filtered over celite and concentrated in vacuo. Purification by column chromatography (EtOAc/pentane, 1/40 -> 1/19, v/v) yielded the product as a colorless oil (2.41 g, 3.03 mmol, 90%).

$^1$H NMR (400 MHz, $CDCl_3$) δ 7.80 – 7.58 (m, 4H), 7.51 – 7.18 (m, 19H), 7.16 – 7.00 (m, 1H), 6.81 (d, *J* = 7.8 Hz, 1H), 6.76 – 6.71 (m, 1H), 5.00 – 4.82 (m, 3H), 4.76 (dd, *J* = 10.7, 3.9 Hz, 1H), 4.08 – 3.79 (m, 4H), 3.65 (d, *J* = 10.2 Hz, 1H), 1.07 (d, *J* = 1.0 Hz, 9H). $^{13}$C NMR (101 MHz, $CDCl_3$) δ 143.5, 143.3, 137.9, 137.8, 137.7, 137.7, 136.1, 136.0, 135.7, 135.6, 133.6, 133.5, 132.9, 132.8, 130.0, 129.9, 129.9, 129.5, 128.9, 128.8, 128.7, 128.7, 128.7, 128.4, 128.3, 128.2, 128.1, 128.1, 128.0, 127.9, 127.9, 127.8, 126.5, 124.6, 124.5, 120.6, 119.5, 82.9, 80.4, 77.6, 77.5, 77.2, 77.1, 76.8, 76.5, 76.0, 75.5, 75.5, 74.6, 65.6, 63.2, 62.1, 27.0, 26.9, 19.5. HRMS (ESI) m/z: $[M+Na]^+$ calculated for $C_{44}H_{45}F_3N_4O_5SiNa$ 817.3004 found 817.3005.

**2,3-di-*O*-benzyl-6-*O*-benzoyl-cyclophellitol alkene (12b)**

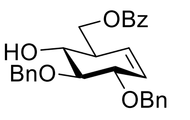


Diol **12a** (1.36 g, 4.00 mmol) was dissolved in DCM (20 ml). $Et_3N$ (2.79 ml, 20.0 mmol) and benzoyl chloride (0.56 ml, 4.80 mmol) were added at -50 °C and the reaction was slowly warmed to room temperature overnight. $H_2O$ and DCM were added and the layers were separated. The organic phase was washed with $NaHCO_3$ (aq. sat.) and brine, dried over $MgSO_4$, filtered and concentrated under reduced pressure. Column chromatography (EtOAc/pentane, 1/19 -> 1/9, v/v) afforded the product (1.45 g, 3.27 mmol, 82%).

$^1$H NMR (400 MHz, $CDCl_3$) δ 8.07 – 7.95 (m, 2H, $H_{arom}$), 7.58 – 7.52 (m, 1H, $H_{arom}$), 7.45 – 7.40 (m, 2H, $H_{arom}$), 7.40 – 7.27 (m, 5H, $H_{arom}$), 5.83 – 5.78 (m, 1H, $H_{alkene}$), 5.73 – 5.68 (m, 1H, $H_{alkene}$), 5.04 (d, J = 11.2 Hz, 1H, $CH_2$-Bn), 4.80 – 4.61 (m, 3H, $CH_2$-Bn), 4.58 (dd, J = 10.9, 3.7 Hz, 1H, H-6a), 4.44 (dd, J = 10.9, 5.5 Hz, 1H, H-6b), 4.26 – 4.20 (m, 1H, H-2), 3.79 – 3.65 (m, 2H, H-3, H-4), 2.80 – 2.76 (d, 1H, H-OH), 2.74 – 2.67 (m, 1H, H-5). $^{13}$C NMR (101 MHz, $CDCl_3$) δ 166.7 (C=O), 133.15, 129.73, 128.72, 128.63, 128.51, 128.11, 128.08, 128.03, 127.99, 127.80, 127.42 ($C_{arom}$, $C_{alkene}$) 83.78 (C-3), 80.37 (C-2), 75.15 (C-Bn), 71.64 (C-Bn), 69.93 (C-4), 64.44 (C-6), 43.43 (C-5). HRMS (ESI) m/z: $[M+Na]^+$ calculated for $C_{28}H_{28}NaO_5$ 467.1829, found 467.1832.

**2,3-di-*O*-benzyl-4-*O*-(2-azido-2-deoxy-3,4-di-*O*-benzyl-6-*O*-tert-butyldiphenylsilyl-α-D-glucopyranosyl)-6-*O*-benzoyl-cyclophellitol alkene (14a)**

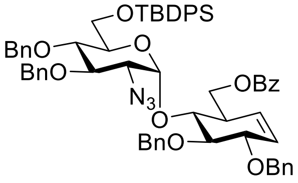


Donor **13c** (2.41 g, 3.03 mmol) and acceptor **12b** (1.04 g, 2.33 mmol) were co-evaporated with anhydrous toluene thrice. the mixture was dissolved in DCM (15 ml) and activated MS 3 Å were added. The mixture was stirred at room temperature overnight. The mixture was cooled to -78 °C. TfOH (0.04 ml, 0.45 mmol) was added and the reaction was warmed to -30 °C in 70 minutes and kept at this temperature for 60 minutes. The reaction was quenched with $NaHCO_3$ (aq. sat.), diluted with $Et_2O$ and washed with $H_2O$, $NaHCO_3$ (aq. sat.), and brine, dried over $MgSO_4$ and filtered. Volatiles were removed under reduced pressure and column chromatography (EtOAc/pentane, 1/19 -> 1/9, v/v) afforded the product (2.02 g, 1.92 mmol, 83%).

$^1$H NMR (400 MHz, $CDCl_3$) δ 7.82 (m, 2H, $H_{arom}$), 7.56 (m, 4H, $H_{arom}$), 7.46 – 7.13 (m, 34H, $H_{arom}$), 5.80 (dt, *J* = 10.1, 2.4 Hz, 1H, $H_{alkene}$), 5.71 (d, *J* = 3.9 Hz, 1H, H-1'), 5.61 (dt, *J* = 10.3, 2.2 Hz, 1H, $H_{alkene}$), 5.11 – 4.95 (m, 2H, $CH_2$-Bn), 4.91 – 4.82 (m, 3H, $CH_2$-Bn), 4.73 – 4.62 (m, 3H, $CH_2$-Bn), 4.46 (dd, *J* = 11.2, 3.3

Hz, 1H, H-6), 4.33 – 4.23 (m, 2H, H-2, H-6), 4.04 – 3.91 (m, 3H, H-3′, H-3, H-4), 3.86 (m, 2H, H-4′/H-5,′ H-6′), 3.75 (m, 2H, H-4′/H-5′, H-6′), 3.29 (dd, *J* = 10.3, 4.0 Hz, 1H, H-2′), 2.77 (m, 1H, H-5), 0.98 (s, 9H, TBDPS). $^{13}$C NMR (101 MHz, $CDCl_3$) δ 139.0, 138.3, 138.5, 136.0, 135.8, 133.7, 133.3, 133.2, 129.9, 129.8, 129.8, 129.7, 128.7, 128.7, 128.7, 128.6, 128.4, 128.2, 128.2, 128.0, 128.0, 127.9, 127.9 ($C_{arom}$, $C_{alkene}$), 127.7 ($C_{alkene}$), 98.3 (C-1′), 84.5 (C-3), 81.1 (C-2), 80.3 (C-3′/C-4), 78.2 (C-4′/C-5′), 75.8, 75.3, 75.0 (C-Bn), 74.5 (C-3′/C-4), 72.9 (C-4′/C-5′), 71.9 (C-Bn), 64.6 (C-6), 63.7 (C-2′), 62.3 (C-6′), 43.4 (C-5), 27.1 (TBDPS), 19.5 (TBDPS quaternary C). HRMS (ESI) m/z: $[M+Na]^+$ calculated for $C_{64}H_{67}N_3O_9SiNa$ 1072.4539, found 1072.4573.

**2,3-di-*O*-benzyl-4-*O*-(2-*N*-acetyl-2-deoxy-3,4-di-*O*-benzyl-6-*O*-tert-butyldiphenylsilyl-α-D-glucopyranosyl)-6-*O*-benzoyl-cyclophellitol alkene (14b)**

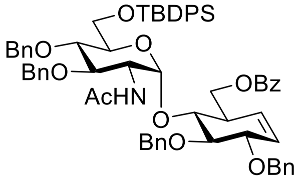

Azide **14a** (0.22 g, 0.21 mmol) was dissolved in THF/$H_2O$ (2.6 ml, 17/3, v/v), $PPh_3$ (0.14 g, 0.50 mmol) and pyridine (5 µl, 0.06 mmol) were added. The solution was stirred at 50 °C for 3 hours. The reaction was concentrated under reduced pressure, dissolved in DCM and dried over $MgSO_4$. The solids were removed by filtration and the filtrate was concentrated under reduced pressure. The residue was co-evaporated with toluene and dissolved in DCM (2.0 ml). Acetic anhydride (0.30 ml, 3.1 mmol) and pyridine (0.25 ml, 3.1 mmol) were added and the reaction was stirred overnight. The reaction was cooled to 0 °C and quenched with $H_2O$. The layers were separated and the organic phase was washed with $CuSO_4$ (aq. sat), $NaHCO_3$ (aq. sat) and brine, dried over $MgSO_4$, filtered and concentrated under reduced pressure. Column chromatography (EtOAc/pentane, 1/9 -> 2/8, v/v) afforded the product (0.20 g, 0.19 mmol, 89%).

$^1$H NMR (400 MHz, $CDCl_3$) δ 7.97 – 7.92 (m, 2H, $H_{arom}$), 7.71 – 7.63 (m, 4H, $H_{arom}$), 7.54 – 7.48 (m, 1H, $H_{arom}$), 7.43 – 7.14 (m, 34H, $H_{arom}$), 6.63 (d, *J* = 9.7 Hz, 1H, NH), 5.83 (dt, *J* = 10.3, 2.5 Hz, 1H, $H_{alkene}$), 5.68 (ddd, *J* = 10.3, 2.8, 1.6 Hz, 1H, $H_{alkene}$), 4.97 – 4.91 (m, 2H, $CH_2$-Bn, H-1′), 4.87 (d, *J* = 10.6 Hz, 1H, $CH_2$-Bn), 4.80 (d, *J* = 11.1 Hz, 1H, $CH_2$-Bn), 4.68 (d, *J* = 10.7 Hz, 2H, $CH_2$-Bn), 4.66 – 4.61 (m, 2H, $CH_2$-Bn), 4.58 (m, 2H, $CH_2$-Bn, H-6), 4.50 (dd, *J* = 11.2, 4.9 Hz, 1H, H-6), 4.39 (td, *J* = 10.0, 3.5 Hz, 1H, H-2′), 4.19 (m, 1H, H-2), 3.98 – 3.85 (m, 5H, H-4′, H-5′, H-6′, H-4), 3.84 – 3.72 (m, 2H, H-3′, H-3), 2.66 (m, 1H, H-5), 1.46 (s, 3H, Acetyl), 1.03 (s, 9H, TBDPS). $^{13}$C NMR (101 MHz, $CDCl_3$) δ 170.5 (C=O), 166.3, 138.8, 138.4, 138.0, 137.8, 136.1, 135.8, 133.8, 133.3, 133.1, 130.1, 129.9, 129.8, 129.8, 128.9, 128.8, 128.7, 128.7, 128.6, 128.5, 128.4, 128.4, 128.3, 128.3, 128.2, 128.1, 128.0, 128.0, 127.9, 127.9, 127.8, 127.8 ($C_{arom}$, $C_{alkene}$), 126.9 ($C_{alkene}$), 125.8 ($C_{arom}$), 100.6 (C-1′), 82.0 (C-3), 81.5 (C-3′), 79.1 (C-2), 77.9 (C-4′/C-5′/C-4), 77.6 (C-4′/C-5′/C-4), 75.5, 75.4, 75.0 (C-Bn), 73.6 (C-4′/C-5′/C-4), 71.7 (C-Bn), 64.2 (C-6), 62.7 (C-6′), 53.6 (C-2′), 44.2 (C-5), 27.0 (TBDPS), 23.0 (Acetyl), 19.5 (TBDPS quaternary C). HRMS (ESI) m/z: $[M+Na]^+$ calculated for $C_{66}H_{71}NO_{10}SiNa$ 1088.4739, found 1088.4741.

**2,3-di-*O*-benzyl-4-*O*-(2-*N*-acetyl-2-deoxy-3,4-di-*O*-benzyl-6-*O*-tert-butyldiphenylsilyl-α-D-glucopyranosyl)-cyclophellitol alkene (14c)**

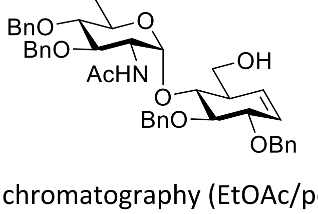

Benzoyl ester **14b** (0.52 g, 0.49 mmol) was dissolved in MeOH/DCM (13.5 ml, 4.4/1, v/v). NaOMe (5.4 M in MeOH, 0.03 ml, 0.15 mmol) was added and the reaction was stirred overnight. The reaction was quenched with $NH_4Cl$. Volatiles were removed under reduced pressure. The residue was dissolved in EtOAc and washed with $H_2O$ and brine, dried over $MgSO_4$, filtered and concentrated under reduced pressure. Column chromatography (EtOAc/pentane, 1/9 -> 2/3, v/v) yielded the product (0.46 g, 0.47 mmol, 97%).

$^1$H NMR (400 MHz, $CDCl_3$) δ 7.70 (m, 5H, $H_{arom}$), 7.47 – 7.13 (m, 30H, $H_{arom}$), 6.68 (d, *J* = 9.7 Hz, 1H, NH), 5.87 (dt, *J* = 10.3, 2.6 Hz, 1H, $H_{alkene}$), 5.63 (ddd, *J* = 10.3, 2.9, 1.6 Hz, 1H, $H_{alkene}$), 4.94 – 4.89 (m, 2H, H-1′, $CH_2$-Bn), 4.83 (d, *J* = 10.6 Hz, 1H, $CH_2$-Bn), 4.76 – 4.66 (m, 3H, $CH_2$-Bn), 4.64 (d, *J* = 2.2 Hz, 1H, $CH_2$-Bn), 4.60 (d, *J* = 6.2 Hz, 1H, $CH_2$-Bn), 4.57 – 4.50 (m, 1H, $CH_2$-Bn), 4.35 (td, *J* = 9.7, 3.4 Hz, 1H, H-2′), 4.18 (m, 1H, H-2), 3.95 (dd, *J* = 11.0, 4.1 Hz, 1H, H-6′), 3.92 – 3.82 (m, 4H, H-5′, H-6′, H-4, H-6), 3.80 –

3.74 (m, 2H, H-4, H-3), 3.74 – 3.68 (m, 1H, H-3'), 3.61 (dd, *J* = 11.0, 3.5 Hz, 1H, H-6), 2.38 (m, 1H, H-5), 1.47 (s, 3H, Acetyl), 1.05 (s, 9H, TBDPS). $^{13}$C NMR (101 MHz, CDCl$_3$) δ 170.5 (C=O), 138.7, 138.3, 138.0, 137.6, 136.1, 135.8, 133.7, 133.3, 129.9 (C$_{arom}$), 129.5 (C$_{alkene}$), 128.9, 128.8, 128.7, 128.6, 128.5, 128.4, 128.3, 128.2, 128.1, 128.0, 127.9, 127.8, 127.8, 127.7 (C$_{arom}$), 126.9 (C$_{alkene}$), 100.4 (C-1'), 81.7 (C-3), 81.3 (C-3'), 78.8 (C-2), 78.1 (C-4'/C-5'/C-4), 77.9 (C-4'/C-5'/C-4), 75.3, 75.3, 74.8 (C-Bn), 73.7 (C-4'/C-5'/C-4), 71.6 (C-Bn), 62.9 (C-6'), 62.7 (C-6), 53.4 (C-2'), 46.2 (C-5), 27.0 (TBDPS), 22.9 (Acetyl), 19.5 (TBDPS, quaternary C). HRMS (ESI) m/z: [M+Na]$^+$ calculated for $C_{59}H_{67}NO_9SiNa$ 984.4477, found 984.4506.

**2,3-di-*O*-benzyl-4-*O*-(2-*N*-acetyl-2-deoxy-3,4-di-*O*-benzyl-6-*O*-tert-butyldiphenylsilyl-α-D-glucopyranosyl)-6-*O*-tert-butyloxycarbonyl-cyclophellitol alkene (14d)**

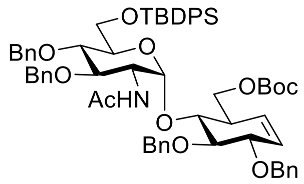


Alcohol **14c** (0.72 g, 0.75 mmol) was co-evaporated three times with toluene and dissolved in dry THF (5.4 ml, 0.14 M). $Boc_2O$ (0.19 ml, 0.81 mmol) and DMAP (9 mg, 75 µmol) were added and the reaction was stirred at room temperature. After 1 hour the reaction was cooled to 0 °C and quenched with $H_2O$. The reaction mixture was further diluted with $H_2O$ and extracted with $Et_2O$. The organic layer was washed with $NH_4Cl$ (aq. sat.), $NaHCO_3$ (aq. sat.) and brine, dried over $MgSO_4$, filtered and concentrated in vacuo. Flash column chromatography (EtOAc/PE, 1/9 -> 2/3) yielded the product (0.60 g, 0.57 mmol, 76%).

$^1$H NMR (300 MHz, CDCl$_3$) δ 7.70 (m, 5H, H$_{arom}$), 7.48 – 7.07 (m, 30H, H$_{arom}$), 6.75 (d, *J* = 9.7 Hz, 1H, NH), 5.82 (dt, *J* = 10.2, 2.5 Hz, 1H, H$_{alkene}$), 5.62 (dt, *J* = 10.3, 2.7, 1.6 Hz, 1H, H$_{alkene}$), 5.00 – 4.83 (m, 3H, CH$_2$-Bn, H-1'), 4.80 (d, *J* = 11.1 Hz, 1H, CH$_2$-Bn), 4.73 – 4.53 (m, 5H, CH$_2$-Bn), 4.44 – 4.27 (m, 3H, H-2, H-6), 4.27 – 4.19 (m, 1H, H-2), 4.03 – 3.84 (m, 4H, H-4'/5', H-4'/5', H-6'), 3.84 – 3.69 (m, 3H, H-3', H-3, H-4), 2.52 (s, 1H, H-5), 1.45 (s, 3H, Acetyl), 1.42 (s, 9H, t-Butyl), 1.06 (s, 9H, TBDPS). $^{13}$C NMR (75 MHz, CDCl$_3$) δ 170.4 (C=O, acetyl), 153.5 (C=O, Boc), 138.7, 138.4, 138.0, 137.7, 136.0, 135.7, 133.8, 133.1, 129.8, 128.7, 128.7, 128.6, 128.5, 128.5, 128.3, 128.2, 128.1, 128.1, 128.1, 127.9, 127.9, 127.8, 127.8, 127.6, 127.5 (C$_{arom}$, C$_{alkene}$), 126.9 (C$_{alkene}$), 100.6 (C-1'), 81.9 (C-3'/C-3), 81.8 (C-3'/C-3), 79.3 (C-2), 77.8 (C-4'/5'/C-4), 77.7 (C-4'/5'), 75.3, 75.2, 75.0 (C-Bn), 73.5 (C-4'/5'/4), 71.6 (C-Bn), 66.2 (C-6), 62.6 (C-6'), 53.6 (C-2'), 44.1 (C-5), 27.8 (t-butyl), 27.0 (TBDPS), 22.8 (Acetyl), 19.5 (TBDPS, quaternary C). HRMS (ESI) m/z: [M+H]$^+$ calculated for $C_{64}H_{76}NO_{11}Si$ 1062.5182, found 1062.5182.

**1-iodo-2,3-di-*O*-benzyl-4-*O*-(2-*N*-acetyl-2-deoxy-3,4-di-*O*-benzyl-6-*O*-tert-butyldiphenylsilyl-α-D-glucopyranosyl)-6,7-*O*-carbonyl-cyclophellitol alkane (14e)**

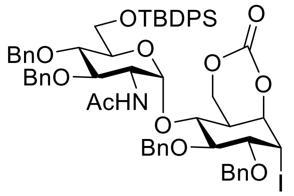


To a solution of Boc protected **14d** (0.22 g, 0.21 mmol) in DCM/AcOH (1.1 ml, 0.2 M, 2/1, v/v), NIS (0.09 g, 0.41 mmol) was added. After 22 hours the reaction mixture was diluted with $Et_2O$ and quenched with $Et_3N$. The organic layer was washed with $NH_4Cl$ (aq. sat.), $NaHCO_3$ (aq. sat.), $Na_2S_2O_3$ (aq. sat.) and brine, dried over $MgSO_4$, filtered and concentrated in vacuo. Flash column chromatography (EtOAc/PE, 1/9 -> 2/3) yielded the product (0.21 g, 0.19 mmol, 91%).

$^1$H NMR (400 MHz, CDCl$_3$) δ 7.78 – 7.59 (m, 5H, H$_{arom}$), 7.51 – 7.12 (m, 30H, H$_{arom}$), 6.43 (d, *J* = 9.7 Hz, 1H, NH), 5.08 (d, *J* = 10.5 Hz, 1H, CH$_2$-Bn), 5.03 (d, *J* = 12.2 Hz, 1H, H-6), 4.92 – 4.82 (m, 4H, H-1', H-7, CH$_2$-Bn), 4.70 – 4.63 (m, 4H, H-1, CH$_2$-Bn), 4.60 (d, *J* = 10.5 Hz, 1H, CH$_2$-Bn), 4.54 (d, *J* = 11.0 Hz, 1H, CH$_2$-Bn), 4.41 (td, *J* = 10.0, 3.3 Hz, 1H, H-2'), 4.06 (t, *J* = 6.7 Hz, 1H, H-6), 4.00 – 3.82 (m, 5H, H-4', H-6', H-5', H-3), 3.82 – 3.72 (m, 2H, H-3', H-4), 3.13 (dd, *J* = 9.2, 4.1 Hz, 1H, H-2), 2.63 (d, *J* = 10.1 Hz, 1H, H-5), 1.25 (s, 3H, Acetyl), 1.06 (s, 9H, TBDPS). $^{13}$C NMR (101 MHz, CDCl$_3$) δ 170.5 (C=O Acetyl), 146.9 (C=O, Carbonate), 138.5, 138.1, 137.1, 136.9, 135.9, 135.63, 133.7, 132.9, 130.0, 129.9, 128.8, 128.7, 128.7, 128.6, 128.6, 128.5, 128.4, 128.3, 128.2, 128.1, 128.1, 128.0, 127.9, 127.8, 127.8, 127.7, 127.3 (C$_{arom}$), 101.5 (C-1'), 82.2 (C-3), 81.7 (C-7), 81.6 (C-3'), 77.7 (C-4'/C-5'), 77.4 (C-4), 76.8 (C-2), 76.3, 75.4, 75.2 (C-Bn), 74.1 (C-4'/C-5'), 72.3 (C-Bn), 68.5 (C-6), 62.7 (C-6'), 53.4 (C-2'), 35.2 (C-5), 30.5 (C-1), 27.0

(TBDPS), 22.6 (Acetyl). HRMS (ESI) m/z: $[M+H]^+$ calculated for $C_{60}H_{67}INO_{11}Si$ 1132.3523, found 1132.3525.

**2,3-di-*O*-benzyl-4-*O*-(2-*N*-acetyl-2-deoxy-3,4-di-*O*-benzyl-6-*O*-tert-butyldiphenylsilyl-α-D-glucopyranosyl)-cyclophellitol (15a)**

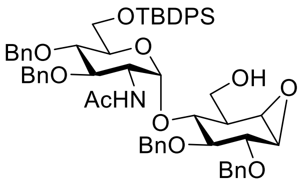


Iodide **14e** (0.12 g, 0.11 mmol) was dissolved in MeOH/DCM (1.9 ml, 0.06 M, 1.6/1, v/v), NaOMe (4.37 M in MeOH, 0.09 ml, 0.39 mmol) was added and the reaction was stirred for 20 hours. $Et_3N{\cdot}HCl$ was added and the solution was concentrated in vacuo. EtOAc was added and the solution was washed with $H_2O$ and brine, dried over $MgSO_4$, filtered and concentrated in vacuo. The product was used and analyzed without further purification. (0.12 g, quantitative)

$^1H$ NMR (400 MHz, $CDCl_3$) δ 7.71 (m, 5H, $H_{arom}$), 7.48 – 7.14 (m, 30H, $H_{arom}$), 6.82 (d, *J* = 9.7 Hz, 1H, NH), 4.95 (d, *J* = 11.0 Hz, 1H, $CH_2$-Bn), 4.88 (d, *J* = 3.5 Hz, 1H, H-1’), 4.81 (m, 2H, $CH_2$-Bn), 4.72 – 4.64 (m, 4H, $CH_2$-Bn), 4.47 (d, *J* = 11.2 Hz, 1H, $CH_2$-Bn), 4.34 (td, *J* = 9.9, 3.4 Hz, 1H, H-2’), 4.10 – 4.04 (m, 1H, H-6), 3.97 (m, 2H, H-6’, H-6), 3.92 – 3.82 (m, 3H, H-5’, H-6’, H-2), 3.80 (d, *J* = 9.2 Hz, 1H, H-4’), 3.71 – 3.61 (m, 2H, H-3’, H-4), 3.55 (dd, *J* = 10.1, 7.7 Hz, 1H, H-3), 3.34 – 3.32 (m, 1H, $H_{epoxide}$), 3.15 (d, *J* = 3.7 Hz, 1H, $H_{epoxide}$), 2.03 – 1.96 (m, 1H, H-5), 1.79 (s, 1H, OH), 1.44 (s, 3H, Acetyl), 1.05 (s, 9H, TBDPS). $^{13}C$ NMR (101 MHz, $CDCl_3$) δ 170.4 (C=O), 138.7, 138.3, 137.6, 137.3, 136.2, 136.1, 135.8, 133.8, 133.2, 129.9, 128.9, 128.8, 128.8, 128.7, 128.6, 128.6, 128.4, 128.4, 128.3, 128.3, 128.3, 128.2, 128.1, 128.0, 127.9, 127.8, 127.8, 127.7 ($C_{arom}$), 100.9 (C-1’), 83.3 (C-3), 81.6 (C-3’), 80.2 (C-5’/C-2), 77.7 (C-4’), 76.1 (C-4), 75.68, 75.2, 75.0 (C-Bn), 73.9 (C-5’/C-2), 72.9 (C-Bn), 62.8 (C-6’), 61.9 (C-6), 56.7 ($C_{epoxide}$), 53.4 (C-2’), 52.4 ($C_{epoxide}$), 44.7 (C-5), 29.9, 27.7, 27.0 (TBDPS), 22.9 (Acetyl), 19.5 (TBDPS, quaternary C). HRMS (ESI) m/z: $[M+Na]^+$ calculated for $C_{59}H_{67}NO_{10}SiNa$ 1000.4426, found 1000.4452.

**2,3-di-*O*-benzyl-4-*O*-(2-*N*-acetyl-2-deoxy-3,4-di-*O*-benzyl-6-*O*-tert-butyldiphenylsilyl-α-D-glucopyranosyl)-glucurono-cyclophellitol (15b)**

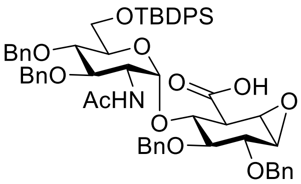


Alcohol **15a** (0.13 g, 0.13 mmol) was dissolved in *t*-BuOH/DCM/$H_2O$ (4.9 ml, 5/4/1, v/v). and cooled down to 0 °C. TEMPO (4 mg, 0.03 mmol) and BAIB (0.10 g, 0.32 mmol) were added and the reaction was stirred 23 hours. The reaction was diluted with DCM and quenched with $Na_2S_2O_3$ (aq. sat.). The layers were separated and the water layer was acidified with AcOH. The water layer was extracted four times with DCM. The combined organic layers were dried over $MgSO_4$, filtered and concentrated under reduced pressure. Column chromatography (EtOAc/pentane 1% AcOH, 1/4 -> 1/1, v/v) afforded the product (0.10 mg, 0.10 mmol, 80%).

$^1H$ NMR (400 MHz, $CDCl_3$) δ 7.71 (m, *J* = 27.7, 6.6, 1.7 Hz, 5H, $H_{arom}$), 7.47 – 7.09 (m, 30H, $H_{arom}$), 6.07 (d, *J* = 9.6 Hz, 1H, NH), 4.96 – 4.73 (m, 6H, H-1’, $CH_2$-Bn), 4.66 – 4.53 (m, 3H, $CH_2$-Bn), 4.27 (td, *J* = 10.1, 3.6 Hz, 1H, H-2’), 4.05 (dd, *J* = 11.5, 2.5 Hz, 1H, H-6’), 4.01 – 3.93 (m, 2H, H-4’, H-4), 3.92 – 3.86 (m, 1H, H-2), 3.83 – 3.74 (m, 2H, H-5’, H-6’), 3.69 (t, *J* = 10.4, 9.0 Hz, 1H, H-3’), 3.46 (dd, *J* = 10.2, 8.2 Hz, 1H, H-3), 3.30 (t, *J* = 2.9 Hz, 1H, $H_{epoxide}$), 3.16 (d, *J* = 3.6 Hz, 1H, $H_{epoxide}$), 2.79 (dd, *J* = 8.9, 2.2 Hz, 1H, H-5), (s, 3H, Acetyl), 1.05 (s, 9H, TBDPS). $^{13}C$ NMR (101 MHz, $CDCl_3$) δ 170.8 (C=O), 138.7, 138.5, 138.1, 137.7, 137.2, 136.5, 135.9, 133.8, 133.6, 130.0, 129.9, 129.2, 128.9, 128.9, 128.8, 128.7, 128.6, 128.5, 128.4, 128.3, 128.2, 128.0, 127.9, 127.80, 125.51 ($C_{arom}$), 100.4 (C-1’), 81.8 (C-3’), 81.6 (C-3), 79.1 (H-2), 77.7 (C-4’/C-4), 75.7 (C-4’/C-4), 75.6, 75.5, 75.4 (C-Bn), 73.3 (C-5’), 73.0 (C-Bn), 62.4 (C-6’), 54.2 ($C_{epoxide}$), 53.8 ($C_{epoxide}$), 53.5 (C-2’), 48.8 (C-5), 27.1 (TBDPS), 19.5 (TBDPS quaternary C), 14.4 (Acetyl). HRMS (ESI) m/z: $[M+Na]^+$ calculated for $C_{59}H_{65}NO_{11}SiNa$ 1014.4219, found 1014.4223.

## 2,3-di-*O*-benzyl-4-*O*-(2-*N*-acetyl-2-deoxy-3,4-di-*O*-benzyl-α-D-glucopyranosyl)-glucurono-cyclophellitol (16a)

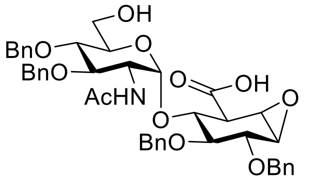

Silyl protected **15b** (89 mg, 0.090 mmol) was dissolved in anhydrous THF (1.9 ml). $Et_3N{\cdot}3HF$ (0.046 ml, 0.28 mmol) was added and the reaction was stirred for 20 hours. More $Et_3N{\cdot}3HF$ (0.030 ml, 0.18 mmol) was added and the reaction was stirred 23 hours. The reaction was diluted with DCM and washed with water. The water layer was extracted four times with DCM. The combined organic layers were dried over $MgSO_4$, filtered and concentrated under reduced pressure. Column chromatography (EtOAc/pentane 1% AcOH, 1/9 -> 1/4, v/v) yielded the product (62 mg, 0.082 mmol, 87%).

$^1$H NMR (400 MHz, $CDCl_3$) δ 7.42 – 7.06 (m, 20H, $H_{arom}$), 6.37 (d, *J* = 9.6 Hz, 1H, NH), 4.96 (d, *J* = 10.9 Hz, 1H, $CH_2$-Bn), 4.85 (d, *J* = 3.8 Hz, 1H, H-1'), 4.80 (d, *J* = 11.4 Hz, 3H, $CH_2$-Bn), 4.69 (dd, *J* = 12.3, 10.9 Hz, 2H, $CH_2$-Bn), 4.59 (d, *J* = 11.4 Hz, 1H, $CH_2$-Bn), 4.53 (d, *J* = 10.9 Hz, 1H, $CH_2$-Bn), 4.33 (td, *J* = 10.0, 3.8 Hz, 1H, H-2'), 4.02 – 3.94 (m, 2H, H-6', H-4), 3.92 (d, *J* = 8.4 Hz, 1H, H-2), 3.86 – 3.76 (m, 3H, H-4', H-5', H-6'), 3.68 – 3.62 (m, 1H, H-3'), 3.49 (dd, *J* = 10.5, 8.4 Hz, 1H, H-3), 3.40 (t, *J* = 2.9 Hz, 1H, $H_{epoxide}$), 3.18 (d, *J* = 3.6 Hz, 1H, $H_{epoxide}$), 2.88 (dd, *J* = 9.1, 2.1 Hz, 1H, H-5), 1.19 (s, 3H, Acetyl). $^{13}$C NMR (101 MHz, $CDCl_3$) δ 173.3 (C=O), 138.7, 138.4, 138.1, 137.8, 137.3, 129.2, 128.9, 128.9, 128.8, 128.6, 128.5, 128.5, 128.4, 128.4, 128.4, 128.4, 128.0, 127.7, 127.7, 127.7, 125.5 ($C_{arom}$), 100.6 (C-1'), 81.9 (C-3'), 81.6 (C-3), 79.4 (C-2), 77.7 (C-4'), 76.8 (C-4), 75.9, 75.3, 75.2 (C-Bn), 73.4 (C-5'), 72.9 (C-Bn), 61.1 (C-6'), 54.5 ($C_{epoxide}$), 53.8 (C-2', $C_{epoxide}$), 49.5 (C-5), 22.8 (Acetyl). HRMS (ESI) m/z: $[M+Na]^+$ calculated for $C_{43}H_{47}NO_{11}Na$ 776.3041 found 776.3058.

## 4-*O*-(2-*N*-acetyl-2-deoxy-α-D-glucopyranosyl)-glucurono-cyclophellitol (2)

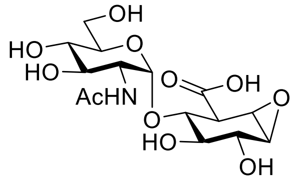

Ammonia (10 ml) was condensed at -70 °C. Sodium (17 mg, 0.72 mmol) was added and the solution turned blue. Benzyl protected **16a** (14 mg, 0.018 mmol), dissolved in anhydrous THF (2ml) and *t*-BuOH (0.068 ml, 0.72 mmol) was added. After stirring at -60°C for 15 minutes the blue color faded and more sodium (12 mg, 0.51 mmol) was added. After another 30 minutes the blue color faded again the reaction was quenched with $NH_4Cl$ (66 mg, 1.23 mmol) The ammonia was evaporated, water was added and the compound was desalted by size exclusion over HW-40 (1% AcOH in water). Lyophilization afforded the compound as a white solid. (6.42 mg, 0.016 mmol, 91%).

$^1$H NMR (400 MHz, $D_2O$) δ 5.16 (d, *J* = 3.8 Hz, 1H, H-1'), 3.84 – 3.79 (m, 1H, H-2'), 3.79 – 3.73 (m, 4H, H-3', H-4, H-6'), 3.72 – 3.66 (m, 2H, H-4', H-2), 3.50 – 3.42 (m, 3H, H-5', H-3, $H_{epoxide}$), 3.15 (d, *J* = 3.8 Hz, 1H, $H_{epoxide}$), 2.81 (dd, *J* = 9.6, 1.9 Hz, 1H, H-5), 1.99 (s, 3H, Acetyl). $^{13}$C NMR (101 MHz, $D_2O$) δ 181.5 (C=O/COOH), 174.3 (C=O/COOH), 97.5 (C-1'), 76.2 (C-3), 74.9 (C-3'), 71.8 (C-4'/C-2), 71.4 (C-4), 70.9 (C-4'/C-2), 69.6 (C-5'), 60.0 (C-6'), 56.2 ($C_{epoxide}$), 55.4 ($C_{epoxide}$), 53.9 (C-2'), 51.0 (C-5), 21.9 (Acetyl). HRMS (ESI) m/z: $[M+Na]^+$ calculated for $C_{15}H_{23}NO_{11}Na$ 416.1163, found 416.1164.

## 2,3-di-*O*-benzyl-4-*O*-(2-*N*-acetyl-2-deoxy-3,4-di-*O*-benzyl-6-*O*-sulfo-α-D-glucopyranosyl)-glucurono-cyclophellitol (16b)

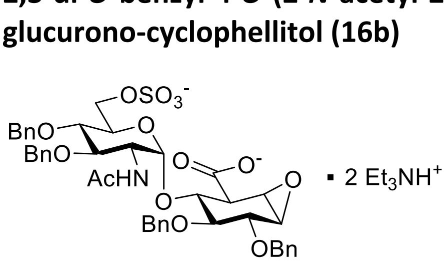

Alcohol **16a** (0.062 g, 0.082 mmol) was dissolved in dry DMF (1.1 ml, 0.08 M). $SO_3{\cdot}Et_3N$ (0.031 g, 0.17 mmol) was added and the mixture was stirred for 4 hours. $Et_3N$ (0.07 ml, 0.5 mmol) was added, the reaction mixture was diluted with MeOH (2 ml) and concentrated under reduced pressure. The crude mixture was analyzed and used without purification.

$^1$H NMR (400 MHz, MeOD) δ 7.37 – 7.09 (m, 20H, $H_{arom}$), 5.35 (d, *J* = 3.9 Hz, 1H, H-1'), 4.82 – 4.66 (m, 5H, $CH_2$-Bn), 4.64 – 4.52 (m, 3H, $CH_2$-Bn), 4.34 (d, *J* = 10.6 Hz, 1H, H-6'), 4.17 (d, *J* = 10.6 Hz, 1H, H-6'), 4.10 (dd, *J* = 9.9, 4.0 Hz, 1H, H-2'), 4.01 (t, *J* = 9.7 Hz, 1H, H-4), 3.83 – 3.75 (m, 2H, H-4', H-5'), 3.70 – 3.66 (m, 1H, H-3), 3.64 (d, *J* = 1.3 Hz, 1H, H-3'), 3.61 (d, *J* = 9.6 Hz, 1H, H-2), 3.44 – 3.40 (m, 1H, $H_{epoxide}$,

3.29 – 3.23 (m, 2H, $H_{epoxide}$, H-5), 3.16-3.10 (m, 12H, $Et_3NH^+$) 1.67 (d, *J* = 1.4 Hz, 3H), 1.30-1.22 (m, 18H, $Et_3NH^+$). $^{13}$C NMR (101 MHz, MeOD) δ 174.2 (C=O/COOH), 172.9 (C=O/COOH), 140.1, 139.8, 139.6, 139.0, 129.5, 129.5, 129.3, 129.2, 129.0, 128.7, 128.6, 128.5, 128.5 ($C_{arom}$), 99.3 (C-1'), 84.8 (C-3), 82.0 (C-3'), 81.4 (C-4'/C-5'), 79.0 (C-2), 76.2, 75.9, 75.2, 73.3 (C-Bn), 72.8 (C-4) , 71.5 (C-4'/C-5'), 66.8 (C-6'), 55.6 ($C_{epoxide}$), 54.2 (C-2'), 53.8 ($C_{epoxide}$), 49.4 (C-5), 47.9 ($Et_3NH^+$, $CH_2$), 22.9 (Acetyl), 9.3 ($Et_3N$, $CH_3$). HRMS (ESI) m/z: $[M+H]^+$ calculated for $C_{43}H_{47}NO_{14}S$ 834.2790, found 834.2785.

### 4-*O*-(2-*N*-acetyl-2-deoxy-6-*O*-sulfo-α-D-glucopyranosyl)-glucurono-cyclophellitol (3)

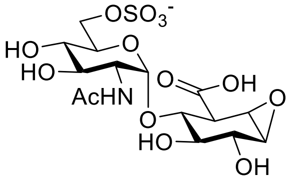


Ammonia was condensed at -60°C under an inert atmosphere and Na (37 mg, 1.6 mmol) was added. The solution turned dark blue. Benzyl protected **16b** (35 μmol, 33 mg) was dissolved in THF/*t*-BuOH (0.6 ml, 2/3, v/v) and added to the solution. After stirring for 45 minutes the reaction was quenched with $NH_4Cl$ (0.10 g, 1.92 mmol). Ammonia was evaporated at room temperature, diluted in water and concentrated under reduced pressure. The compound was desalted by size exclusion over HW-40 (1% AcOH in water). Lyophilization afforded the compound as a white solid. (14.9 mg, 0.03 mmol, 87% over 2 steps).

$^1$H NMR (400 MHz, $D_2O$) δ 5.22 (d, *J* = 3.8 Hz, 1H, H-1'), 4.24 (ddd, *J* = 72.7, 11.1, 2.1 Hz, 2H, H-6'), 3.84 (m, *J* = 10.6, 3.6 Hz, 2H, H-2', H-5'), 3.82 – 3.76 (m, 2H, H-4, H-2/H-3), 3.70 (t, *J* = 10.7, 9.1 Hz, 1H, H-3'/H-4'), 3.56 (t, *J* = 10.1, 9.1 Hz, 1H, H-3'/H-4'), 3.50 – 3.44 (m, 2H, $H_{epoxide}$, H-2/H-3), 3.15 (d, *J* = 3.8 Hz, 1H, $H_{epoxide}$), 2.83 (dd, *J* = 9.7, 1.9 Hz, 1H, H-5), 1.99 (s, 3H, Acetyl). $^{13}$C NMR (101 MHz, $D_2O$) δ 180.6 (C=O/COOH/COOH), 177.7 (C=O/COOH/COOH), 174.3 (C=O/COOH/COOH), 97.4 (C-1'), 76.5 (C-3), 74.4 (C-2/C-4), 71.6 (C-2/C-4), 70.8 (C-3'), 69.9 (C-5'), 69.0 (C-4'), 66.3 (C-6'), 56.1 ($C_{epoxide}$), 55.3 ($C_{epoxide}$), 53.7 (C-2'), 50.9 (C-5), 22.0 (Acetyl). HRMS (ESI) m/z: $[M+2H]^+$ calculated for $C_{15}H_{24}NO_{14}S$ 474.09120 found 474.09129.

### 2,3-di-*O*-benzyl-4-*O*-(2-azido-2-deoxy-3,4-di-*O*-benzyl-6-*O*-tert-butyldiphenylsilyl-α-D-glucopyranosyl)-cyclophellitol alkene (17)

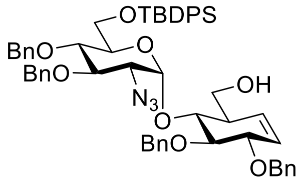


Ester **14a** (0.98 g, 0.93 mmol, 1 eq) was dissolved in MeOH/DCM (1/1, v/v, 18 ml). NaOMe (25 wt%, 0.1 ml, 0.44 mmol) was added and the reaction mixture was stirred overnight. $NH_4Cl$ was added and volatiles were removed under reduced pressure. The crude product was purified using column chromatography (EtOAc/pentane, 1/19 -> 3/17, v/v) this yielded the product as a colourless oil. (0.91 g, quant.)

$^1$H NMR (400 MHz, $CDCl_3$) δ 7.72 – 7.64 (m, 5H, $H_{arom}$), 7.43 – 7.23 (m, 28H, $H_{arom}$), 7.18 – 7.12 (m, 2H, $H_{arom}$), 5.80 (dt, J = 10.2, 2.4 Hz, 1H, $H_{alkene}$), 5.65 (d, J = 4.0 Hz, 1H, H-1'), 5.59 (dt, J = 10.2, 2.1 Hz, 1H, $H_{alkene}$), 5.06 (d, J = 10.9 Hz, 1H, $CH_2$-Bn), 4.95 (d, J = 10.9 Hz, 1H, $CH_2$-Bn), 4.87 (s, 2H, $CH_2$-Bn), 4.83 (d, J = 10.7 Hz, 1H, $CH_2$-Bn), 4.70 – 4.58 (m, 3H, $CH_2$-Bn), 4.25 – 4.20 (m, 1H, H-2), 4.01 – 3.89 (m, 3H, H-3', H-4', H-4), 3.88 – 3.81 (m, 3H, H-5', H-6'), 3.75 – 3.58 (m, 3H, H-3, H-6), 3.29 (dd, J = 10.3, 4.0 Hz, 1H, H-2'), 2.52 – 2.46 (m, 1H, H-5), 1.69 (s, 1H, OH), 1.04 (s, 9H, TBDPS). $^{13}$C NMR (101 MHz, $CDCl_3$) δ 139.04, 138.34, 137.95, 137.88, 135.99, 135.77, 133.41, 133.05, 129.85, 128.87, 128.64, 128.62, 128.57, 128.54, 128.50, 128.27, 128.08, 128.01, 127.87, 127.83, 127.77, 127.65, 127.54 ($C_{arom}$, $C_{alkene}$), 98.22 (C-1'), 84.17 (C-3), 80.88 (C-2), 80.34 (C-4'), 78.42 (C-3'), 75.66, 75.30, 74.63 (C-Bn), 74.35 (C-4), 72.87 (C-5'), 71.74 (C-Bn), 63.55 (C-2'), 63.02 (C-6'), 62.55 (C-6), 45.46 (C-5), 26.97 (TBDPS), 19.38 (TBDPS quaternary C). HRMS (ESI) m/z: $[M+Na]^+$ calculated for $C_{57}H_{63}N_3O_8SiNa$ 968.4277, found 968.4274.

**2,3-di-*O*-benzyl-4-*O*-(2-azido-2-deoxy-3,4-di-*O*-benzyl-6-*O*-tert-butyldiphenylsilyl-α-D-glucopyranosyl)-6-*O*-tert-butyloxycarbonyl-cyclophellitol alkene (17a)**

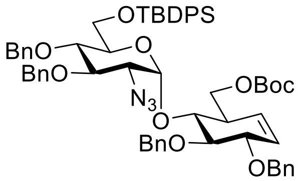


Alcohol **17** (0.92 g, 0.93 mmol) was co-evaporated with toluene and subsequently dissolved in THF (9.3 ml, 0.1 M). DMAP (91 mg, 0.74 mmol) and $Boc_2O$ (0.41 g, 1.87 mmol) were added. The reaction was stirred at room temperature for 3 hours. Water was added and the mixture was stirred for 15 minutes. The mixture was extracted three times with $Et_2O$, the combined organic layers were washed with $NH_4Cl$ (aq. sat.), $NaHCO_3$ (aq. sat.) and brine, dried over $MgSO_4$, filtered and concentrated under reduced pressure. The product was obtained after column chromatography ($Et_2O$/pentane, 0/1 -> 1/9, v/v) as a colorless oil. (0.80 g, 82%)

$^1$H NMR (500 MHz, $CDCl_3$) δ 7.73 – 7.59 (m, 4H, $H_{arom}$), 7.44 – 7.12 (m, 26H, $H_{arom}$), 5.76 (dt, *J* = 10.2, 2.4 Hz, 1H, $H_{alkene}$), 5.66 (d, *J* = 3.9 Hz, 1H, H-1'), 5.57 (dt, *J* = 10.2, 2.3 Hz, 1H, $H_{alkene}$), 5.05 (d, *J* = 10.9 Hz, 1H, $CH_2$-Bn), 4.94 (d, *J* = 10.9 Hz, 1H, $CH_2$-Bn), 4.91 – 4.83 (m, 3H, $CH_2$-Bn), 4.74 (d, *J* = 10.9 Hz, 1H, $CH_2$-Bn), 4.70 – 4.60 (m, 2H, $CH_2$-Bn), 4.26 – 4.16 (m, 2H, H-6, H-2), 4.03 – 3.94 (m, 3H, H-3', H-6, H-6'), 3.93 – 3.81 (m, 4H, H-4, H-3, H-6', H-4'), 3.75 – 3.69 (m, 1H, H-5'), 3.28 (dd, *J* = 10.4, 3.9 Hz, 1H, H-2'), 2.69 – 2.61 (m, 1H, H-5), 1.25 (s, 9H, t-Butyl), 1.03 (s, 9H, TBDPS). $^{13}$C NMR (126 MHz, $CDCl_3$) δ 153.5 (Boc), 139.0, 138.3, 138.1, 136.0, 135.7, 133.8, 133.2, 129.7, 129.7, 128.6, 128.6, 128.5, 128.5, 128.4, 128.0, 128.0, 127.9, 127.8, 127.7, 127.6, 127.5, 127.5 ($C_{arom}$, $C_{alkene}$), 98.0 (C-1'), 84.3 (C-3'/C-4'/C-4), 82.1 (Boc), 80.7 (C-2), 80.3 (C-3), 78.2 (C-3'/C-4'/C-4), 75.7, 75.1, 74.6 (C-Bn), 74.1 (C-3'/C-4'/C-4), 72.7 (C-5'), 71.8 (C-Bn), 66.6 (C-6), 63.7 (C-2'), 62.3 (C-6'), 43.0 (C-5), 27.7, 27.0 (TBDPS, t-Butyl), 19.4 (TBDPS). HRMS (ESI) m/z: $[M+Na]^+$ calculated for $C_{62}H_{71}N_3O_{10}SiNa$ 1068.48009, found 1068.48007.

**1-iodo-2,3-di-*O*-benzyl-4-*O*-(2-azido-2-deoxy-3,4-di-*O*-benzyl-6-*O*-tert-butyldiphenylsilyl-α-D-glucopyranosyl)-6,7-*O*-carbonyl-cyclophellitol alkane (17b)**

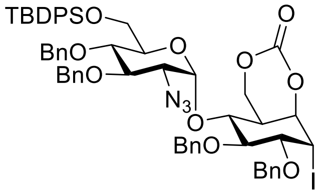


Alkene **17a** (0.80 g, 0.77 mmol) was dissolved in AcOH/DCM (1/2, v/v, 5.1 ml, 0.15 M). NIS (0.35 g , 1.54 mmol) was added and the reaction was stirred for 18 hours in the dark. The mixture was diluted with $Et_2O$, washed with $Na_2S_2O_3$ (aq.), $NaHCO_3$ (aq. sat.) and brine, dried over $MgSO_4$, filtered and concentrated under reduced pressure. The product was obtained after column chromatography ($Et_2O$/pentane, 1/4 -> 1/1, v/v) as a foam. (0,61 g, 71%)

$^1$H NMR (500 MHz, $CDCl_3$) δ = 7.64 (ddt, *J*=6.6, 2.8, 1.4, 4H, $H_{arom}$), 7.45 – 7.22 (m, 24H, $H_{arom}$), 7.21 – 7.11 (m, 2H, $H_{arom}$), 5.50 (d, *J*=3.9, 1H, H1'), 5.03 (d, *J*=10.7, 1H, $CH_2$-Bn), 4.93 – 4.83 (m, 5H, H-7, $CH_2$-Bn), 4.72 – 4.63 (m, 2H, $CH_2$-Bn), 4.63 – 4.54 (m, 3H, H-1, H-6, $CH_2$-Bn), 4.27 (dd, *J*=11.6, 3.3, 1H, H-6), 4.00 – 3.93 (m, 2H, H-6', H3), 3.94 – 3.82 (m, 4H, H-3', H-6', H-4, H-4'), 3.63 (dt, *J*=9.7, 2.3, 1H, H-5'), 3.36 (dd, *J*=10.2, 3.9, 1H, H-2'), 3.13 (dd, *J*=8.8, 4.0, 1H, H-2), 2.79 – 2.73 (m, 1H, H-5), 1.06 (s, 9H, TBDPS). $^{13}$C NMR (126 MHz, $CDCl_3$) δ 146.7 (C=O, Boc), 138.5, 137.9, 137.8, 137.2, 136.0, 135.8, 133.6, 132.9, 129.9, 129.9, 128.7, 128.5, 128.3, 128.3, 128.2, 128.2, 128.1, 128.1, 127.9, 127.8, 127.7, 127.4, ($C_{arom}$) 98.6 (C-1'), 83.4 (C-3), 81.1 (C-7), 80.2 (C-3'), 78.0 (C-4/C-4'), 77.2 (C-2), 75.8, 75.4, 75.1 (C-Bn), 74.0 (C-4/C-4'), 73.4 (C5'), 72.5 (C-Bn), 68.6 (C-6), 63.6 (C-2'), 62.0 (C-6'), 34.0 (C-5), 29.9 (C-1), 27.1 (TBDPS), 19.5 (TBDPS). HRMS (ESI) m/z: $[M+NH_4]^+$ calculated for $C_{58}H_{66}IN_4O_{10}Si$ 1133.3587, found 1133.3593.

**2,3-di-*O*-benzyl-4-*O*-(2-azido-2-deoxy-3,4-di-*O*-benzyl-6-*O*-tert-butyldiphenylsilyl-α-D-glucopyranosyl)-cyclophellitol (18)**

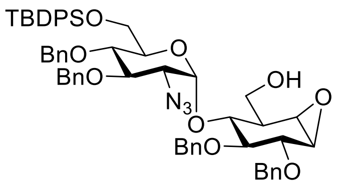

Iodide **17b** (0.606 g 0.543 mmol) was dissolved in DCM/MeOH (5.4 ml, 1/1, v/v). NaOMe (4.4M, 0.04 ml, 1.63 mmol) was added and the reaction was left to stir overnight. More NaOMe (4.4M, 0.04 ml, 1.63 mmol) was added and the reaction was stirred for 7 hours. Upon completion the reaction was quenched with $NH_4Cl$ (35 mg, 0.65 mmol). The solvent was removed under reduced pressure and column chromatography (EtOAc/pentane, 3/17 -> 7/13, v/v) afforded the product as an oil (0.492 g, 0.511 mmol, 94%).

$^1$H NMR (400 MHz, $CDCl_3$) δ 7.71 – 7.62 (m, 4H, $H_{arom}$), 7.44 – 7.24 (m, 24H, $H_{arom}$), 7.18 – 7.12 (m, 2H, $H_{arom}$), 5.61 (d, *J* = 4.0 Hz, 1H, H-1'), 4.99 (d, *J* = 10.9 Hz, 1H, $CH_2$-Bn), 4.91 – 4.76 (m, 5H, $CH_2$-Bn), 4.69 – 4.61 (m, 2H, $CH_2$-Bn), 3.97 – 3.78 (m, 6H, H-3', H-4, H-6, H-6'), 3.78 – 3.65 (m, 4H, H-2, H-3, H-4', H-5'), 3.41 – 3.36 (m, 1H, $H_{epoxide}$), 3.24 (dd, *J* = 10.3, 4.0 Hz, 1H, H-2'), 3.17 (d, *J* = 3.8 Hz, 1H, $H_{epoxide}$), 2.23 – 2.16 (m, 1H, H-5), 1.05 (s, 9H, TBDPS). $^{13}$C NMR (101 MHz, $CDCl_3$) δ 138.9, 138.0, 137.5, 136.0, 135.8, 133.6, 133.2, 129.8, 128.7, 128.6, 128.6, 128.5, 128.3, 128.2, 128.1, 128.0, 128.0, 127.8, 127.7, 127.6, 127.5 ($C_{arom}$), 97.9 (C-1'), 84.8, 80.5 (C-2/C3/C4/C4'/C5'), 80.3 (C-3'), 78.3(C-2/C3/C4/C4'/C5'), 75.7, 75.2, 74.6, 72.9 (C-Bn), 70.4 (C-2/C3/C4/C4'/C5'), 63.4 (C-2'), 62.7 (C-6/C-6'), 62.1 (C-6/C-6'), 56.0 ($C_{epoxide}$), 52.8 ($C_{epoxide}$), 43.2 (C-5), 27.0 (TBDPS), 19.4 (TBDPS). HRMS (ESI) m/z: $[M+NH_4]^+$ calculated for $C_{57}H_{67}N_4O_9Si$ 979.4672, found 979.4669.

**2,3-di-*O*-benzyl-4-*O*-(2-azido-2-deoxy-3,4-di-*O*-benzyl-6-*O*-tert-butyldiphenylsilyl-α-D-glucopyranosyl)-glucurono-cyclophellitol (19)**

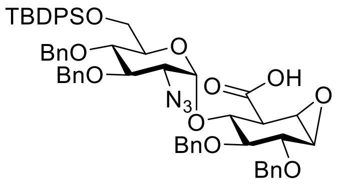

Alcohol **18** (0.492 g, 0.511 mmol) was dissolved in *t*-BuOH/ DCM/$H_2O$ (3.5 ml, 6/4/1, v/v). TEMPO (0.016 g, 0.10 mmol) and BAIB (0.412 g, 1.28 mmol) were added and the reaction was stirred overnight. After TLC analysis (EtOAc/pentane, 1/3 + 0.5% $Et_3N$) showed full conversion of the starting material the reaction was diluted with DCM and water. The organic layer was washed with $Na_2S_2O_3$ and the layers were separated. The water layer was extracted four times with DCM. The combined organic layers were dried over $MgSO_4$, filtered and concentrated under reduced pressure. The product was obtained after column chromatography (EtOAc/pentane, 3/17 -> 1/4 + 0.5% AcOH, v/v) as an oil. (0.538 g, 0.525 mmol, quant.)

$^1$H NMR (400 MHz, $CDCl_3$) δ 7.72 – 7.63 (m, 4H, $H_{arom}$), 7.43 – 7.25 (m, 24H, $H_{arom}$), 7.21 – 7.16 (m, 2H, $H_{arom}$), 5.38 (d, *J* = 3.7 Hz, 1H, H-1'), 4.94 – 4.80 (m, 5H, $CH_2$-Bn), 4.76 – 4.62 (m, 3H, $CH_2$-Bn), 4.04 (t, *J* = 9.6 Hz, 1H, H-4), 4.00 – 3.89 (m, 2H, H-3', H-6'), 3.86 – 3.79 (m, 3H, H-2, H-6', H-4'), 3.64 (m, 1H, H-5'), 3.54 (dd, *J* = 9.6, 8.2 Hz, 1H, H-3), 3.29 (dd, *J* = 10.2, 3.8 Hz, 1H, H-2'), 3.21 – 3.18 (m, 1H, $H_{epoxide}$), 3.09 (d, *J* = 3.6 Hz, 1H, $H_{epoxide}$), 2.89 (dd, *J* = 9.4, 1.5 Hz, 1H, H-5), 1.04 (s, 9H, TBDPS). $^{13}$C NMR (101 MHz, $CDCl_3$) δ 172.2 (COOH), 138.7, 138.2, 137.9, 137.4, 136.2, 135.8, 133.6, 133.3, 129.9, 129.8, 128.7, 128.6, 128.6, 128.5, 128.4, 128.2, 128.1, 128.1, 127.9, 127.9, 127.9, 127.8, 127.7 ($C_{arom}$), 98.4 (C-1'), 83.0 (C-3), 80.3 (C-3'), 79.4, 78.1 (C-2/C-4'), 75.8, 75.2, 75.0 (C-Bn), 73.6 (C-4), 73.2 (C-Bn), 72.5 (C-5'), 63.8 (C-2'), 62.3 (C-6'), 53.9 ($C_{epoxide}$), 53.5 ($C_{epoxide}$), 48.1 (C-5), 27.0 (TBDPS), 19.4 (TBDPS). HRMS (ESI) m/z: $[M+NH_4]^+$ calculated for $C_{57}H_{65}N_4O_{10}Si$ 993.44645 found 993.44650.

**2,3-di-*O*-benzyl-4-*O*-(2-azido-2-deoxy-3,4-di-*O*-benzyl-α-D-glucopyranosyl)-glucurono-cyclophellitol (20)**

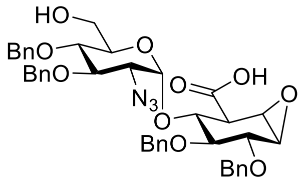

Silyl protected **19** (0.525 g, 0.538 mmol) was dissolved in THF (2.7 ml), $3HF\cdot Et_3N$ (0.44 ml, 2.69 mmol (8.07 mmol HF)) was added and the mixture was stirred for 45 hours. The reaction was poured over water and extracted four times with DCM. The combined organic layers were dried over $MgSO_4$ and concentrated under reduced pressure. Column

chromatography (EtOAc/pentane 0.5% AcOH, 7/20 -> 9/20, v/v) afforded the product as a white solid. (0.340 g, 0.461 mmol, 86%)

$^{1}$H NMR (500 MHz, $CDCl_3$) δ 7.36 – 7.23 (m, 20H, $H_{arom}$), 5.41 (d, *J* = 4.0 Hz, 1H, H-1'), 4.90 (s, 2H, $CH_2$-Bn), 4.87 – 4.79 (m, 3H, $CH_2$-Bn), 4.74 (d, *J* = 11.3 Hz, 1H, $CH_2$-Bn), 4.63 (dd, *J* = 14.7, 11.2 Hz, 2H, $CH_2$-Bn), 4.12 (t, *J* = 9.7 Hz, 1H, H-4), 3.93 – 3.85 (m, 2H, H-3', H-2), 3.81 (dd, *J* = 11.8, 2.5 Hz, 1H, H-6'), 3.72 (ddd, *J* = 10.1, 4.8, 2.5 Hz, 1H, H-5'), 3.64 – 3.58 (m, 2H, H-6', H-3), 3.46 – 3.41 (m, 2H, H-4', $H_{epoxide}$), 3.26 (dd, *J* = 10.3, 3.9 Hz, 1H, H-2'), 3.20 (d, *J* = 3.7 Hz, 1H, $H_{epoxide}$), 3.04 (dd, *J* = 9.5, 2.1 Hz, 1H, H-5). $^{13}$C NMR (126 MHz, $CDCl_3$) δ 173.8 (C-6), 138.8, 137.8, 137.7, 137.4, 128.7, 128.7, 128.6, 128.4, 128.2, 128.2, 128.1, 128.1, 127.5, 127.4 ($C_{arom}$), 98.7 (C-1'), 83.0 (C-3), 80.3 (C-3'), 79.6 (C-2), 78.4 (C-4'), 75.6, 75.2, 74.9 (C-Bn), 74.3 (C-4), 73.2 (C-Bn), 72.1 (C-5), 63.6 (C-2'), 61.9 (C-6'), 54.3 ($C_{epoxide}$), 53.7 ($C_{epoxide}$), 48.4 (C-5). HRMS (ESI) m/z: $[M+NH_4]^+$ calculated for $C_{41}H_{47}N_4O_{10}$ 755.3287 found 755.3284.

**4-*O*-(2-deoxy-α-D-glucopyranosyl)-glucurono-cyclophellitol (4)**

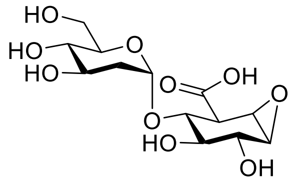


Ammonia (8 ml) was condensed at -70 °C. Sodium (31 mg, 1.35 mmol) was added and the solution turned blue. Benzyl protected **20** (50 mg, 0.068 mmol), dissolved in anhydrous THF (2ml) and *t*-BuOH (0.16 ml, 1.69 mmol) was added. After stirring at -60 °C for 45 minutes the reaction was quenched with $NH_4Cl$ (109 mg, 2.04 mmol) The ammonia was evaporated, water was added and the compound was desalted by size exclusion over HW-40 (1% AcOH in water). Lyophilization afforded the compound as a white solid. (22.7 mg, 0.068 mmol, quant.)

$^{1}$H NMR (500 MHz, $D_2O$) δ 5.31 (d, *J* = 3.7 Hz, 1H, H-1'), 3.88 (ddd, *J* = 11.9, 9.1, 4.9 Hz, 1H, H-3'), 3.84 – 3.76 (m, 4H, H-6', H-4, H-2), 3.67 (dt, *J* = 10.0, 3.1 Hz, 1H, H-5'), 3.48 – 3.45 (m, 1H, $H_{epoxide}$), 3.43 (dd, *J* = 10.2, 8.7 Hz, 1H, H-3), 3.35 (dd, *J* = 10.0, 9.2 Hz, 1H, H-4'), 3.18 (d, *J* = 3.7 Hz, 1H, $H_{epoxide}$), 2.80 (dd, *J* = 9.8, 1.9 Hz, 1H, H-5), 2.19 (ddd, *J* = 13.3, 5.0, 1.3 Hz, 1H, H-2'), 1.64 (ddd, *J* = 13.2, 11.9, 3.9 Hz, 1H, H-2'). $^{13}$C NMR (126 MHz, $D_2O$) δ 179.8 (COOH), 97.7 (C-1'), 75.7 (C-3), 73.7 (C-2/C-4), 71.6 (C-5'), 70.4 (C-2/C-4), 70.3 (C-4'), 67.6 (C-3'), 59.7 (C-6'), 55.6 ($C_{epoxide}$), 54.7 ($C_{epoxide}$), 50.4 (C-5), 36.4 (C-2'). HRMS (ESI) m/z: $[M+H]^+$ calculated for $C_{13}H_{24}O_{10}$ 354.13947 found 354.13937.

**2,3-di-*O*-benzyl-4-*O*-(2-amino-2-deoxy-3,4-di-*O*-benzyl-α-D-glucopyranosyl)-glucurono-cyclophellitol (21)**

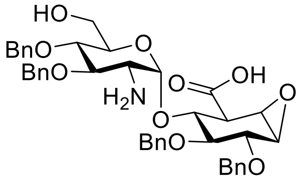


Azide **20** (50 mg, 0.068 mmol) was dissolved in MeOH/toluene (2.5 ml, 4/1, v/v). Zn dust (132 mg, 2.02 mmol) and $NH_4Cl$ (144 mg, 2.69 mmol) were added portion wise over the 2 hours. The reaction was stirred for another hour followed by filtration over silica with MeOH/toluene (1/1, v/v). This afforded the poorly soluble product which was analyzed and used without further purification.

$^{1}$H NMR (400 MHz, MeOD) δ 7.39 – 7.22 (m, 20H, $H_{arom}$), 5.52 (d, *J* = 3.6 Hz, 1H, H-1'), 4.79 (d, *J* = 11.3 Hz, 1H, $CH_2$-Bn), 4.75 (d, *J* = 11.9 Hz, 1H, $CH_2$-Bn), 4.69 (d, *J* = 9.6 Hz, 2H, $CH_2$-Bn), 4.54 (d, *J* = 11.3 Hz, 1H, $CH_2$-Bn), 4.21 (t, *J* = 9.7 Hz, 1H, H-4), 4.00 (d, *J* = 7.1 Hz, 1H, H-2), 3.95 – 3.84 (m, 3H, H-3', H-6', H-5'), 3.84 – 3.74 (m, 2H, H-3, H-6'), 3.64 (t, *J* = 9.0 Hz, 1H, H-4'), 3.50 (dd, *J* = 4.1, 1.6 Hz, 1H, $H_{epoxide}$), 3.28 (d, *J* = 3.7 Hz, 1H, $H_{epoxide}$), 3.23 (dd, *J* = 10.1, 3.6 Hz, 1H, H-2'), 3.10 (d, *J* = 9.7 Hz, 1H, H-5). HRMS (ESI) m/z: $[M+H]^+$ calculated for $C_{41}H_{46}NO_{10}$ 712.3116 found 712.3112.

**4-*O*-(2-amino-2-deoxy-α-D-glucopyranosyl)-glucurono-cyclophellitol (5)**

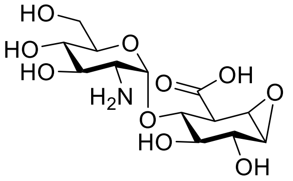


Anhydrous THF (1 ml). and *t*-BuOH (0.32 ml, 3.4 mmol) were added to benzyl protected **21** (crude, 0.068 mmol). The flask was cooled to -70°C and ammonia (5 ml) was condensed directly in the flask. Sodium (63 mg, 2.72 mmol) was added and the solution turned blue. After stirring at -60°C for 45 minutes the reaction was quenched with $NH_4Cl$ (0.18 g, 3.4 mmol)

The ammonia was evaporated, water was added and the compound was desalted by size exclusion over HW-40 (1% AcOH in water). Lyophilization afforded the compound as a white solid. (12.1 mg, 0.035 mmol, 51% over 2 steps.)

$^1$H NMR (500 MHz, $D_2O$) δ 5.49 (d, *J* = 3.8 Hz, 1H, H-1'), 3.93 – 3.85 (m, 3H, H-3', H-4, H-2), 3.83 (d, *J* = 2.9 Hz, 2H, H-6'), 3.75 (dt, *J* = 10.1, 2.9 Hz, 1H, H-5'), 3.58 – 3.50 (m, 3H, H-3, H-4', $H_{epoxide}$), 3.30 (dd, *J* = 10.7, 3.8 Hz, 1H, H-2'), 3.21 (d, *J* = 3.7 Hz, 1H, $H_{epoxide}$), 2.90 (dd, *J* = 9.8, 1.8 Hz, 1H, H-5). $^{13}$C NMR (126 MHz, $D_2O$) δ 177.7 (C-6), 96.1 (C-1'), 76.4 (C-3), 75.2 (C-2/C-3'/C-4), 72.1 (C-5'), 71.4, 69.7 (C-2/C-3'/C-4), 69.2 (C-4'), 59.7 (C-6'), 56.2 ($C_{epoxide}$), 55.4 ($C_{epoxide}$), 54.4 (C-2'), 50.9 (C-5). HRMS (ESI) m/z: $[M+H]^+$ calculated for $C_{13}H_{22}NO_{10}$ 352.1235, found 352.1238.

## 4-*O*-(2-*N*-azidoacetyl-2-deoxy-α-D-glucopyranosyl)-glucurono-cyclophellitol (6)

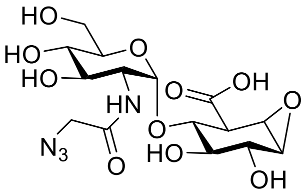

Azidoacetic acid (50 µmol) was dissolved in DMF (1 ml), 2,3,4,5,6-pentafluorophenol (46 mg, 0.26 mmol), $Et_3N$ (20 µl, 0.26 mmol) and DIC (7.8 µl, 50 µmol) were added and the mixture was stirred for 90 minutes. Of this stock solution 1.5 eq compared to amine (0.25 ml, 13 µmol) was added to amine **5** and stirred overnight. LC-MS indicated full conversion and the product was purified over HW-40 eluting with AcOH in water (1%, v/v). The fractions were concentrated under reduced pressure and lyophilized to yield the product as a white solid (0.45 mg, 1.04 µmol, 12%).

$^1$H NMR (600 MHz, $D_2O$) δ 5.17 (d, *J* = 3.8 Hz, 1H, H-1'), 4.03 (d, *J* = 1.1 Hz, 2H,$CH_2N_3$), 3.89 (dd, *J* = 10.7, 3.8 Hz, 1H, H-2'), 3.79 – 3.71 (m, 5H, H-4, H-6', H-2, H-3'), 3.69 (dt, *J* = 10.1, 3.1 Hz, 1H, H-5'), 3.50 – 3.45 (m, 3H, H-3, H-4', $H_{epoxide}$), 3.19 – 3.13 (m, 1H, $H_{epoxide}$/$Et_3N$), 2.87 (dd, *J* = 9.5, 1.9 Hz, 1H, H-5). $^{13}$C NMR (151 MHz, $D_2O$) δ 178.3 (C=O), 171.6 (C=O), 98.5 (C-1'), 77.0, 76.0 (C-2/C-3/C-3'/C-4/C-4'), 72.9 (C-5'), 72.2, 71.7, 70.4 (C-2/C-3/C-3'/C-4/C-4'), 60.9 (C-6'), 57.0 ($C_{epoxide}$), 56.4 ($C_{epoxide}$), 54.9 (C-2'), 52.7 ($CH_2N_3$), 51.5 (C-5). HRMS (ESI) m/z: $[M+H]^+$ calculated for $C_{15}H_{23}N_4O_{11}$ 435.1358, found 435.1358.

## 2,3-di-*O*-benzyl-6-*O*-(4-methoxybenzyl)-cyclophellitol alkene (12c)

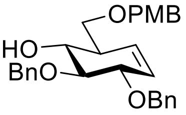

A solution of diol **12a** (1.00 g, 2.94 mmol) in anhydrous MeCN (15 mL, 0.2 M) was flushed with nitrogen and heated to 60 °C. PMB-Cl (0.59 ml, 4.41 mmol), KI (0.49 g, 2.94 mmol), $K_2CO_3$ (0.45 g, 3.23 mmol) and 2-aminoethyldiphenylborinate (0.07 g, 0.29 mmol) were added and the reaction mixture was stirred for 4 h at 60°C. After the mixture was cooled to room temperature, it was diluted with EtOAc. The organic phase was washed with $H_2O$ and brine. The aqueous layers were extracted three times with EtOAc. The combined organic phases were dried over $MgSO_4$, filtered and concentrated under reduced pressure. Purification by column chromatography (EtOAc/PE: 1/4, v/v) afforded the product as yellow oil. (0.99 g, 2.16 mmol, 73%)

$^1$H NMR (400 MHz, $CDCl_3$): δ 7.37 – 7.27 (m, 10H, $H_{arom}$), 7.25 – 7.21 (m, 2H, $H_{arom}$), 6.89 – 6.83 (m, 2H, $H_{alkene}$), 5.72 (dt, *J* = 10.2, 2.4 Hz, 1H, $H_{alkene}$), 5.60 (dt, *J* = 10.2, 1.9 Hz, 1H, $H_{epoxide}$), 4.99 (d, *J* = 11.3 Hz, 1H, $CH_2$-Bn), 4.78 (d, *J* = 11.5 Hz, 1H, $CH_2$-Bn), 4.66 (d, *J* = 11.9 Hz, 2H, $CH_2$-Bn), 4.46 (s, 2H, $CH_2$-PMB), 4.22 – 4.16 (m, 1H, H-4), 3.78 (s, 3H, $CH_3$), 3.73 – 3.51 (m, 4H, H-2, H3, H6), 2.98 (s, 1H, OH). $^{13}$C NMR (101 MHz, $CDCl_3$): δ 159.3, 138.8, 138.4, 130.3, 129.4, 128.6, 128.5, 128.3, 128.1, 128.0, 127.9, 127.8, 127.8, 126.8, 113.9 ($C_{arom}$, $C_{alkene}$), 83.9 (C-2/C-3), 80.2 (C-4), 75.0 (C-Bn), 73.1 (C-PMB), 71.6 (C-Bn), 71.3 (C-2/C-3), 70.9 (C-6), 55.4 ($CH_3$-PMB), 44.1 (C-5).

## 8-azidooctyl 4-methylbenzenesulfonate (23)

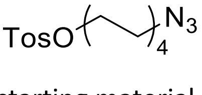

8-chlorooctan-1-ol **(22a)** (22.4 ml, 67.7 mmol) was dissolved in DMSO (34 ml, 2 M), followed by the addition of $NaN_3$ (6.60 g, 102 mmol). After stirring for 22 hours at 80 °C, NMR analysis of the reaction mixture showed total consumption of the starting material. The reaction mixture was cooled to room temperature and diluted with EtOAc. The organic layer was washed ten times with $H_2O$ and the resulting aqueous layer was extracted three times with EtOAc. The combined organic phases were dried over $MgSO_4$, filtered and concentrated

under reduced pressure. The crude azide (**22b**) was dissolved in anhydrous DCM (335 ml, 0.2 M). The solution was flushed with nitrogen and $Et_3N$ (13.9 ml, 100 mmol) was added. After 30 min, the mixture was cooled to 0 °C and TsCl (19.0 g, 100 mmol) was added dropwise. TLC monitoring after 1 hour indicated the presence of significant amount of starting material, so another portion of $Et_3N$ (13.9 ml, 100 mmol) and and TsCl (19.0 g, 100 mmol) as well as a catalytic amount of DMAP (1.63 g, 13.4 mmol) were added to the mixture. After 18 hours, the reaction was quenched with $H_2O$ and the mixture was diluted with DCM. The organic phase was washed with 1 M HCl, $NaHCO_3$ (aq. sat.) and brine, was dried over $MgSO_4$, filtered and concentrated under reduced pressure. Purification by column chromatography (EtOAc/PE: 1/20 -> 1/6, v/v) afforded the product as yellow oil. (19.9 g, 61.1 mmol, 92%)

$^1$H NMR (400 MHz, $CDCl_3$): δ 7.82 – 7.76 (m, 2H), 7.35 (d, *J* = 8.0 Hz, 2H), 4.02 (t, *J* = 6.5 Hz, 2H), 3.25 (t, *J* = 6.9 Hz, 2H), 2.45 (s, 3H), 1.59 (dq, *J* = 14.8, 6.8 Hz, 4H), 1.38 – 1.19 (m, 8H) $^{13}$C NMR (101 MHz, $CDCl_3$) δ 144.8, 133.3, 129.9, 128.0, 70.7, 51.5, 29.0, 28.9, 26.7, 25.3, 21.8.

**2,3-di-*O*-benzyl-4-(8-azidooctyl)-6-*O*-(4-methoxybenzyl)-cyclophellitol alkene (24a)**

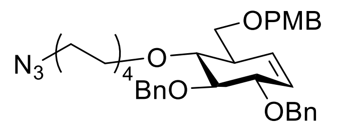


PMB-protected cyclohexene **12c** (230 mg, 500 µmol) was co-evaporated with toluene and subsequently dissolved in THF (1 ml, 0.5 M). The solution was flushed with nitrogen and cooled to 0 °C. KHMDS (0.5 M in toluene, 1.5 mL, 750 µmol) was added to the solution and the reaction mixture was stirred for 1 hour at 0°C. After 8-azidooctyl 4-methylbenzenesulfonate (**23**) (488 mg, 1.50 mmol) was added, the reaction mixture was warmed to room temperature and stirred for 18 h. The mixture was quenched by the addition of $H_2O$ and diluted with EtOAc. The aqueous phase was extracted three times with EtOAc and the combined organic phases were washed with $H_2O$ and brine. The organic phase was dried over $MgSO_4$, filtered and concentrated under reduced pressure. Purification by column chromatography (EtOAc/PE: 1/15 -> 1/4, v/v) afforded the product as colourless oil. (219 mg, 357 µmol, 71%)

$^1$H NMR (400 MHz, $CDCl_3$): δ 7.43 – 7.24 (m, 12H, $H_{arom}$), 6.92 – 6.87 (m, 2H, $H_{arom}$), 5.74 – 5.65 (m, 2H, $H_{alkene}$), 4.90 (s, 2H, $CH_2$-Bn), 4.70 (s, 2H, $CH_2$-Bn), 4.53 (d, *J* = 11.9 Hz, 1H, $CH_2$-PMB), 4.43 (d, *J* = 11.9 Hz, 1H, $CH_2$-PMB), 4.24 – 4.19 (m, 1H, H-2), 3.88 (dt, *J* = 8.9, 6.7 Hz, 1H, H-8), 3.82 (s, 3H, $CH_3$), 3.74 (dd, *J* = 10.1, 7.8 Hz, 1H, H-3), 3.56 (d, *J* = 4.0 Hz, 2H, H-6), 3.47 (t, *J* = 9.9 Hz, 1H, H-4), 3.36 (dt, *J* = 8.9, 7.0 Hz, 1H, H-8), 3.27 (t, *J* = 7.0 Hz, 2H, H-15), 2.53 – 2.41 (m, 1H, H-5), 1.69 – 1.16 (m, 12H, H-9, H-10, H-11, H-12, H-13, H-14) $^{13}$C NMR (101 MHz, $CDCl_3$): δ 159.3, 139.1, 138.6, 130.4, 129.5, 129.3, 128.5, 128.4, 127.9, 127.9, 127.7, 127.5, 127.0, 113.8 ($C_{arom}$, $C_{alkene}$), 85.3 (C-3), 80.8 (C-2), 78.7 (C-4), 75.3 (C-Bn), 73.6 (C-8), 72.9 (C-PMB), 72.2 (C-Bn), 68.9 (C-6), 55.3 ($CH_3$-PMB), 51.5 (C-14), 44.5 (C-5), 30.5, 29.5, 29.2, 28.9, 26.8, 26.2 (C-9, C-10, C-11, C-12, C-13, C-14). HRMS (ESI) m/z: $[M+Na]^+$ calculated for $C_{37}H_{47}N_3NaO_5$ 636.3413, found 636.3412.

**2,3-di-*O*-benzyl-4-(8-azidooctyl)-cyclophellitol alkene (24b)**

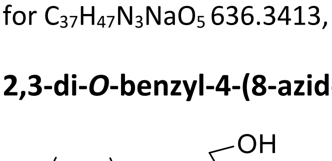


DDQ (39.0 mg, 175 µmol) was added to a solution of alkylated cyclohexene **24a** (91.0 mg, 149 µmol) in a mixture of DCM/$H_2O$ (3 ml, 19/1, v/v). After the reaction mixture was stirred for 18 hours at room temperature, the mixture was diluted with EtOAc. The organic layer was washed with $NaHCO_3$ (aq. sat.), $H_2O$ and brine. The organic phase was dried over $MgSO_4$, filtered and concentrated under reduced pressure. Purification by column chromatography (EtOAc/PE: 1/3, v/v) afforded the product as colorless oil. (62.0 mg, 126 µmol, 85%)

$^1$H NMR (400 MHz, $CDCl_3$): δ 7.40 – 7.24 (m, 10H, $H_{arom}$), 5.74 (dt, *J* = 10.1, 2.5 Hz, 1H, $H_{alkene}$), 5.53 (dt, *J* = 10.2, 1.8 Hz, 1H, $H_{alkene}$), 4.88 (d, *J* = 2.6 Hz, 2H, $CH_2$-Bn), 4.68 (s, 2H, $CH_2$-Bn), 4.21 – 4.13 (m, 1H, H-2), 3.97 (dt, *J* = 8.8, 6.9 Hz, 1H, H-8), 3.79 – 3.71 (m, 3H, H-3, H-6), 3.55 (dt, *J* = 8.8, 7.4 Hz, 1H, H-8), 3.47 (t, *J* = 9.7 Hz, 1H, H-4), 3.24 (t, *J* = 7.0 Hz, 2H, H15), 2.52 – 2.45 (m, 1H, H-5), 2.09 – 1.97 (m, 1H, OH), 1.73 – 1.16 (m, 12H, H-9, H-10, H-11, H-12, H-13, H-14)) $^{13}$C NMR (101 MHz, $CDCl_3$): δ 139.0, 138.5, 128.6, 128.5, 128.0, 127.9, 127.8, 127.7 ($C_{arom}$, $C_{alkene}$), 85.1 (C-3), 80.8 (C-2), 80.4 (C-4), 75.3 (C-Bn), 73.6 (C-8), 72.3 (C-Bn), 64.2 (C-6), 51.6 (C-15), 45.8 (C-5), 30.6, 29.5, 29.2, 28.9, 26.8, 26.2 (C-9, C-

10, C-11, C-12, C-13, C-14). HRMS (ESI) m/z: $[M+Na]^+$ calculated for $C_{29}H_{39}N_3NaO_4$ 516.2838, found 516.2834.

**2,3-di-*O*-benzyl-4-(8-azidooctyl)-cyclophellitol (25a)**

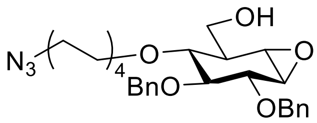

Deprotected alkylated cyclohexene **24b** (631 mg, 1.28 mmol) was dissolved in $CH_2Cl_2$ (13 ml, 0.1 M), followed by the addition of mCPBA (442 mg, 1.97 mmol) at 0°C. After stirring for 18 hours at 0°C, TLC monitoring indicated the presence of significant amount of starting material therefore mCPBA (574 mg, 2.56 mmol) was added to the reaction mixture. The mixture was stirred for another 24 hours at 0°C, followed by the dilution with EtOAc. The organic layer was washed with a mixture of $NaHCO_3/Na_2S_2O_3$ (1/1, v/v, aq. sat.) and brine. The combined aqueous layers were extracted three times with EtOAc. The combined organic layers were dried over $MgSO_4$, filtered and concentrated under reduced pressure. Purification by column chromatography (EtOAc/PE: 2/5) afforded epoxide the product as colorless oil. (571 mg, 1.12 mmol, 87%)

$^1$H NMR (400 MHz, $CDCl_3$): δ 7.42 – 7.26 (m, 10H, $H_{arom}$), 4.81 (d, *J* = 2.8 Hz, 2H, $CH_2$-Bn), 4.74 (dd, *J* = 28.7, 11.4 Hz, 2H, $CH_2$-Bn), 4.00 (dd, *J* = 10.8, 5.0 Hz, 1H, H-6), 3.92 – 3.85 (m, 2H, H-6, H-8), 3.80 (dd, *J* = 8.2, 0.5 Hz, 1H, H-2), 3.52 – 3.43 (m, 2H, H-3, H-8), 3.32 (dd, *J* = 3.6, 0.6 Hz, 1H, $H_{epoxide}$), 3.28 – 3.21 (m, 3H, H-4, H-15), 3.15 (d, *J* = 3.8 Hz, 1H, $H_{epoxide}$), 2.14 (dtd, *J* = 6.7, 5.3, 1.6 Hz, 1H, H-5), 1.62 – 1.21 (m, 12H, H-9, H-10, H-11, H-12, H-13, H-14) $^{13}$C NMR (101 MHz, $CDCl_3$): δ 138.7, 137.7, 128.6, 128.4, 128.1, 128.0, 127.8, 127.7 ($C_{arom}$), 84.9 (C-3), 79.7 (C-2), 76.3 (C-4), 75.4 (C-Bn), 73.8 (C-8), 73.3 (C-Bn), 63.1 (C-6), 55.9 ($C_{epoxide}$), 53.1 ($C_{epoxide}$), 51.5 (C-15), 44.0 (C-5), 30.4, 29.4, 29.1, 28.9, 26.7, 26.1 (C-9, C-10, C-11, C-12, C-13, C-14). HRMS (ESI) m/z: $[M+Na]^+$ calculated for $C_{29}H_{39}N_3NaO_5$ 532.2787, found 532.2782.

**2,3-di-*O*-benzyl-4-(8-azidooctyl)-glucurono-cyclophellitol (25b)**

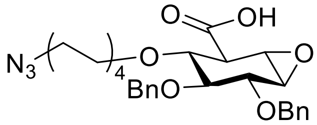

Epoxide **25a** (200 mg, 393 µmol) was dissolved in a mixture of DCM/*t*-BuOH/$H_2O$ (12 ml, 4/4/1, v/v/v). After the addition of TEMPO (12.3 mg, 78.6 µmol) and BAIB (316 mg, 982 µmol) at 0 °C, the reaction mixture was stirred for 22 hours at 0 °C. The reaction was quenched with $Na_2S_2O_3$ (aq. sat.), followed by the extraction of the acidified aqueous layer (pH = 3) three times with DCM. The combined organic layers were dried over $MgSO_4$, filtered and concentrated under reduced pressure. Purification by column chromatography (EtOAc/PE: 1/6, 1% AcOH) afforded the product as colorless oil. (185 mg, 354 µmol, 90%) To remove residual AcOH, the product was co-evaporated three times with EtOAc, toluene and chloroform.

$^1$H NMR (400 MHz, $CDCl_3$): δ 7.36 – 7.28 (m, 10H, $H_{arom}$), 4.81 (d, *J* = 1.1 Hz, 2H, $CH_2$-Bn), 4.74 (dd, *J* = 27.0, 11.3 Hz, 2H, $CH_2$-Bn), 3.90 – 3.82 (m, 2H, *H*-2, H-8), 3.65 – 3.53 (m, 2H, *H*-4, H-8), 3.52 – 3.44 (m, 2H, H-3, $H_{epoxide}$), 3.24 (t, *J* = 7.0 Hz, 2H, H-15), 3.20 (d, *J* = 3.6 Hz, 1H, $H_{epoxide}$), 2.98 (dd, *J* = 10.0, 1.6 Hz, 1H, H-5), 1.61 – 1.18 (m, 12H, H-9, H-10, H-11, H-12, H-13, H-14). $^{13}$C NMR (101 MHz, $CDCl_3$): δ 175.3, 138.6, 137.7, 128.7, 128.5, 128.2, 128.1, 127.8, 127.8 ($C_{arom}$), 84.2 (C-3), 79.3 (C-2), 75.5 (C-Bn), 74.9 (C-4), 73.9 (C-8), 73.5 (C-Bn), 54.4 ($C_{epoxide}$), 53.8 ($C_{epoxide}$), 51.6 (C-15), 48.6 (C-5), 30.3, 29.3, 29.1, 28.9, 26.7, 25.9 (C-9, C-10, C-11, C-12, C-13, C-14). HRMS (ESI) m/z: $[M+Na]^+$ calculated for $C_{29}H_{37}N_3NaO_6$ 546.2580, found 546.2580.

**4-(8-aminooctyl)-glucurono-cyclophellitol (26)**

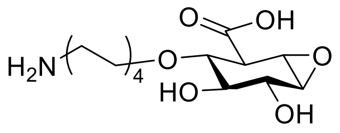

In a 25 ml-flask, carboxylic acid **25** (40.0 mg, 76.5 µmol) was dissolved in anhydrous THF, followed by the addition of *t*-BuOH (73 µl, 765 µmol) and a glass-coated stirring bar. The flask was flushed with nitrogen and attached to a flow of gas ammonia. By cooling the reaction mixture to -78 °C, 7 ml of liquid ammonia was condensed. Freshly cut sodium (35.0 mg, 1.53 mmol) was added portion wise at the same temperature under a flow of nitrogen. After stirring for 30 minutes, the reaction was quenched with AcOH (21 eq.), warmed to room temperature and

stirred for 1 hour. The reaction mixture was concentrated under reduced pressure and co-evaporated three times with $H_2O$ using a water-jet-pump. Preparative RP-HPLC (linear gradient, solutions used: A: 50 mM AcOH in $H_2O$, B: $CH_3CN$) and subsequent lyophilisation gave the amine product. (6.70 mg, 21.1 µmol, 28%)

$^1H$ NMR (500 MHz, $D_2O$): δ 3.80 (d, *J* = 8.4 Hz, 1H, H-2), 3.73 (dt, *J* = 9.0, 6.2 Hz, 1H, H-8), 3.58 (dt, *J* = 9.0, 6.9 Hz, 1H, H-8), 3.49 – 3.44 (m, 2H, H-4, $H_{epoxide}$), 3.37 (dd, *J* = 10.4, 8.5 Hz, 1H, H-3), 3.15 (d, *J* = 3.7 Hz, 1H, $H_{epoxide}$), 2.97 (t, *J* = 7.6 Hz, 2H, H-15), 2.72 (dd, *J* = 9.8, 1.8 Hz, 1H, H-5), 1.67 – 1.24 (m, 12H, H-9, H-10, H-11, H-12, H-13, H-14). $^{13}C$ NMR (126 MHz, $D_2O$): δ 178.0 (C=OO), 76.9 (C-4), 75.8 (C-3), 73.0 (C-7), 71.2 (C-2), 56.5 ($C_{epoxide}$), 55.5 ($C_{epoxide}$), 51.0 (C-5), 39.5 (C-15), 29.2, 28.0, 27.9, 26.6, 25.3, 25.0 (C-9, C-10, C-11, C-12, C-13, C-14). HRMS (ESI) m/z: $[M+H]^+$ calculated for $C_{15}H_{28}NO_6$ 318.1917, found 318.1913.

## 4-(8-Cy5-aminooctyl)-glucurono-cyclophellitol (8)

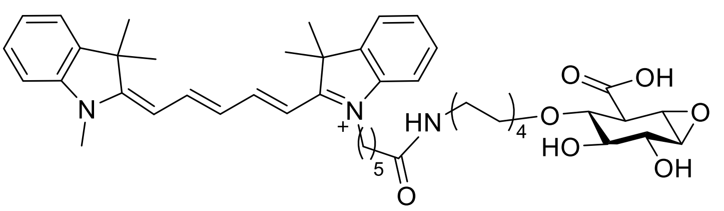


Crude amine **26** (10.3 mg, 32.5 µmol) was dissolved in anhydrous DMF (0.5 mL), followed by the addition of Cy5-NHS ester (30.0 mg, 48.8 µmol) and DIPEA (10.6 µl, 65.0 µmol). After the reaction mixture was stirred for 18 hours, it was purified by preparative RP-HPLC (linear gradient, solutions used: A: 50 mM AcOH in $H_2O$, B: $CH_3CN$) and lyophilized. The product was obtained as blue solid. (3.50 mg, 4.48 µmol, 14%)

$^1H$ NMR (500 MHz, MeOD): δ 8.25 (t, *J* = 13.0 Hz, 2H), 7.49 (d, *J* = 7.5 Hz, 2H), 7.44 – 7.38 (m, 2H), 7.32 – 7.24 (m, 4H), 6.64 (t, *J* = 12.4 Hz, 1H), 6.29 (dd, *J* = 13.7, 6.5 Hz, 2H), 4.11 (t, *J* = 7.4 Hz, 2H), 3.79 – 3.71 (m, 1H), 3.69 (d, *J* = 8.2 Hz, 1H), 3.67 – 3.59 (m, 4H), 3.48 (t, *J* = 9.8 Hz, 1H), 3.39 (m, 1H), 3.27 (dd, *J* = 10.1, 8.3 Hz, 1H), 3.12 (t, *J* = 7.0 Hz, 2H), 2.99 (d, *J* = 3.6 Hz, 1H), 2.69 (dd, *J* = 8.1, 1.5 Hz, 1H), 2.20 (t, *J* = 7.2 Hz), 1.87 – 1.79 (m, 2H), 1.73 (s, 12H), 1.71 – 1.66 (m, 2H), 1.47 – 1.24 (m, 14H) $^{13}C$ NMR (126 MHz, MeOD): δ 155.5, 144.3, 143.6, 142.7, 142.5, 129.8, 129.7, 126.7, 126.3, 123.4, 123.3, 112.1, 111.9, 104.4, 78.2, 77.9, 73.9, 73.0, 56.9, 56.7, 52.0, 50.6, 50.5, 44.8, 40.4, 36.7, 31.6, 31.3, 30.5, 30.3, 30.3, 28.2, 28.0, 27.9, 27.8, 27.4, 27.0, 26.6 HRMS (ESI) m/z: $[M]^+$ calculated for $C_{47}H_{64}N_3O_7$ 782.4739, found 782.4772.

## 8-benzyloxycarbonylamino-octan-1-ol (28a)

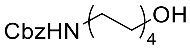


8-Amino-octan-1-ol **27** (6.65 g, 45.8 mmol) was dissolved in acetone/water (700 ml, 2/1, v/v). $NaHCO_3$ (11.53 g, 137.3 mmol) was added followed by dropwise addition of carboxybenzyl chloride (9.78 ml, 68.7 mmol). After TLC showed full conversion the acetone was removed in vacuo. The remaining water layer was extracted with EtOAc (3x). The combined organic layers were washed with brine, dried over $MgSO_4$ and concentrated in vacuo. The product was obtained by column chromatography (EtOAc/pentane, 3/7, v/v) as an oil. (11.26 g, 40.30 mmol, 88%)

$^1H$ NMR (400 MHz, $CDCl_3$) δ 7.39 – 7.28 (m, 5H), 5.09 (s, 2H), 3.63 (t, *J* = 6.6 Hz, 2H), 3.18 (q, *J* = 6.7 Hz, 2H), 1.62 – 1.40 (m, 4H), 1.41 – 1.23 (m, 8H). $^{13}C$ NMR (101 MHz, $CDCl_3$) δ 156.5, 136.8, 128.6, 128.3, 128.2, 66.7, 63.1, 41.2, 32.8, 30.1, 29.4, 29.3, 26.7, 25.8. HRMS (ESI) m/z: $[M+Na]^+$ calculated for $C_{16}H_{25}NO_3Na$ 302.1727, found 302.1734.

## 8-benzyloxycarbonylamino-1-trityloxy octane (28b)

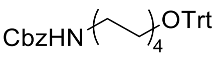


Alcohol **28a** (9.8 g, 35 mmol) was dissolved in DMF (58 ml, 0.6 M). Trityl chloride (13.7 g, 175 mmol) and $Et_3N$ (24.5 ml, 175 mmol) were added and the reaction was stirred for 24h. Trityl chloride (4.4 g, 17.5 mmol) and $Et_3N$ (5.0 ml, 36 mmol) were added and the reaction was stirred for 48 h. Upon completion the mixture was diluted with $H_2O$ and extracted three times with $Et_2O$. The combined organic layers were washed with $H_2O$ (5x) and brine, dried over $MgSO_4$, filtered and concentrated in vacuo. The product was used without further purification.

$^{1}$H NMR (400 MHz, $CDCl_3$) δ 7.47 – 7.41 (m, 6H), 7.37 – 7.25 (m, 11H), 7.25 – 7.19 (m, 3H), 5.09 (s, 2H), 4.71 (s, 1H), 3.17 (q, *J* = 6.7 Hz, 2H), 3.03 (t, *J* = 6.6 Hz, 2H), 1.65 – 1.55 (m, 2H), 1.51 – 1.40 (m, 2H), 1.39 – 1.19 (m, 8H). $^{13}$C NMR (101 MHz, $CDCl_3$) δ 156.5, 144.6, 136.8, 128.8, 128.6, 128.3, 128.2, 127.8, 126.9, 86.4, 66.7, 63.7, 41.2, 30.1, 30.1, 29.5, 29.3, 26.8, 26.3. HRMS (ESI) m/z: $[M+Na]^+$ calculated for $C_{35}H_{39}NO_3Na$ 544.2822, found 544.2830.

## 8-*N*-benzyl(benzyloxycarbonyl)-1-trityloxy octane (29b)

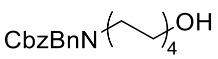


Carbamate **28b** (18.3 g, 35 mmol) was dissolved in DMF (96 ml, 0.36 M) and the solution was cooled to 0°C. NaH (60 wt%, 2.9 g, 74 mmol) was added and the reaction was stirred at 0°C for 10 minutes. Benzyl bromide (5.9 ml, 49 mmol) and TBAI (1.3 g, 3.5 mmol) were added and the solution was stirred overnight at room temperature. The reaction mixture was cooled down to 0°C and quenched with $H_2O$. It was then further diluted with $H_2O$ and extracted with $Et_3O$ (3x). The combined organic layers were washed with $H_2O$ (5x) and brine, dried over $MgSO_4$, filtered and concentrated under reduced pressure. Column chromatography ($Et_2O$/PE, 2/98 -> 1/4, v/v) yielded the product. (16.3 g, 26.7 mmol, 76%, over 2 steps)

Trityl ether **29a** (16.3 g, 26 mmol) was dissolved in DCM/MeOH (104 ml, 1/1, 0.25 M). TFA (3% in $H_2O$, 7.5 ml, 3.0 mmol) was added and the reaction was stirred for 27 hours. More TFA (0.23 ml, 3.0 mmol) was added and the reaction was stirred for another 24 hours. TLC showed complete conversion and the reaction was quenched with $NaHCO_3$, concentrated in vacuo, dissolved in EtOAc and washed three times with $H_2O$ and brine, dried over $MgSO_4$, filtrated and concentrated in vacuo. Column chromatography (EtOAc/PE, 1/9 -> 3/7, v/v) afforded the product. (8.1 g, 21.9 mmol, 84%)

$^{1}$H NMR (400 MHz, $CDCl_3$) δ 7.40 – 7.12 (m, 10H), 5.16 (d, *J* = 13.1 Hz, 2H), 4.48 (d, 2H), 3.57 (t, *J* = 6.7 Hz, 2H), 3.29 – 3.14 (m, 2H), 2.32 (s, 1H), 1.57 – 1.43 (m, 4H), 1.38 – 1.14 (m, 8H). $^{13}$C NMR (101 MHz, $CDCl_3$) δ 156.8, 156.2, 137.9, 136.8, 136.7, 128.5, 128.4, 127.9, 127.8, 127.3, 67.1, 62.7, 50.4, 50.1, 47.2, 46.2, 32.6, 29.2, 29.2, 28.0, 27.6, 26.6, 25.6. HRMS (ESI) m/z: $[M+Na]^+$ calculated for $C_{23}H_{31}NO_3Na$ 392.2196, found 392.2200.

## 8-*N*-benzyl(benzyloxycarbonyl)-1-iodo-octane(30)

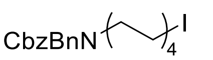


Alcohol **29b** (8.1 g, 21.3 mmol) was dissolved in DCM (250 ml, 0.085 M) and cooled to 0°C. $PPh_3$ (6.7 g, 25.6 mmol), imidazole (1.7 g, 25.6 mmol) and $I_2$ (6.5 g, 26 mmol) were added to the solution. After stirring for 1 hour at 0 °C the reaction was warmed to rt and stirred for an additional 4 hours. The reaction was quenched with $NaHCO_3$ (aq. sat.) and the water layer was extracted three times with DCM. The combined organic layers were washed with $NH_4Cl$ (aq. sat.), $NaHCO_3$ (aq. sat.), $Na_2S_2O_3$ (aq. sat.) and brine, dried over $MgSO_4$, filtrated and concentrated in vacuo. Column chromatography (EtOAc/PE, 1/24 -> 1/4, v/v) yielded the product. (10.4 g, 21.6 mmol, 97%)

$^{1}$H NMR (400 MHz, $CDCl_3$) δ 7.42 – 7.13 (m, 10H), 5.17 (d, *J* = 11.8 Hz, 2H), 4.49 (d, *J* = 8.0 Hz, 2H), 3.30 – 3.11 (m, 4H), 1.83 – 1.70 (m, 2H), 1.58 – 1.41 (m, 2H), 1.41 – 1.15 (m, 8H). $^{13}$C NMR (101 MHz, $CDCl_3$) δ 156.8, 156.2, 138.0, 128.6, 128.5, 128.0, 127.9, 127.3, 67.2, 50.5, 50.2, 47.2, 46.3, 33.5, 30.4, 29.1, 28.4, 28.1, 27.7, 26.7, 7.4. HRMS (ESI) m/z: $[M+Na]^+$ calculated for $C_{23}H_{30}INO_2Na$ 502.1213, found 502.1216.

## Phenyl 2-azido-2-deoxy-3-*O*-benzyl-6-*O*-(2-Naphthylmethyl)-1-thio-α-D-glucopyranoside (31b)

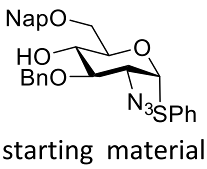


Phenyl 2-azido-2-deoxy-3-*O*-benzyl-4,6-benzylidene-1-thio-α-D-glucopyranoside (**31a**, 1.70 g, 3.6 mmol) was suspended in MeOH (30 mL). DCE (5 ml) was added to obtain a clear solution. The solution was heated to 50°C and camphorsulfonic acid (0.42 g, 1.8 mmol) was added. When TLC analysis showed full conversion of the starting material the reaction was cooled down to 0°C and quenched with $Et_3N$. Solvents were removed in vacuo and the crude residue was dissolved in EtOAc, washed with HCl (aq. 1M), $NaHCO_3$ (aq. sat.), brine and dried over $MgSO_4$.

The solvent was evaporated and the crude diol was dissolved in anhydrous MeCN (15 ml). 2-aminoethyl diphenyl borinate (0.081 g, 0.36 mmol), 2-bromomethyl-naphtalene (1.2 g, 5.4 mmol), KI (0.60 g, 3.6 mmol) and $K_2CO_3$ (0.55 g, 4.0 mmol) were added. The mixture was heated to 60°C and stirred overnight. The reaction mixture was transferred to a separatory funnel containing EtOAc and $H_2O$, layers were separated and the water layer was re-extracted three times with EtOAc. The organic layers were combined, washed with brine, dried over $MgSO_4$, filtered and concentrated in vacuo. Column chromatography (EtOAc/pentane, 1/9 -> 1/3, v/v) afforded the product. (1.77 g, 3.35 mmol, 93%)

$^1$H NMR (400 MHz, Chloroform-*d*) δ 7.84 – 7.17 (m, 17H, $H_{arom}$), 5.54 (d, *J* = 5.4 Hz, 1H, H-1), 4.91 (d, *J* = 11.1 Hz, 1H, CH2-Bn/Nap), 4.80 (d, *J* = 11.1 Hz, 1H, $CH_2$-Bn/Nap), 4.71 – 4.58 (m, 2H, $CH_2$-Bn/Nap), 4.34 (dt, *J* = 9.6, 4.2 Hz, 1H, H-5), 3.85 (dd, *J* = 10.0, 5.4 Hz, 1H, H-2), 3.78 – 3.66 (m, 3H, H-4, H-6), 3.66-3.62 (m, 1H, H-3) 2.66 (d, *J* = 3.2 Hz, 1H, OH). $^{13}$C NMR (101 MHz, $CDCl_3$) δ 138.0, 135.3, 133.3, 132.0, 129.2, 128.8, 128.4, 128.3, 128.3, 128.0, 127.9, 127.9, 126.7, 126.3, 126.1, 125.7 ($C_{arom}$), 87.4 (C-1), 81.5 (C-3), 73.8(C-Bn/Nap), 72.2 (C-5), 71.3 (C-4), 69.7 (C-6), 63.7 (C-2). HRMS (ESI) m/z: $[M+Na]^+$ calculated for $C_{30}H_{29}N_3O_4SNa$ 550.1771, found 550.1771.

### Phenyl 2-azido-2-deoxy-3-*O*-benzyl-4-*O*-(8-*N*-benzyl-(benzyloxycarbonyl)-1-octyl)-6-*O*-(2-Naphthylmethyl)-1-thio-α-D-glucopyranoside (32a)

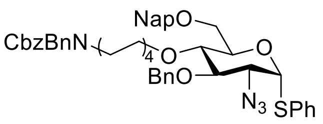


Alcohol **31b** (2.3 g, 6.2 mmol) and iodide **30** (7.7 g, 16 mmol) were dissolved in dry DMF (13 ml, 0.5 M) and the solution was cooled to 0°C. NaH (60% dispersion in mineral oil, 0.43 g, 10.7 mmol) was added and the reaction was stirred for 18 hours at room temperature. The reaction mixture was cooled to 0°C and quenched with water. The water layer was extracted with $Et_2O$ (3x), the combined organic layers were washed five times with water and brine, dried over $MgSO_4$, filtrated and concentrated in vacuo. Column chromatography ($Et_2O$/PE, 1/49 -> 1/4, v/v) yielded the product. (4.4 g, 5.4 mmol, 88%)

$^1$H NMR (400 MHz, Chloroform-*d*) δ 7.85 – 7.71 (m, 5H, $H_{arom}$), 7.54 – 7.12 (m, 22H, $H_{arom}$), 5.60 (d, *J* = 5.4 Hz, 1H, H-1), 5.17 (d, *J* = 9.5 Hz, 2H, $CH_2$-Cbz linker), 4.91 – 4.75 (m, 3H, $CH_2$-Bn/Nap), 4.60 (d, *J* = 12.1 Hz, 1H, $CH_2$-Bn/Nap), 4.49 (d, *J* = 8.5 Hz, 2H, $CH_2$-Bn), 4.30 (d, *J* = 9.7 Hz, 1H, H-5), 3.91 (dd, *J* = 10.3, 5.4 Hz, 1H, H-2), 3.83 – 3.64 (m, 4H, H-3, H-6, H-8), 3.54 (t, *J* = 9.4 Hz, 1H, H-4), 3.39 (m, 1H, H-8), 3.20 (m, *J* = 26.6 Hz, 2H, H-15), 1.41 (m, *J* = 21.9 Hz, 4H, H-9, H-14), 1.10 (s, 8H, H-10, H-11, H-12, H-13). $^{13}$C NMR (101 MHz, $CDCl_3$) δ 138.0, 135.5, 133.4, 133.2, 132.2, 129.3, 129.2, 128.8, 128.7, 128.7, 128.7, 128.7, 128.7, 128.6, 128.4, 128.4, 128.3, 128.2, 128.2, 128.2, 128.1, 128.1, 128.0, 127.9, 127.9, 127.8, 127.8, 127.5, 127.4, 127.4, 126.5, 126.4, 126.2 ($C_{arom}$), 87.5 (C-1), 82.0 (C-3), 77.9 (C-4), 76.0, 73.8 (C-Bn/Nap), 73.6 (C-8) 72.2 (C-5), 68.5 (C-6), 67.4 (C-Cbz), 64.2 (C-2), 50.4 (C-Cbz), 47.4 (C-15) 30.7, 30.6, 30.0, 29.5, 27.0, 26.3 ($CH_2$). HRMS (ESI) m/z: $[M+Na]^+$ calculated for $C_{53}H_{58}N_4O_6SNa$ 901.3969, found 901.3969.

### Phenyl 2-azido-2-deoxy-3-*O*-benzyl-4-*O*-(8-*N*-benzyl-(benzyloxycarbonyl)-1-octyl)-1-thio-D-glucopyranoside (32b)

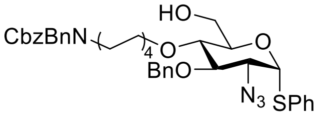


Naphtyl ether **32a** (4.8 g, 5.5 mmol) was dissolved in DCM/MeOH (38 ml, 0.15 M, 4/1, v/v) and flushed with $N_2$ for 15 min. DDQ (3.7 g, 16 mmol) was added and the reaction was stirred for 2.5 hours in the dark. The reaction mixture was concentrated in vacuo and the residue was dissolved in EtOAc. The organic layer was washed with $NaHCO_3$ (aq. sat. 3x) and brine, dried over $MgSO_4$, filtrated and concentrated in vacuo. Column chromatography ($Et_2O$/PE, 3/47 -> 2/3. v/v) yielded the product. (2.6 g, 3.9 mmol, 72%)

$^1$H NMR (400 MHz, Chloroform-*d*) δ 7.52 – 7.47 (m, 2H, $H_{arom}$), 7.42 – 7.14 (m, 18H, $H_{arom}$), 5.55 (d, *J* = 5.3 Hz, 1H, H-1), 5.17 (d, *J* = 12.9 Hz, 2H, $CH_2$-Cbz), 4.88 (s, 2H, $CH_2$-Bn), 4.53 – 4.46 (m, 2H, $CH_2$-Bn), 4.19 (dt, *J* = 10.0, 3.3 Hz, 1H, H-5), 3.85 (dd, *J* = 10.3, 5.3 Hz, 1H, H-2), 3.77 (m, 4H, H-3, H-6, H-8), 3.58 (d, *J* = 7.9 Hz, 1H, H-8), 3.43 (t, *J* = 9.4 Hz, 1H, H-4), 3.30 – 3.14 (m, 2H, H-15), 1.56 – 1.44 (m, 4H, H-9,

H-14), 1.25 (s, 8H, H-10, H-11, H-12, H-13). $^{13}$C NMR (101 MHz, $CDCl_3$) δ 132.8, 129.4, 128.8, 128.7, 128.7, 128.4, 128.2, 128.2, 128.1 ($C_{arom}$), 87.3 (C-1), 81.8 (C-3), 78.6 (C-4), 75.9 (C-Bn), 73.7 (C-8), 72.8 (C-5), 67.4 (C-Cbz), 64.2 (C-2), 61.8 (C-6), 50.4 (C-Bn), 46.4 (C-15) 30.6, 29.5, 26.9, 26.3 ($CH_2$). HRMS (ESI) m/z: $[M+Na]^+$ calculated for $C_{42}H_{50}N_4O_6SNa$ 761.3343, found 761.3365.

**Phenyl 2-azido-2-deoxy-3-*O*-benzyl-4-*O*-(8-*N*-benzyl-(benzyloxycarbonyl)-1-octyl)-6-*O*-tert-butyldiphenylsilyl-1-thio-α-D-glucopyranoside (32c)**

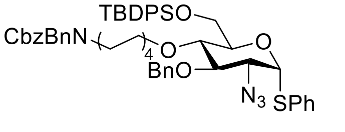


Alcohol **32b** (3.4 g, 4.5 mmol) was dissolved in DMF (22.8 ml, 0.2 M). TBDPSCl (2.5 g, 9.1 mmol) and imidazole (1.6 g, 22.8 mmol) were added. After stirring for 17 hours TLC showed complete conversion and the reaction was quenched with $H_2O$. The reaction mixture was then diluted further with $H_2O$ and extracted five times with $Et_2O$. The combined organic layers were washed five times with $H_2O$ and brine, dried over $MgSO_4$, filtrated and concentrated in vacuo. Column chromatography ($Et_2O$/PE, 3/47, v/v) yielded the product. (4.3 g, 4.4 mmol, 95% contaminated with excess silyl reagent)

$^1$H NMR (400 MHz, Chloroform-*d*) δ 7.76 – 7.63 (m, 8H, $H_{arom}$), 7.48 – 7.13 (m, 27, $H_{arom}$), 5.59 (d, *J* = 5.4 Hz, 1H, H-1), 5.17 (d, *J* = 10.4 Hz, 2H, $CH_2$-Cbz), 4.88 (s, 2H, $CH_2$-Bn) 4.49 (d, *J* = 7.9 Hz, 2H, $CH_2$-Bn), 4.14 (d, *J* = 9.5 Hz, 1H, H-5), 3.94 (dd, *J* = 11.6, 3.3 Hz, 1H, H-6), 3.88 (dd, *J* = 10.2, 5.4 Hz, 1H, H-2), 3.86 – 3.80 (m, 2H, H-6, H-8), 3.73 (dd, *J* = 10.2, 8.9 Hz, 1H, H-3), 3.68 – 3.60 (m, 2H, H-4, H-8), 3.21 (d, *J* = 27.3 Hz, 2H, H-15), 1.53 (s, 4H, H-9, H-14), 1.22 (t, *J* = 14.6 Hz, 8H, H-10, H-11, H-12, H-13), 1.03 (s, 9H, TBDPS). $^{13}$C NMR (101 MHz, $CDCl_3$) δ 136.1, 135.8, 135.0, 131.6, 129.9, 129.2, 128.8, 128.8, 128.6, 128.3, 128.0, 127.9, 127.8, 127.5 ($C_{arom}$), 87.3 (C-1), 82.0 (C-3), 78.4 (C-4), 76.1 (C-Bn), 73.7 (C-8), 73.3 (C-5), 67.4 (C-Cbz), 64.3 (C-2), 62.5 (C-6), 30.7 ($CH_2$), 26.8 (TBDPS), 26.5 ($CH_2$), 19.6 (TBDPS, quaternary C). HRMS (ESI) m/z: $[M+Na]^+$ calculated for $C_{58}H_{68}N_4O_6SSiNa$ 999.4521, found 999.4539.

**2-azido-2-deoxy-3-*O*-benzyl-4-*O*-(8-*N*-benzyl-(benzyloxycarbonyl)-1-octyl)-6-*O*-tert-butyldiphenylsilyl-D-glucopyranose (33a)**

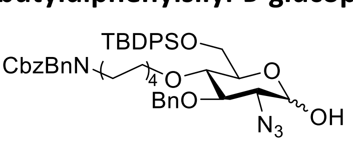


Thioglycoside **32c** (6.2 g, 6.3 mmol) was dissolved in acetone/$H_2O$/DCM (49.5 ml, 0.13 M, 9/1/1, v/v). The reaction mixture was flushed with $N_2$. NIS (3.1 g, 13.5 mmol) was added and the reaction was stirred in the dark for 7 hours. The reaction was quenched with solid $Na_2S_2O_3$. The reaction mixture was concentrated in vacuo and dissolved in EtOAc. The organic layer was washed with $Na_2S_2O_3$ (aq. sat.) and brine, dried over $MgSO_4$, filtrated and concentrated in vacuo. Column chromatography ($Et_2O$/PE, 1/20 -> 1/4, v/v) yielded the product. (4.4 g, 5.0 mmol, 79%, α/β = 1/5)

$^1$H NMR (400 MHz, $CDCl_3$) δ 7.75 – 7.63 (m, 10H), 7.46 – 7.12 (m, 54H), 5.25 (t, *J* = 3.2 Hz, 1H), 5.17 (d, *J* = 11.4 Hz, 5H), 4.88 – 4.77 (m, 5H), 4.52 – 4.41 (m, 6H), 3.97 – 3.75 (m, 9H), 3.67 – 3.48 (m, 5H), 3.41 – 3.13 (m, 12H), 3.00 (s, 1H), 1.54 – 1.41 (m, 9H), 1.27 – 1.11 (m, 21H), 1.08 – 1.00 (m, 23H). $^{13}$C NMR (101 MHz, $CDCl_3$) δ 138.0, 136.1, 135.7, 129.8, 128.6, 128.3, 128.0, 127.8, 127.7, 96.1, 92.1, 83.1, 80.1, 78.2, 77.6, 76.3, 75.7, 73.4, 72.2, 67.6, 67.3, 64.2, 62.8, 62.6, 30.5, 29.6, 29.4, 27.0, 26.9, 26.2, 19.4. HRMS (ESI) m/z: $[M+Na]^+$ calculated for $C_{52}H_{64}N_4O_7SiNa$ 907.4436, found 907.4436.

**2-azido-2-deoxy-3-*O*-benzyl-4-*O*-(8-*N*-benzyl-(benzyloxycarbonyl)-1-octyl)-6-*O*-tert-butyldiphenylsilyl-D-glucopyranosyl N-phenyltrifluoroacetimidate (33b)**

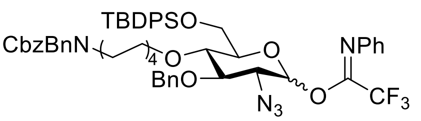


Lactol **33a** (3.8 g, 4.3 mmol) was dissolved in DCM (3.5 ml, 0.2 M). 2,2,2-trifluoro-*N*-phenylacetimidoyl chloride (1.0 ml, 6.4 mmol) and $Cs_2CO_3$ (2.1 g, 6.4 mmol) were added. And the mixture was stirred for 6.5 hours. Upon completion the suspension was filtrated over celite and the filtrate was concentrated under reduced pressure. Column chromatography ($Et_2O$/PE, 1/20 -> 9/10, v/v) yielded the product. (4.2 g, 4.0 mmol, 94%)

$^{1}$H NMR (400 MHz, $CDCl_3$) δ 7.74 – 7.64 (m, 4H), 7.46 – 7.02 (m, 26H), 6.80 (d, *J* = 7.7 Hz, 1H), 6.72 (d, *J* = 7.4 Hz, 1H), 5.17 (d, *J* = 10.6 Hz, 2H), 4.94 – 4.79 (m, 2H), 4.49 (d, *J* = 7.4 Hz, 2H), 3.98 – 3.78 (m, 3H), 3.78 – 3.53 (m, 3H), 3.31 – 3.14 (m, 2H), 1.52 (s, 4H), 1.21 (d, *J* = 16.1 Hz, 8H), 1.05 (s, 9H). $^{13}$C NMR (101 MHz, $CDCl_3$) δ 138.0, 137.8, 135.9, 135.6, 133.7, 133.6, 132.9, 129.9, 129.8, 129.4, 128.9, 128.8, 128.7, 128.6, 128.5, 128.4, 128.3, 128.2, 128.0, 127.9, 127.8, 127.7, 127.4, 127.4, 127.3, 126.3, 124.5, 124.4, 120.6, 119.4, 82.9, 80.3, 77.6, 76.6, 75.9, 74.7, 73.7, 73.5, 67.2, 65.4, 63.0, 62.0, 50.7, 50.6, 50.2, 47.3, 46.4, 30.5, 30.5, 29.7, 29.6, 29.4, 28.2, 27.8, 26.9, 26.9, 26.3, 19.5.

**2,3-di-*O*-benzyl-4-*O*-(2-azido-2-deoxy-3-*O*-benzyl-4-*O*-(8-*N*-benzyl-(benzyloxycarbonyl)-1-octyl)-6-*O*-tert-butyldiphenylsilyl-α-D-glucopyranosyl)-6-*O*-benzoyl-cyclophellitol alkene (34)**

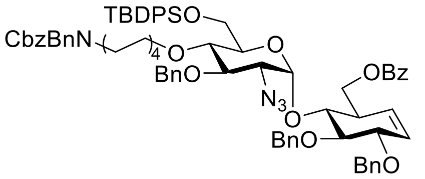


Donor **33b** (1.3 g, 1.3 mmol) and acceptor **12b** (0.39 g, 0.9 mmol) were co-evaporated thrice with toluene and dissolved in dry DCM (10.6 ml, 0.068 M). 3Å molecular sieves were added and the mixture was stirred for 1 hour. The mixture was cooled to -78 °C and TfOH (0.1 M in DCM, 2.8 ml, 0.28 mmol) was added. The reaction mixture was slowly warmed to -30 °C within 1.5 hours and stirred for an additional hour at this temperature. The reaction was quenched with $Et_3N$ at -30 °C and diluted with DCM. The organic layer was washed with water and the water layer was extracted with DCM (2x). The combined organic layers were washed with brine, dried over $MgSO_4$, filtrated and concentrated in vacuo. Column chromatography (EtOAc/pentane, 1/20 -> 1/3, v/v) yielded the product. (1.2 g, 0.88 mmol, 97%)

$^{1}$H NMR (400 MHz, $CDCl_3$) δ 7.86 – 7.12 (m, 45H, $H_{arom}$), 5.81 (dt, *J* = 10.2, 2.4 Hz, 1H, $H_{alkene}$), 5.69 (d, *J* = 4.0 Hz, 1H, H-1'), 5.61 (dt, *J* = 10.2, 2.2 Hz, 1H, $H_{alkene}$), 5.17 (d, *J* = 11.5 Hz, 2H, $CH_2$-Cbz), 5.10 – 4.96 (m, 2H, $CH_2$-Bn), 4.86 (q, *J* = 10.5 Hz, 2H, $CH_2$-Bn), 4.67 (q, J=12 Hz, 2H, $CH_2$-Bn), 4.54 – 4.45 (m, 3H, $CH_2$-Bn, H-6), 4.35 – 4.24 (m, 2H, H-2, H-6), 4.00 (dd, *J* = 9.7, 8.4 Hz, 1H, H-4), 3.97 – 3.91 (m, 2H, H-3', H-3), 3.88 – 3.76 (m, 2H, H-6', H-8), 3.72 – 3.57 (m, 4H, H-4', H-5', H-6', H-8), 3.31 – 3.15 (m, 3H, H-2', H-15), 2.79 (dq, *J* = 6.4, 3.0 Hz, 1H, H-5), 1.49 (s, 4H, H-9, H-14), 1.21 – 1.14 (m, 8H, H-10, H-11, H-12, H-13), 0.98 (s, 9H, TBDPS). $^{13}$C NMR (101 MHz, $CDCl_3$) δ 138.9, 138.2, 138.1, 137.0, 135.9, 135.8, 135.6, 135.6, 133.7, 133.3, 133.0, 129.8, 129.7, 129.6, 129.6, 129.5, 128.6, 128.5, 128.5, 128.4, 128.4, 128.3, 128.2, 128.0, 127.9, 127.9, 127.8, 127.7, 127.6, 127.5, 127.5 ($C_{arom}$, $C_{alkene}$), 98.0 (C-1'), 84.4 (C-3'/C-3), 80.9 (C-2), 80.0 (C-3'/C-3),77.9 (C-4'/C-5'), 75.5 (C-Bn), 74.8 (C-Bn), 74.1 (C-4), 73.2 (C-6'), 72.9 (C-4'/C-5'), 71.7 (C-Bn), 67.2 (C-Cbz), 64.3 (C-6), 63.4 (C-2'), 62.1 (C-8), 50.5 (C-Bn), 46.3 (C-15), 43.1 (C-5), 30.5, 29.8, 29.6, 29.4, 28.2, 27.8 ($CH_2$), 26.9 (TBDPS), 26.9, 26.8, 26.2 ($CH_2$), 19.4 (TBDPS quaternary C). HRMS (ESI) m/z: $[M+Na]^+$ calculated for $C_{80}H_{90}N_4O_{11}SiNa$ 1333.6268, found 1333.6309.

**2,3-di-*O*-benzyl-4-*O*-(2-*N*-acetyl-2-deoxy-3-*O*-benzyl-4-*O*-(8-*N*-benzyl-(benzyloxycarbonyl)-1-octyl)-6-*O*-tert-butyldiphenylsilyl-α-D-glucopyranosyl)-6-*O*-benzoyl-cyclophellitol alkene (35a)**

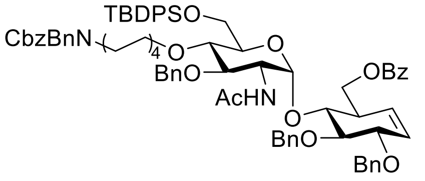


Azide **34** (1.2 g, 0.88 mmol) was dissolved in THF/$H_2O$ (10 ml, 0.085 M, 17/3, v/v). $PPh_3$ (0.58 g, 2.2 mmol) and pyridine (0.080 ml, 1.0 mmol) were added. The reaction mixture was heated to 50 °C and stirred for 3 hours. The reaction mixture was concentrated in vacuo. The residue was dissolved in DCM, dried with $MgSO_4$, and concentrated in vacuo.

The crude product was co-evaporated with toluene and dissolved in DCM (8.8 ml, 0.1 M). Acetic anhydride (1.40 ml, 13.4 mmol) and pyridine (1.2 ml, 13.4 mmol) were added and the reaction mixture was stirred for 16.5 hours. The mixture was cooled to 0 °C and quenched with water. The organic layer was washed with $CuSO_4$ (aq. sat.), $NaHCO_3$ (aq. sat.) and brine, dried over $MgSO_4$, filtrated and concentrated in vacuo. Column chromatography (EtOAc/PE, 1/9 -> 2/3, v/v) yielded the product. (1.1 g, 0.86 mmol, 98%)

$^{1}H$ NMR (400 MHz, $CDCl_3$) δ 7.96 – 7.14 (m, 45H, $H_{arom}$), 6.61 (d, *J* = 9.7 Hz, 1H, NH), 5.83 (dt, *J* = 10.3, 2.6 Hz, 1H, $H_{alkene}$), 5.68 (ddd, *J* = 10.3, 2.8, 1.6 Hz, 1H, $H_{alkene}$), 5.18 (d, *J* = 10.5 Hz, 2H, $CH_2$-Cbz), 4.97 – 4.88 (m, 2H, H-1′, $CH_2$-Bn), 4.81 – 4.53 (m, 7H, H-6, $CH_2$-Bn), 4.53 – 4.46 (m, 4H, H-6, $CH_2$-Bn, $CH_2$-Bn), 4.32 (td, *J* = 9.8, 3.4 Hz, 1H, H-2′), 4.19 (dt, *J* = 6.3, 2.1 Hz, 1H, H-2), 3.91 – 3.84 (m, 2H, H-6′, H-4), 3.80 (m, *J* = 8.6, 6.7, 3.0 Hz, 4H, H-4′/H-5′, H-6′, H-3, H-8), 3.70 – 3.64 (m, 2H, H-3′, H-4′/H-5′), 3.64 – 3.57 (m, 1H, H-8), 3.22 (d, *J* = 28.5 Hz, 2H, H-15), 2.65 (dt, *J* = 9.3, 3.5 Hz, 1H, H-5), 1.49 (t, *J* = 10.0 Hz, 4H, H-9, H-14), 1.44 (s, 3H, $CH_3$ acetyl), 1.20 (d, *J* = 18.5 Hz, 8H, H-10, H-11, H-12, H-13), 1.02 (s, 9H, TBDPS). $^{13}C$ NMR (101 MHz, $CDCl_3$) δ 166.3, (Acetyl), 138.9, 138.2, 138.0, 137.7, 136.0, 135.7, 133.9, 133.2, 133.2, 130.1, 129.8, 129.8, 129.7, 128.8, 128.8, 128.7, 128.6, 128.5, 128.4, 128.4, 128.2, 128.2, 128.1, 128.0, 127.8, 127.8, 127.7, 127.7, 126.9 ($C_{arom}$, $C_{alkene}$), 100.5 (C-1′), 81.8 (C-3′/C-3), 81.6 (C-3′/C-3), 79.16 (C-2′), 77.8 (C-4′/C-5′), 77.5 (C-4), 75.3, 75.0 (C-Bn), 73.7 (C-4′/C-5′), 73.4 (C-8), 71.6 (C-Bn), 67.3 (C-Cbz), 64.1 (C-6), 62.6 (C-6′), 53.4 (C-2), 50.7 (C-Bn), 47.4 (C-15), 46.5 (C-15), 44.2 (C-5′), 30.7, 29.9, 29.8, 29.6 ($CH_2$), 27.0 (TBDPS), 26.4 ($CH_2$), 22.9 ($CH_3$ acetyl), 19.5 (TBDPS, quaternary C). HRMS (ESI) m/z: $[M+Na]^+$ calculated for $C_{82}H_{94}N_2O_{12}SiNa$ 1349.6468, found 1349.6487.

### 2,3-di-*O*-benzyl-4-*O*-(2-*N*-acetyl-2-deoxy-3-*O*-benzyl-4-*O*-(8-*N*-benzyl-(benzyloxycarbonyl)-1-octyl)-6-*O*-tert-butyldiphenylsilyl-α-D-glucopyranosyl)-cyclophellitol alkene (35b)

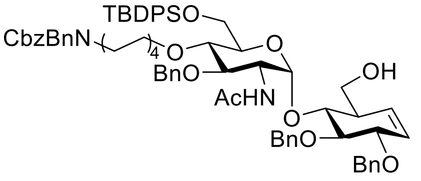


Benzoyl ester **35a** (1.1 g, 0.86 mmol) was dissolved in MeOH/DCM (21 ml, 0.04 M, 9.5/1, v/v), NaOMe (4.37 M in MeOH, 0.12 ml, 0.57 mmol) was added and the mixture was stirred for 21.5 hours. $NH_4Cl$ was added and the reaction was concentrated in vacuo. The residue was dissolved in in EtOAc, washed with water and brine, dried over $MgSO_4$, filtrated and concentrated in vacuo. Column chromatography (EtOAc/PE, 1/4 -> 2/3, v/v) yielded the product. (1.0 g, 0.83 mmol, 97%)

$^{1}H$ NMR (400 MHz, $CDCl_3$) δ 7.71 (m, 5H, $H_{arom}$), 7.45 – 7.11 (m, 35H, $H_{arom}$), 6.70 (d, *J* = 9.7 Hz, 1H, NH), 5.85 (dt, *J* = 10.3, 2.6 Hz, 1H, $H_{alkene}$), 5.63 (ddd, *J* = 10.3, 2.8, 1.6 Hz, 1H, $H_{alkene}$), 5.17 (d, *J* = 10.8 Hz, 2H, $CH_2$-Cbz), 4.93 (d, *J* = 11.2 Hz, 1H, $CH_2$-Bn), 4.90 (d, *J* = 3.5 Hz, 1H, H-1′), 4.71 (dd, *J* = 11.3, 5.7 Hz, 2H, $CH_2$-Bn), 4.68 – 4.55 (m, 2H, $CH_2$-Bn), 4.50 (dd, *J* = 10.4, 5.9 Hz, 3H, $CH_2$-Bn, $CH_2$-Bn), 4.29 (td, *J* = 9.8, 3.5 Hz, 1H, H-2′), 4.19 (m, 1H, H-2), 3.94 – 3.89 (m, 2H, H-6′, H-6), 3.87 – 3.79 (m, 4H, H-4′, H-5′, H-6′, H-8), 3.78 (d, *J* = 2.7 Hz, 1H, H-3), 3.63 (m, 2H, H-3′, H-6), 3.59 – 3.51 (m, 2H, H-4, H-8), 3.22 (dt, *J* = 29.4, 7.7 Hz, 2H, H-15), 2.38 (dq, *J* = 6.3, 3.1 Hz, 1H, H-5), 2.24 (s, 1H, OH), 1.55 – 1.47 (m, 4H, H-9, H-13), 1.46 (s, 3H, $CH_3$ acetyl), 1.20 (d, *J* = 19.2 Hz, 8H, H-10, H-11, H-12, H-13), 1.05 (s, 9H, TBDPS). $^{13}C$ NMR (101 MHz, $CDCl_3$) δ 170.4 (Acetyl), 138.7, 138.1, 138.0, 137.6, 137.0, 136.0, 135.7, 133.8, 133.2, 129.8, 129.8, 129.6, 128.7, 128.6, 128.5, 128.4, 128.3, 128.3, 128.1, 128.0, 128.0, 127.9, 127.8, 127.7, 127.5, 127.4, 127.3, 126.7 ($C_{arom}$, $C_{alkene}$), 100.2 (C-1′), 81.5 (C-3′, C-3), 79.1 (C-2), 78.0 (C-4′/C-5′), 77.8 (C-4), 75.0 (C-Bn), 74.9 (C-Bn), 73.7 (C-4′/C-5′), 73.2 (C-8), 71.5 (C-Bn), 67.2 (C-Cbz), 62.8 (C-6′), 62.4 (C-6), 53.2 (C-2′), 50.6 (C-Bn), 50.3 (C-Bn), 47.3 (C-15), 46.3 (C-5), 30.7, 30.5, 29.6, 29.4, 28.2, 27.8 ($CH_2$), 26.9 (TBDPS), 26.3 ($CH_2$), 22.8 ($CH_3$ acetyl), 19.9 (TBDPS, quaternary C). HRMS (ESI) m/z: $[M+Na]^+$ calculated for $C_{75}H_{90}N_2O_{11}SiNa$ 1245.6206, found 1245.6245.

### 2,3-di-*O*-benzyl-4-*O*-(2-*N*-acetyl-2-deoxy-3-*O*-benzyl-4-*O*-(8-*N*-benzyl-(benzyloxycarbonyl)-1-octyl)-6-*O*-tert-butyldiphenylsilyl-α-D-glucopyranosyl)-6-*O*-tert-butyloxycarbonyl-cyclophellitol alkene (35c)

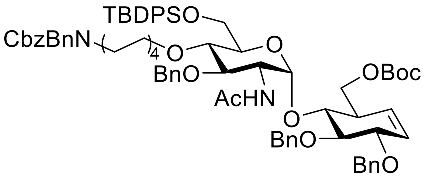


Alcohol **35b** (0.53 g, 0.43 mmol) was co-evaporated with toluene (3x) and dissolved in THF (3.2 ml, 0.14 M). $Boc_2O$ (0.38 g, 1.8 mmol) and DMAP (0.16 mM in THF, 0.32 ml, 0.05 mmol) were added. The reaction was stirred for 1 hour and quenched with $H_2O$. The reaction mixture was diluted with $Et_2O$ and washed with $NH_4Cl$ (aq. sat.), $NaHCO_3$ (aq. sat.) and brine,

dried over $MgSO_4$, filtrated and concentrated in vacuo. Column chromatography (EtOAc/PE, 1/9 -> 3/7, v/v) yielded the product. (0.47 g, 0.35 mmol, 82%)

$^1$H NMR (300 MHz, $CDCl_3$) δ 7.80 – 7.62 (m, 5H, $H_{arom}$), 7.47 – 7.13 (m, 35H, $H_{arom}$), 6.73 (d, *J* = 9.7 Hz, 1H, NH), 5.82 (dt, *J* = 10.3, 2.5 Hz, 1H, $H_{alkene}$), 5.62 (dt, *J* = 10.3, 1.9 Hz, 1H, $H_{alkene}$), 5.18 (d, *J* = 7.4 Hz, 2H, $CH_2$-Cbz), 4.94 (d, *J* = 11.1 Hz, 1H, $CH_2$-Bn), 4.86 (d, *J* = 3.5 Hz, 1H, H-1'), 4.78 (d, *J* = 11.3 Hz, 1H, $CH_2$-Bn), 4.72 – 4.52 (m, 4H, $CH_2$-Bn), 4.52 – 4.46 (m, 2H, $CH_2$-Bn), 4.41 – 4.25 (m, 3H, H-2', H-6), 4.23-4.20 (m, 1H, H-2), 3.96 – 3.76 (m, 5H, H-3, H-4, H-6', H-8), 3.76 – 3.56 (m, 4H, H-3', H-4', H-5', H-8), 3.28-3.16 (m, 2H, H-15), 2.52 (s, 1H, H-5), 1.56 – 1.45 (m, 4H, H-9, H-14), 1.43 (s, 3H, $CH_3$ acetyl), 1.42 (s, 9H, t-Butyl), 1.25 – 1.15 (m, 8H, H-1, H-11, H-12, H-13), 1.05 (s, 9H, TBDPS).
$^{13}$C NMR (75 MHz, $CDCl_3$) δ 170.4 (Acetyl), 153.5 (t-Butyl), 138.8, 138.1, 138.1, 137.9, 137.7, 136.0, 135.7, 133.9, 133.2, 129.7, 128.7, 128.7, 128.6, 128.6, 128.4, 128.3, 128.3, 128.1, 128.0, 127.9, 127.8, 127.7, 127.5, 127.4, 126.9 ($C_{arom}$, $C_{alkene}$), 100.5 (C-1'), 81.8 (C-3', C-3), 79.4 (C-2), 77.7 (C-4'/C-5'), 77.6 (C-3/C-4), 75.1 (C-Bn), 75.0 (C-Bn), 73.6 (C-4'/C-5'), 73.2 (C-8), 71.5 (C-Bn), 67.2 (C-Cbz), 66.2 (C-6), 62.5 (C-6'), 53.4 (C-2'), 50.6 (C-Bn), 47.4 (C-15), 46.4 (C-8), 44.0 (C-5), 30.6 (C-9/C-14), 29.7, 29.4 ($CH_2$), 27.8 (t-butyl), 26.9 (TBDPS), 26.9 , 26.3 ($CH_2$), 22.8 ($CH_3$ acetyl), 19.4 (t-butyl quaternary C). HRMS (ESI) m/z: $[M+H]^+$ calculated for $C_{80}H_{99}N_2O_{13}Si$ 1323.6911, found 1323.6910.

## 1-iodo-2,3-di-*O*-benzyl-4-*O*-(2-*N*-acetyl-2-deoxy-3-*O*-benzyl-4-*O*-(8-*N*-benzyl-(benzyloxycarbonyl)-1-octyl)-6-*O*-tert-butyldiphenylsilyl-α-D-glucopyranosyl)-6,7-*O*-carbonyl-cyclophellitol alkane (35d)

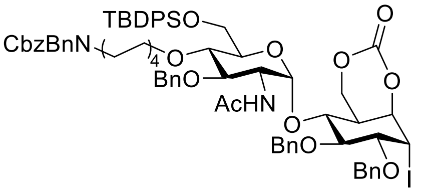


Alkene **35c** (0.47 g, 0.35 mmol) was dissolved in DCM/AcOH (1.8 ml, 0.2 M, 2/1, v/v). NIS (0.16 g, 0.71 mmol) was added and the reaction was stirred for 20 hours. Additional NIS (0.039 g, 0.18 mmol) was added and the reaction was stirred for 2 hours. The solution was diluted with $Et_2O$ and quenched with $Et_3N$. The organic layer was washed with $NH_4Cl$ (aq. sat.), $NaHCO_3$ (aq. sat.), $Na_2S_2O_3$ (aq. sat.) and brine, dried over $MgSO_4$, filtrated and concentrated in vacuo. Column chromatography (EtOAc/PE, 1/9 -> 2/3, v/v) yielded the product. (0.33 g, 0.24 mmol, 68%)

$^1$H NMR (400 MHz, $CDCl_3$) δ 7.74 – 7.59 (m, 5H, $H_{arom}$), 7.47 – 7.10 (m, 35H, $H_{arom}$), 6.37 (d, *J* = 9.7 Hz, 1H, NH), 5.17 (d, *J* = 11.1 Hz, 2H, $CH_2$-Cbz), 5.08 (d, *J* = 10.5 Hz, 1H, $CH_2$-Bn), 5.01 (d, *J* = 12.1 Hz, 1H, H-6), 4.87 – 4.79 (m, 3H, H-1', H-7, $CH_2$-Bn), 4.72 – 4.52 (m, 7H, H-1, $CH_2$-Bn), 4.52 – 4.47 (m, 2H, $CH_2$-Bn), 4.34 (td, *J* = 9.9, 3.4 Hz, 1H, H-2'), 4.04 (dd, *J* = 12.2, 2.7 Hz, 1H, H-1), 3.95 – 3.80 (m, 5H, H-6', H-4'/H-5', H-3, H-8), 3.75 (t, *J* = 9.9 Hz, 1H, H-4) 3.70 – 3.54 (m, 3H, H-3', H-4'/H-5',H-8), 3.29 – 3.15 (m, 2H, H-15), 3.12 (dd, *J* = 9.3, 4.0 Hz, 1H, H-2), 2.61 (dd, *J* = 10.4, 2.1 Hz, 1H, H-5), 1.56 – 1.48 (m, 4H, H-9, H14), 1.25 – 1.17 (m, 8H, H-10, H-11, H-12, H-13), 1.05 (s, 9H). $^{13}$C NMR (101 MHz, $CDCl_3$) δ 170.6 (Acetyl), 146.9 (Carbonate), 138.7, 138.2, 137.2, 136.9, 136.1, 136.0, 135.8, 135.7, 134.0, 133.3, 133.0, 130.1, 129.9, 129.7, 128.9, 128.8, 128.7, 128.7, 128.6, 128.6, 128.6, 128.5, 128.5, 128.4, 128.3, 128.1, 128.0, 128.0, 127.8, 127.8, 127.7, 127.4 ($C_{arom}$), 101.6 (C-1'), 82.3 (C-3), 81.8 (C-7), 81.5 (C-3'), 77.9 (C-4'/C-5'), 77.5 (C-4'), 76.8 (C-2), 76.4 (C-Bn), 75.1 (C-Bn), 74.3 (C-4'/C-5'), 73.5 (C-8), 72.4 (C-Bn), 68.5 (C-6), 67.3 (C-Cbz), 62.8 (C-6'), 53.3 (C-2'), 50.7 (C-Bn), 50.4 (C-Bn), 46.5 (C-15), 35.3 (C-5), 30.7 (C-7/14), 30.6 (C-1), 29.8, 29.6 ($CH_2$), 27.1 (TBDPS), 27.0 , 26.5 ($CH_2$), 22.6 (Acetyl), 19.6 (t-butyl quaternary C). HRMS (ESI) m/z: $[M+H]^+$ calculated for $C_{76}H_{90}N_2O_{13}SiI$ 1393.5251, found 1393.5247.

## 2,3-di-*O*-benzyl-4-*O*-(2-*N*-acetyl-2-deoxy-3-*O*-benzyl-4-*O*-(8-*N*-benzyl-(benzyloxycarbonyl)-1-octyl)6-*O*-tert-butyldiphenylsilyl-α-D-glucopyranosyl)-cyclophellitol (36)

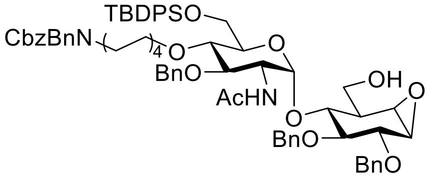


Iodocarbonate **35d** (0.28 g, 0.24 mmol) was dissolved in MeOH/DCM (3.4 ml, 0.7 M, 12:5, v/v). NaOMe (4.37 M in MeOH, 0.13 ml, 0.57 mmol) was added and the reaction was stirred for 15.5 hours. The reaction was quenched with $Et_3N \cdot HCl$ and concentrated in vacuo. The residue was dissolved in EtOAc,

washed with $H_2O$ and brine, dried over $MgSO_4$, filtrated and concentrated in vacuo. The product was used without further purification. (0.26 g, 0.21 mmol, 88%)

$^1$H NMR (400 MHz, $CDCl_3$) δ 7.80 – 7.63 (m, 5H, $H_{arom}$), 7.49 – 7.14 (m, 35H, $H_{arom}$), 6.80 (d, *J* = 9.7 Hz, 1H, NH), 5.17 (d, *J* = 11.1 Hz, 2H, $CH_2$-Cbz), 4.94 (d, *J* = 11.0 Hz, 1H, $CH_2$-Bn), 4.86 (d, *J* = 3.5 Hz, 1H, H-1'), 4.79 (d, *J* = 11.3 Hz, 1H, $CH_2$-Bn), 4.73 – 4.61 (m, 3H, $CH_2$-Bn), 4.52 – 4.44 (m, 3H, $CH_2$-Bn, $CH_2$-Bn), 4.28 (dt, *J* = 9.9, 6.6, 3.3 Hz, 1H, H-2'), 4.10 – 4.02 (m, 1H, H-6), 3.98 – 3.93 (m, 1H, H-6), 3.92 – 3.87 (m, 2H, H-6', H-2), 3.84 – 3.74 (m, 3H, H-4'/H-5', H-6', H-8), 3.64 (d, *J* = 9.9 Hz, 1H, H-4), 3.61 – 3.52 (m, 4H, H-3', H-4'/H-5', H-3, H-8), 3.36 – 3.30 (m, 1H, $H_{epoxide}$), 3.22 (m, 2H, H-15), 3.15 (d, *J* = 3.8 Hz, 1H, $H_{epoxide}$), 2.03 – 1.96 (m, 1H, H-5), 1.88 (s, 1H, OH), 1.55 – 1.45 (m, 4H, H-9, H-14), 1.43 (s, 3H, $CH_3$ acetyl), 1.25 – 1.16 (m, 8H, H-10, H-11, H-12, H-13), 1.05 (s, 9H, TBDPS). $^{13}$C NMR (101 MHz, $CDCl_3$) δ 170.3 (Acetyl), 138.7, 138.1, 137.6, 137.3, 136.1, 135.7, 133.9, 133.3, 129.8, 128.8, 128.7, 128.7, 128.6, 128.4, 128.3, 128.3, 128.2, 128.1, 128.0, 127.9, 127.8, 127.7, 127.6, 127.3, 127.1 ($C_{arom}$), 100.8 (C-1'), 83.2 (C-3'), 81.4 (C-3), 80.1 (C-2), 77.6 (C-4'/C-5'), 75.9 (C-4), 75.6 ($CH_2$-Bn), 74.8 ($CH_2$-Bn), 74.0 (C-4'/C-5'), 73.2 (C-8), 72.8 (C-Bn), 67.3 (C-Cbz), 62.7 (C-6'), 61.8 (C-6), 56.6 ($C_{epoxide}$), 53.2 (C-2'), 52.3 ($C_{epoxide}$), 50.6, 50.3 (C-Bn), 46.4 (C-15), 44.6 (C-5), 30.6 (C-9/14), 29.9, 29.7, 29.5, 27.9 ($CH_2$), 27.0 (TBDPS), 26.4 ($CH_2$), 22.8 (Acetyl), 19.5 (t-butyl quaternary C). HRMS (ESI) m/z: $[M+Na]^+$ calculated for $C_{75}H_{90}N_2O_{12}SiNa$ 1261.6155, found 1261.6184.

## 2,3-di-*O*-benzyl-4-*O*-(2-*N*-acetyl-2-deoxy-3-*O*-benzyl-4-*O*-(8-*N*-benzyl-(benzyloxycarbonyl)-1-octyl)6-*O*-tert-butyldiphenylsilyl-α-D-glucopyranosyl)-glucurono-cyclophellitol (37a)

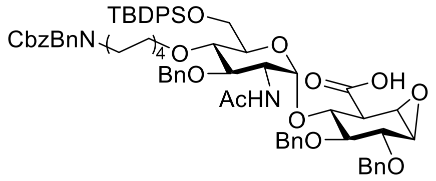


Alcohol **36** (0.25 g, 0.21 mmol) was dissolved in *t*-BuOH/DCM/$H_2O$ (7.7 ml, 26 mM, 5/4/1, v/v). TEMPO (7 mg, 0.04 mmol) and BAIB (0.161 g, 0.50 mmol) were added and the solution was stirred for 24 hours. The reaction was diluted with DCM and $H_2O$ and quenched with $Na_2S_2O_3$ (aq. sat.). The water layer was acidified with AcOH and extracted with DCM (4x). The combined organic layers were dried over $MgSO_4$, filtrated and concentrated in vacuo. Column chromatography (EtOAc/PE, 1/9 -> 1/1, v/v, 1% AcOH) yielded the product. (0.17 g, 0.13 mmol, 67%)

$^1$H NMR (400 MHz, $CDCl_3$) δ 7.83 – 7.58 (m, 5H, $H_{arom}$), 7.47 – 7.14 (m, 35H, $H_{arom}$), 6.07 (d, *J* = 9.6 Hz, 1H, NH), 5.17 (d, *J* = 13.4 Hz, 2H, $CH_2$-Cbz), 4.97 – 4.90 (m, 2H, H-1', $CH_2$-Bn), 4.83 – 4.54 (m, 5H, $CH_2$-Bn), 4.49 (d, *J* = 7.6 Hz, 2H, $CH_2$-Bn), 4.19 (td, *J* = 10.0, 3.6 Hz, 1H, H-2'), 3.97 – 3.91 (m, 2H, H-6', H-4), 3.88 (d, *J* = 8.2 Hz, 1H, H-2), 3.85 – 3.74 (m, 2H, H-6', H-8), 3.74 – 3.62 (m, 3H, H-4', H-5', H-8), 3.58 (t, *J* = 10.4, 8.4 Hz, 1H, H-3'), 3.47 (t, *J* = 10.1, 8.1 Hz, 1H, H-3), 3.32 (t, *J* = 2.9 Hz, 1H, $H_{epoxide}$), 3.22 (m, 2H, H-15), 3.17 (d, *J* = 3.7 Hz, 1H, $H_{epoxide}$), 2.80 (dd, *J* = 8.8, 2.2 Hz, 1H, H-5), 1.54 – 1.47 (m, 4H, H-9, H-14), 1.28 (s, 3H, Acetyl), 1.24 – 1.19 (m, 8H, H-10, H-11, H-12, H-13), 1.03 (s, 9H, TBDPS). $^{13}$C NMR (101 MHz, $CDCl_3$) δ 170.7 (Acetyl), 157.0 (COOH), 138.8, 138.1, 137.7, 137.2, 137.0, 136.4, 136.1, 135.9, 135.8, 133.9, 133.7, 129.9, 129.8, 128.8, 128.7, 128.6, 128.5, 128.4, 128.3, 128.2, 128.1, 128.0, 127.9, 127.9, 127.8, 127.7, 127.5, 127.3, 127.0 ($C_{arom}$), 100.1 (C-1'), 81.8 (C-3'/C-3), 81.6 (C-3'/C-3), 79.2 (C-2), 77.6 (C-4'/C-5'), 75.5 (C-Bn), 75.5 (C-4), 75.3 (C-Bn), 73.3 (C-8), 73.3 (C-4'/C-5'), 72.9 (C-Bn), 67.3 (C-Cbz), 62.3 (C-6'), 54.3 ($C_{epoxide}$), 53.7 ($C_{epoxide}$), 53.3 (C-2'), 50.6, 50.3 (C-Bn), 48.9 (C-5), 46.4 (C-15), 30.7 (C9/14), 29.7, 29.5, 28.3 ($CH_2$), 27.1 (TBDPS), 26.9, 26.4 ($CH_2$), 22.6 (Acetyl), 19.4 (t-butyl quaternary C). HRMS (ESI) m/z: $[M+Na]^+$ calculated for $C_{75}H_{88}N_2O_{13}SiNa$ 1275.5948, found 1275.5986.

## 2,3-di-*O*-benzyl-4-*O*-(2-*N*-acetyl-2-deoxy-3-*O*-benzyl-4-*O*-(8-*N*-benzyl-(benzyloxycarbonyl)-1-octyl)-α-D-glucopyranosyl)-glucurono-cyclophellitol (37b)

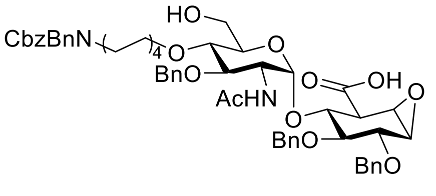


Silyl protected **37a** (0.60 g, 0.48 mmol) was dissolved in THF (7.2 ml, 0.05 M). $Et_3N{\cdot}3HF$ (0.29 ml, 1.8 mmol) was added and the reaction was stirred for 42 hours. The reaction mixture was diluted with DCM and water and the layers were separated. The water layer was extracted with DCM (4x) and the combined organic layers were dried over $MgSO_4$, filtrated and

concentrated in vacuo. Column chromatography (EtOAc/PE, 2/3 -> 1/0, v/v, 1 % AcOH) yielded the product. (0.49 g, 0.48 mmol, quant.)

$^1$H NMR (400 MHz, $CDCl_3$) δ 7.42 – 7.11 (m, 25H, $H_{arom}$), 6.27 (d, *J* = 9.6 Hz, 1H, NH), 5.17 (d, *J* = 11.4 Hz, 2H, $CH_2$-Cbz), 4.96 (d, *J* = 10.8 Hz, 1H, $CH_2$-Bn), 4.85 (d, *J* = 3.7 Hz, 1H, H-1'), 4.83 – 4.52 (m, 5H, $CH_2$-Bn), 4.48 (d, *J* = 9.3 Hz, 2H, $CH_2$-Bn), 4.22 (td, *J* = 10.0, 3.6 Hz, 1H, H-2'), 3.99 – 3.95 (m, 1H, H-4), 3.94 – 3.88 (m, 2H, H-5', H-6'), 3.79 – 3.66 (m, 3H, H-6', H-2, H-8), 3.60 – 3.44 (m, 4H, H-3', H-4', H-3, H-8), 3.43 – 3.40 (m, 1H, $H_{epoxide}$), 3.29 – 3.13 (m, 3H, $H_{epoxide}$, H-15), 2.89 (dd, *J* = 9.0, 2.1 Hz, 1H, H-5), 1.49 (m, 4H, H-9, H-14), 1.20 (d, *J* = 19.1 Hz, 11H, H-10, H-11, H-12, H-13, Acetyl). $^{13}$C NMR (101 MHz, $CDCl_3$) δ 173.0 (Acetyl), 157.0 (COOH), 138.8, 138.1, 138.0, 137.8, 137.3, 137.1, 129.2, 128.9, 128.8, 128.7, 128.6, 128.5, 128.4, 128.4, 128.4, 128.3, 128.1, 128.0, 127.6, 127.6, 127.4, 125.5 ($C_{arom}$), 100.4 (C-1'), 81.7 (C-3'/C-3) 81.6 (C-3'/C-3), 79.4 (C-5), 78.0 (C-4'), 76.9 (C-4), 75.8 (C-Bn), 75.1 (C-Bn), 73.5 (C-2), 73.3 (C-8), 72.9 (C-Bn), 67.3 (C-Cbz), 61.2 (C-6'), 54.6 ($C_{epoxide}$), 53.7 ($C_{epoxide}$), 53.6 (C-2'), 50.6, 50.3 (C-Bn), 49.7 (C-5), 46.4 (C-15), 30.7, 29.7, 29.5, 27.9, 26.9, 26.3 ($CH_2$), 22.7 (Acetyl). HRMS (ESI) m/z: $[M+H]^+$ calc for $C_{59}H_{71}N_2O_{13}$ 1015.4951, found 1015.4954.

## 4-*O*-(2-*N*-acetyl-2-deoxy-4-*O*-(8-amino-1-octyl)-α-D-glucopyranosyl)-glucurono-cyclophellitol (38)

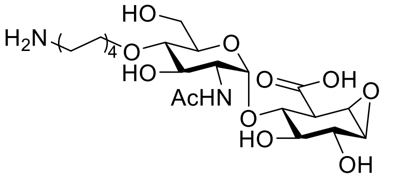


Benzyl protected **37b** (0.10 g, 0.10 mmol) was dissolved in dioxane/water (10 ml, 0.01 M, 8/5) and flushed with $N_2$ for 5 min. 10% Pd/C (0.21 g, 0.2 mmol) was added and the suspension was flushed with $N_2$ for another 5 min. The $N_2$ balloon was replaced with an $H_2$ balloon and the solution was flushed with $H_2$ for 10 min and stirred at rt for 7 hours and 40 minutes. The $H_2$ balloon was replaced by an $N_2$ balloon, the reaction mixture was flushed for 5 min, filtrated over celite and concentrated under reduced pressure. Size exclusion chromatography over HW-40 eluting with $H_2O$ 1% AcOH yielded a broad peak that was collected in three fractions. Based on NMR the pure product fraction was selected. (0.021 g, 0.041 mmol, 41%)

$^1$H NMR (500 MHz, MeOD) δ 5.11 (d, *J* = 3.8 Hz, 1H, H-1'), 3.86 (m, 4H, H-2', H-6', H-4, H-8), 3.77 (ddd, *J* = 10.0, 5.4, 2.1 Hz, 1H, H-5'), 3.74 – 3.66 (m, 2H, H-3', H-2), 3.62 (dd, *J* = 11.6, 5.3 Hz, 1H, H-6'), 3.53 (m, H-8), 3.42 (dd, *J* = 10.0, 8.2 Hz, 1H, H-3), 3.38 (t, *J* = 2.5, 1H, $H_{epoxide}$), 3.10 (dd, *J* = 10.0, 8.8 Hz, 1H, H-4'), 3.01 (d, *J* = 3.6 Hz, 1H, $H_{epoxide}$), 2.90 (t, *J* = 7.7 Hz, 2H, H-15), 2.74 (dd, *J* = 9.2, 2.1 Hz, 1H, H-5), 1.99 (s, 3H, acetyl), 1.69 – 1.62 (m, 2H, H-14), 1.61 – 1.50 (m, 2H, H-9), 1.39 (q, *J* = 5.6, 3.6 Hz, 8H, H-10, H-11, H-12, H-13). $^{13}$C NMR (126 MHz, MeOD) δ 174.0 (Acetyl), 99.8 (C-1'), 80.2 (C-4'), 78.2 (C-4), 77.8 (C-3), 73.9 (C-3'/C-2), 73.7 (C-8), 73.3 (C-5'), 73.2 (C-3'/C-2), 62.7 (C-6'), 57.0 (C-7), 56.6 (C-1), 56.1 (C-2'), 52.9 (C-5), 40.8 (C-15), 31.1 (C-9), 29.9, 29.9 ($CH_2$), 28.7 (C-14), 27.2, 27.2 ($CH_2$), 22.8 (Acetyl). HRMS (ESI) m/z: $[M+H]^+$ calc for $C_{23}H_{41}N_2O_{11}$ 521.2705, found 521.2703.

## 4-*O*-(2-*N*-acetyl-2-deoxy-4-*O*-(8-amino-Cy5-1-octyl)-α-D-glucopyranosyl)-glucurono-cyclophellitol (9)

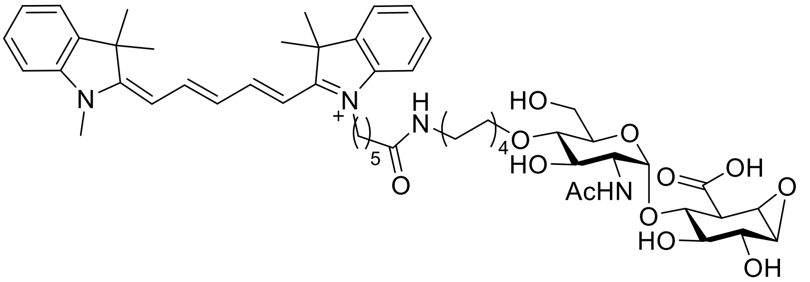


Cy5 carboxylic acid (11.6 mg, 22.3 µmol) was dissolved in DMF (0.25 ml). DIPEA (12 µl, 70 µmol), and pentafluorophenyl trifluoroacetate (3.8 µl, 22.3 µmol) were added and the mixture was stirred for one hour. LC-MS indicated the presence of starting material so more DIPEA (6 µl, 35 µmol) and pentafluorophenyl trifluoroacetate (3.8 µl, 22.3 µmol) were added. After stirring for 30 minutes water (2 µl) and DMF (0.25 ml) were added and the solution was added to amine **38** (12.2 mg, 23µmol).

The reaction was stirred overnight and the product was purified on semi-preparative HPLC eluting with a linear gradient of solution A (MeCN) in solution B (50mM AcOH in $H_2O$). The fractions were concentrated under reduced pressure, coevaporated with water, diluted with water and lyophilized to yield the product as a blue solid. (2,88 mg, 2,82 µmol, 13%)

$^1H$ NMR (850 MHz, $CD_3CN$) δ 8.02 (t, *J* = 13.1 Hz, 2H), 7.47 – 7.42 (m, 2H), 7.40 – 7.34 (m, 2H), 7.26 – 7.19 (m, 4H), 6.50 (t, *J* = 12.4 Hz, 1H), 6.16 (t, *J* = 13.1 Hz, 2H), 5.01 (d, *J* = 3.9 Hz, 1H), 3.96 (t, *J* = 7.5 Hz, 2H), 3.74 – 3.65 (m, 7H), 3.64 – 3.58 (m, 2H), 3.53 (dd, *J* = 12.0, 5.2 Hz, 1H), 3.49 (s, 5H), 3.38 (dd, *J* = 10.0, 8.4 Hz, 1H), 3.35 (dd, *J* = 3.8, 2.1 Hz, 1H), 3.10 (t, *J* = 9.6 Hz, 1H), 3.06 – 2.99 (m, 3H), 2.69 (dd, *J* = 9.3, 2.1 Hz, 1H), 2.08 (t, *J* = 7.3 Hz, 3H), 1.90 (s, 3H), 1.74 – 1.69 (m, 2H), 1.62 (s, 12H), 1.58 – 1.52 (m, 2H), 1.50 – 1.42 (m, 2H), 1.37 – 1.30 (m, 4H), 1.20 (s, 8H). $^{13}C$ NMR (214 MHz, $CD_3CN$) δ 175.5, 174.8, 174.4, 174.2, 154.6, 143.9, 143.2, 142.2, 142.1, 129.6, 126.0, 125.4, 123.2, 123.1, 112.0, 111.8, 103.9, 103.8, 98.3, 98.3, 79.5, 76.8, 76.7, 74.1, 72.5, 72.2, 72.1, 61.6, 57.1, 56.3, 56.3, 55.1, 51.7, 50.0, 50.0, 44.7, 40.0, 36.5, 31.8, 30.5, 29.9, 29.7, 27.7, 27.6, 27.5, 27.3, 26.7, 26.3, 26.1, 23.0. HRMS (ESI) m/z: $[M]^+$ calculated for $C_{55}H_{77}N_4O_{12}$ 985.5523, found 985.5533.

# 2-azido-2-deoxy-3,4-di-*O*-benzyl-6-*O*-tert-butyldiphenylsilyl-D-glucopyranoside (13b)

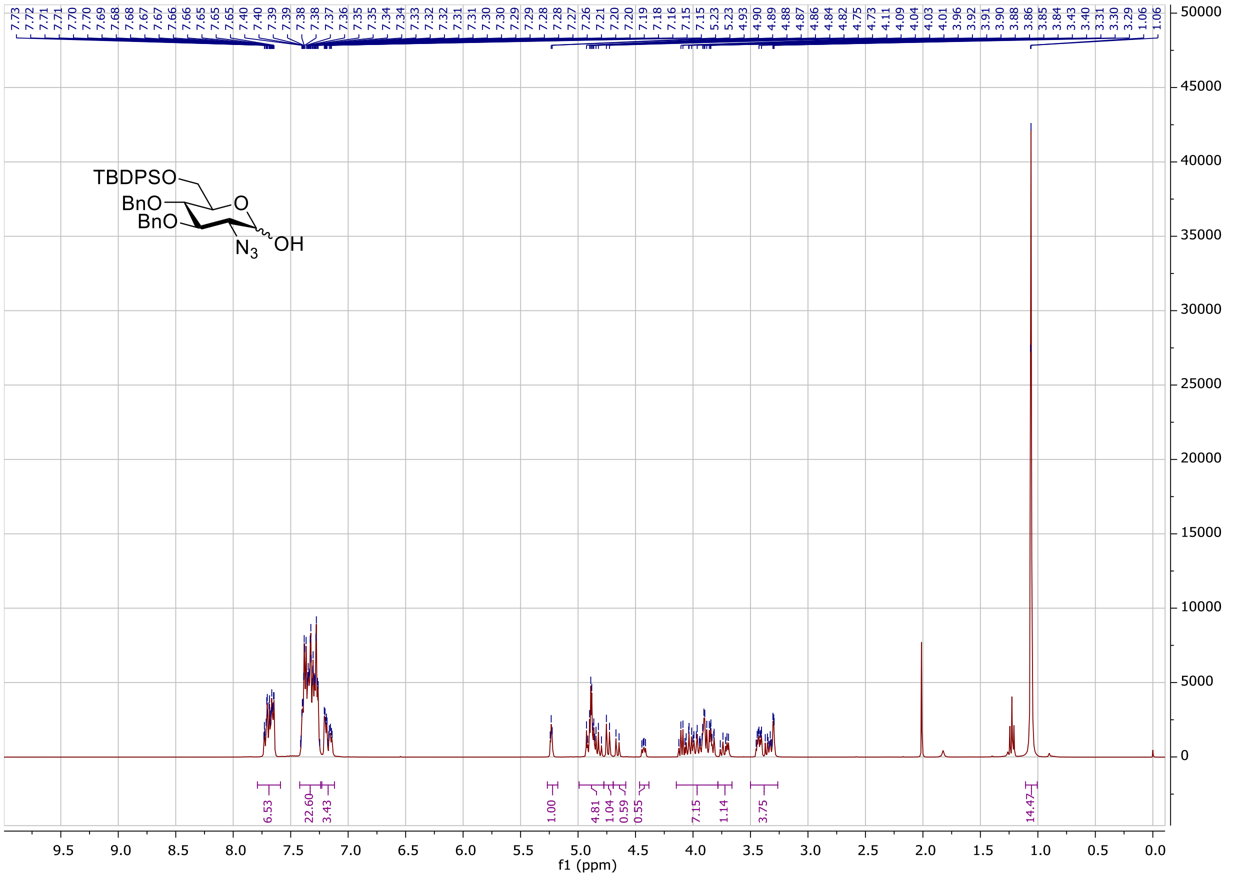


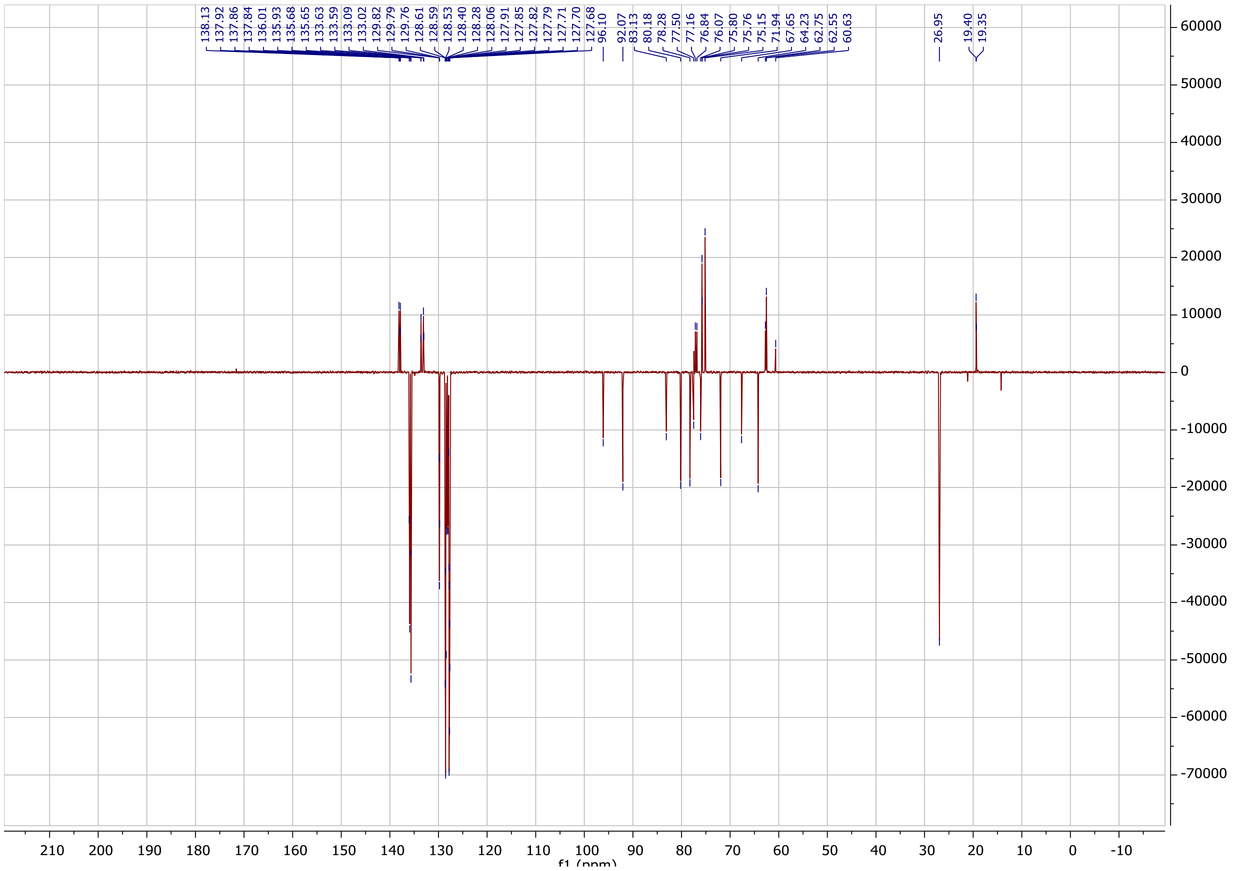

**2-azido-2-deoxy-3,4-di-*O*-benzyl-6-*O*-tert-butyldiphenylsilyl-D-glucopyranosyl N-phenyltrifluoroacetimidate (13c)**

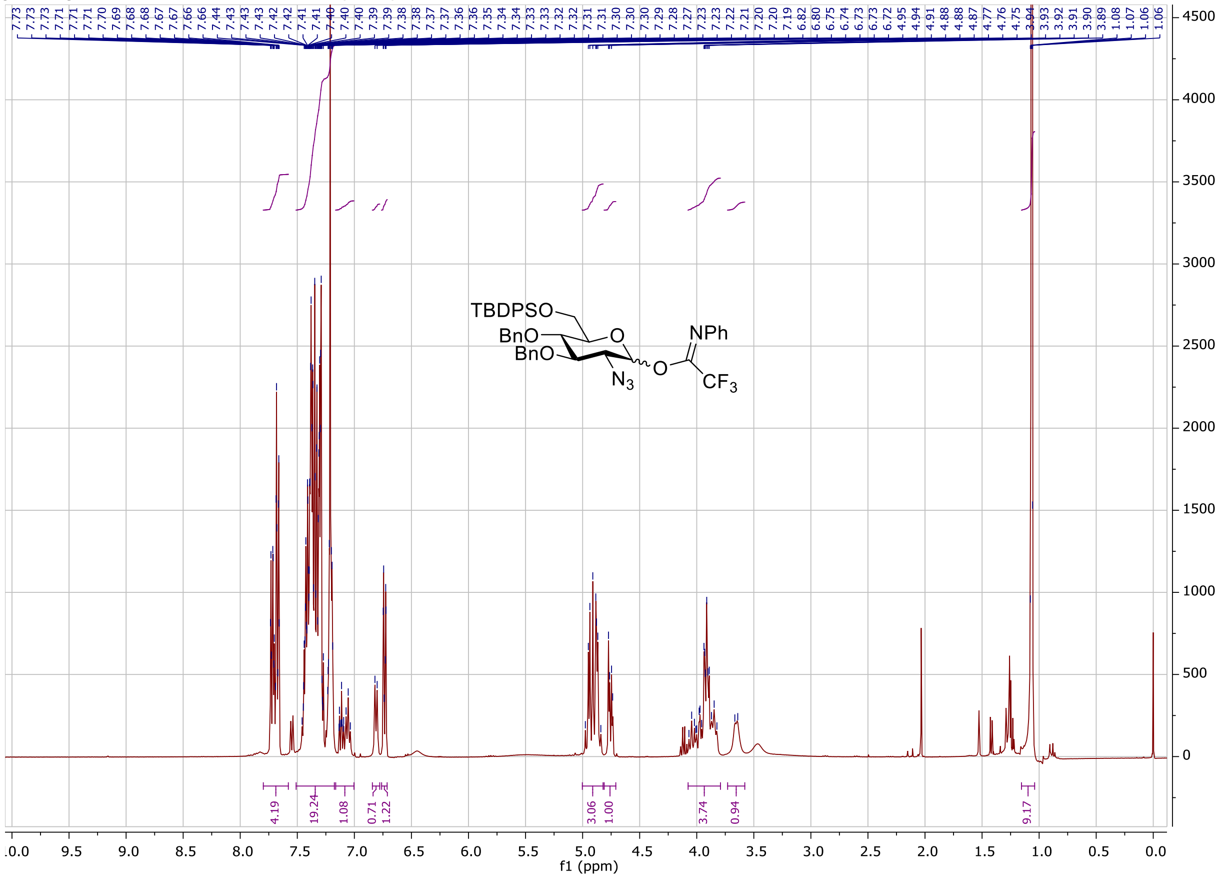

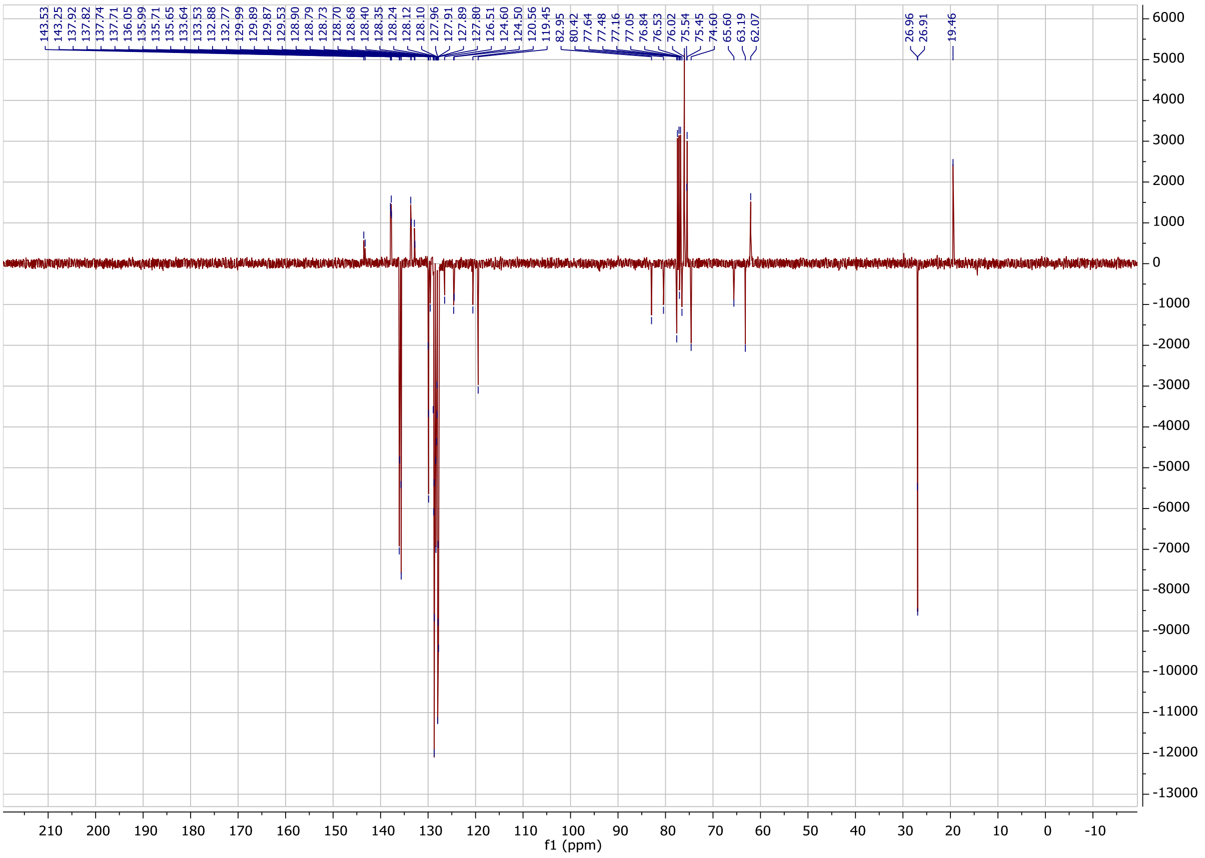

## 2,3-di-*O*-benzyl-6-*O*-benzoyl-cyclophellitol alkene (12b)

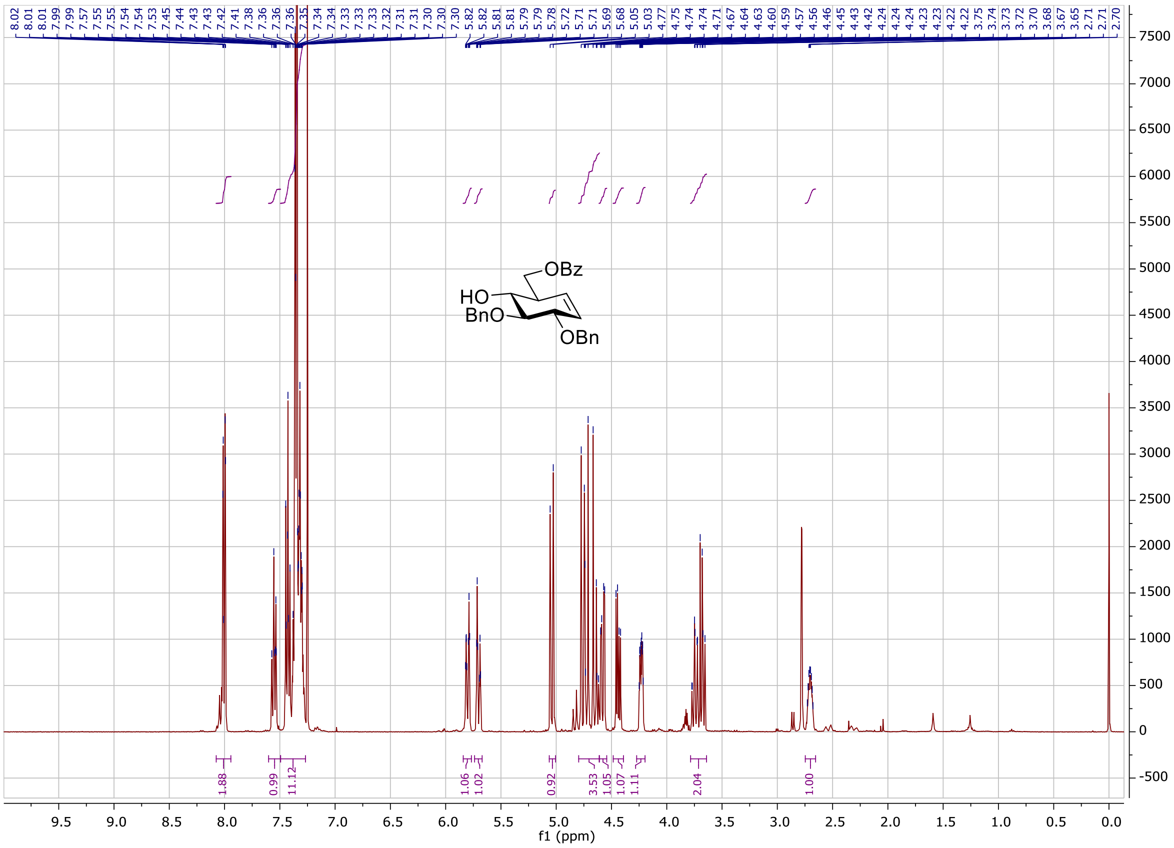


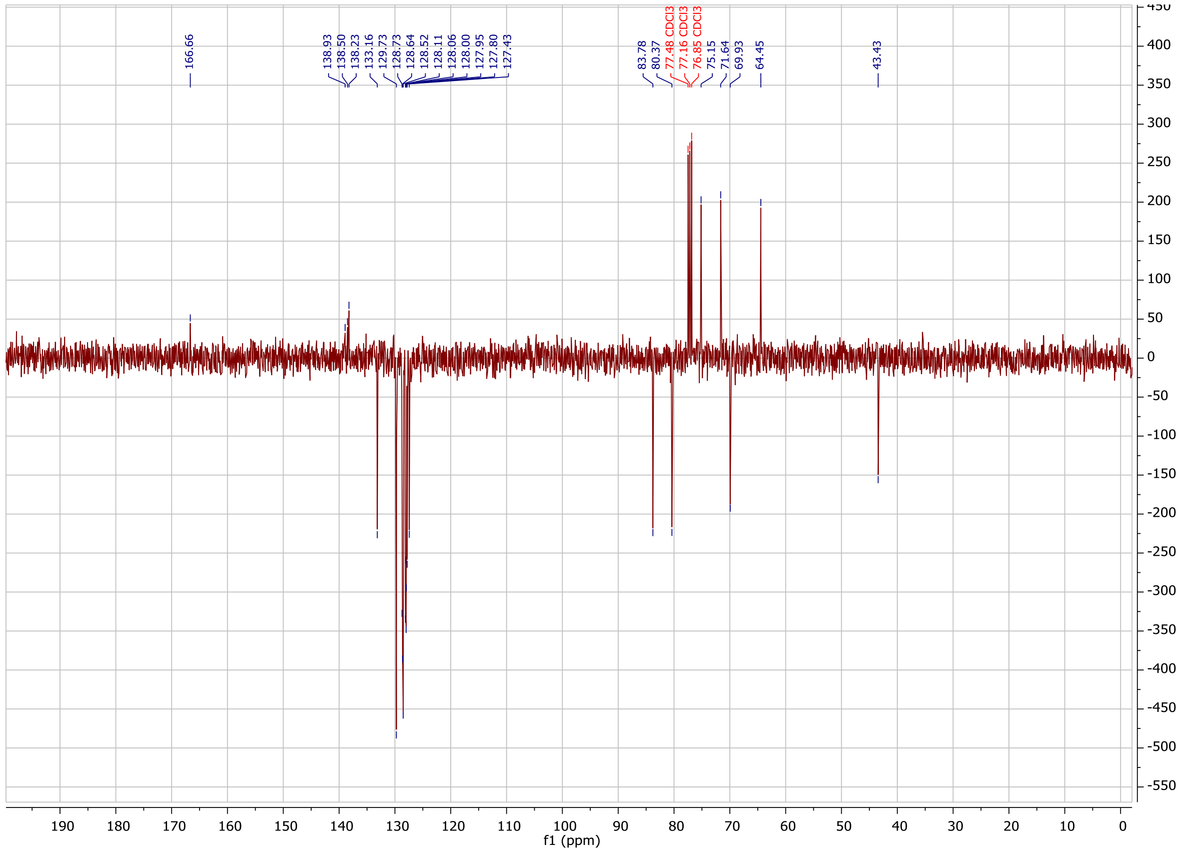

**2,3-di-*O*-benzyl-4-*O*-(2-azido-2-deoxy-3,4-di-*O*-benzyl-6-*O*-tert-butyldiphenylsilyl-α-D-glucopyranosyl)-6-*O*-benzoyl-cyclophellitol alkene (14a)**

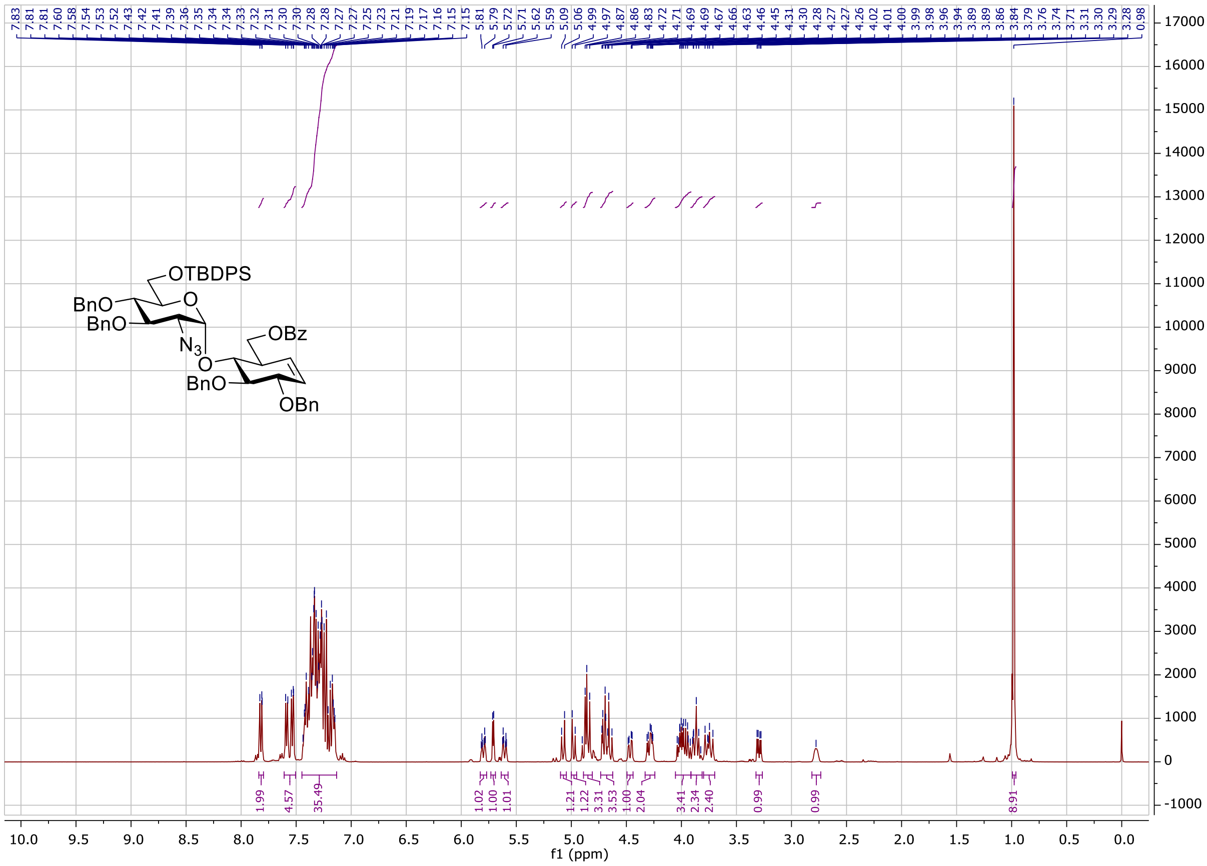

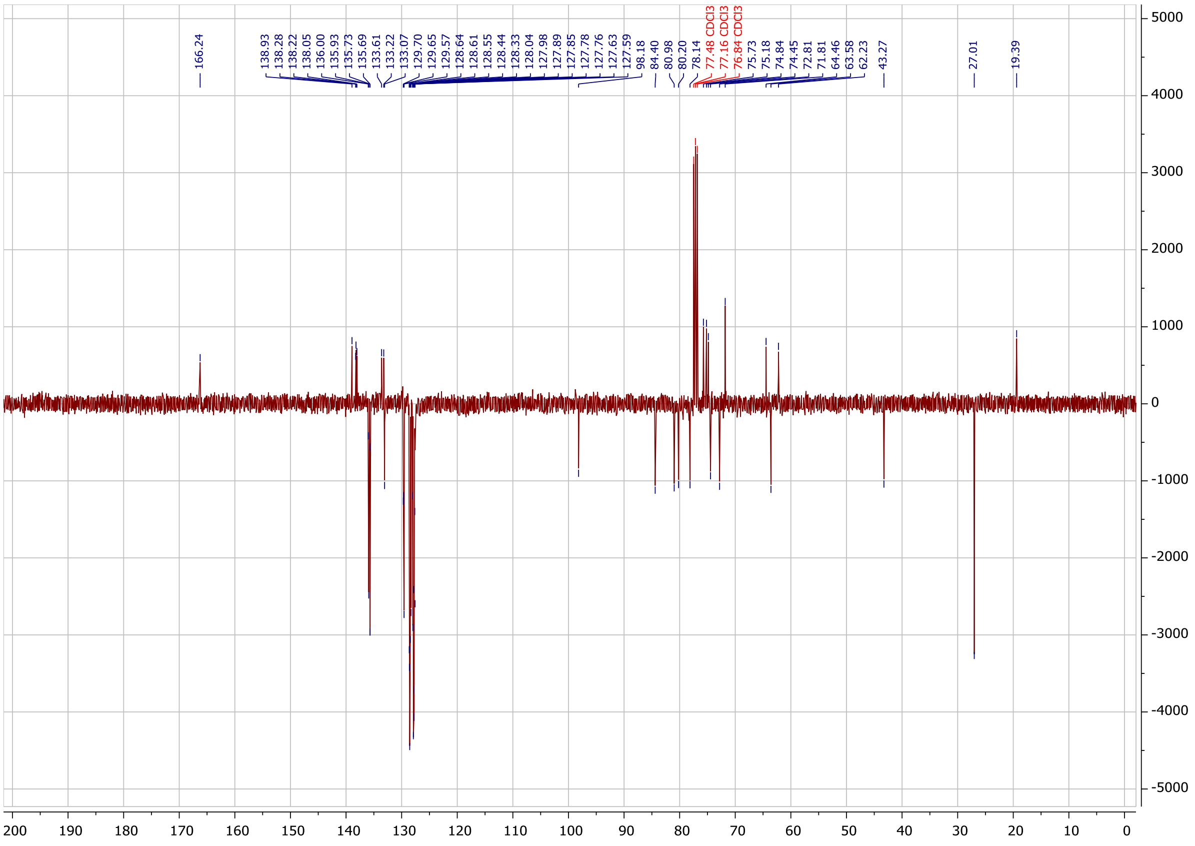

**2,3-di-*O*-benzyl-4-*O*-(2-*N*-acetyl-2-deoxy-3,4-di-*O*-benzyl-6-*O*-tert-butyldiphenylsilyl-α-D-glucopyranosyl)-6-*O*-benzoyl-cyclophellitol alkene (14b)**

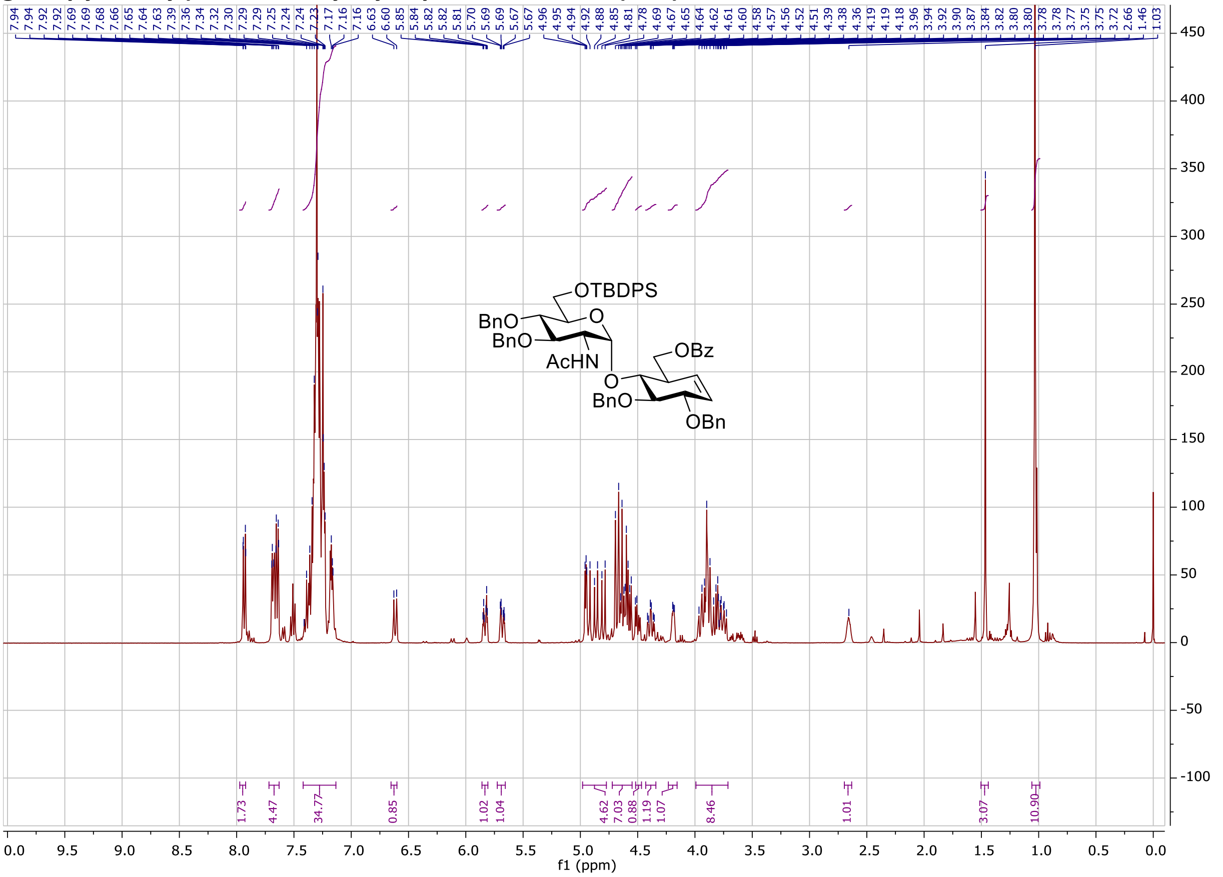


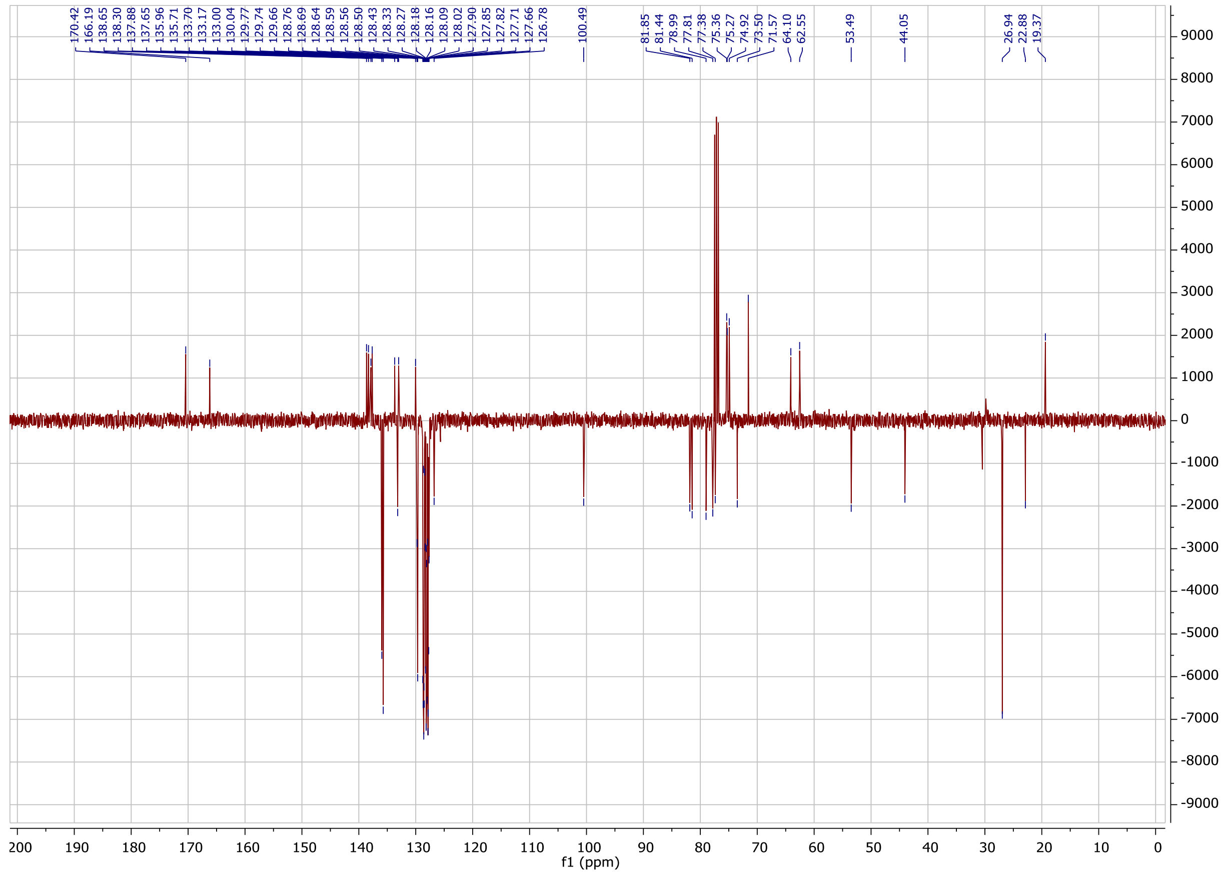

**2,3-di-*O*-benzyl-4-*O*-(2-*N*-acetyl-2-deoxy-3,4-di-*O*-benzyl-6-*O*-tert-butyldiphenylsilyl-α-D-glucopyranosyl)-cyclophellitol alkene (14c)**

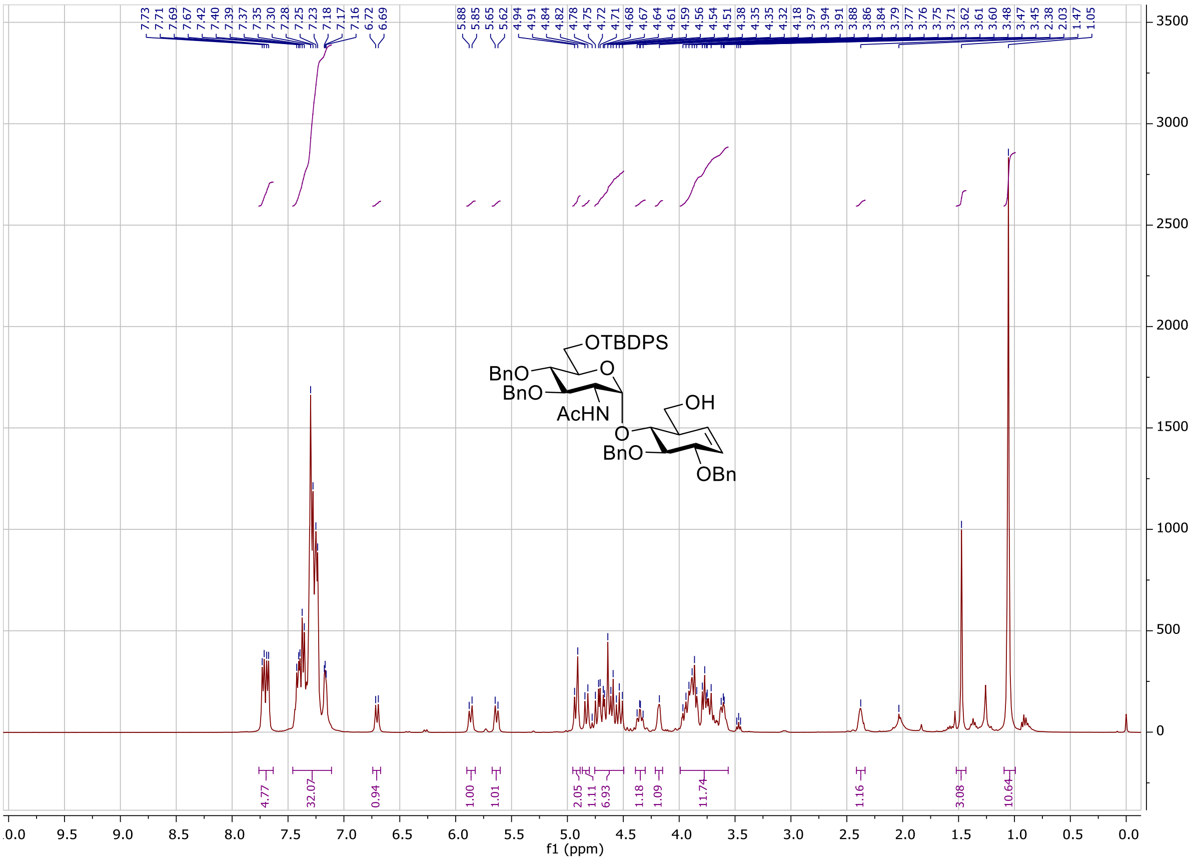


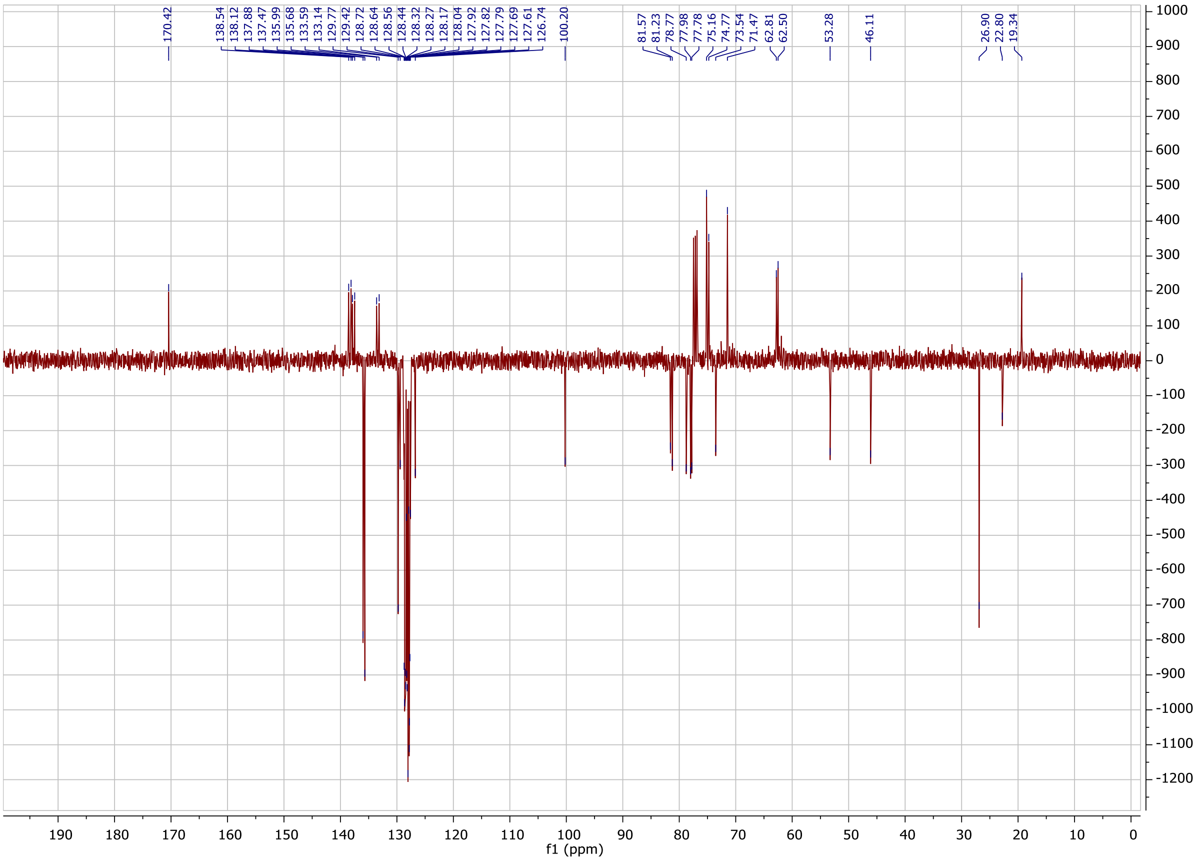

**2,3-di-*O*-benzyl-4-*O*-(2-*N*-acetyl-2-deoxy-3,4-di-*O*-benzyl-6-*O*-tert-butyldiphenylsilyl-α-D-glucopyranosyl)-6-*O*-tert-butyloxycarbonyl-cyclophellitol alkene (14d)**

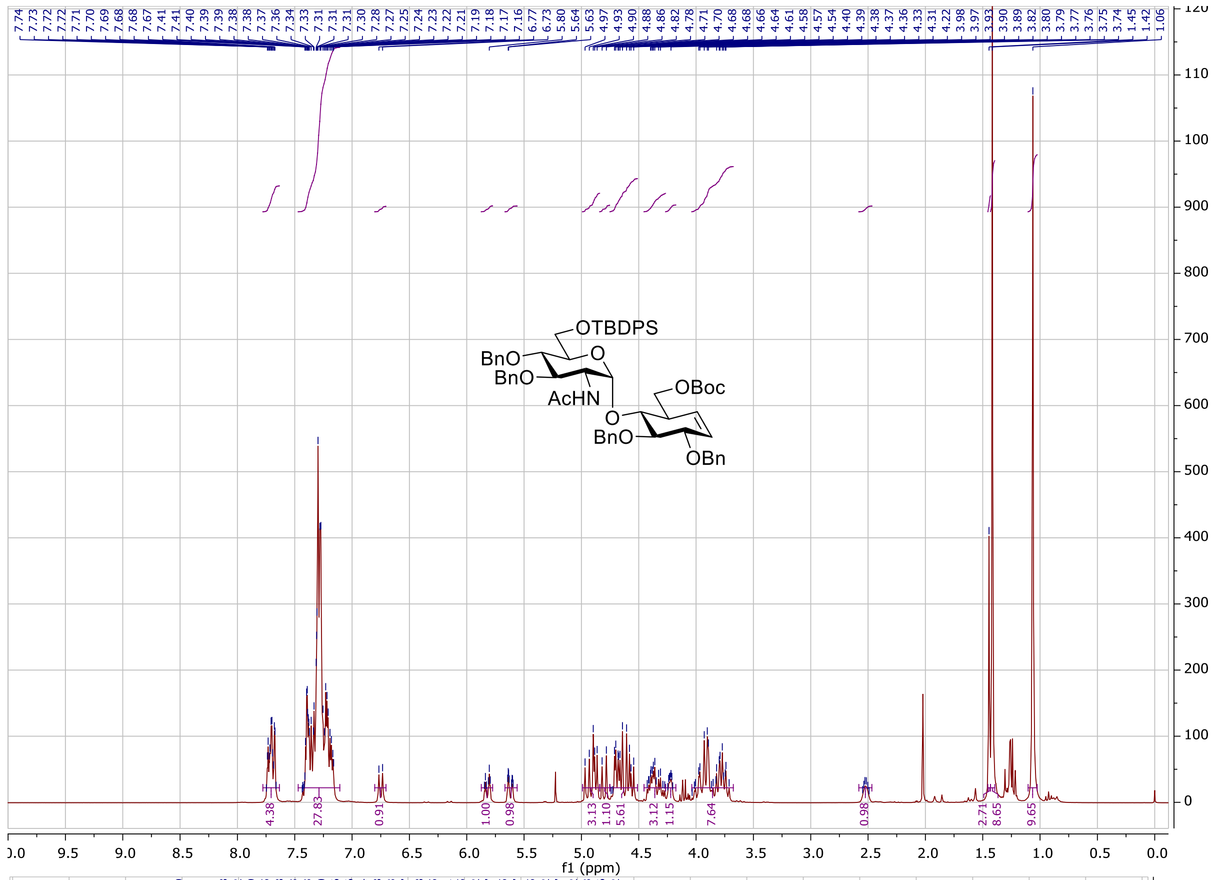

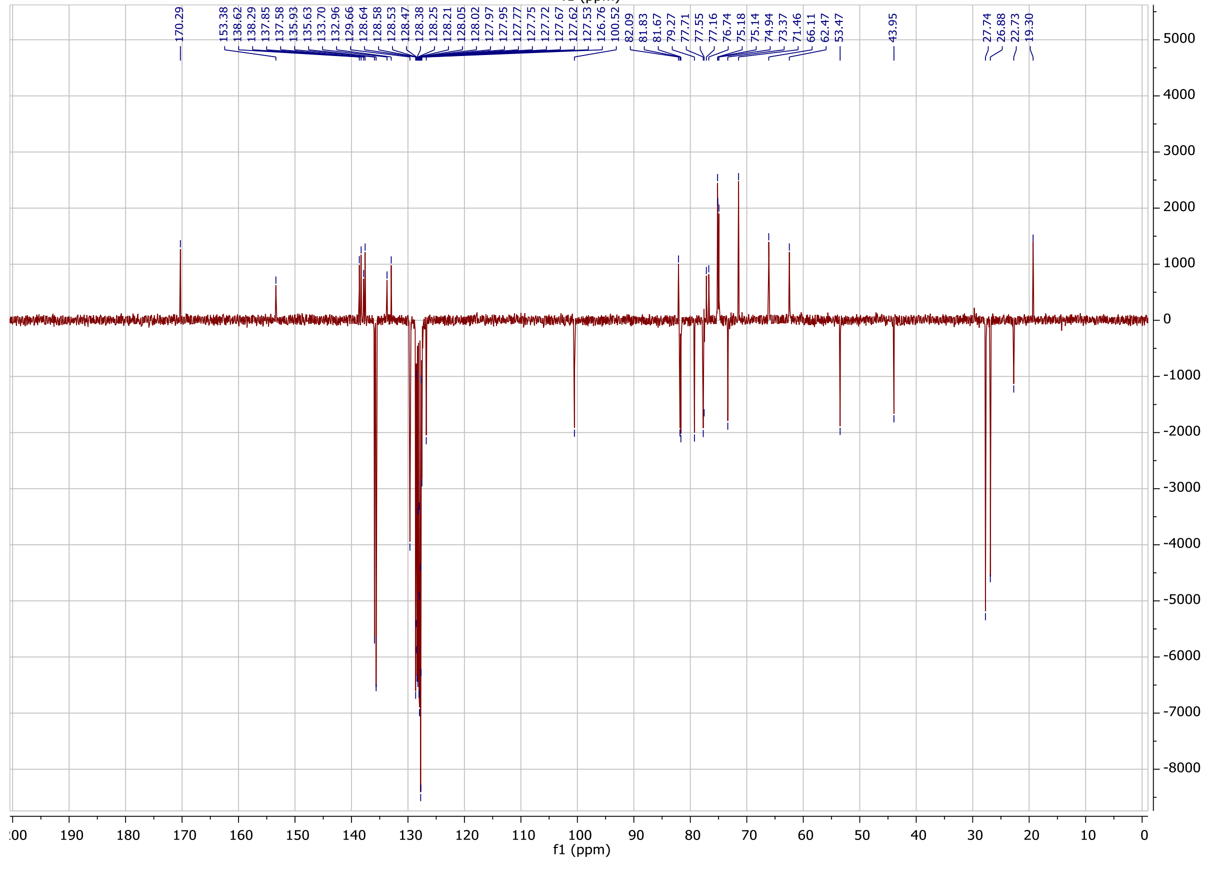

**1-iodo-2,3-di-*O*-benzyl-4-*O*-(2-*N*-acetyl-2-deoxy-3,4-di-*O*-benzyl-6-*O*-tert-butyldiphenylsilyl-α-D-glucopyranosyl)-6,7-*O*-carbonyl-cyclophellitol alkane (14e)**

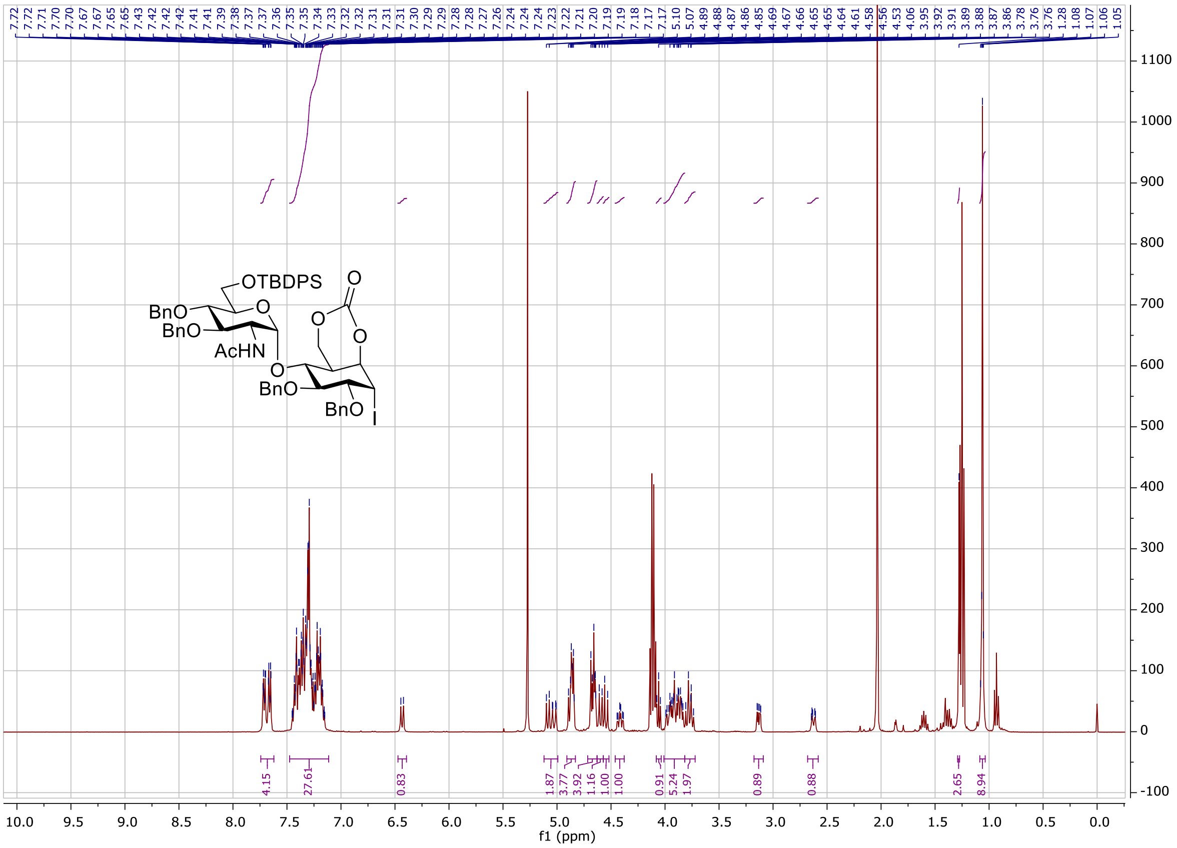


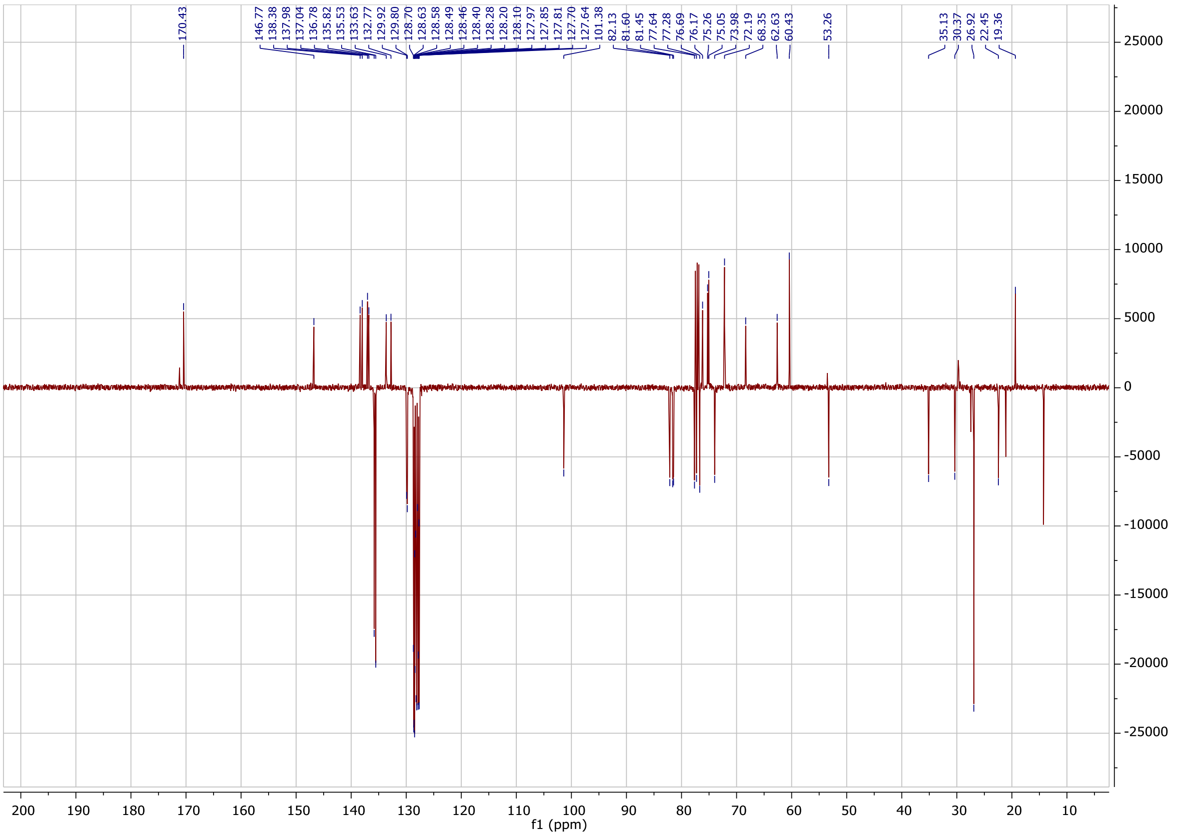

**2,3-di-*O*-benzyl-4-*O*-(2-*N*-acetyl-2-deoxy-3,4-di-*O*-benzyl-6-*O*-tert-butyldiphenylsilyl-α-D-glucopyranosyl)-cyclophellitol (15a)**

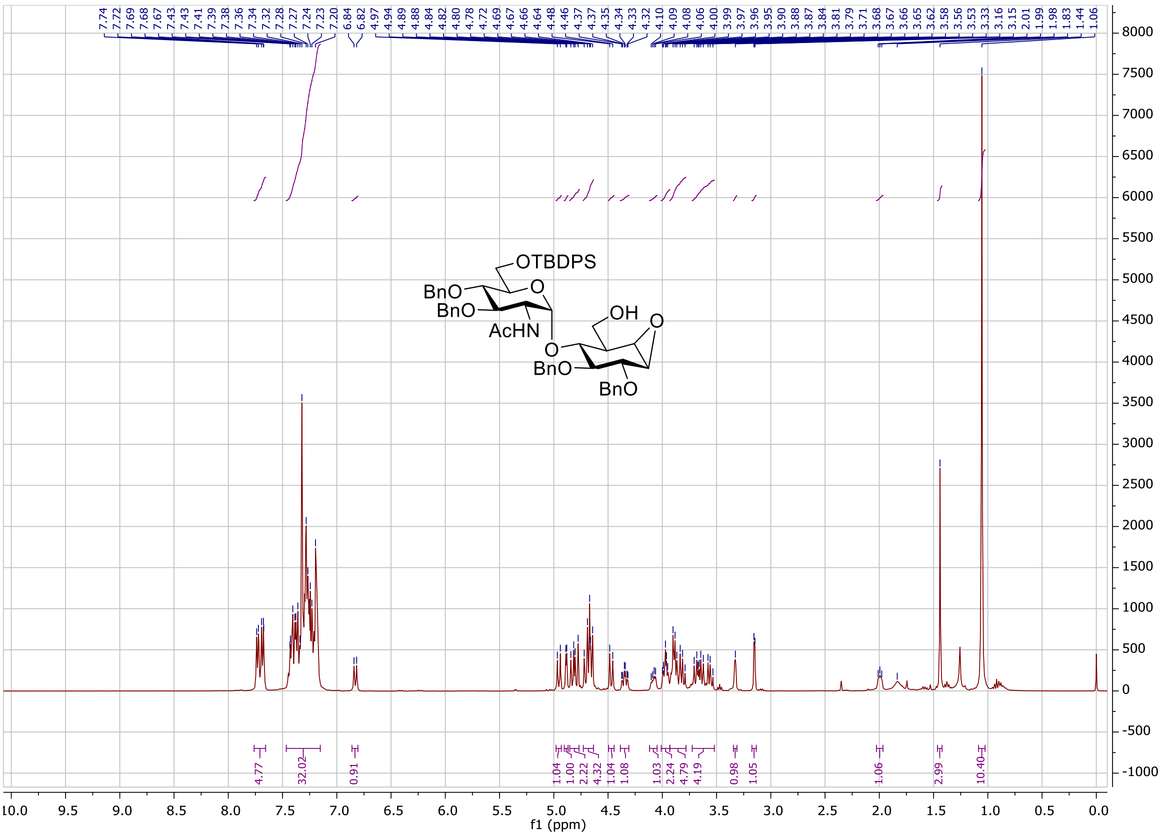


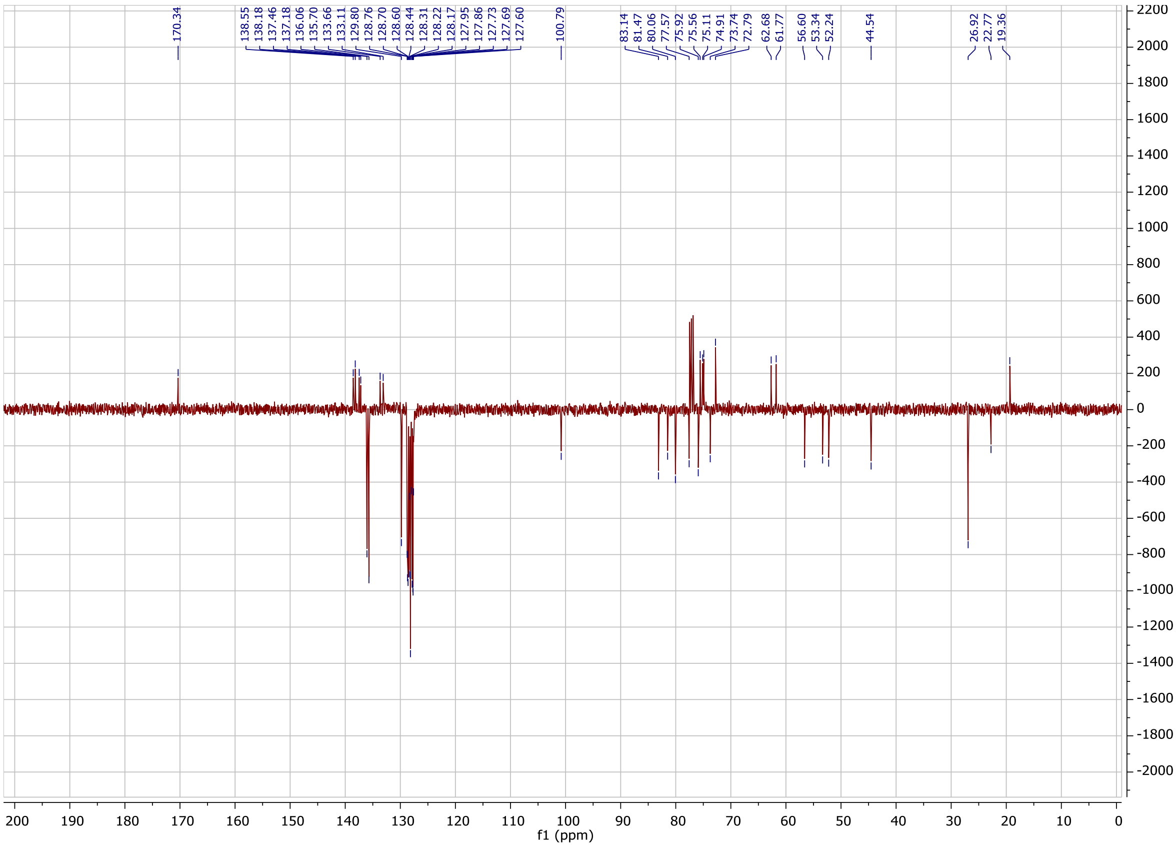

**2,3-di-*O*-benzyl-4-*O*-(2-*N*-acetyl-2-deoxy-3,4-di-*O*-benzyl-6-*O*-tert-butyldiphenylsilyl-α-D-glucopyranosyl)-glucurono-cyclophellitol (15b)**

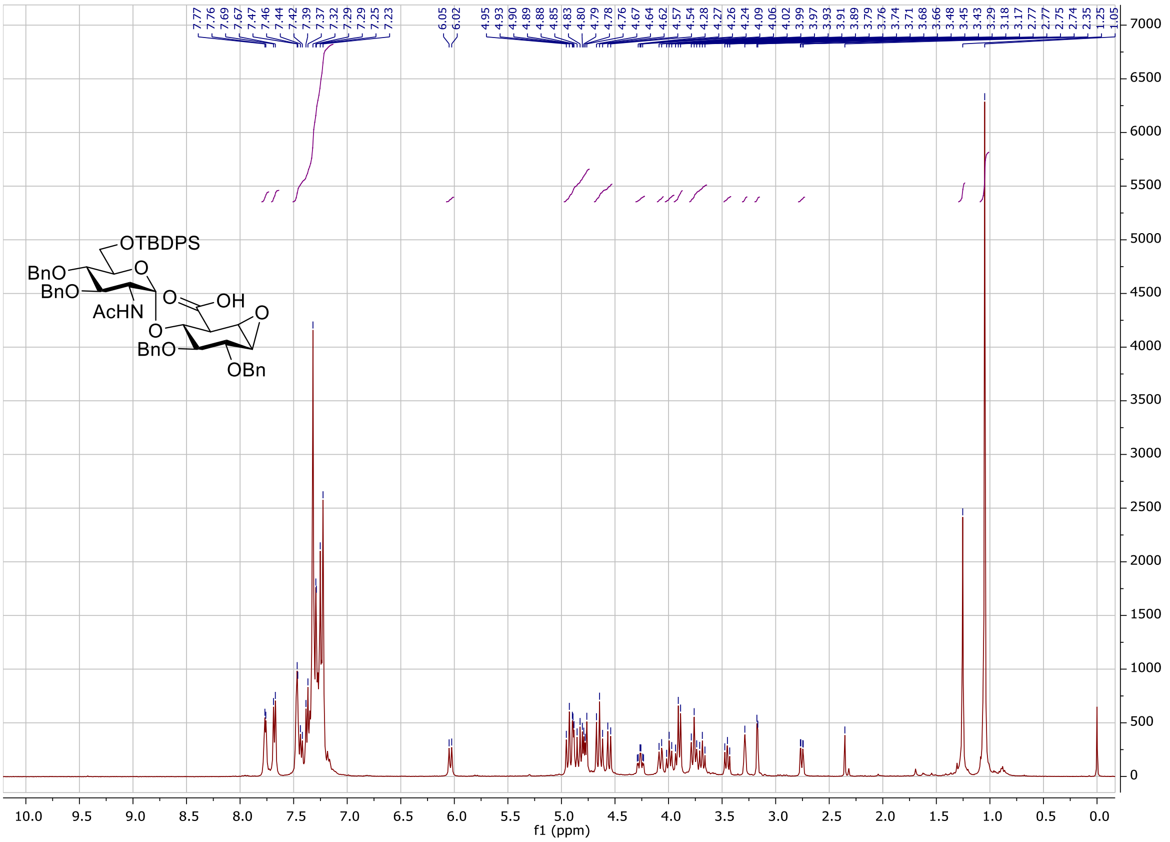


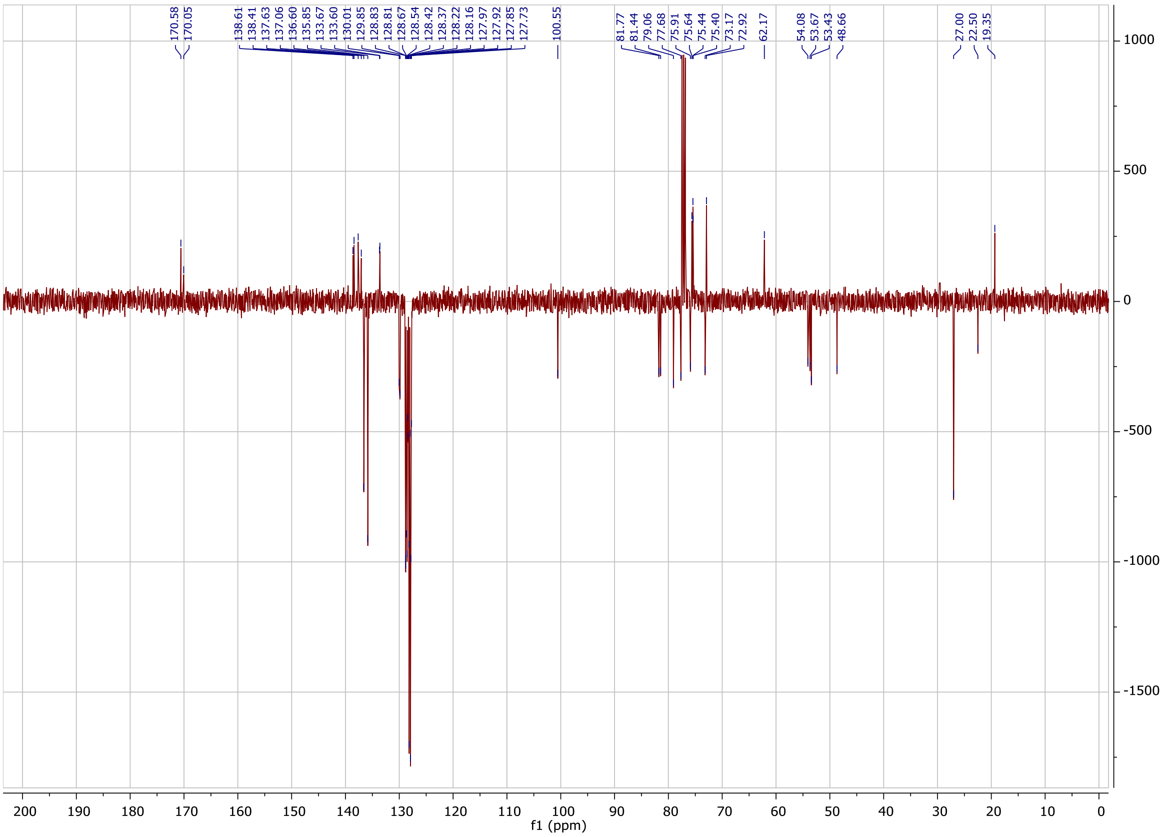

**2,3-di-*O*-benzyl-4-*O*-(2-*N*-acetyl-2-deoxy-3,4-di-*O*-benzyl-α-D-glucopyranosyl)-glucurono-cyclophellitol (16a)**

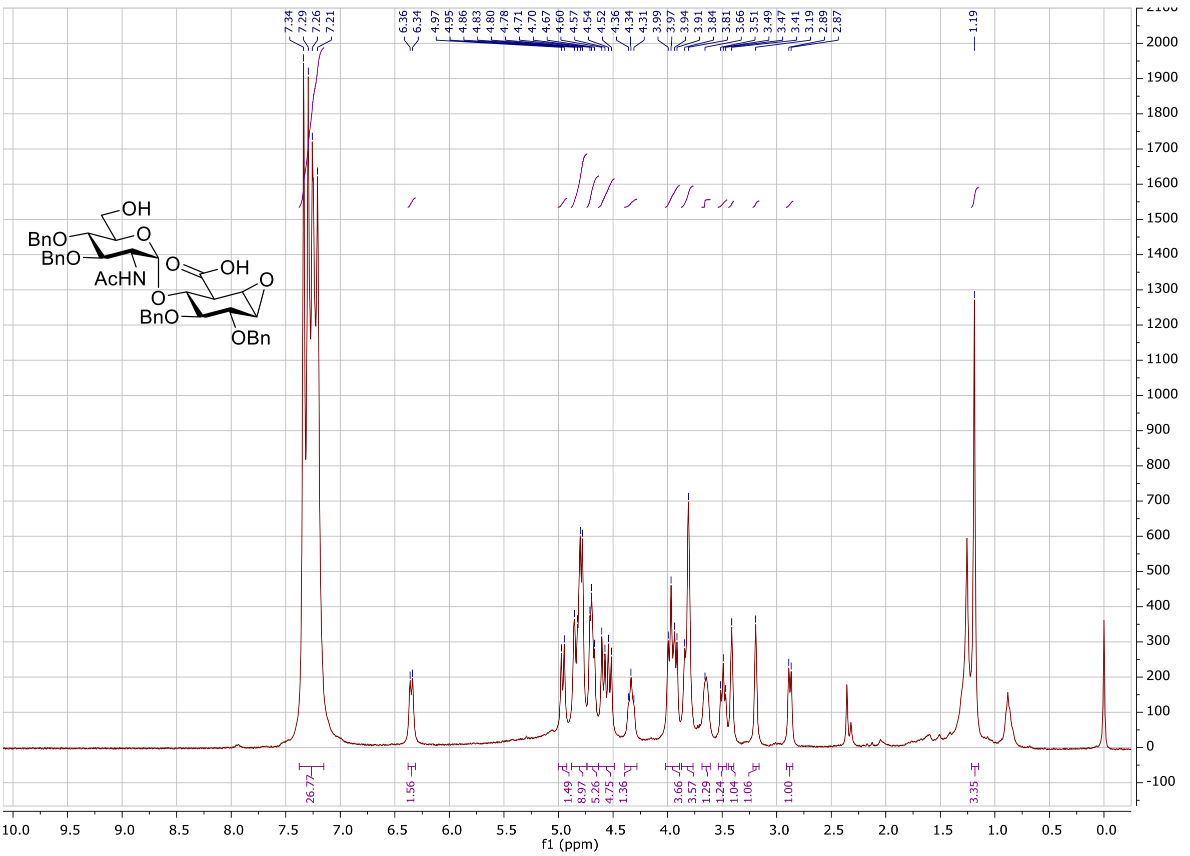

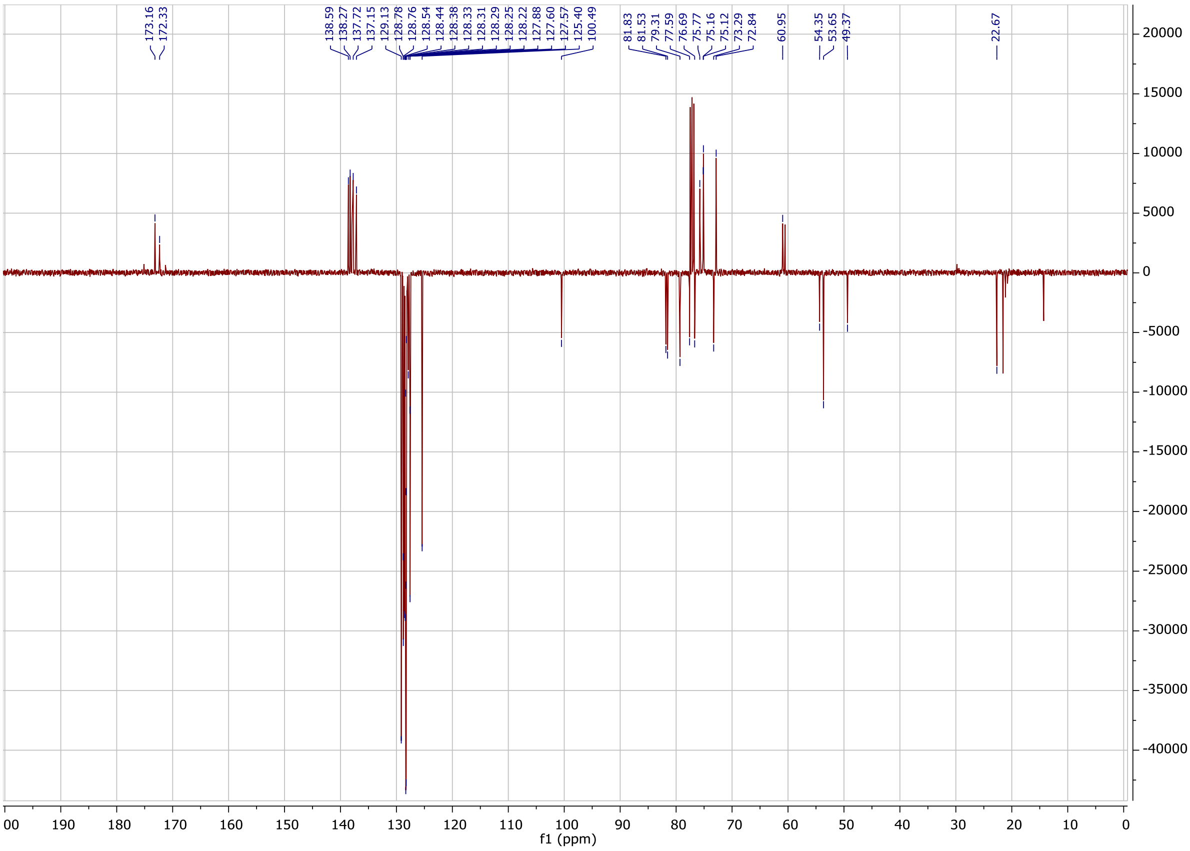

## 4-*O*-(2-*N*-acetyl-2-deoxy-α-D-glucopyranosyl)-glucurono-cyclophellitol (2)

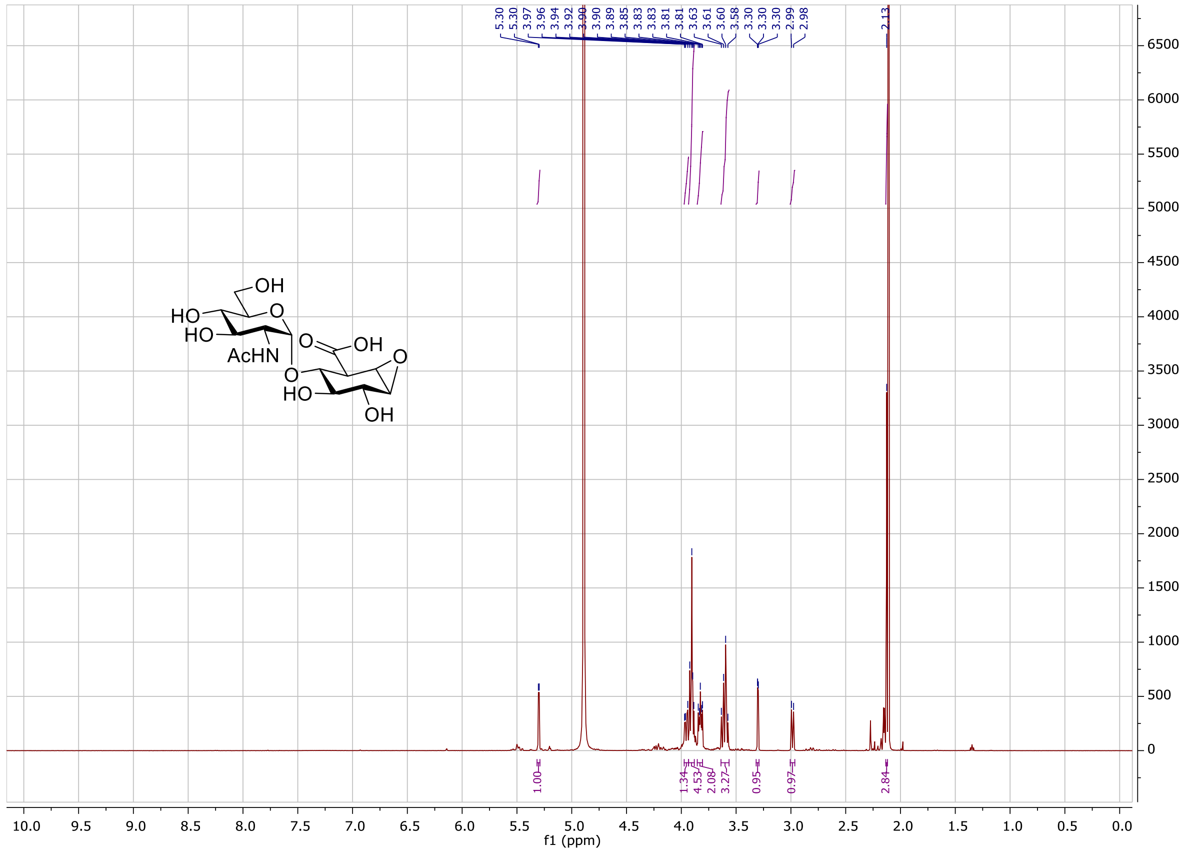


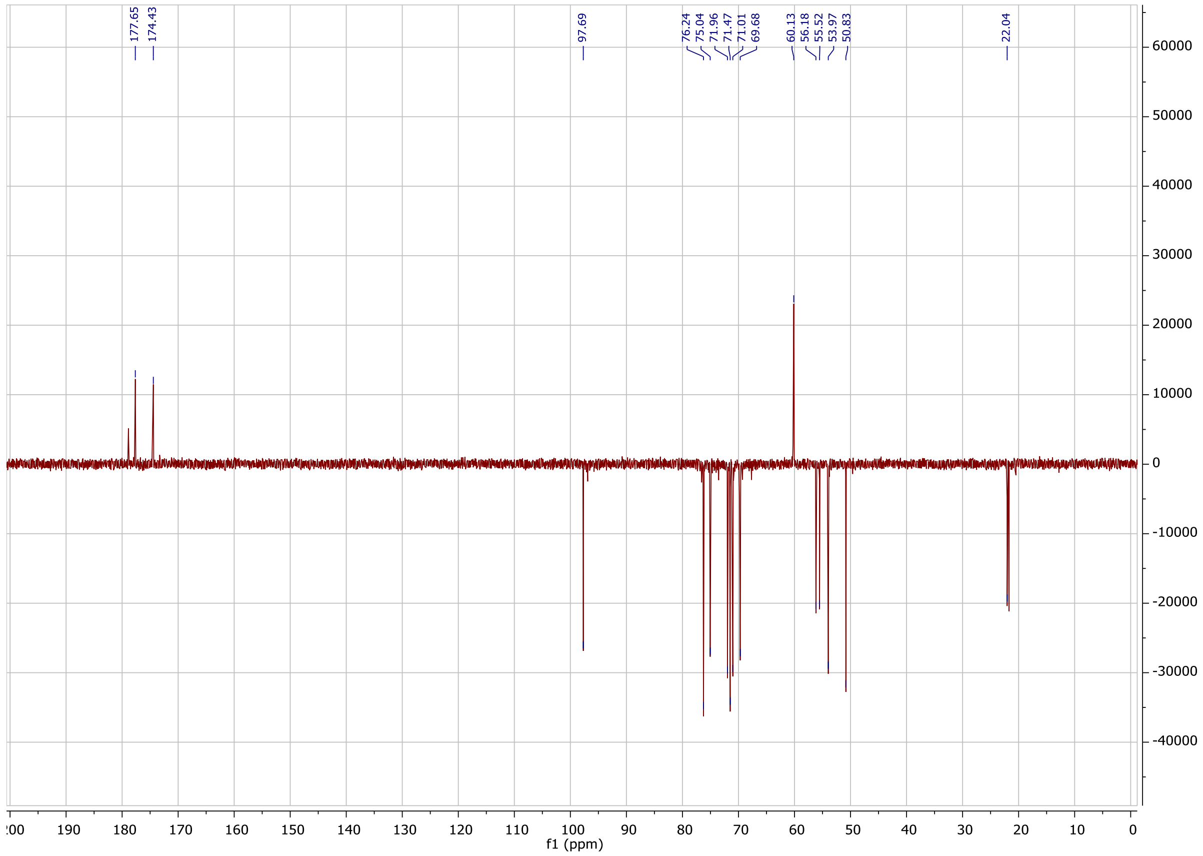

**2,3-di-*O*-benzyl-4-*O*-(2-*N*-acetyl-2-deoxy-3,4-di-*O*-benzyl-6-*O*-sulfo-α-D-glucopyranosyl)-glucurono-cyclophellitol (16b)**

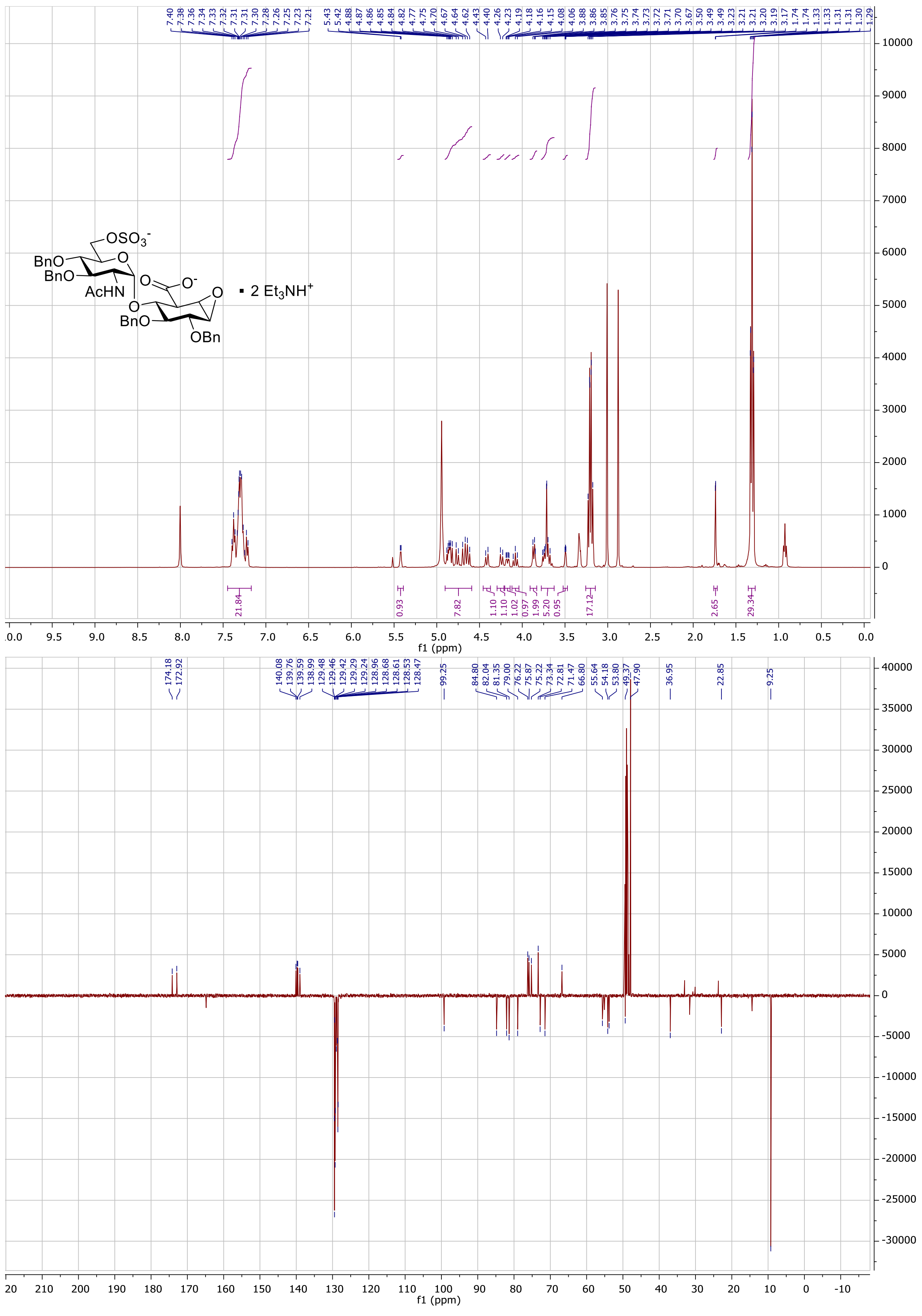

## 4-*O*-(2-*N*-acetyl-2-deoxy-6-*O*-sulfo-α-D-glucopyranosyl)-glucurono-cyclophellitol (3)

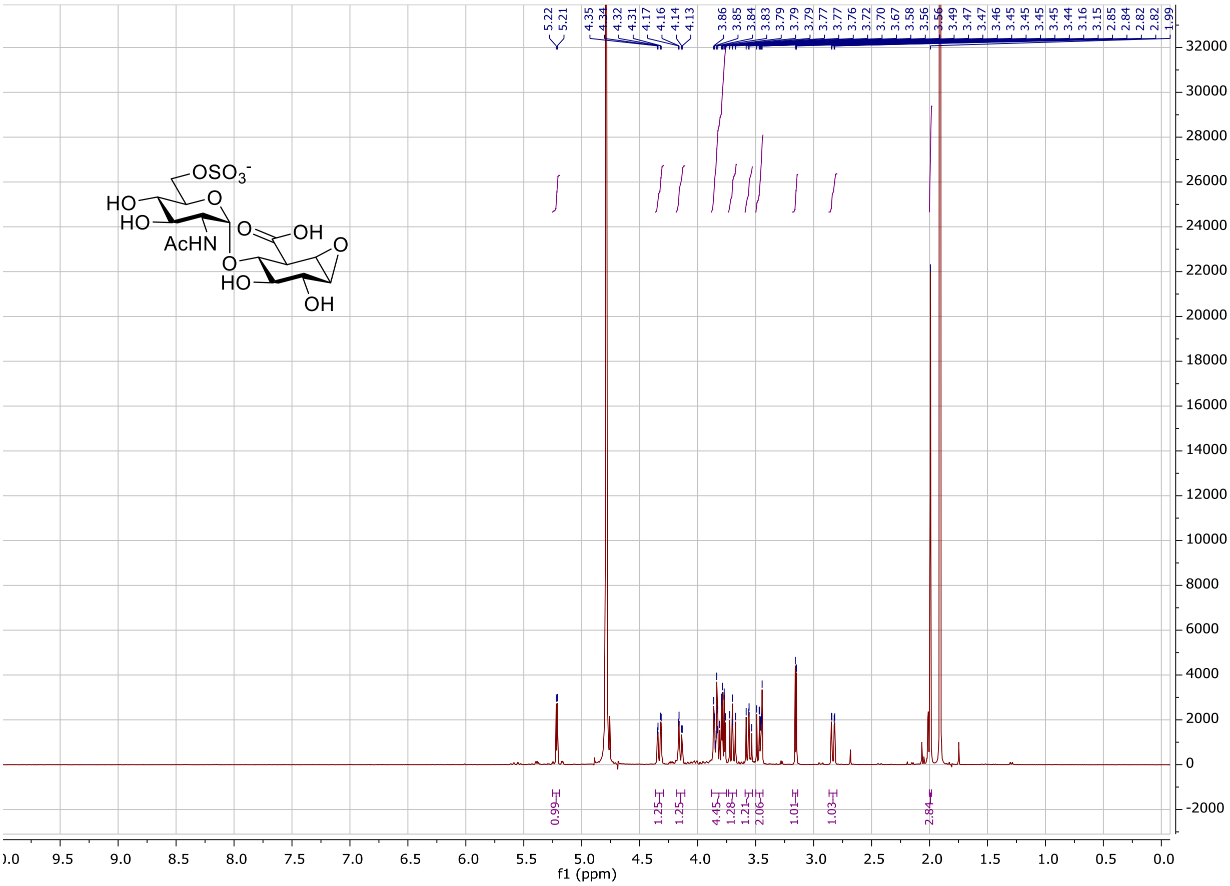


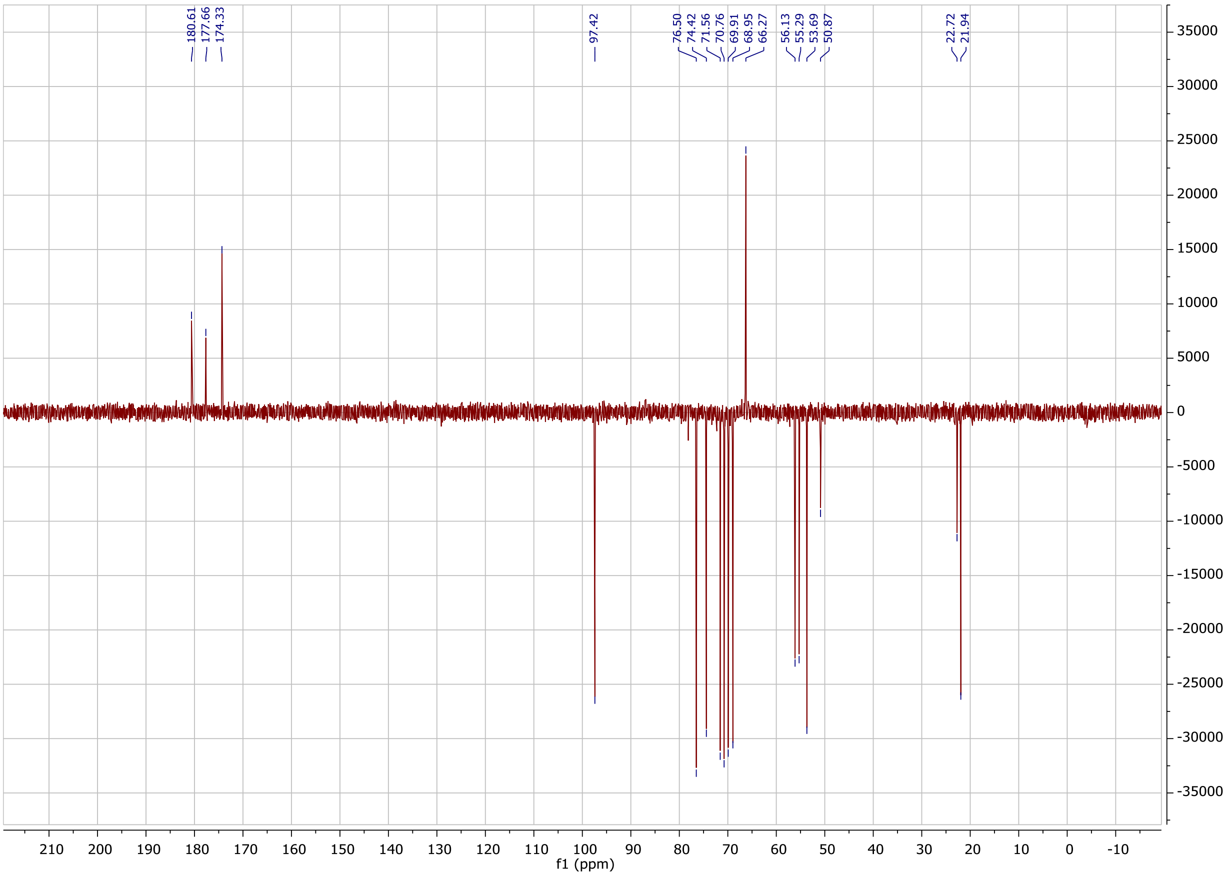

**2,3-di-*O*-benzyl-4-*O*-(2-azido-2-deoxy-3,4-di-*O*-benzyl-6-*O*-tert-butyldiphenylsilyl-α-D-glucopyranosyl)-cyclophellitol alkene (17)**

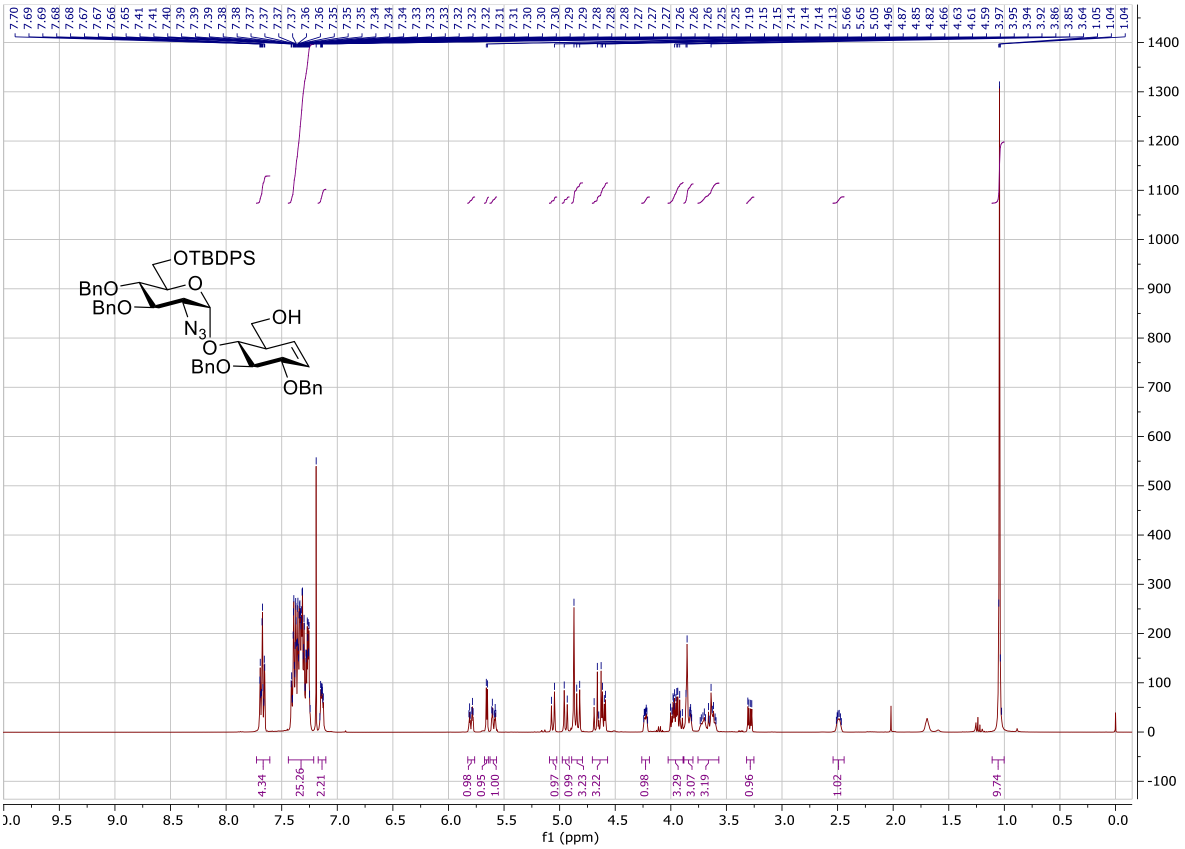


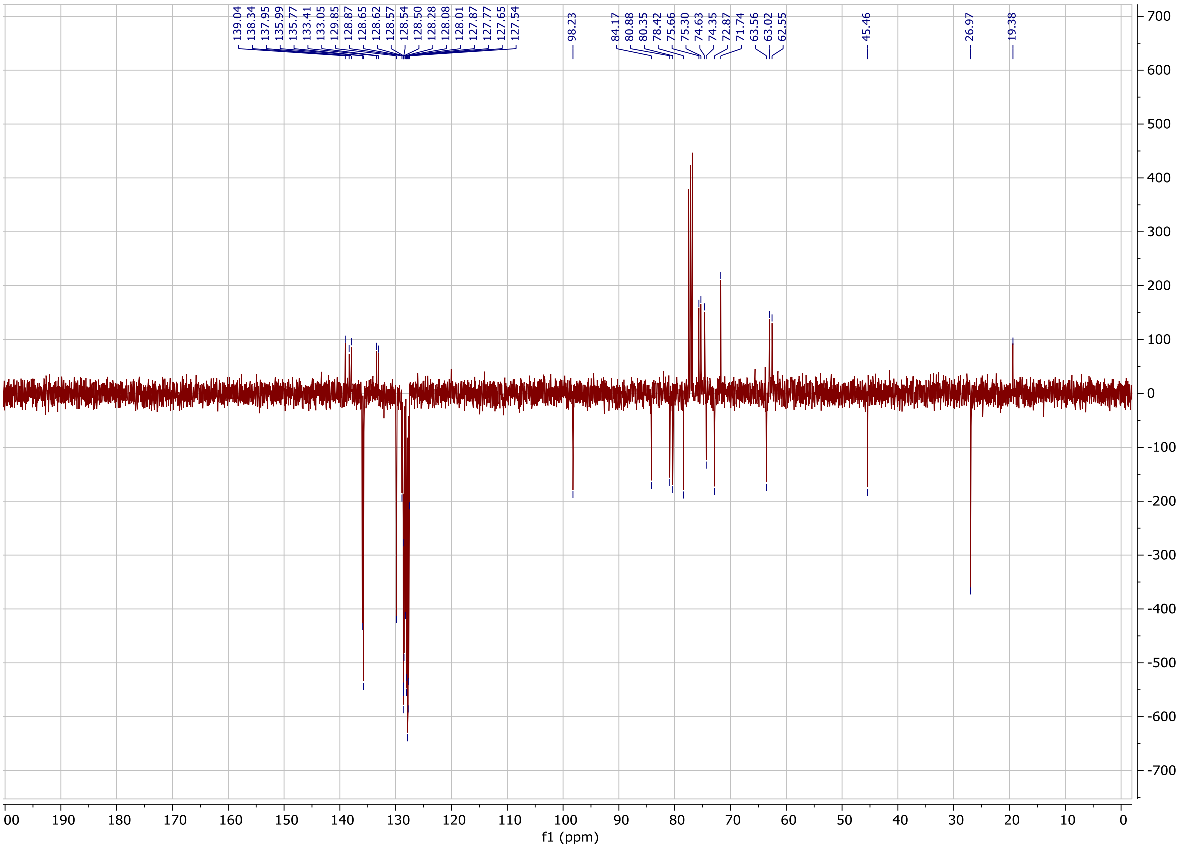

**2,3-di-*O*-benzyl-4-*O*-(2-azido-2-deoxy-3,4-di-*O*-benzyl-6-*O*-tert-butyldiphenylsilyl-α-D-glucopyranosyl)-6-*O*-tert-butyloxycarbonyl-cyclophellitol alkene (17a)**

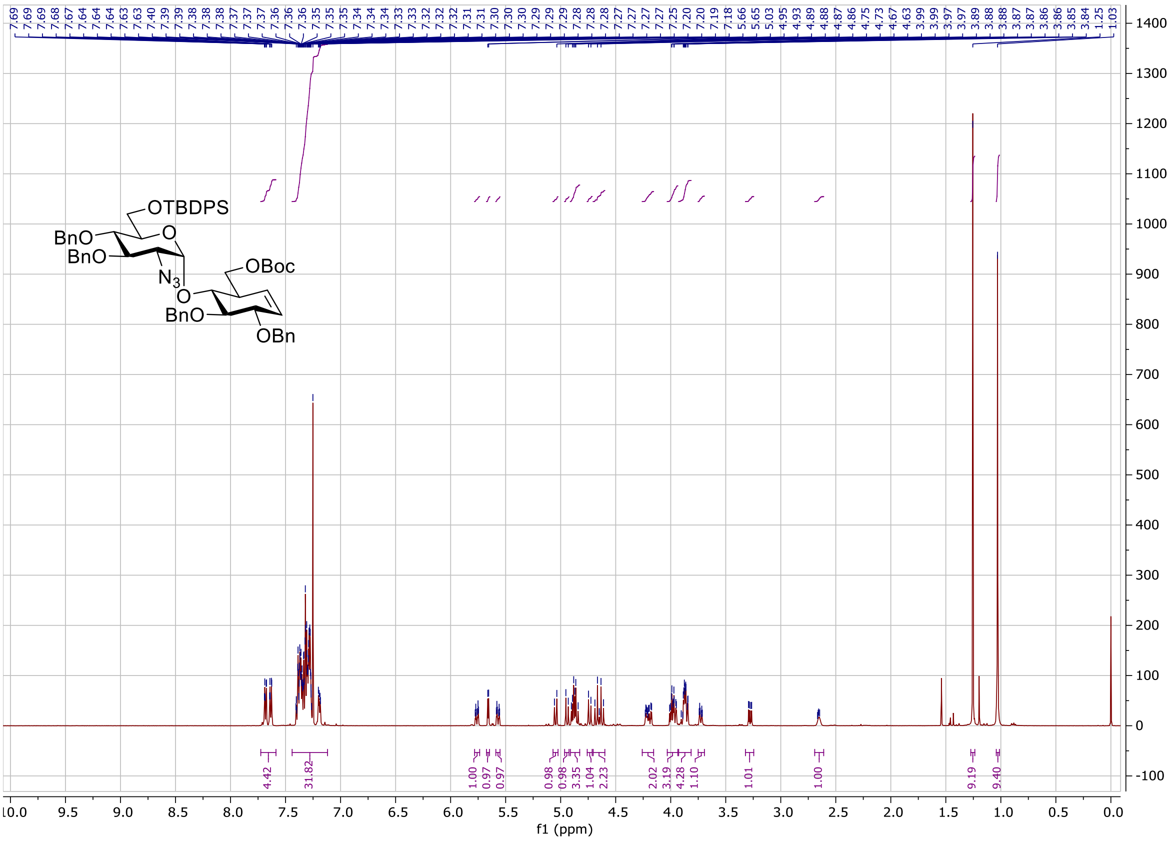


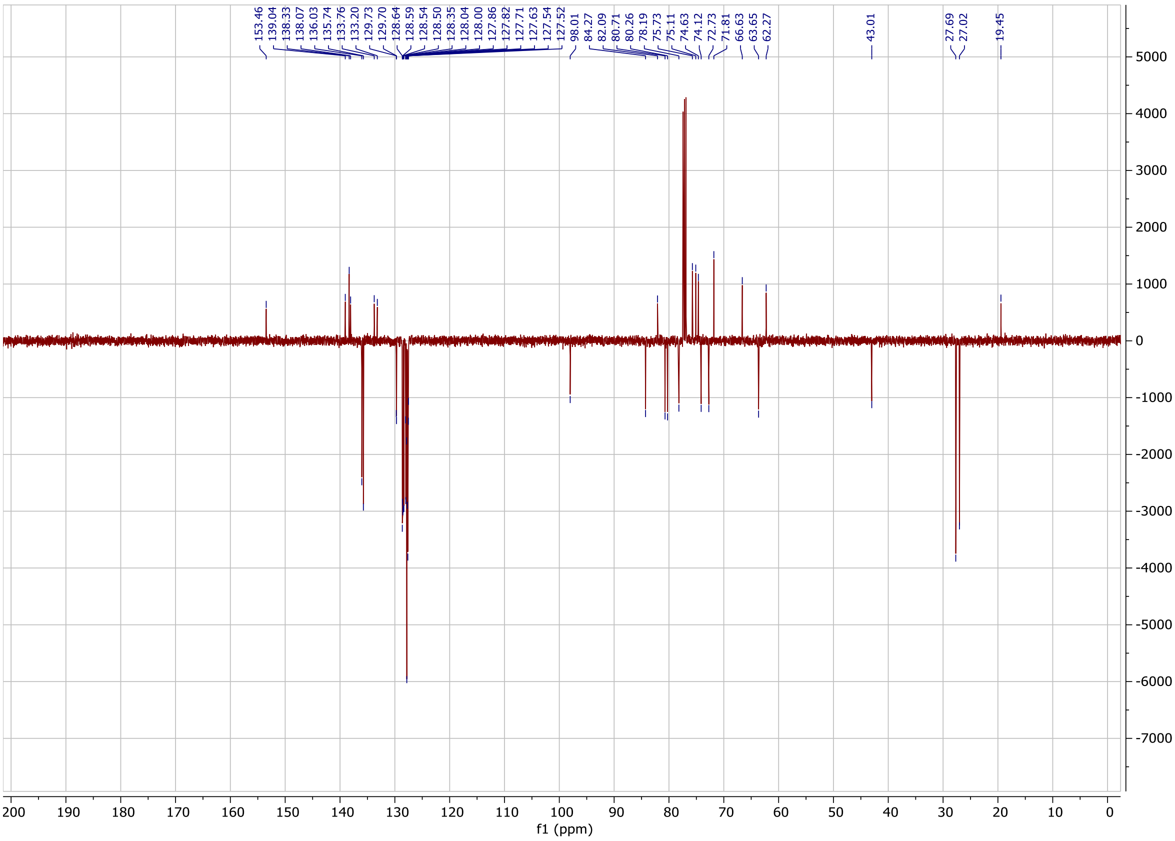

**1-iodo-2,3-di-*O*-benzyl-4-*O*-(2-azido-2-deoxy-3,4-di-*O*-benzyl-6-*O*-tert-butyldiphenylsilyl-α-D-glucopyranosyl)-6,7-*O*-carbonyl-cyclophellitol alkene (17b)**

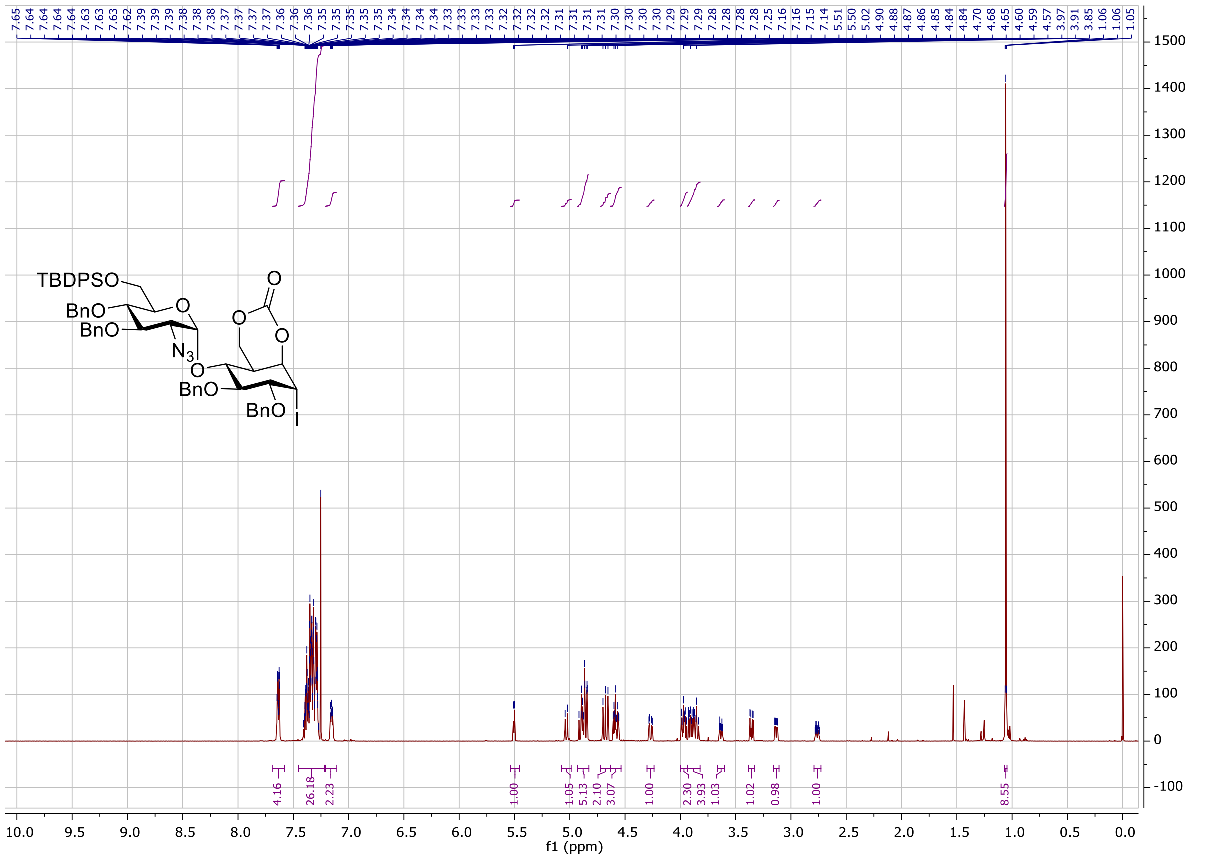

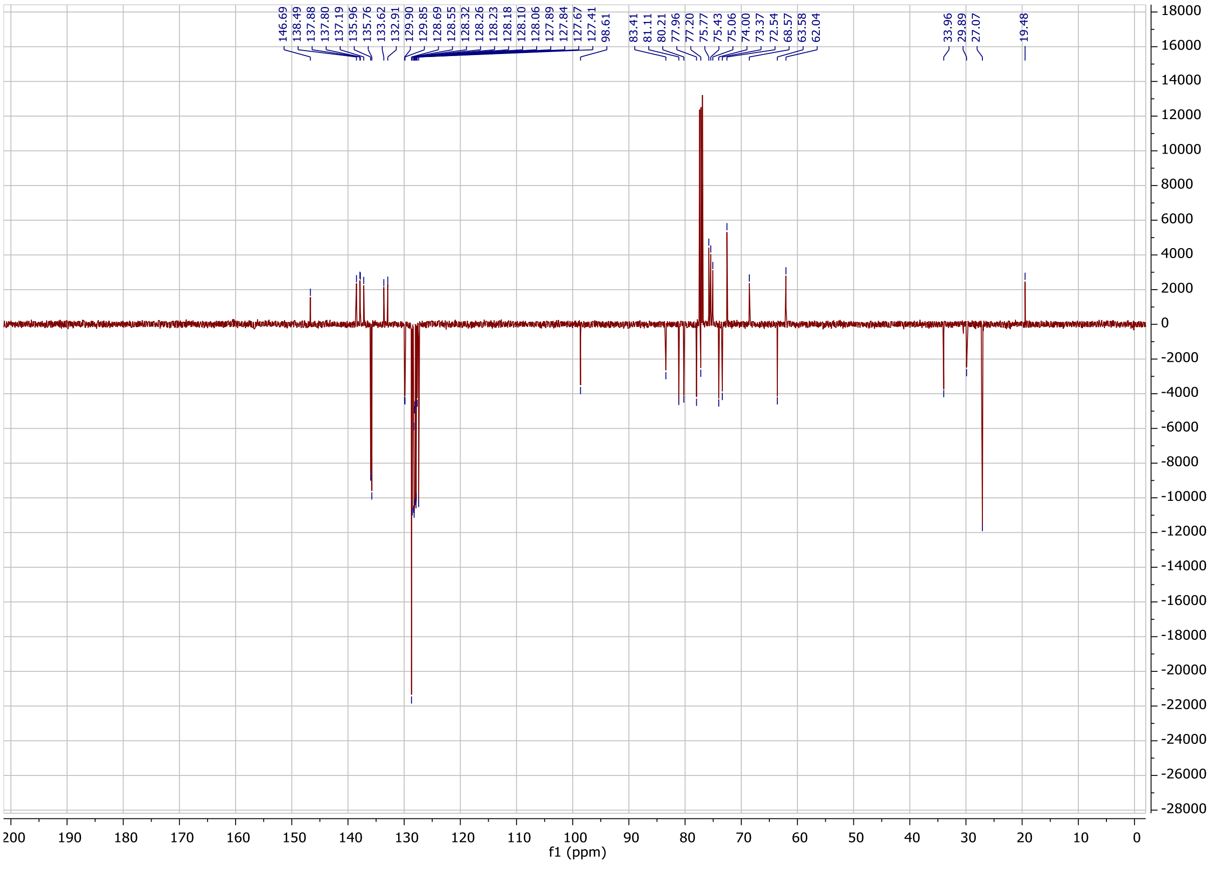

**2,3-di-*O*-benzyl-4-*O*-(2-azido-2-deoxy-3,4-di-*O*-benzyl-6-*O*-tert-butyldiphenylsilyl-α-D-glucopyranosyl)-cyclophellitol (18)**

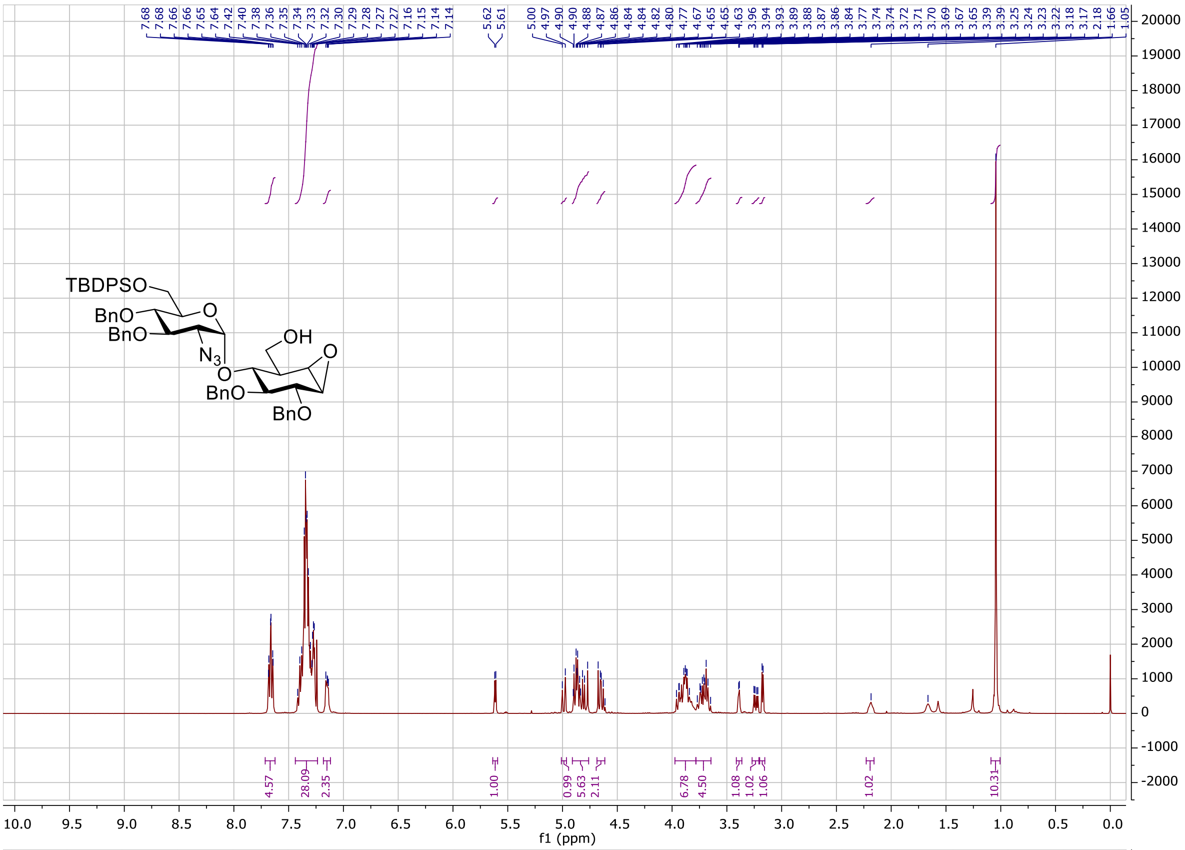

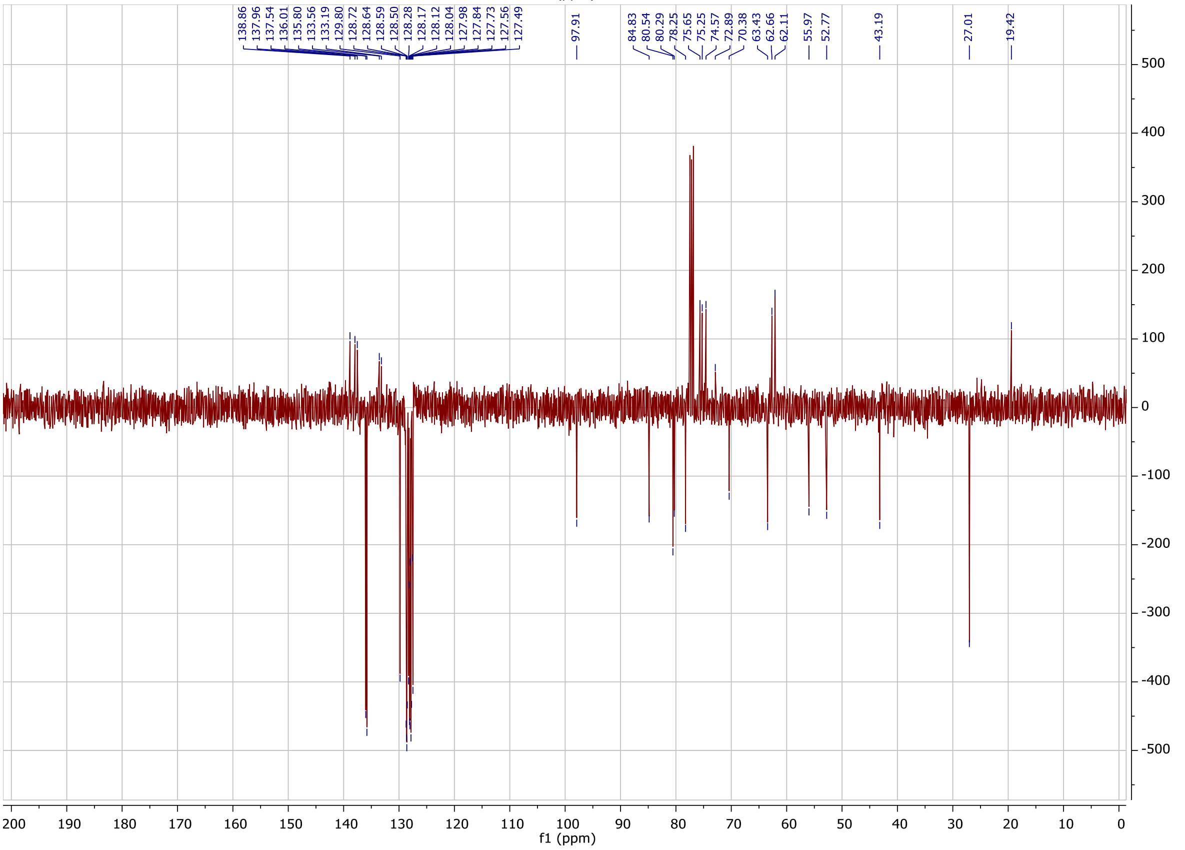

**2,3-di-*O*-benzyl-4-*O*-(2-azido-2-deoxy-3,4-di-*O*-benzyl-6-*O*-tert-butyldiphenylsilyl-α-D-glucopyranosyl)-glucurono-cyclophellitol (19)**

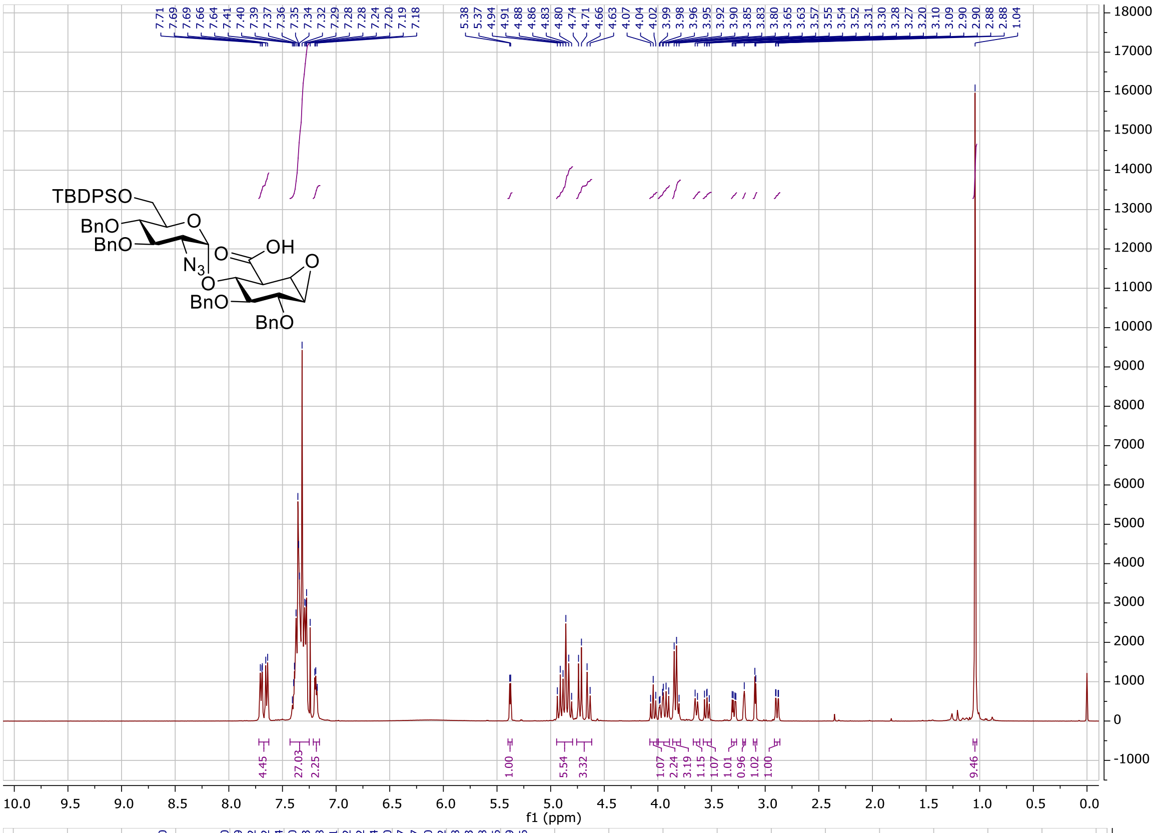


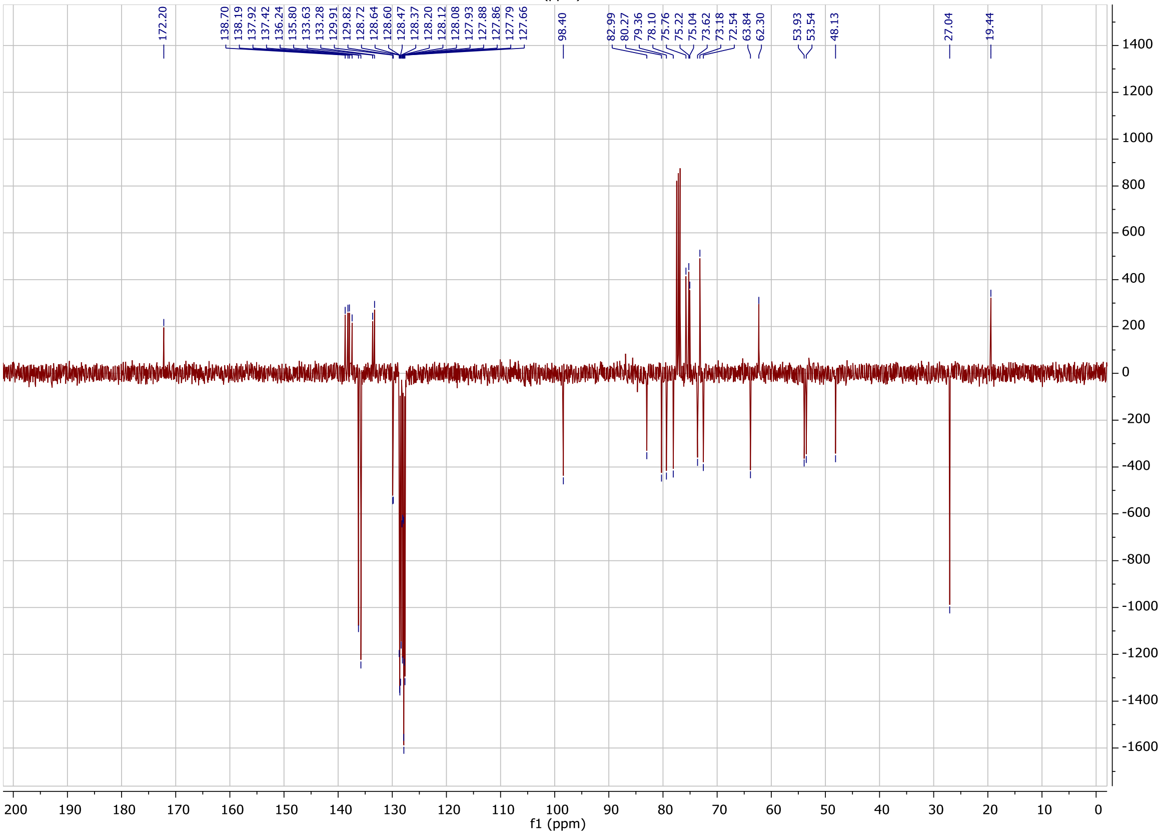

**2,3-di-*O*-benzyl-4-*O*-(2-azido-2-deoxy-3,4-di-*O*-benzyl-α-D-glucopyranosyl)-glucurono-cyclophellitol (20)**

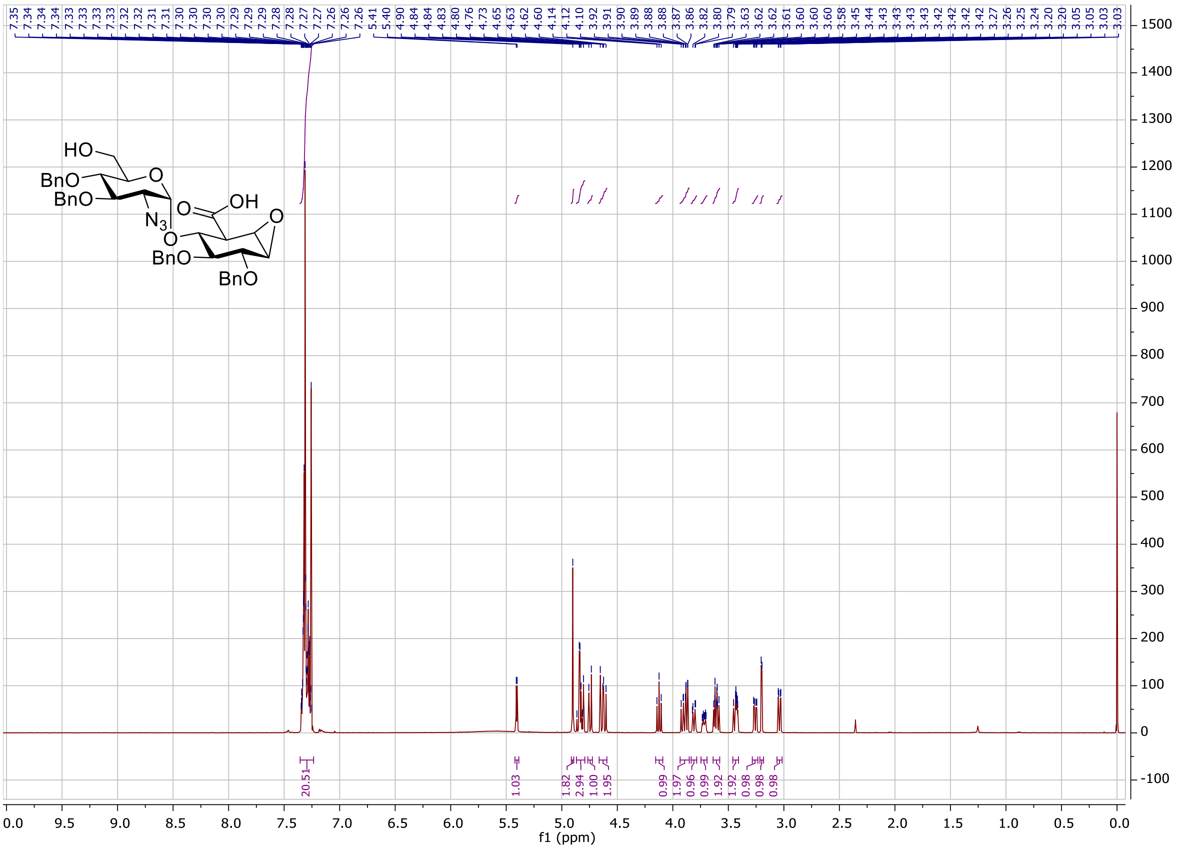

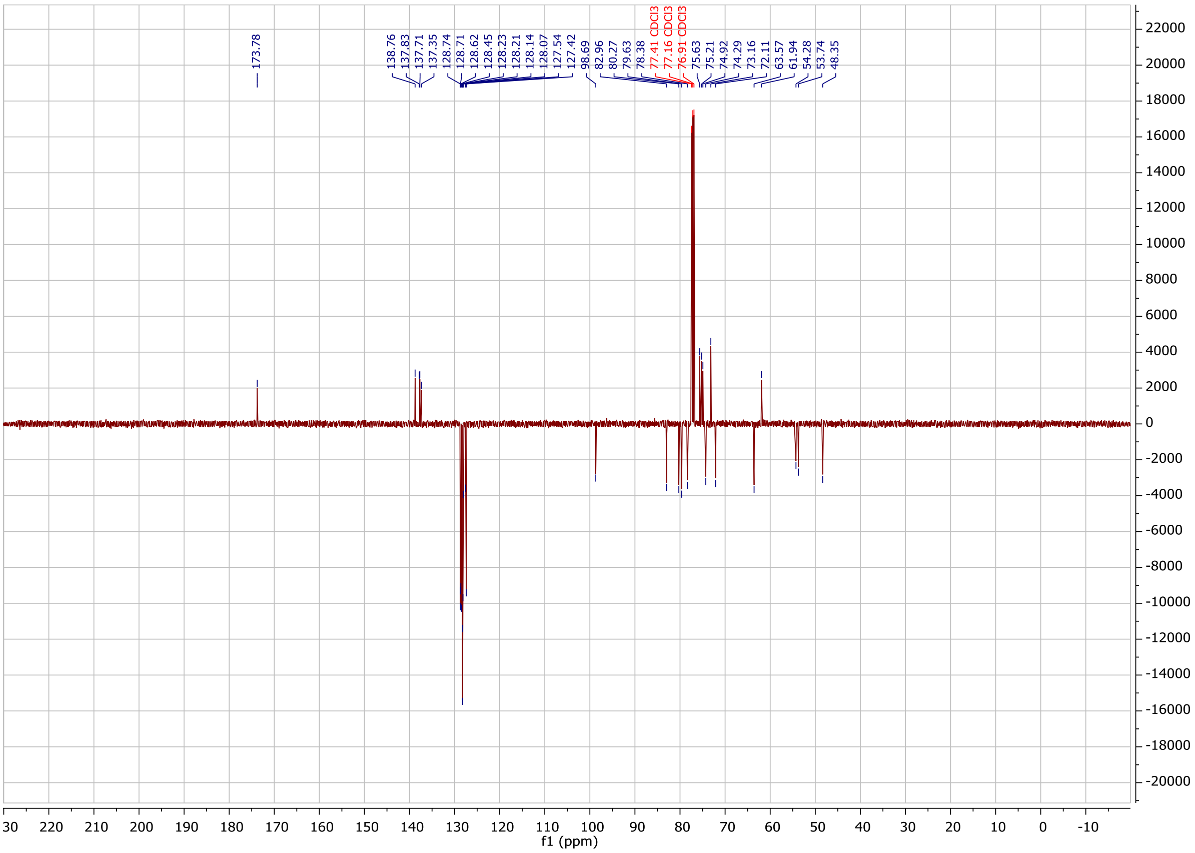

# 4-*O*-(2-deoxy-α-D-glucopyranosyl)-glucurono-cyclophellitol (4)

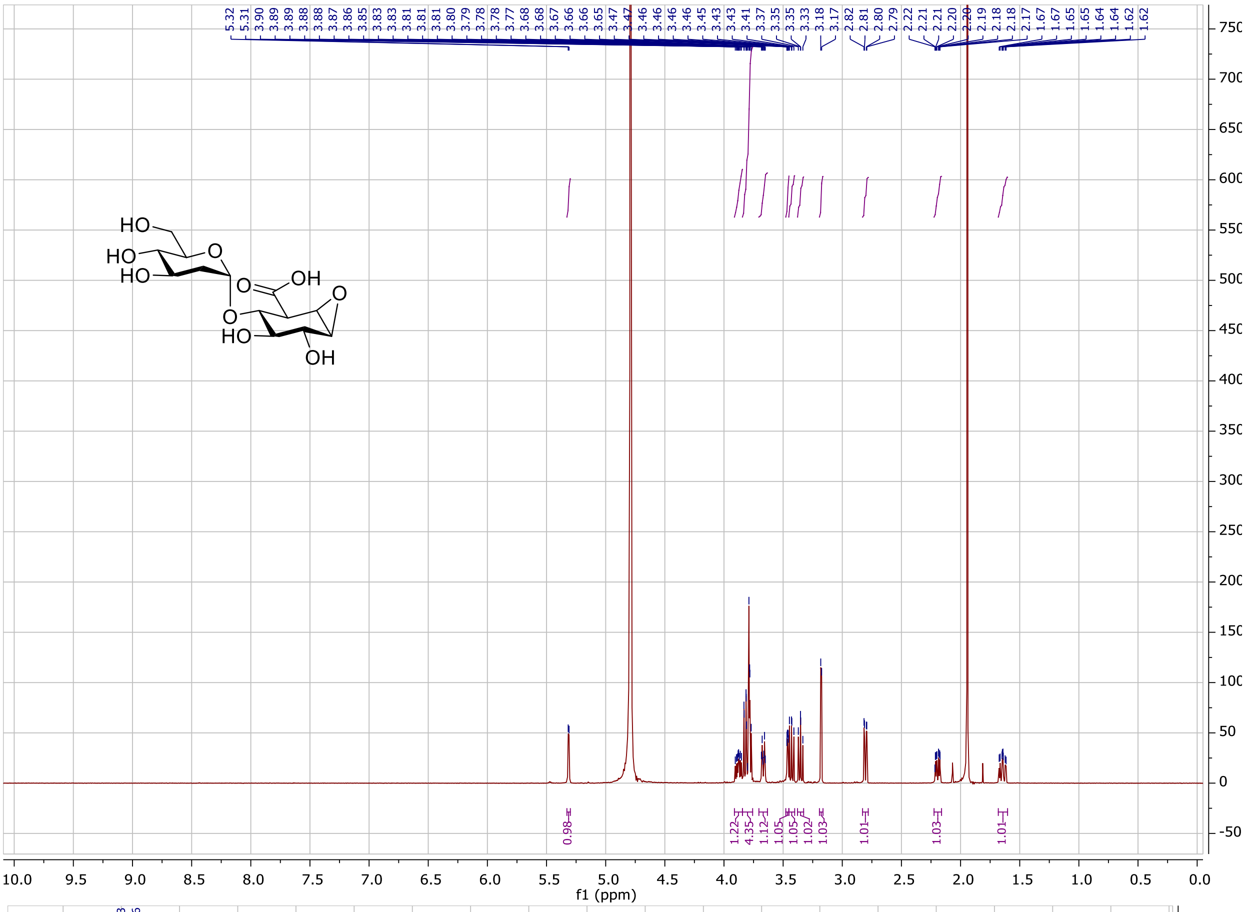

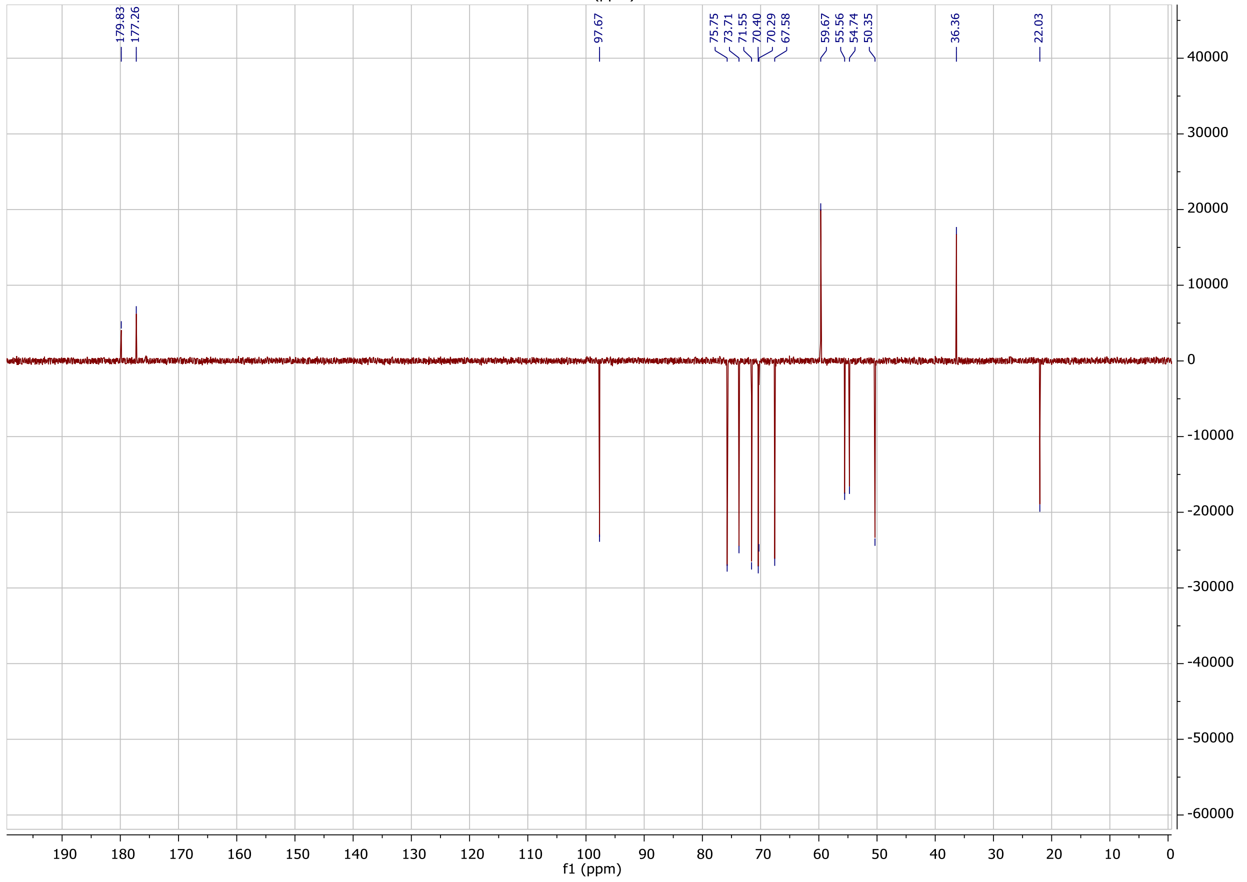

**2,3-di-*O*-benzyl-4-*O*-(2-amino-2-deoxy-3,4-di-*O*-benzyl-α-D-glucopyranosyl)-glucurono-cyclophellitol (21)**

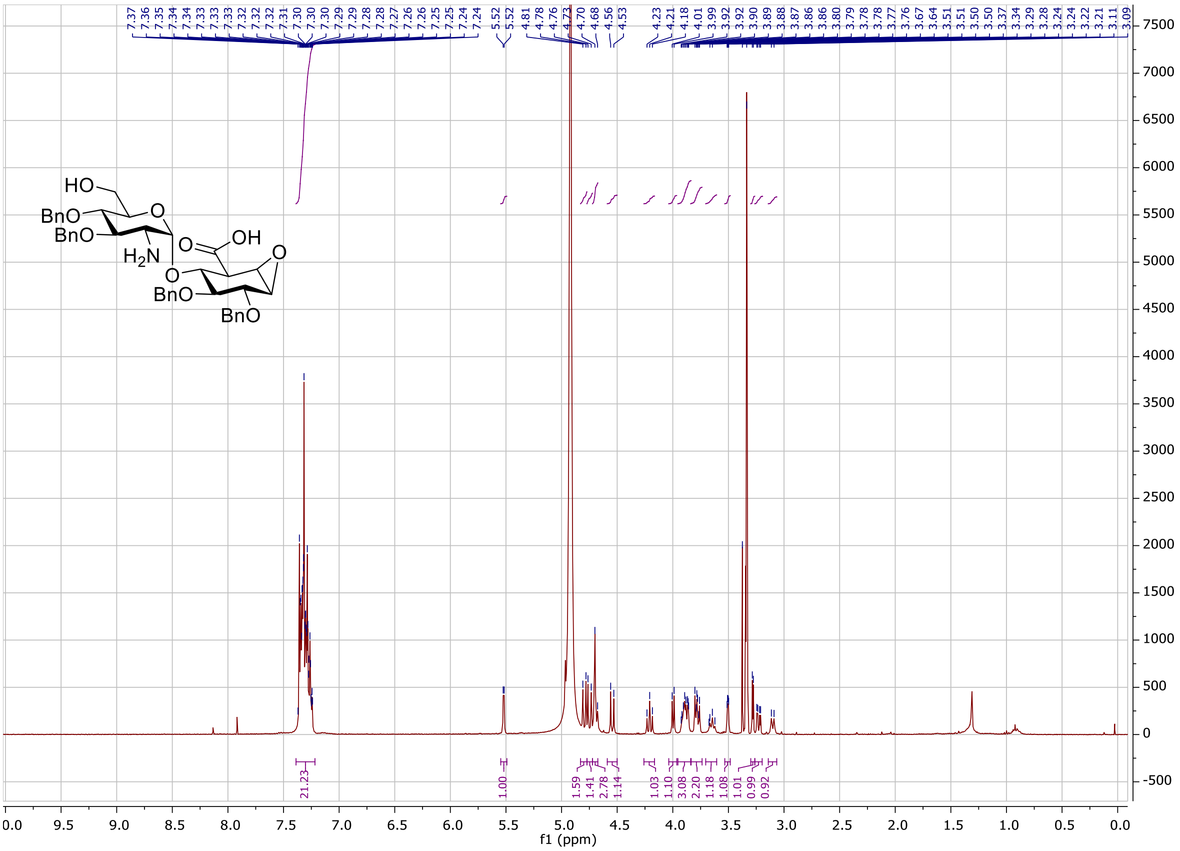

# 4-*O*-(2-amino-2-deoxy-α-D-glucopyranosyl)-glucurono-cyclophellitol (5)

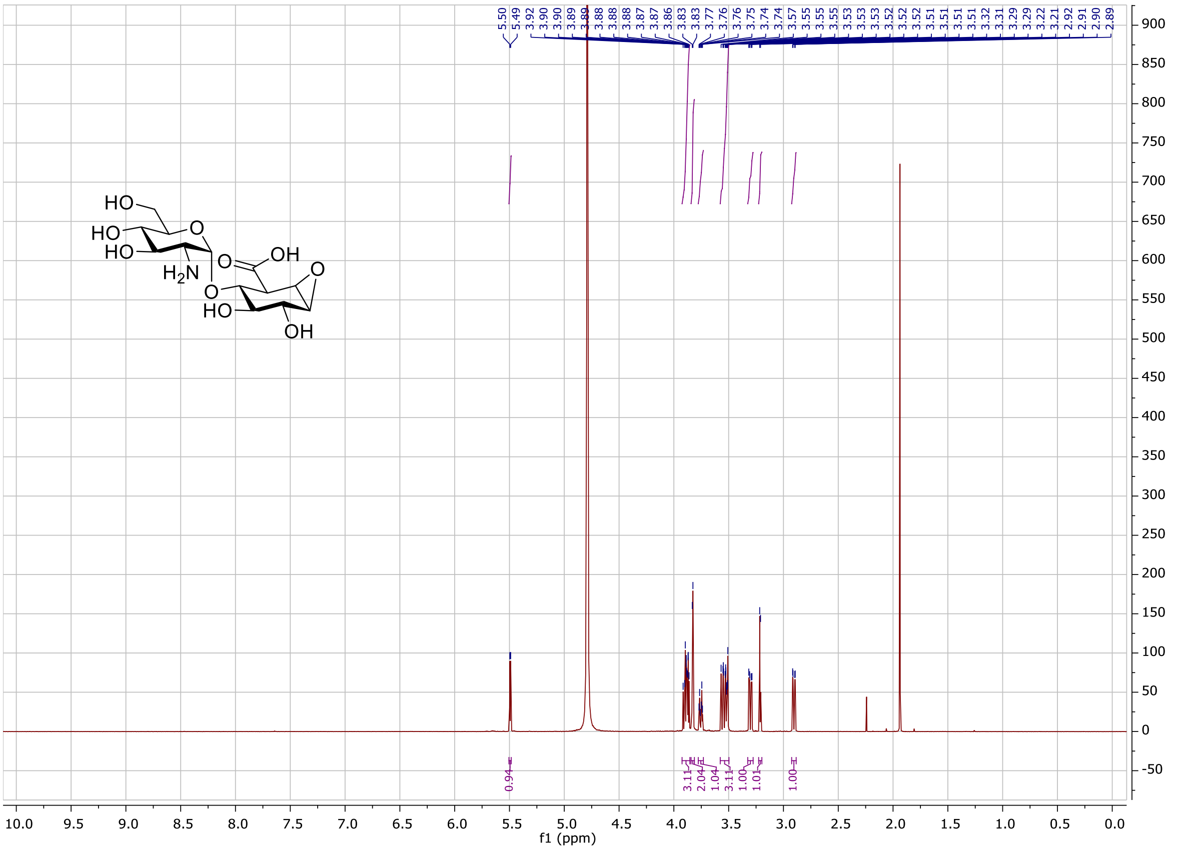


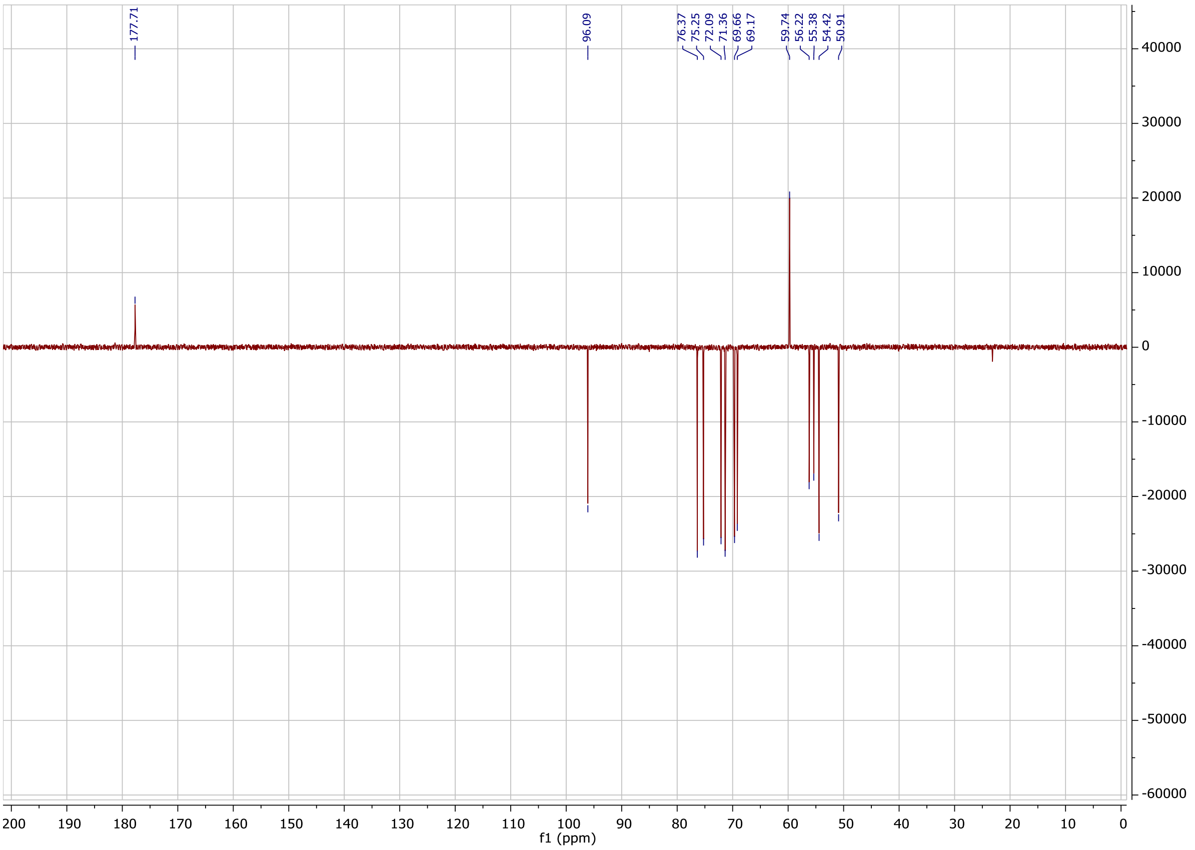

**4-*O*-(2-*N*-azidoacetyl-2-deoxy-α-D-glucopyranosyl)-glucurono-cyclophellitol (6)**

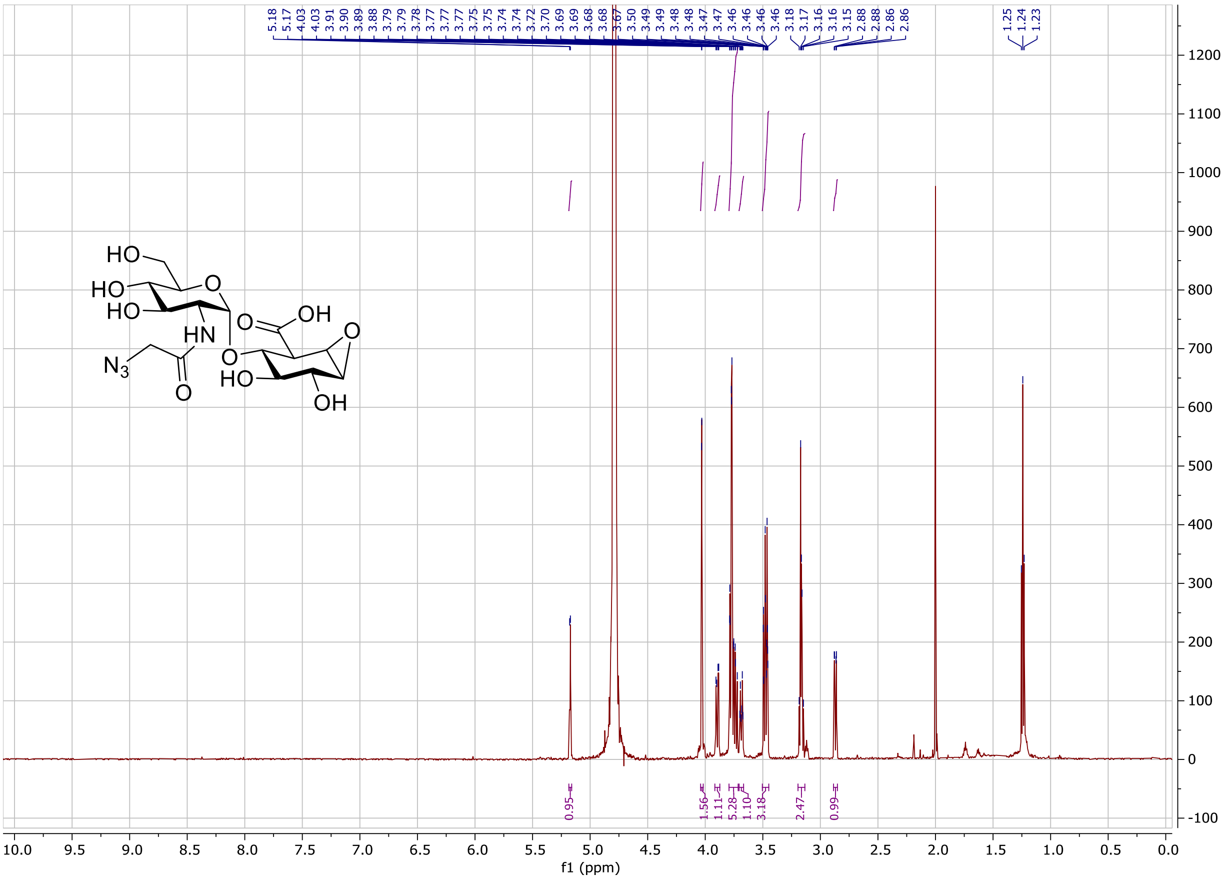


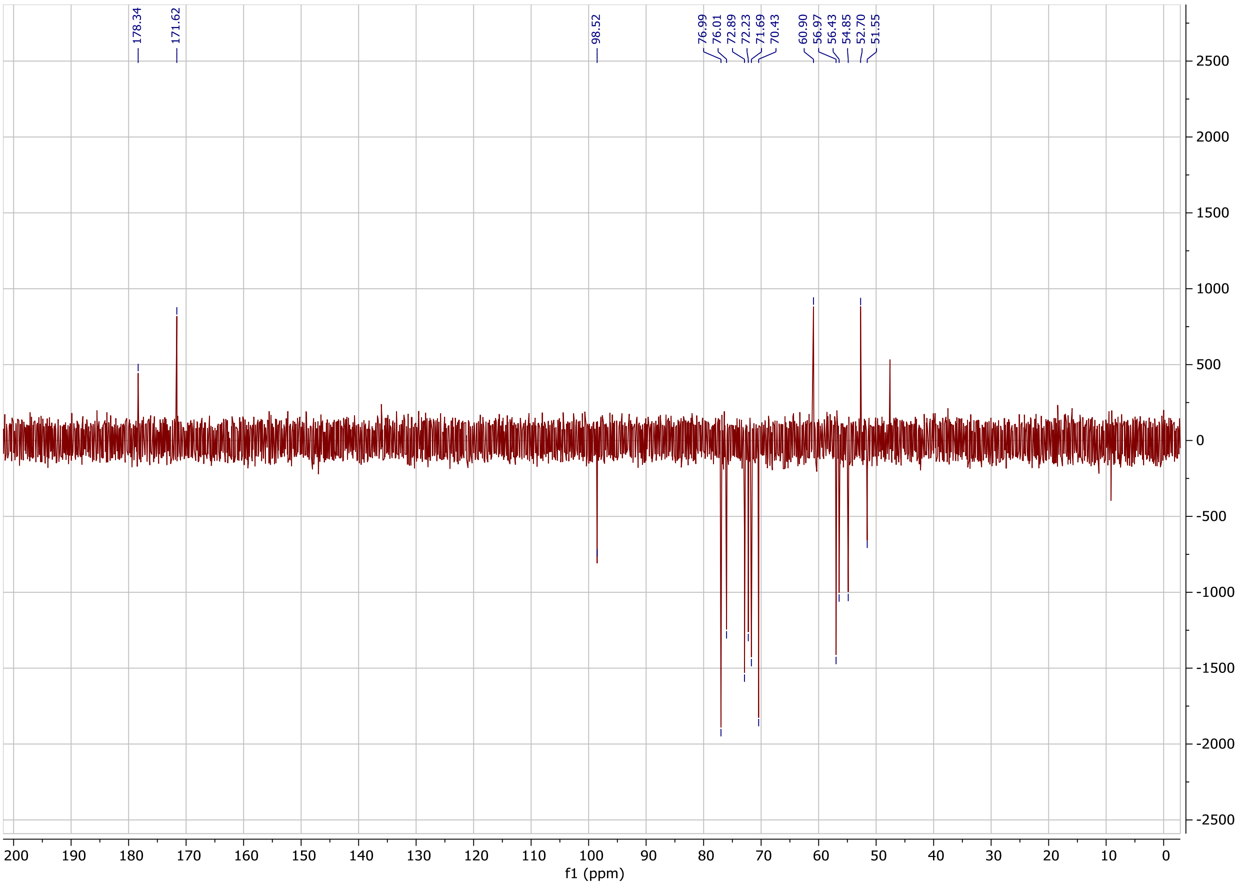

## 2,3-di-*O*-benzyl-6-*O*-(4-methoxybenzyl)-cyclophellitol alkene (12c)

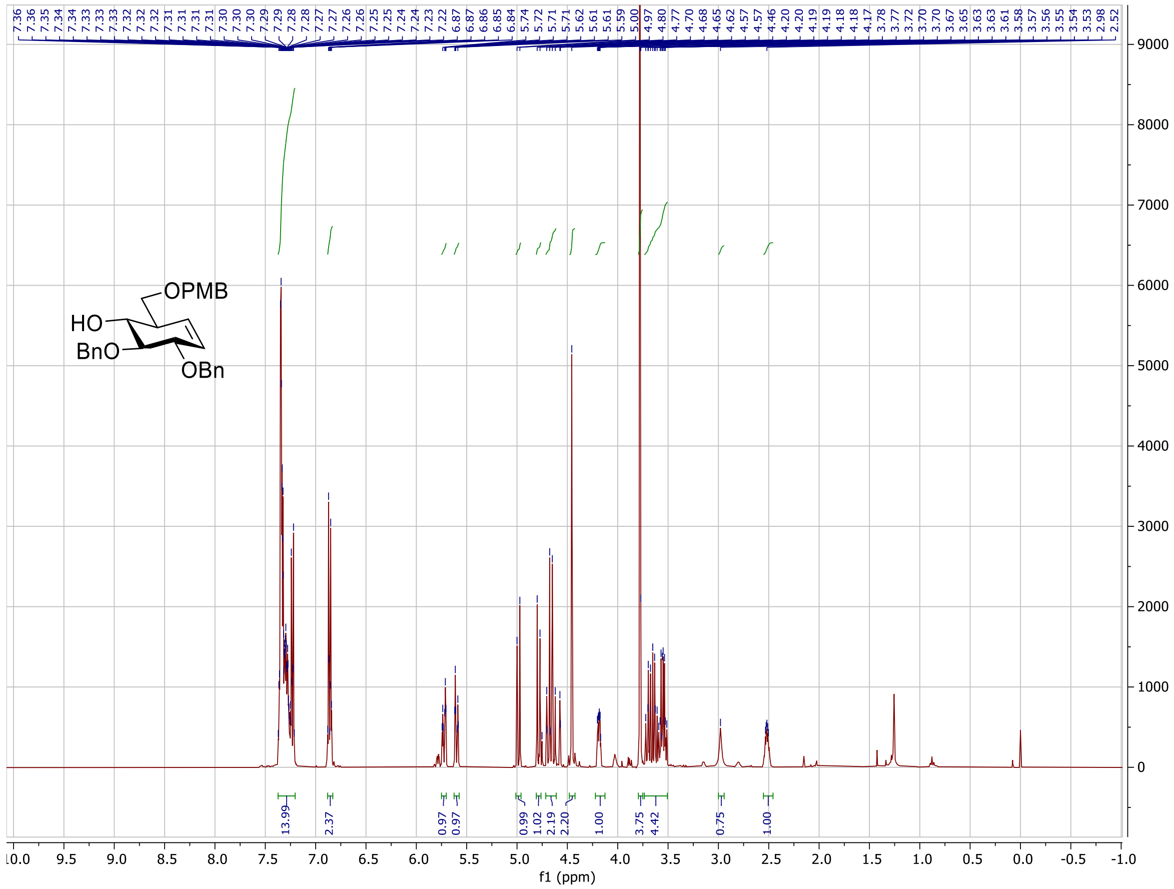


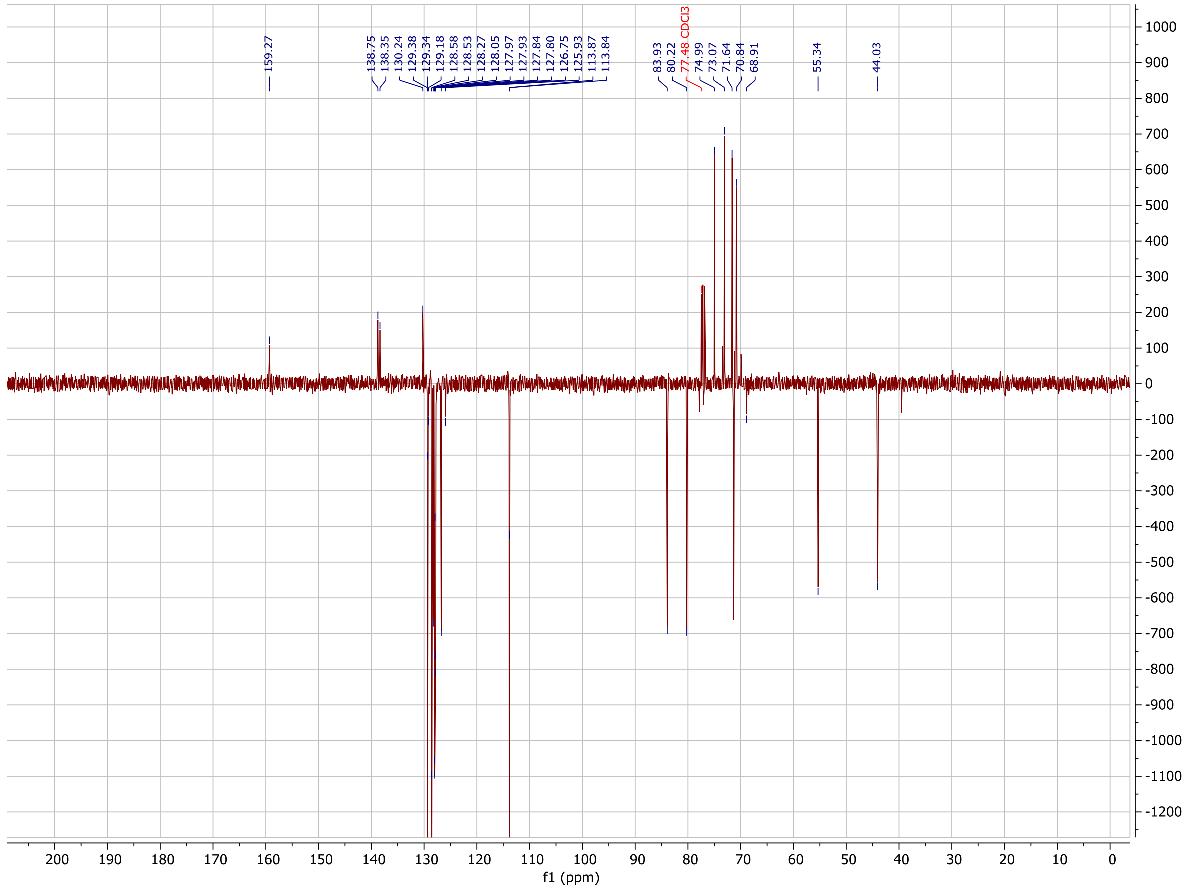

## 8-azidooctyl 4-methylbenzenesulfonate (23)

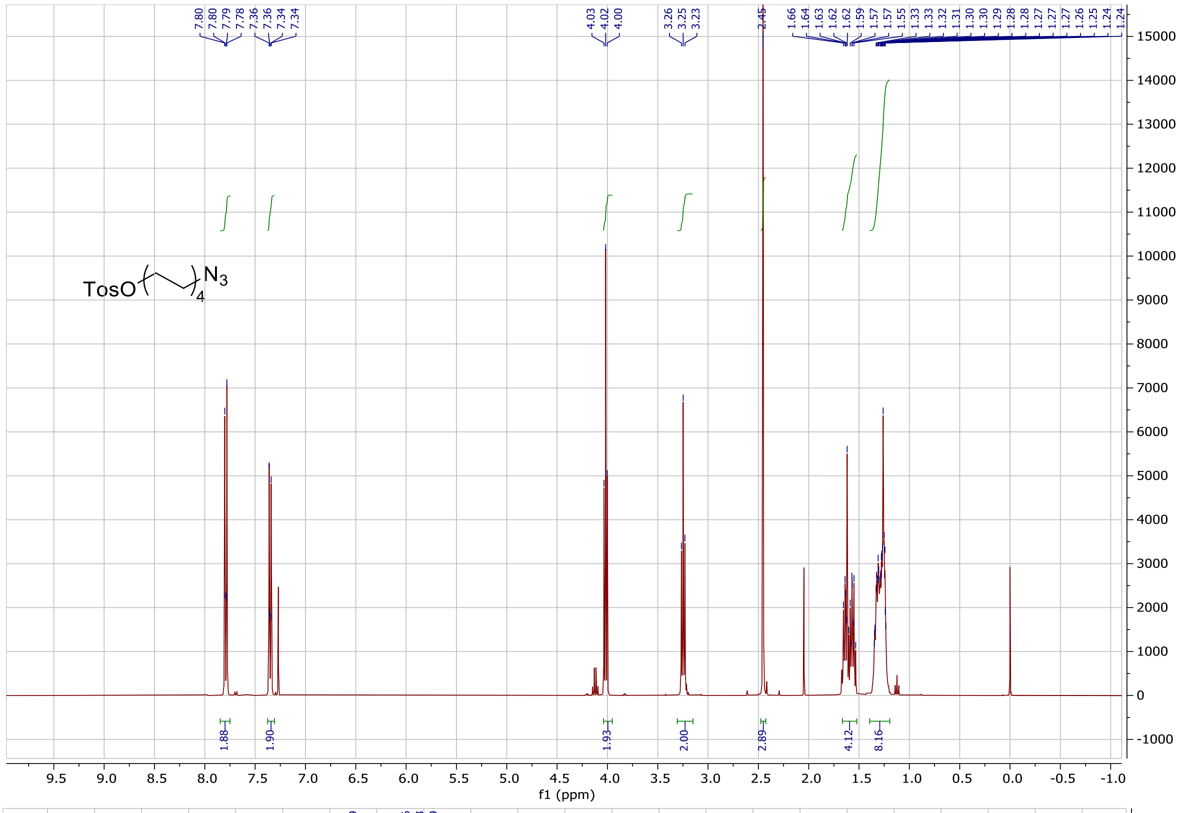


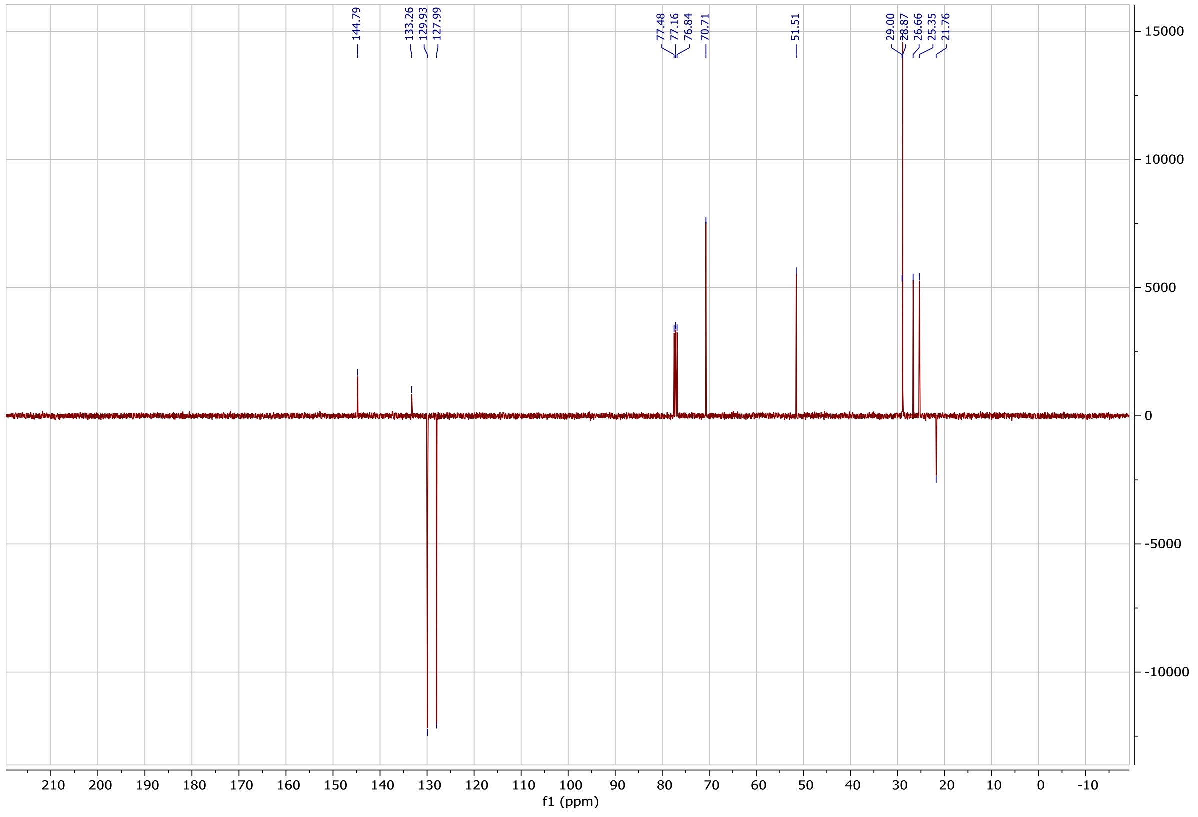

## 2,3-di-*O*-benzyl-4-(8-azidooctyl)-6-*O*-(4-methoxybenzyl)-cyclophellitol alkene (24a)

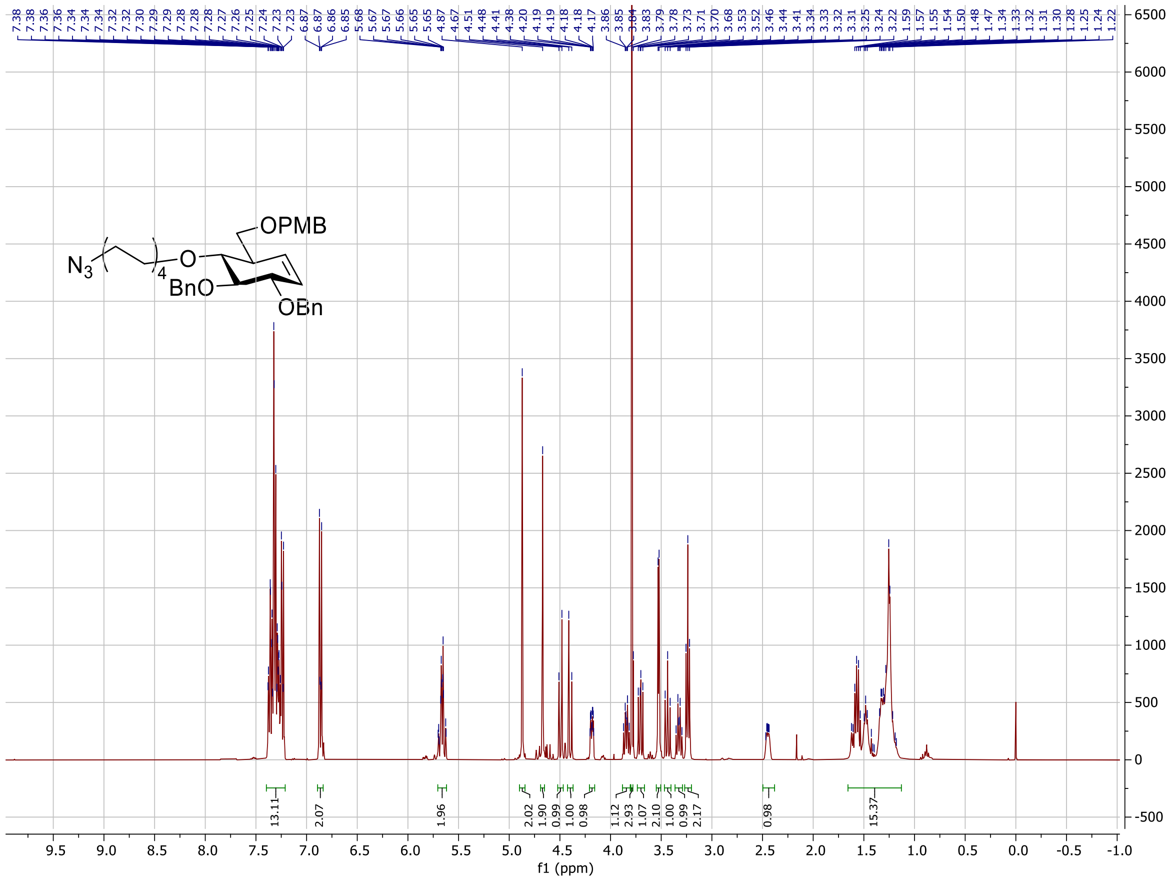

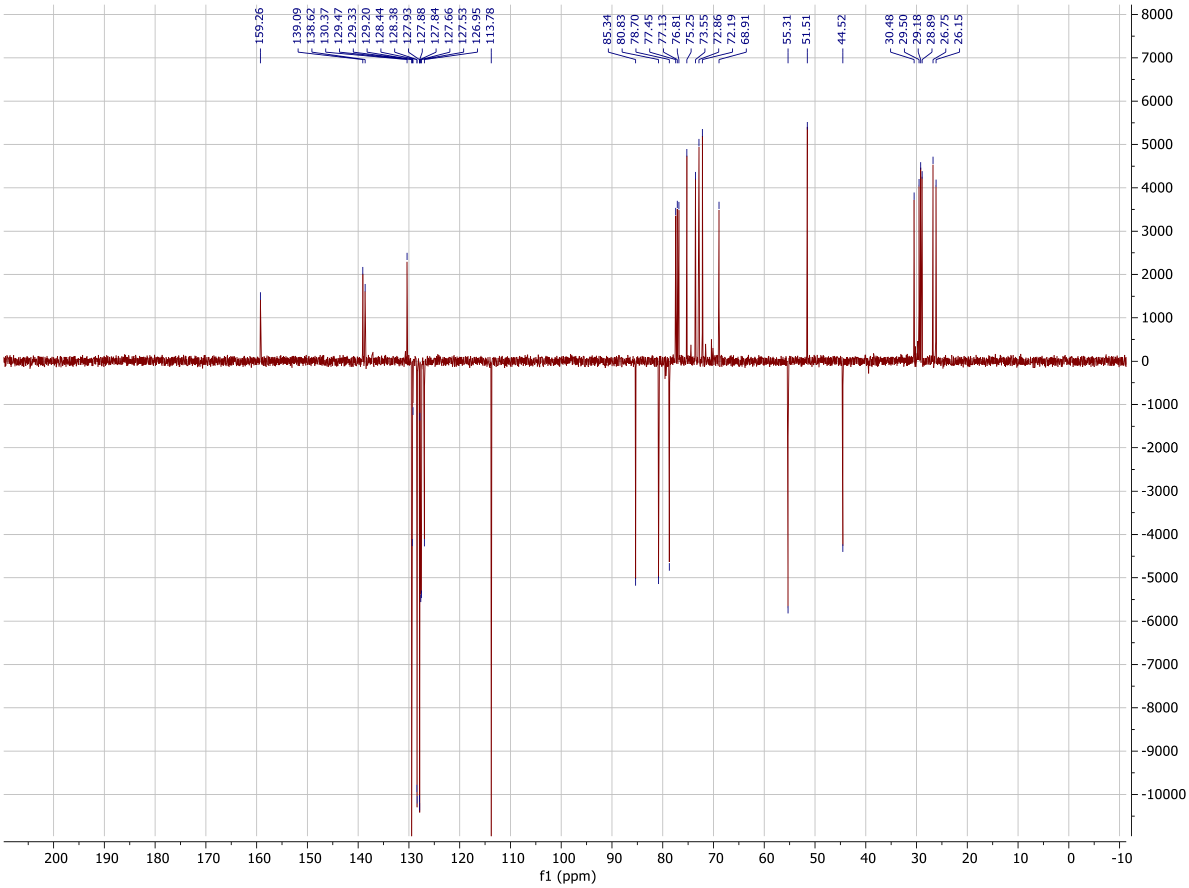

## 2,3-di-*O*-benzyl-4-(8-azidooctyl)-cyclophellitol alkene (24b)

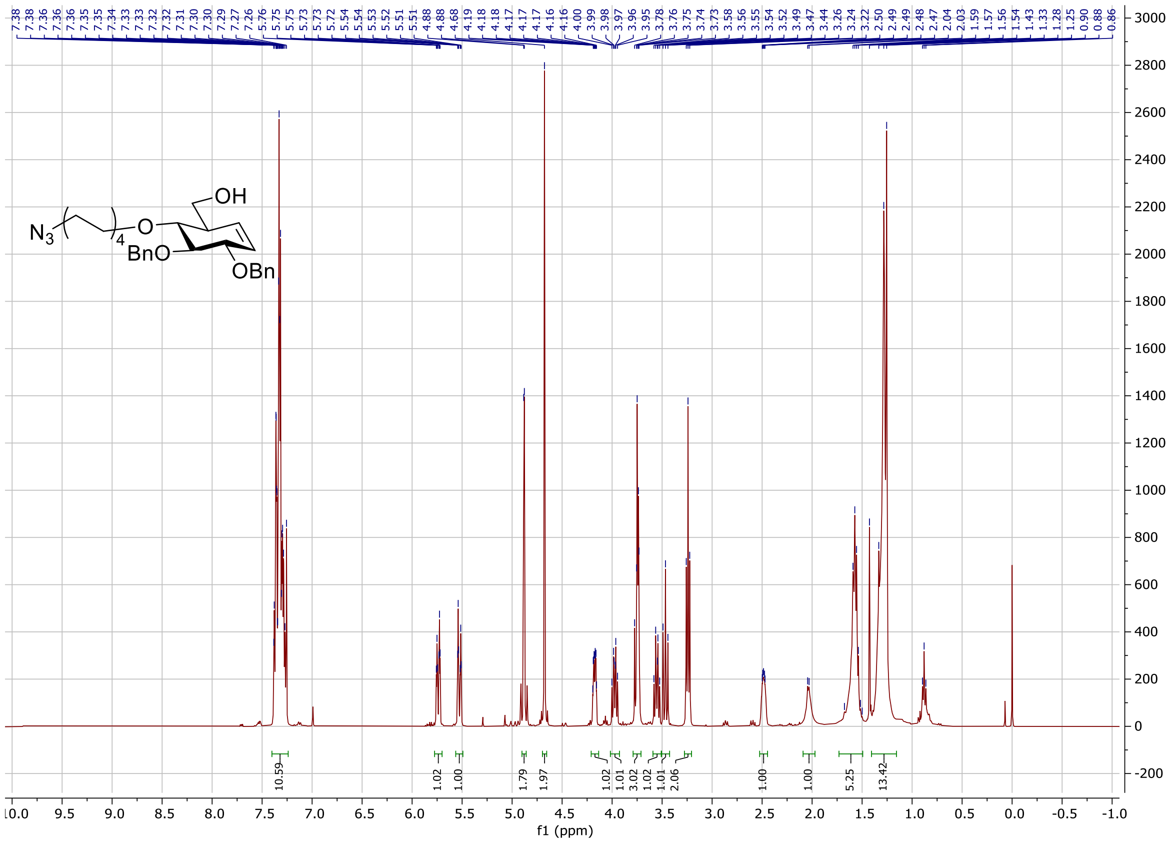


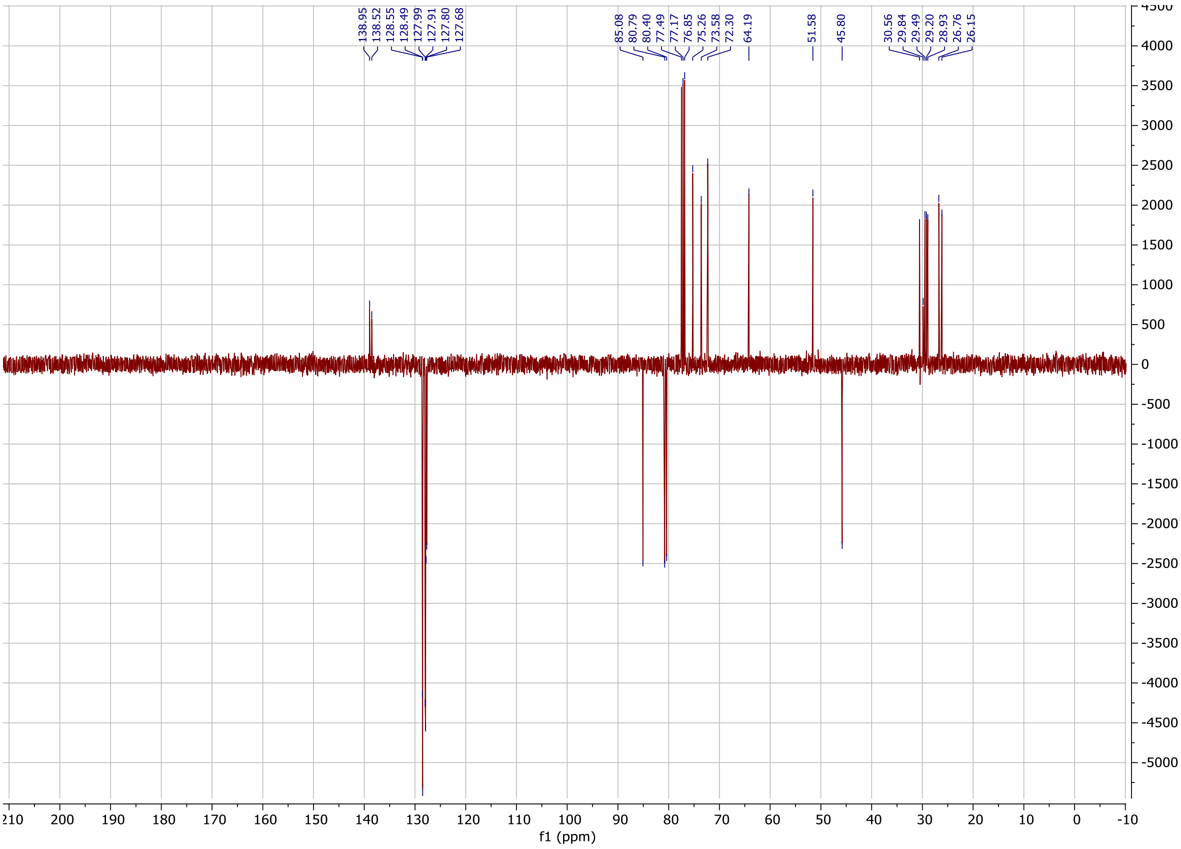

## 2,3-di-*O*-benzyl-4-(8-azidooctyl)-cyclophellitol (25a)

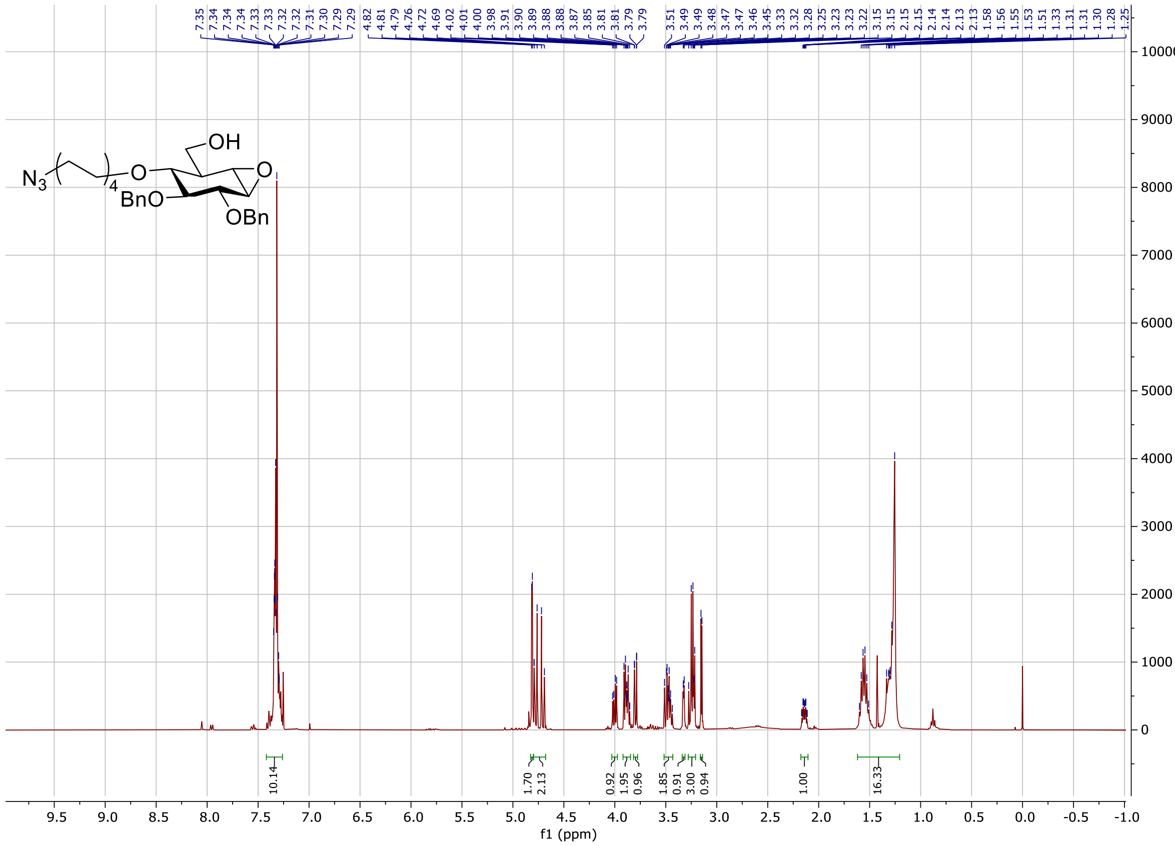


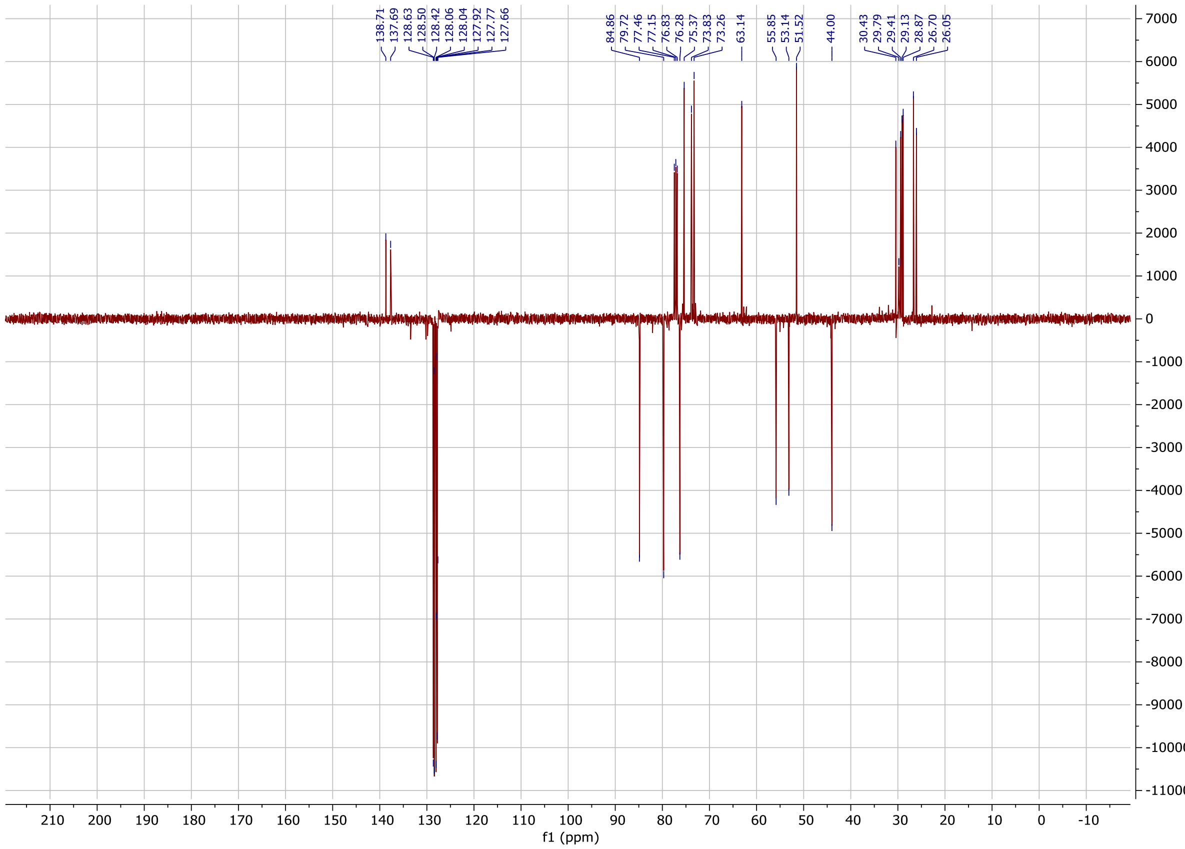

## 2,3-di-*O*-benzyl-4-(8-azidooctyl)-glucurono-cyclophellitol (25b)

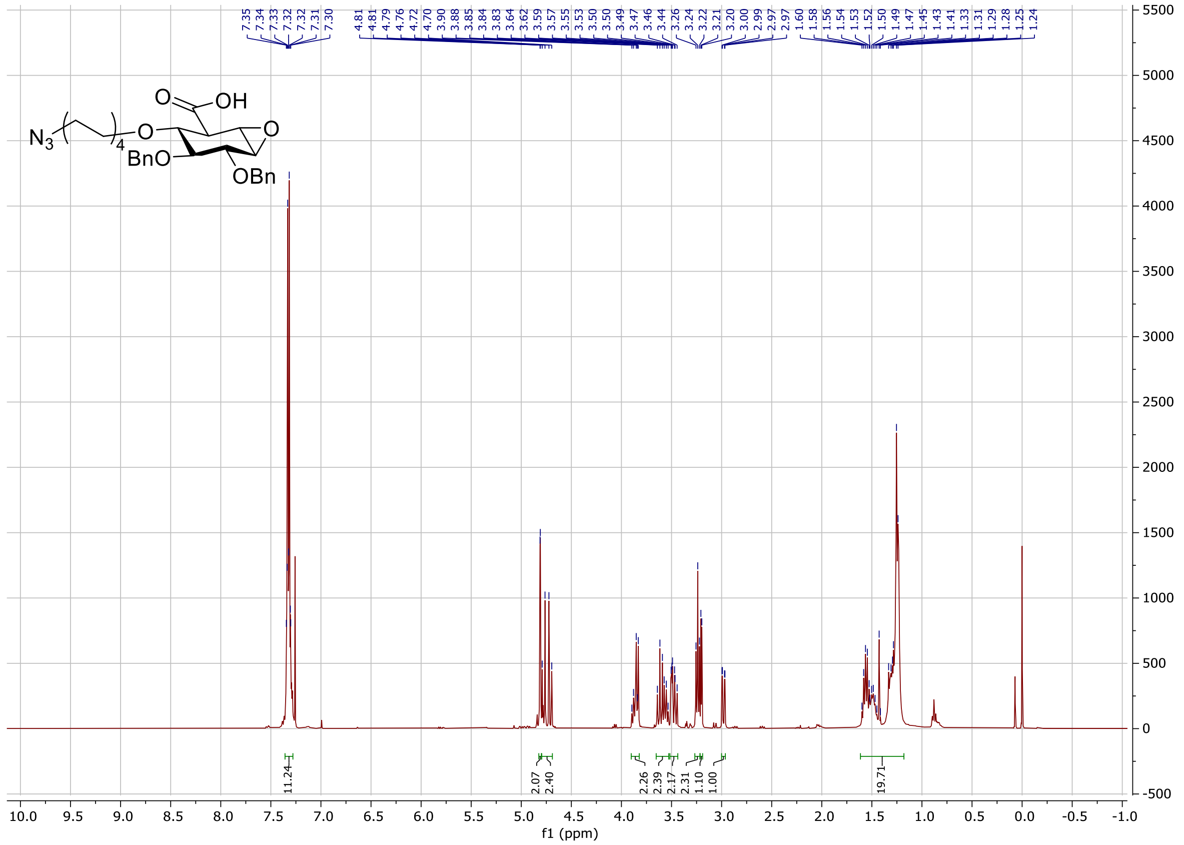


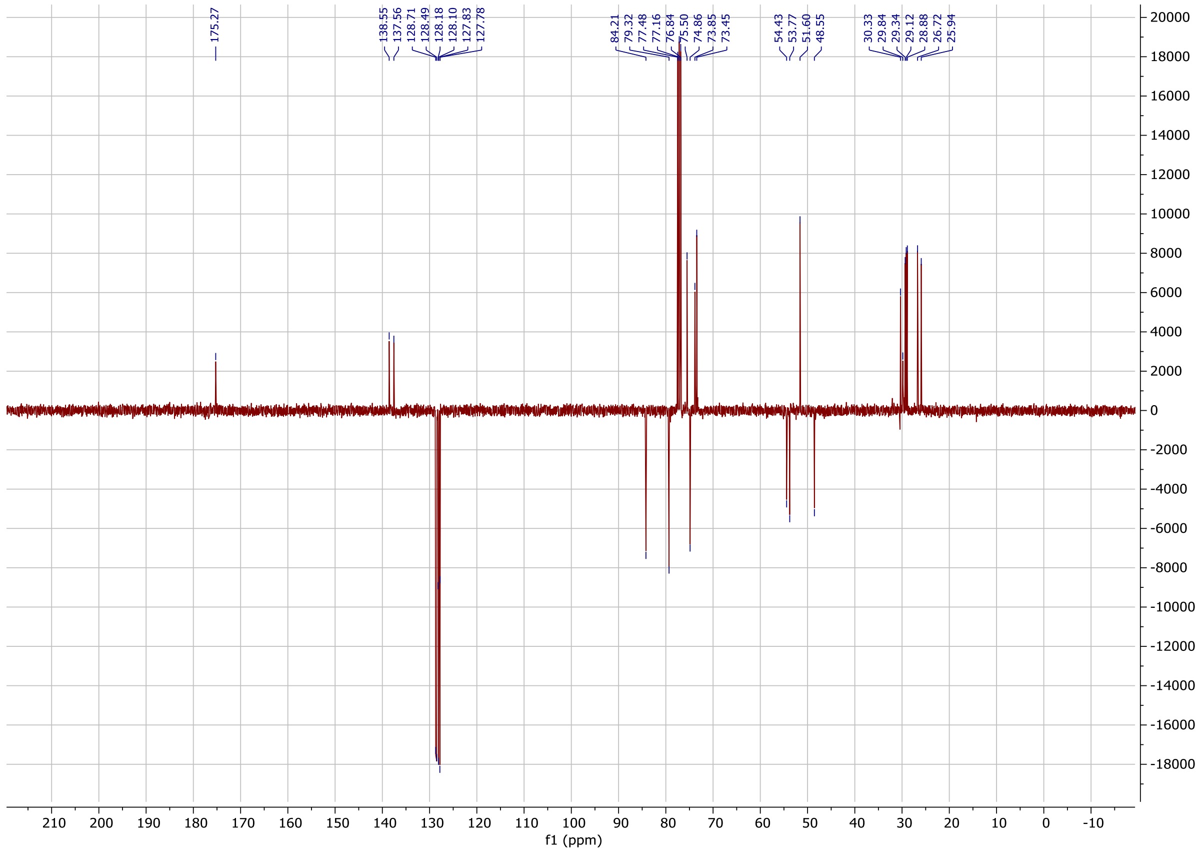

# 4-(8-aminooctyl)-glucurono-cyclophellitol (26)

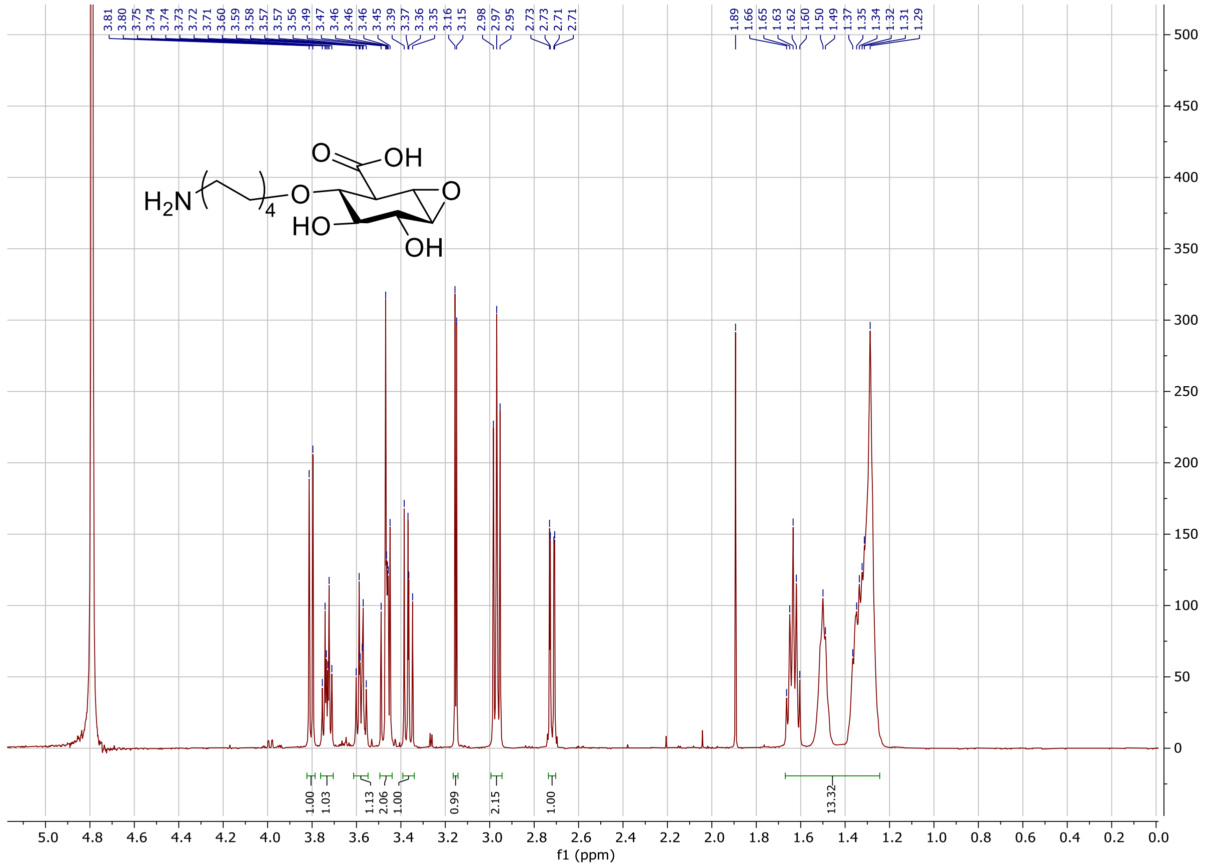


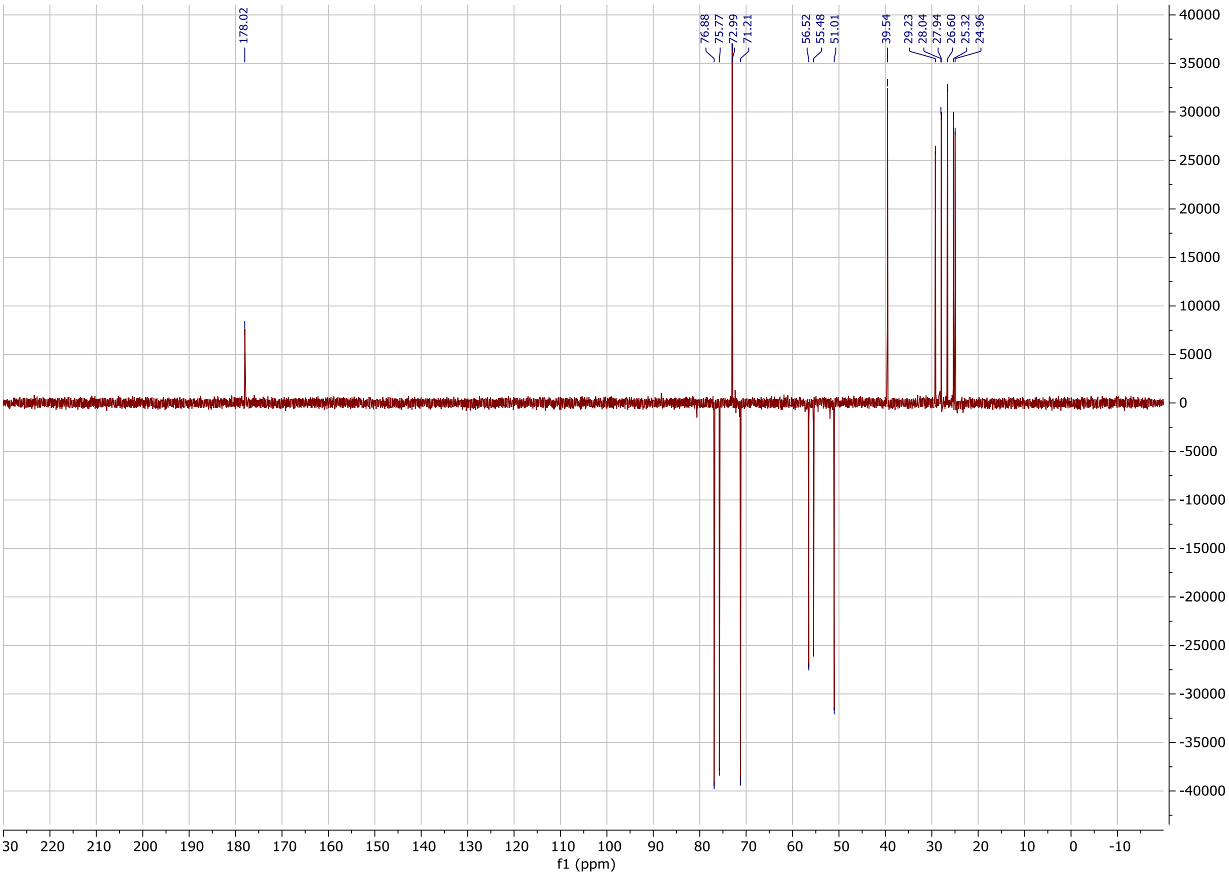

## 4-(8-Cy5-aminooctyl)-glucurono-cyclophellitol (8)

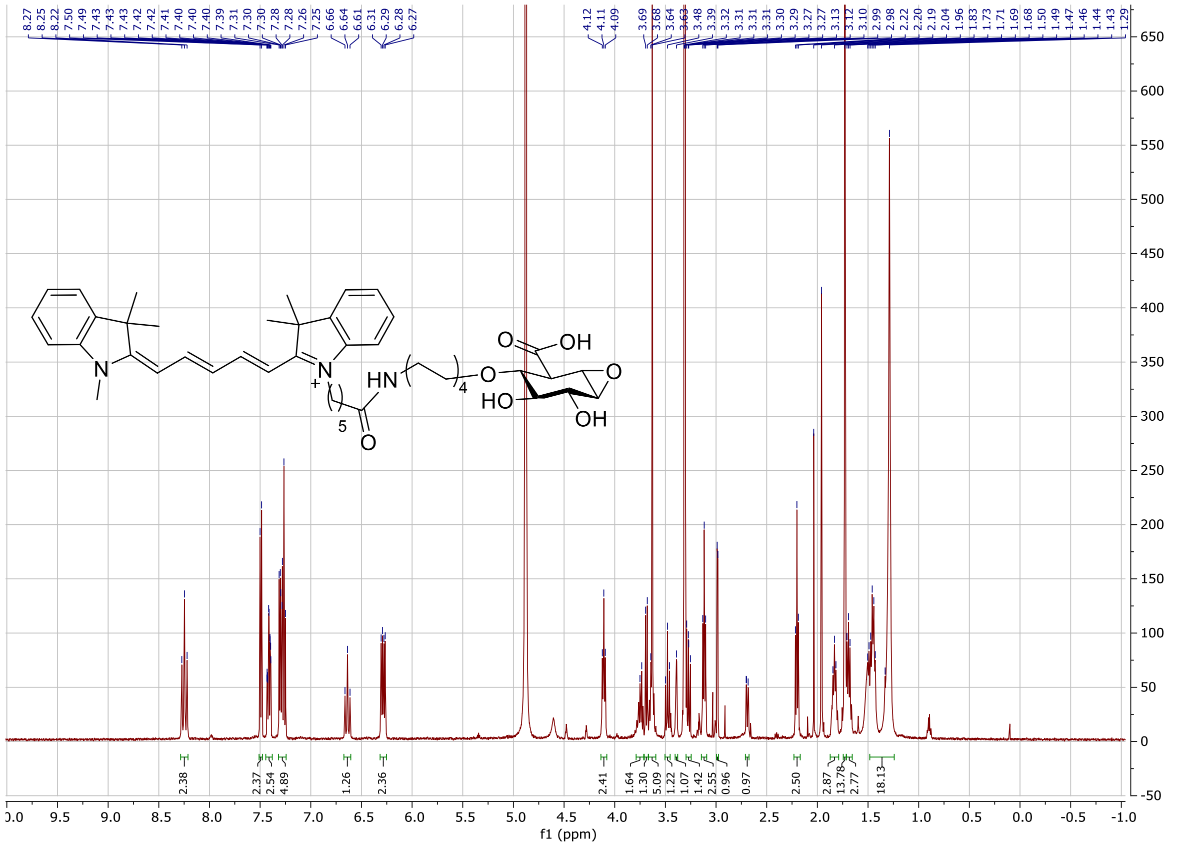


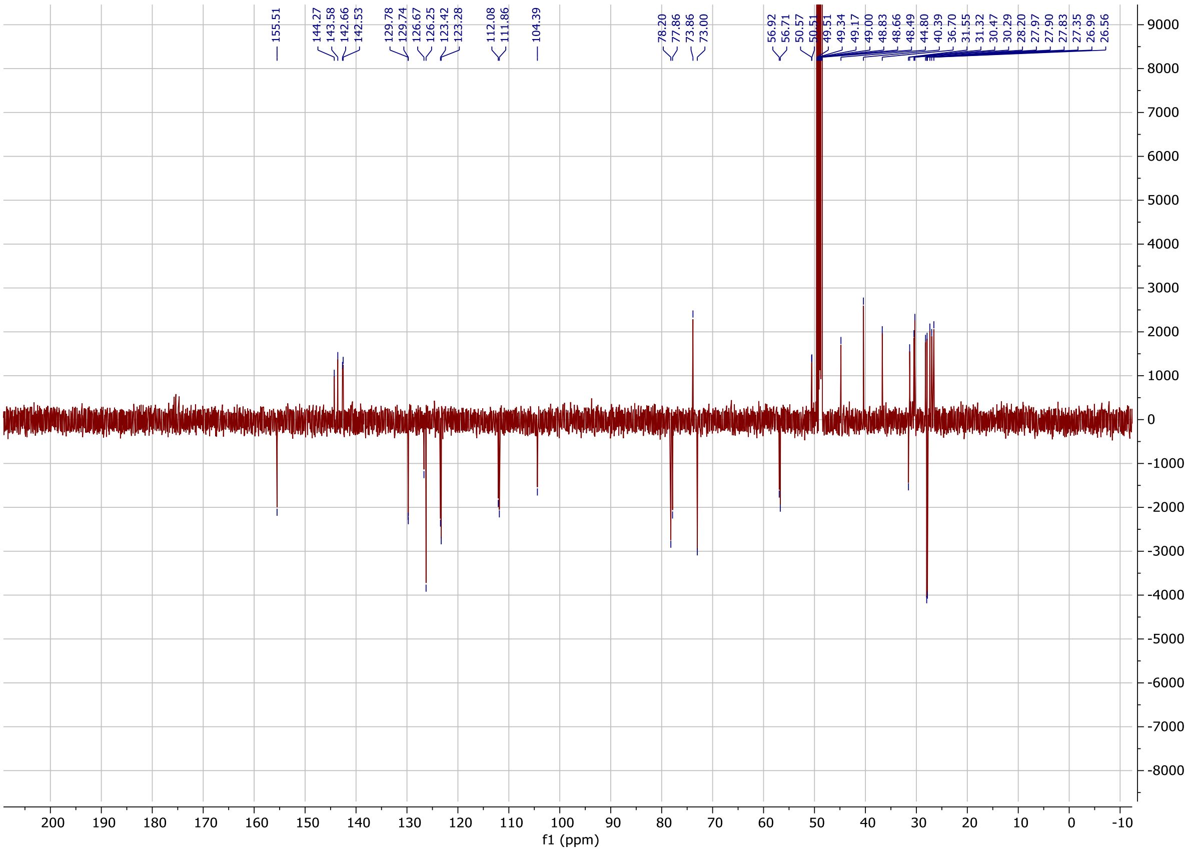

## 8-Benzyloxycarbonylamino-octan-1-ol (28a)

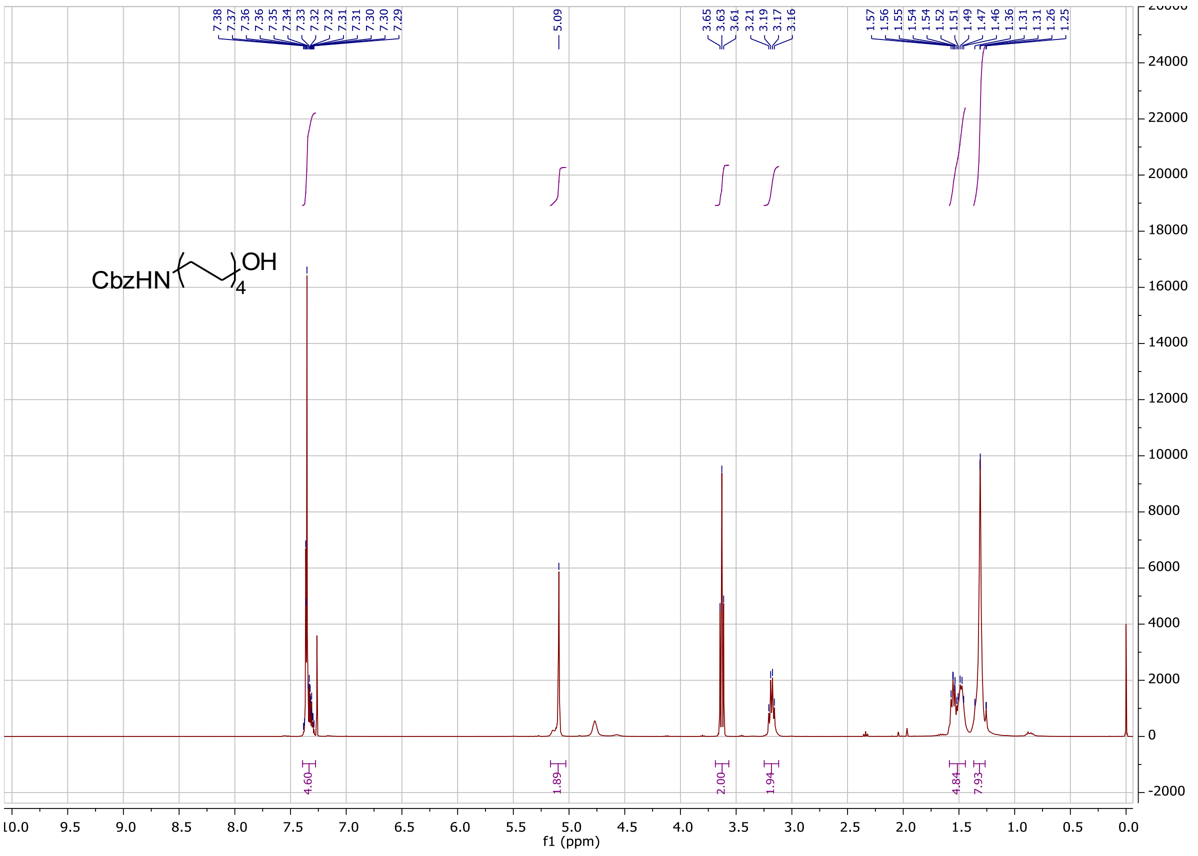


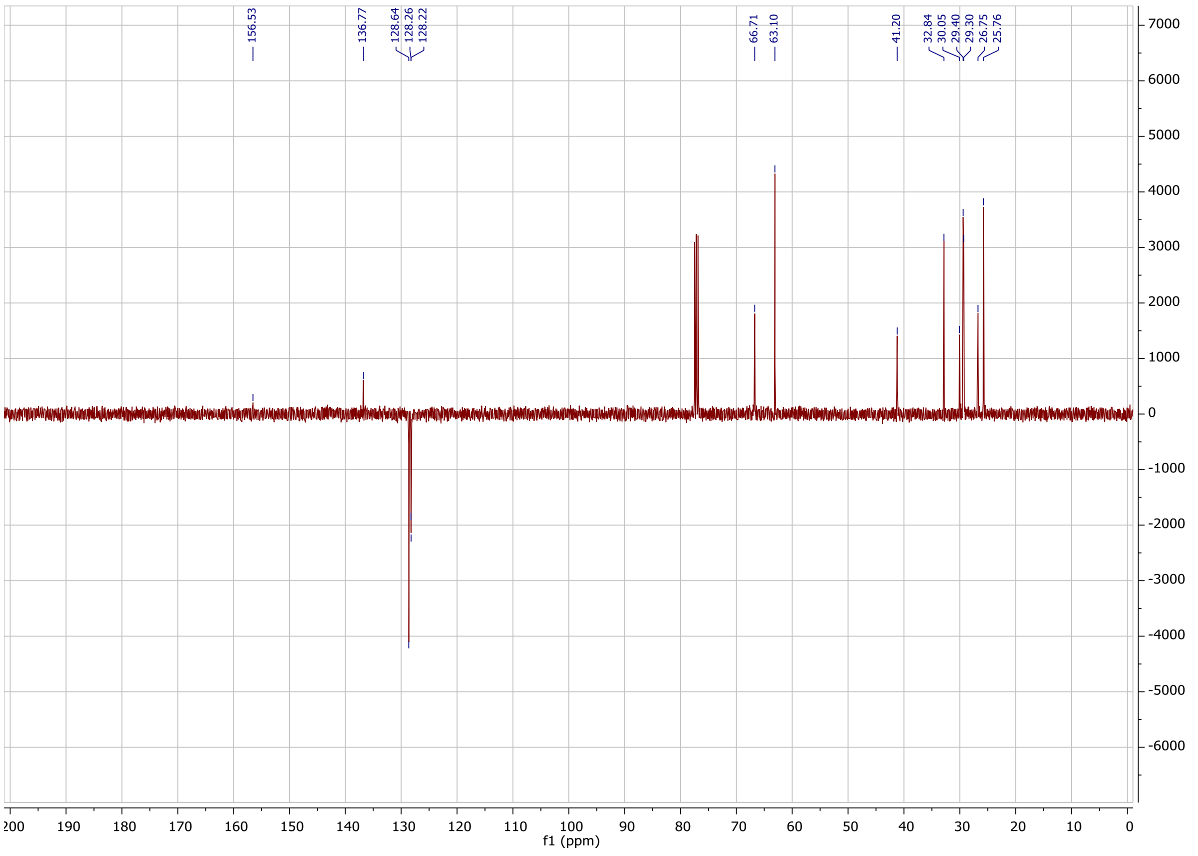

## 8-Benzyloxycarbonylamino-1-trityloxy octane (28b)

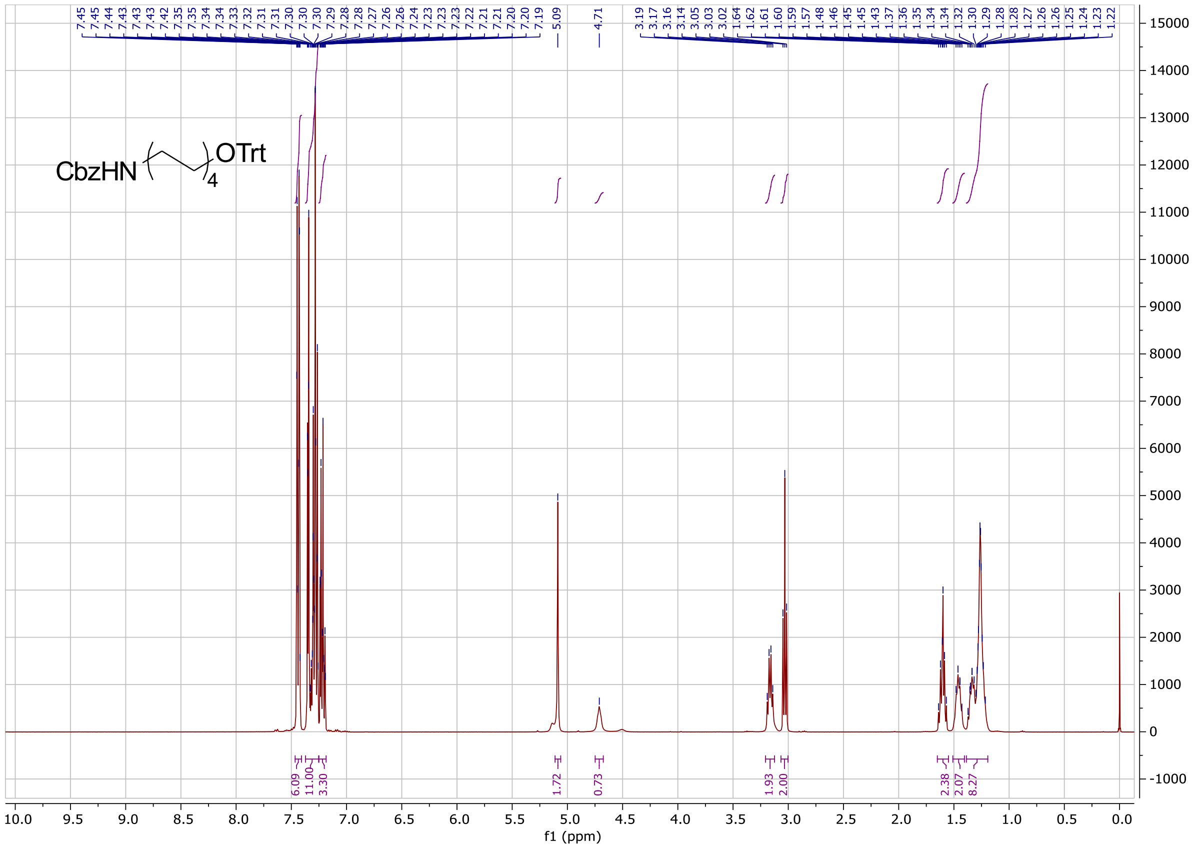

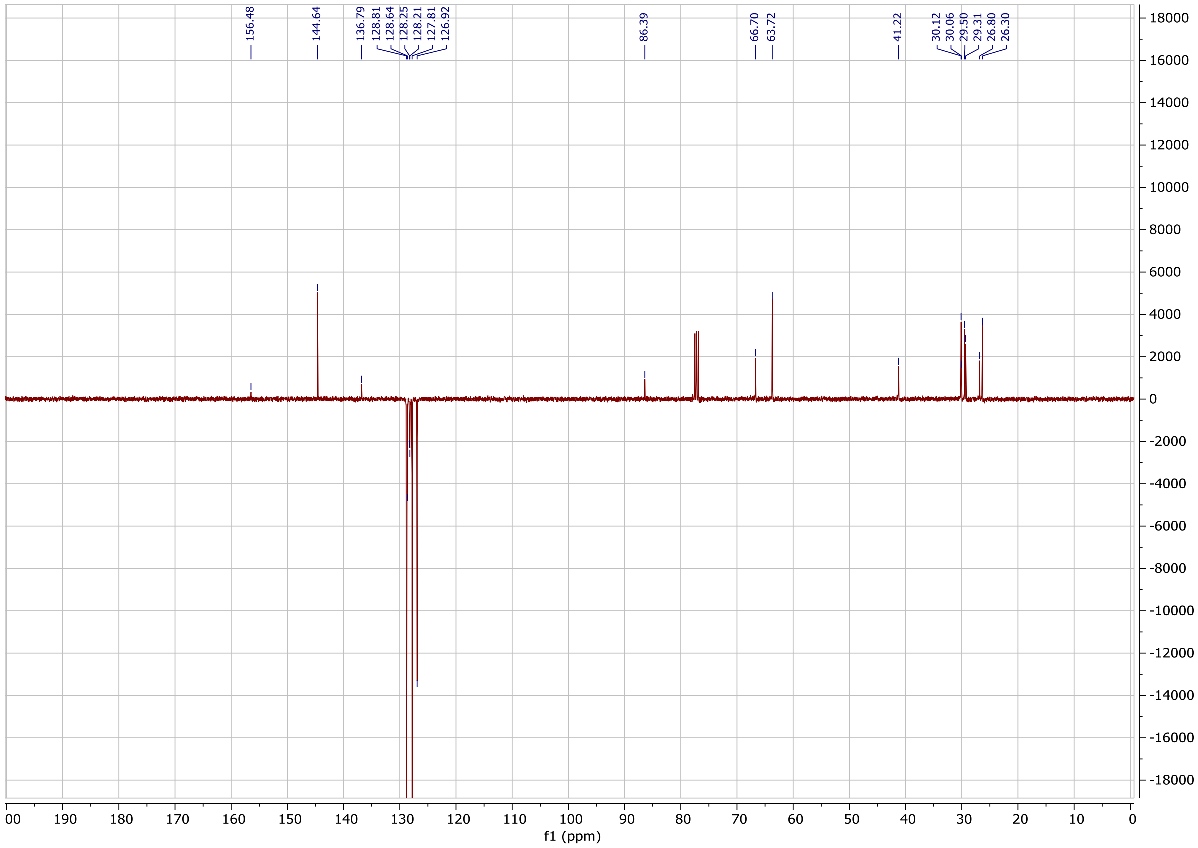

## 8-*N*-benzyl-(benzyloxycarbonyl)-1-trityloxy octane (29b)

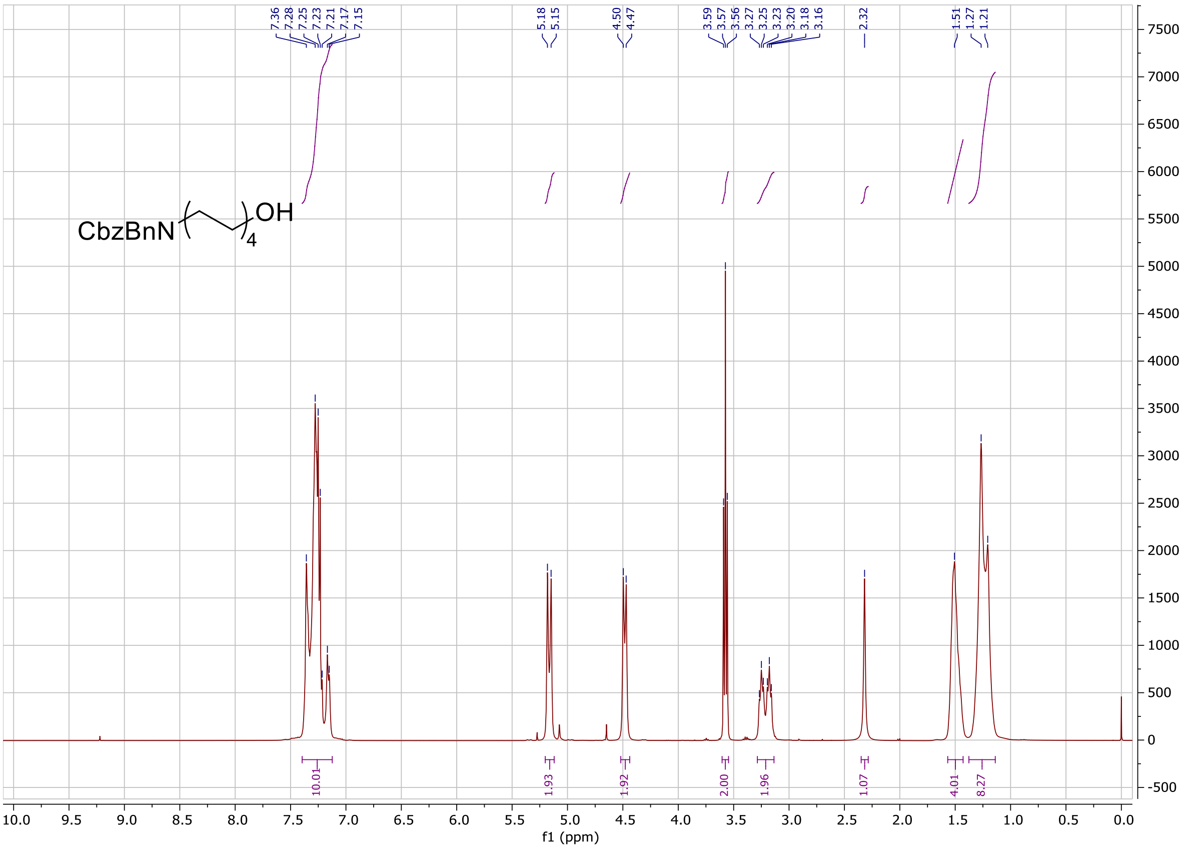

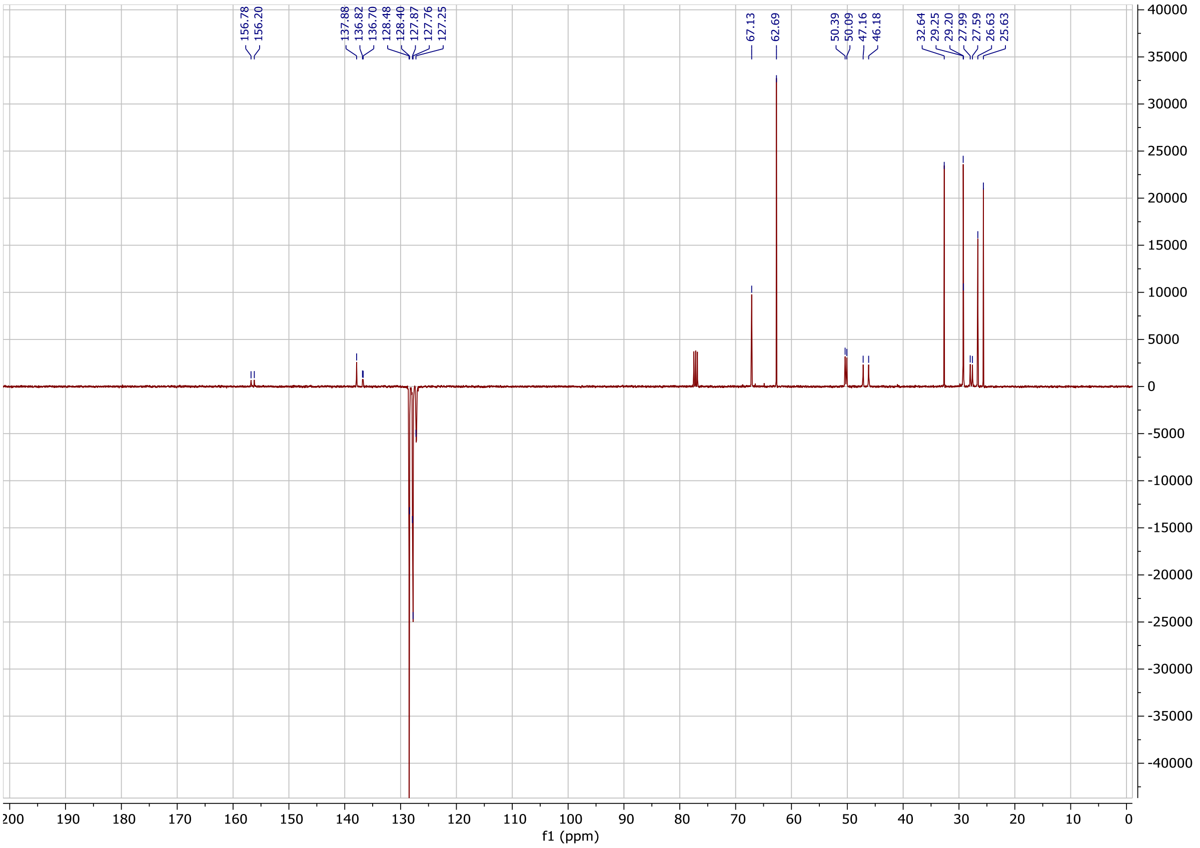

**8-*N*-benzyl(benzyloxycarbonyl)-1-iodo-octane(30)**

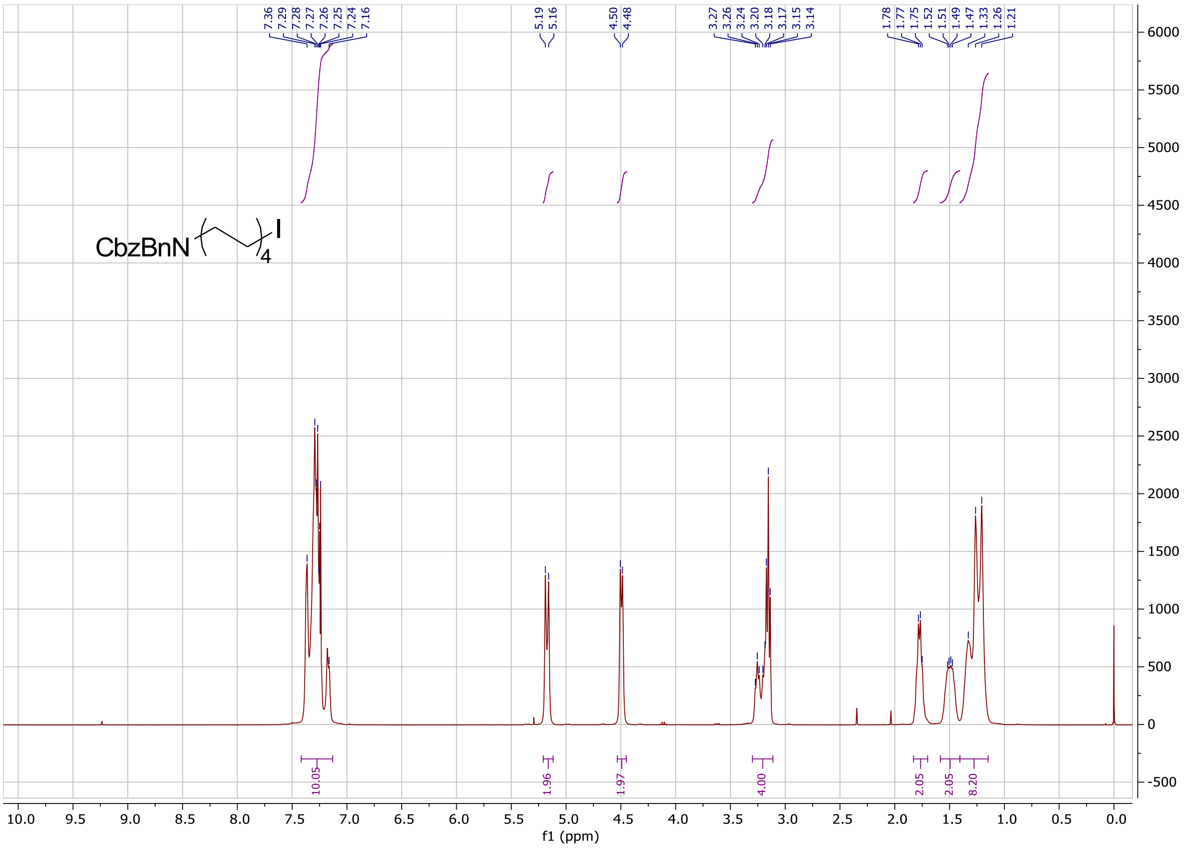


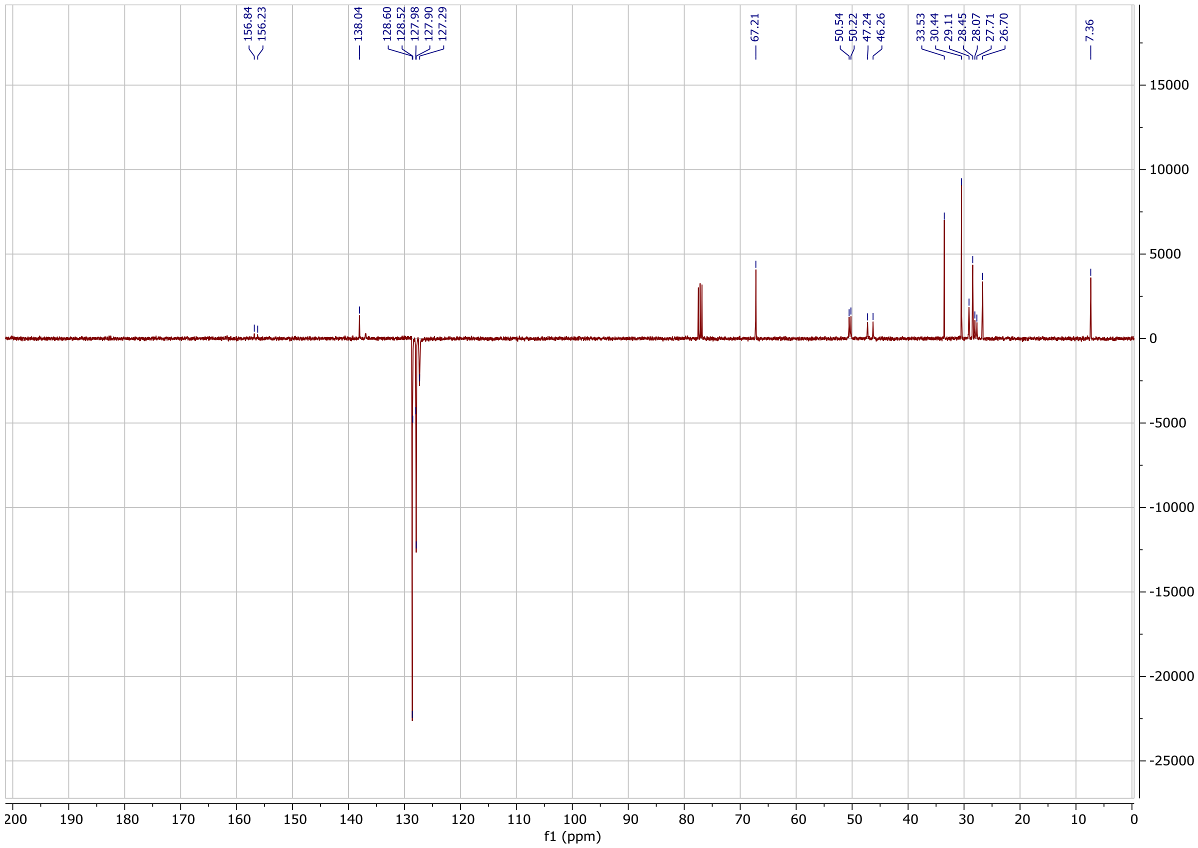

# Phenyl 2-azido-2-deoxy-3-O-benzyl-6-O-(2-Naphthylmethyl)-1-thio-α-D-glucopyranoside (31b)

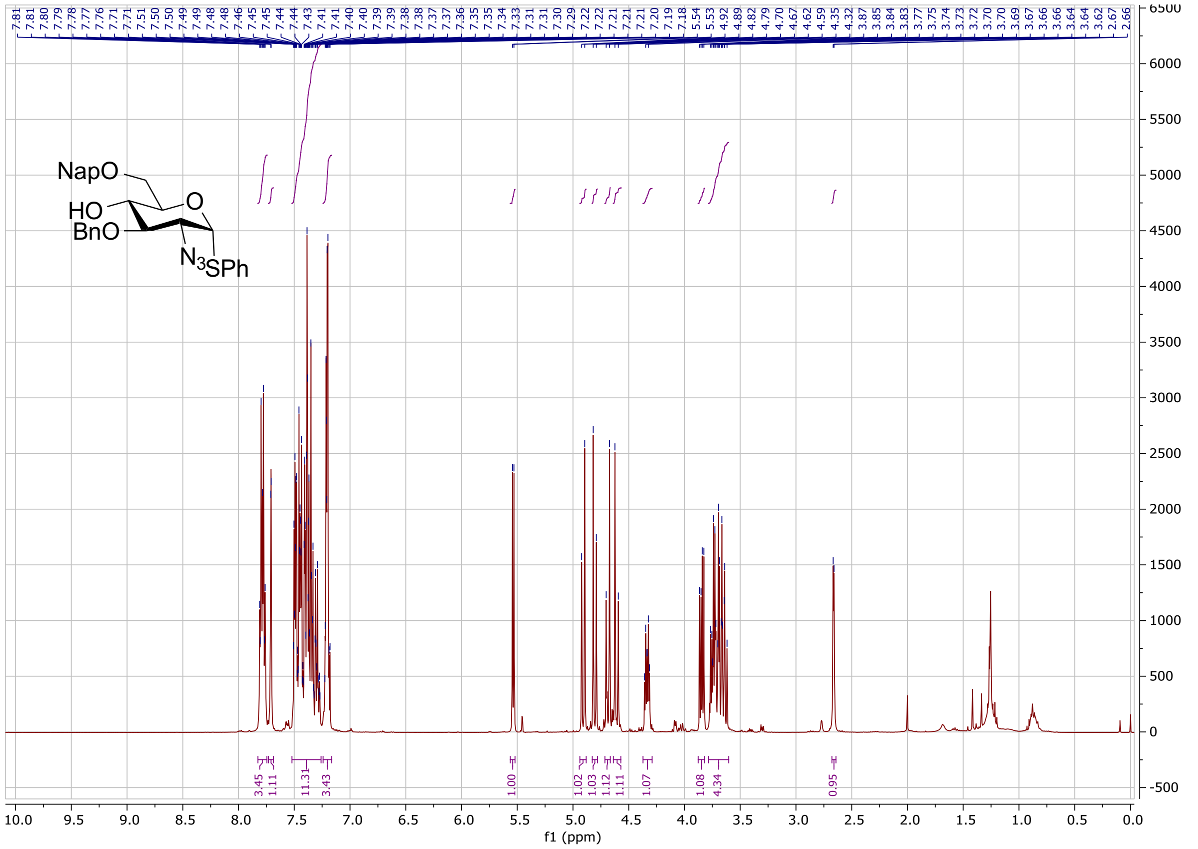


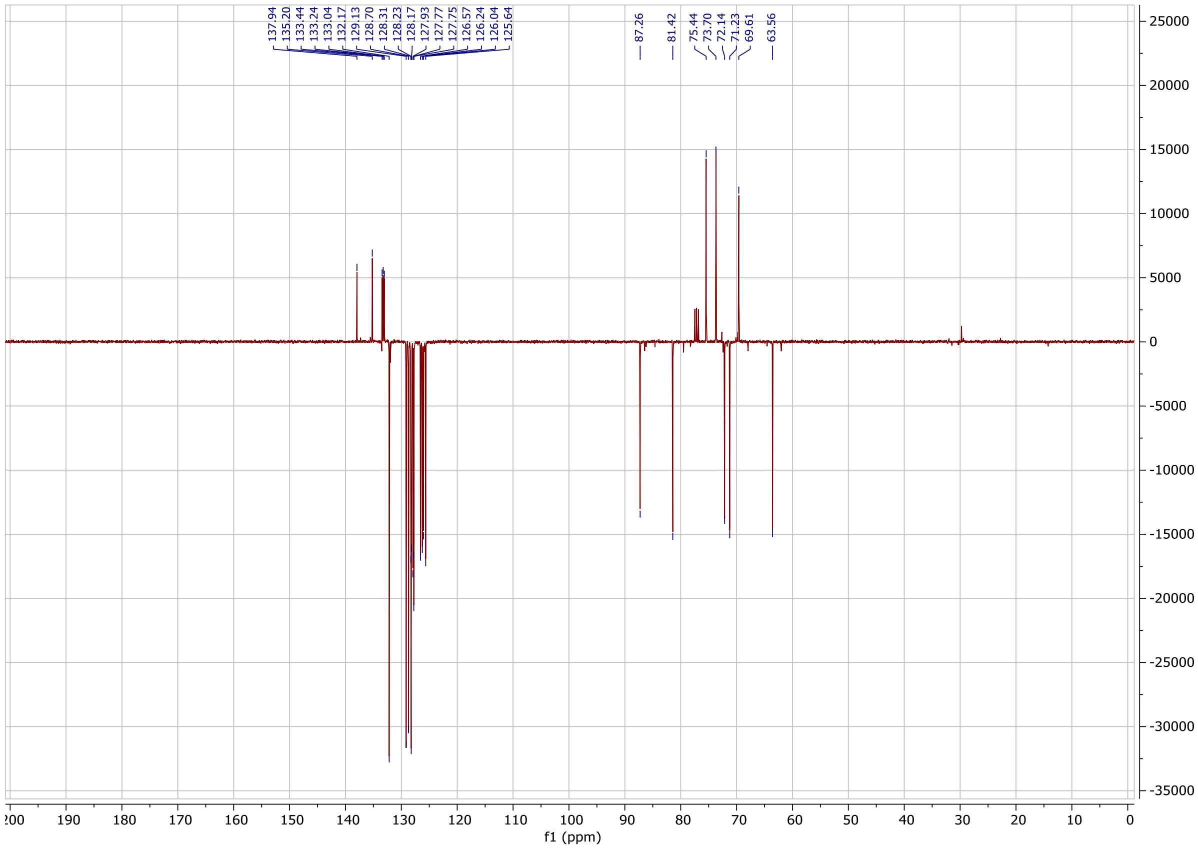

## Phenyl 2-azido-2-deoxy-3-*O*-benzyl-4-*O*-(8-*N*-benzyl(benzyloxycarbonyl)-1-octyl)-6-*O*-(2-Naphthylmethyl)-1-thio-α-D-glucopyranoside (32a)

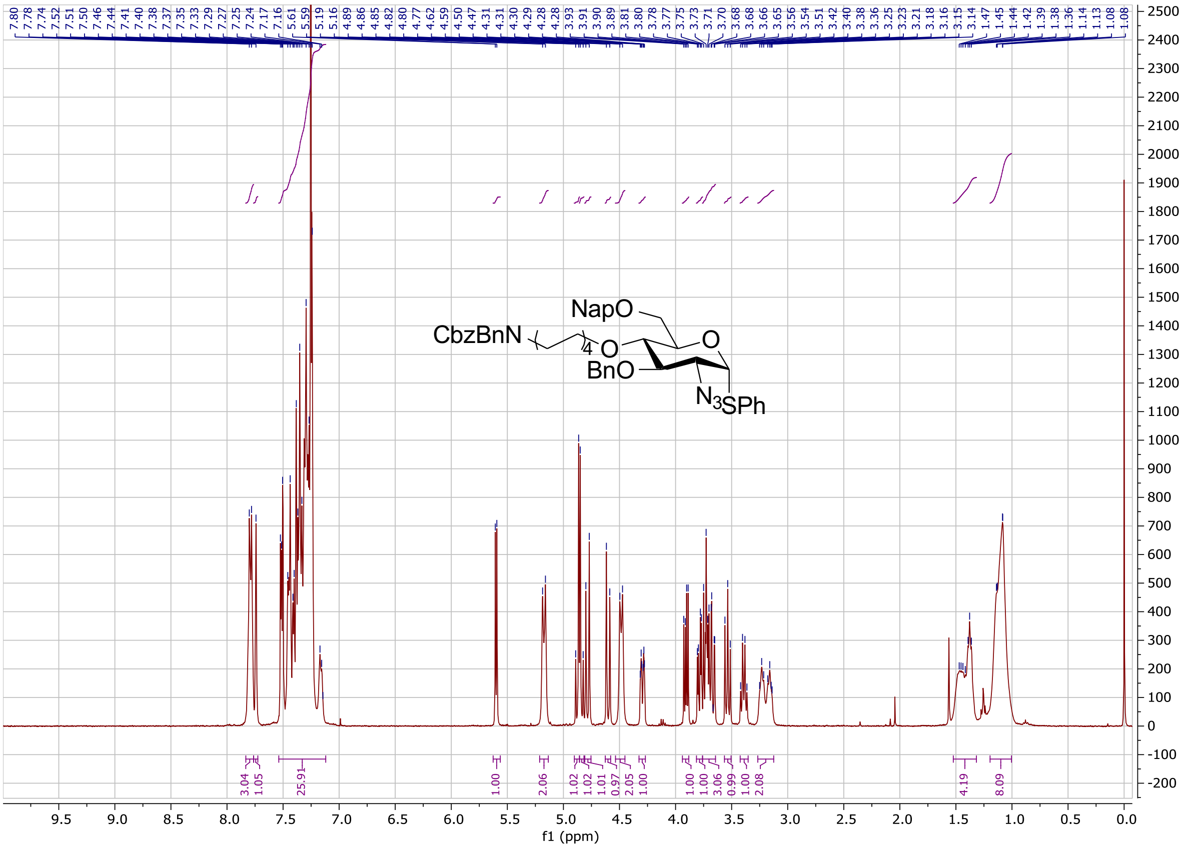


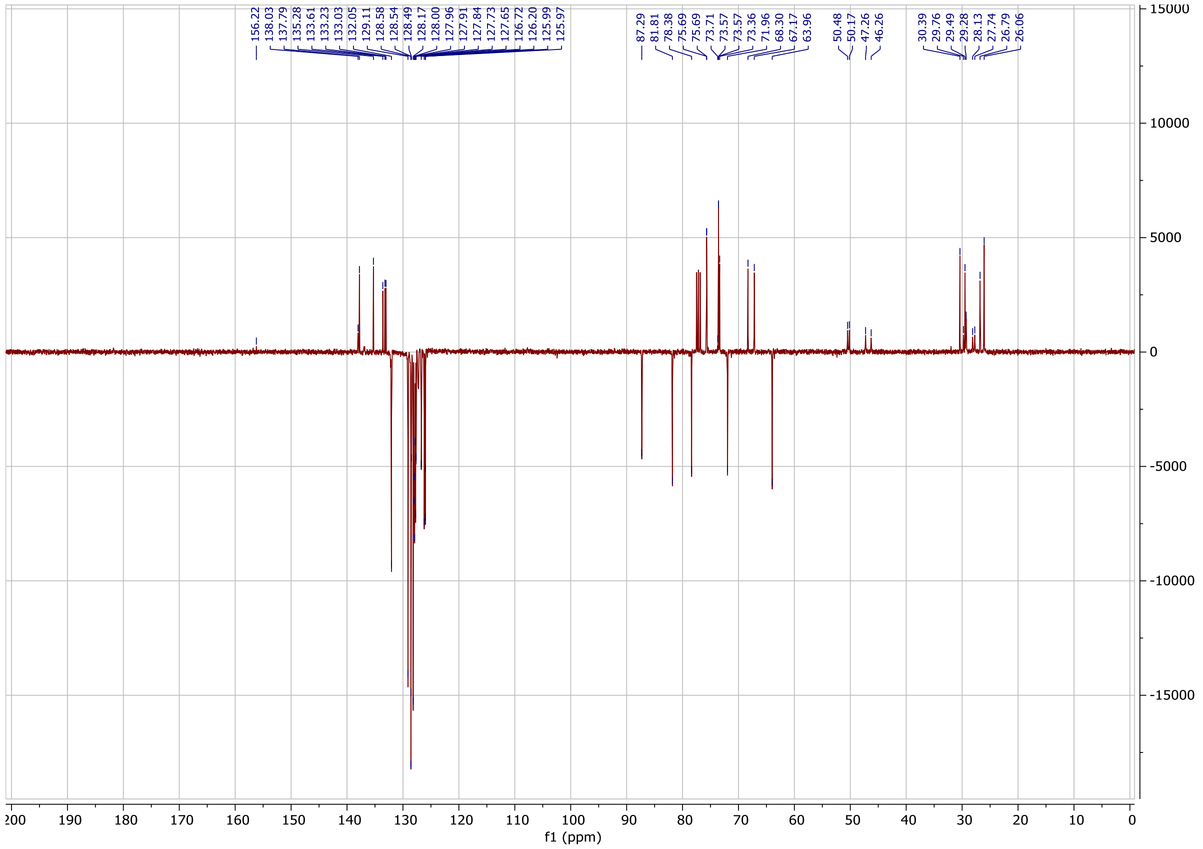

**Phenyl 2-azido-2-deoxy-3-*O*-benzyl-4-*O*-(8-*N*-benzyl(benzyloxycarbonyl)-1-octyl)-1-thio-α-D-glucopyranoside (32b)**

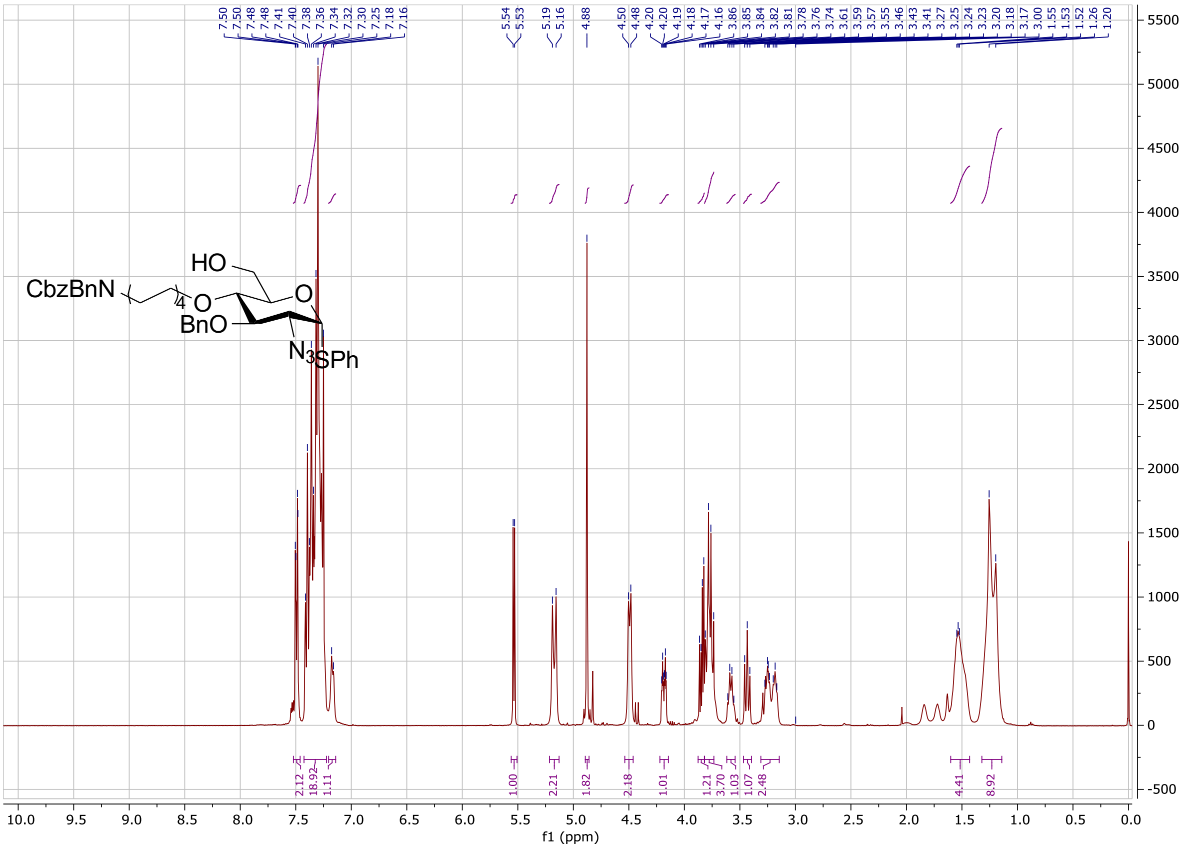


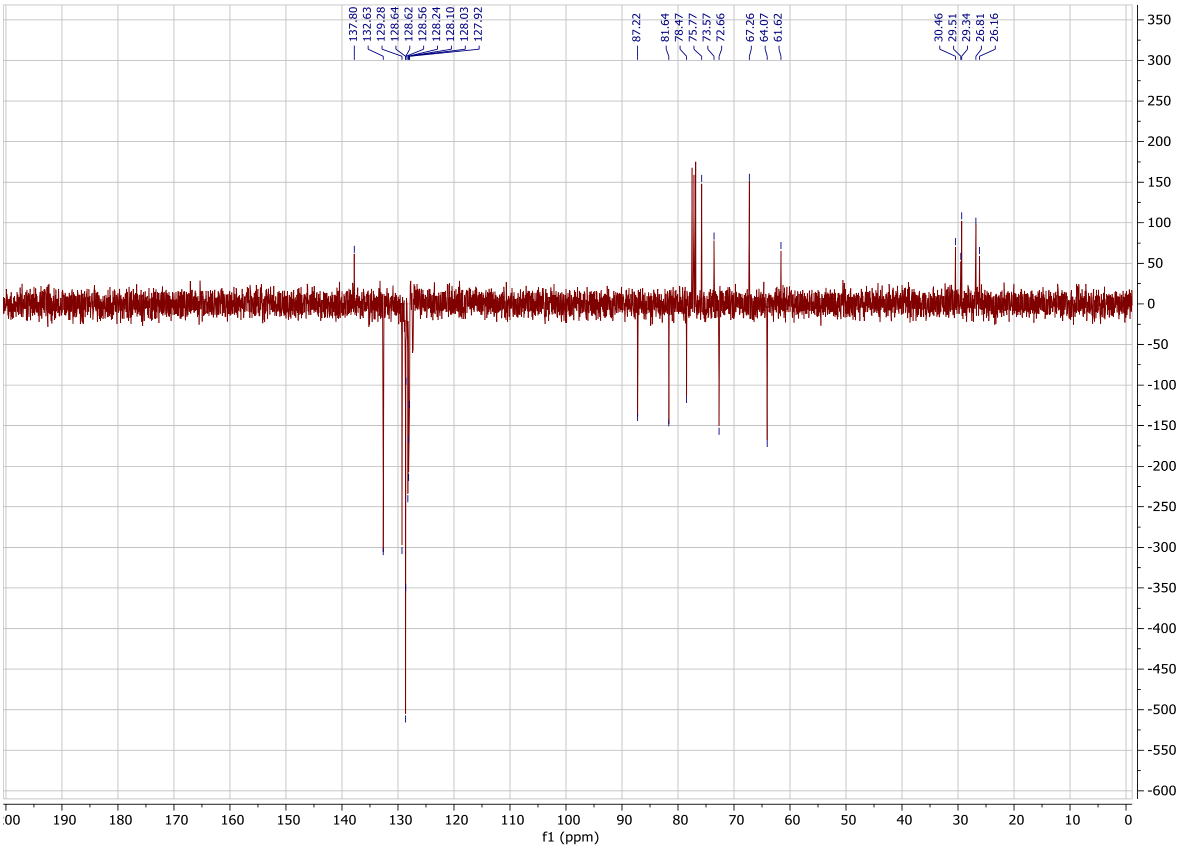

**Phenyl 2-azido-2-deoxy-3-*O*-benzyl-4-*O*-(8-*N*-benzyl-(benzyloxycarbonyl)-1-octyl)-6-*O*-tert-butyldiphenylsilyl-1-thio-α-D-glucopyranoside (32c)**

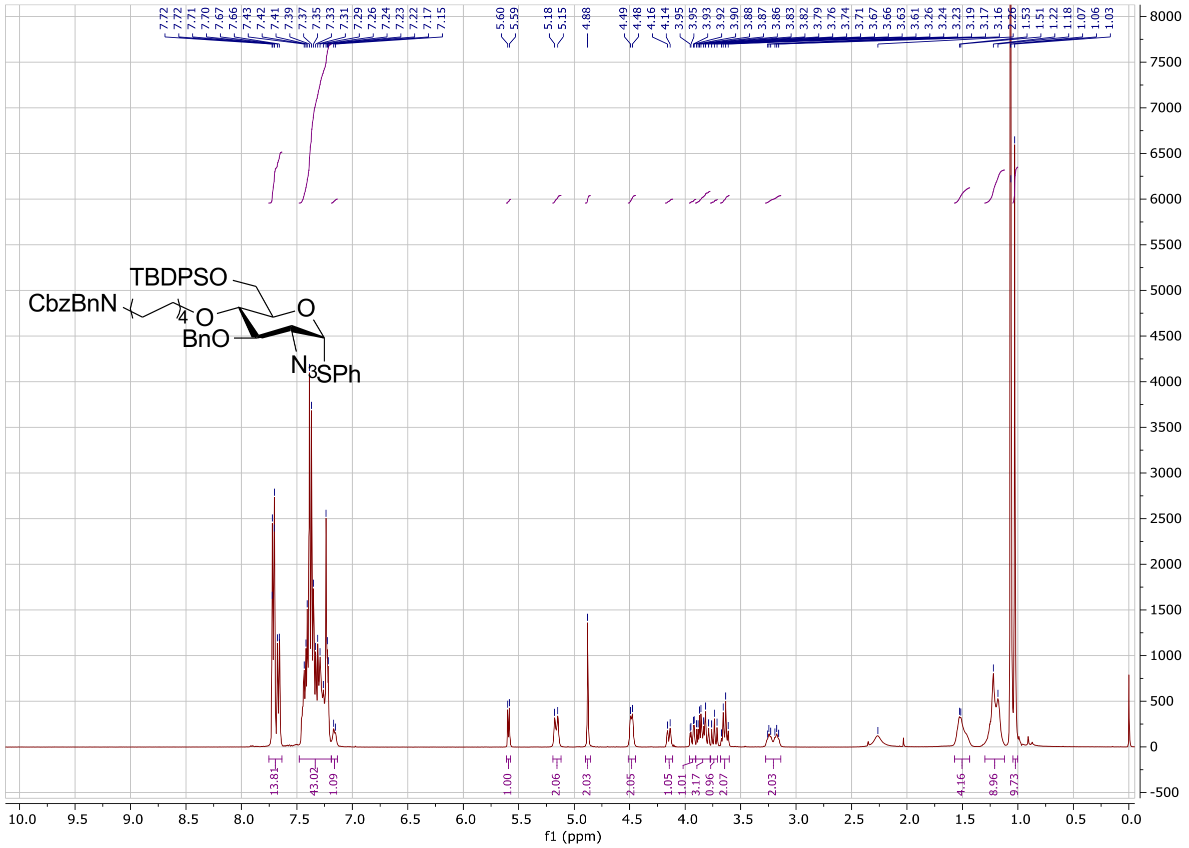


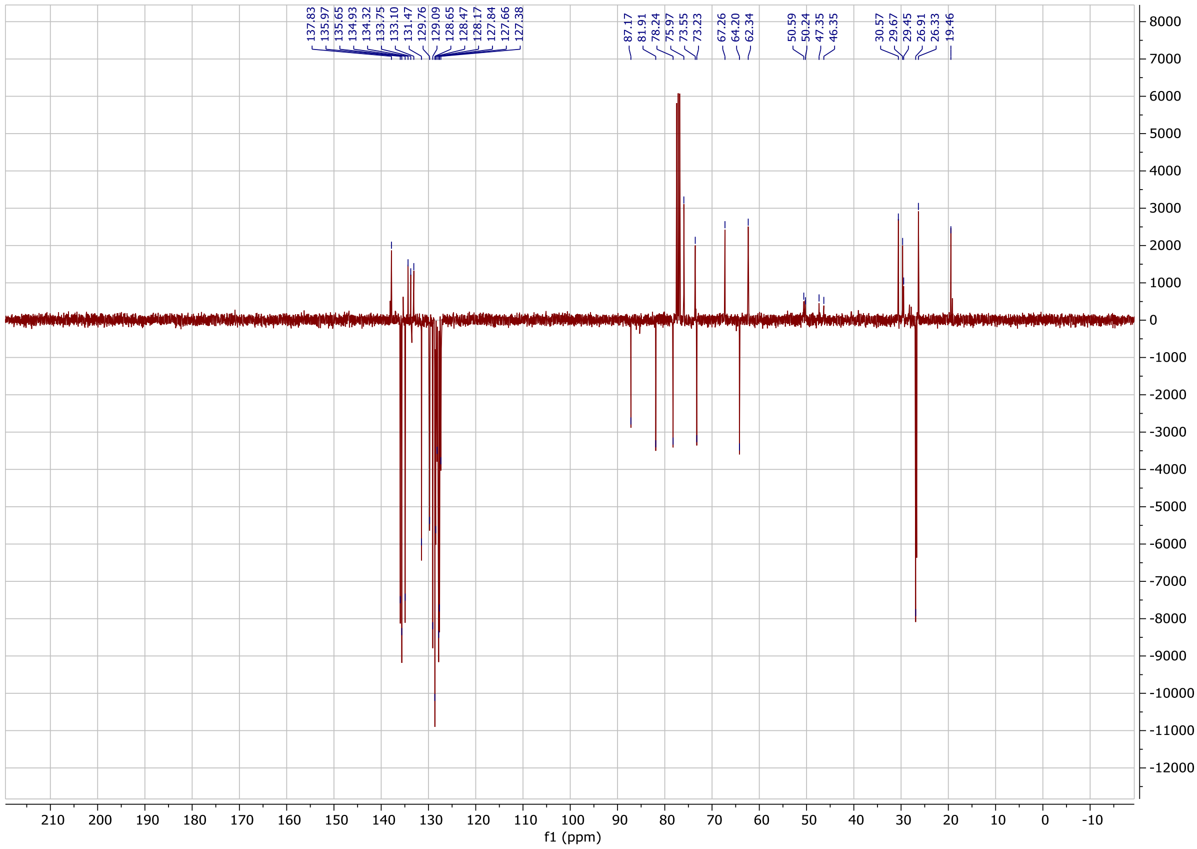

**2-azido-2-deoxy-3-*O*-benzyl-4-*O*-(8-*N*-benzyl(benzyloxycarbonyl)-1-octyl)-6-*O*-tert-butyldiphenylsilyl-D-glucopyranose (33a)**

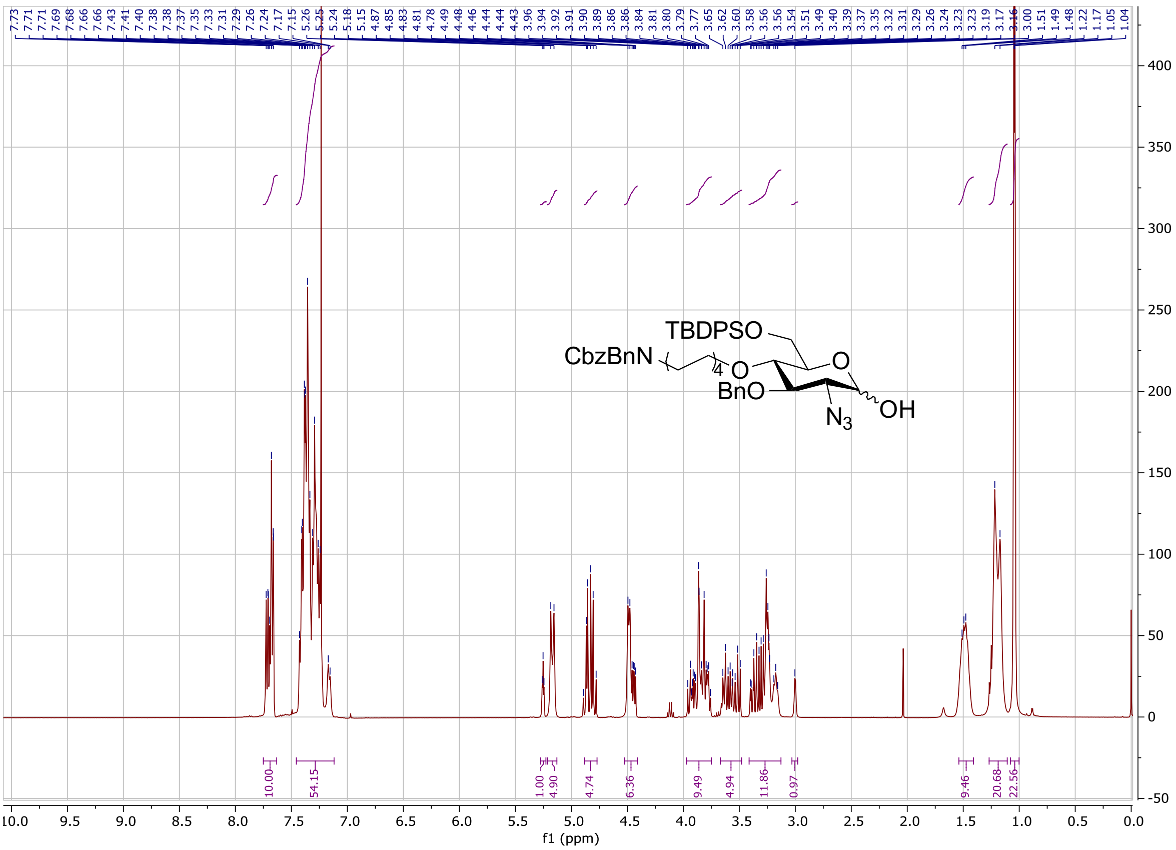

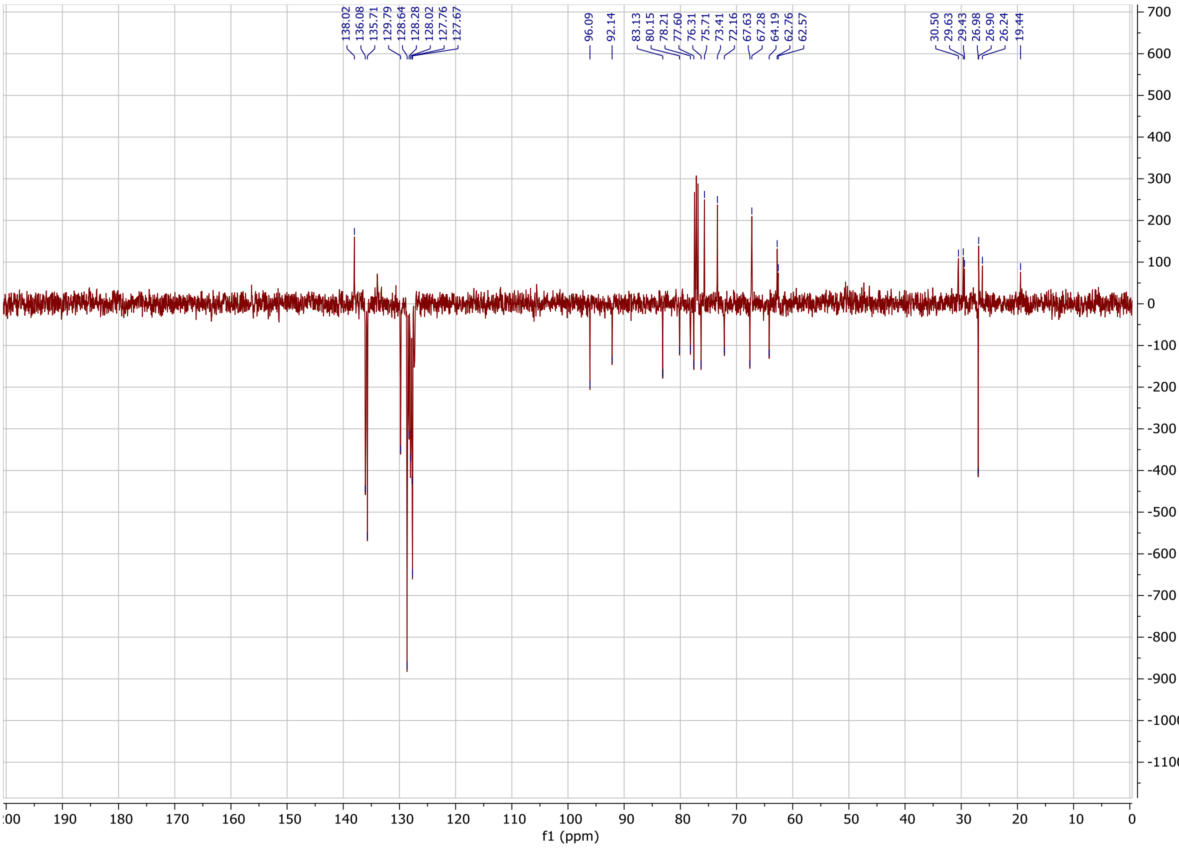

**2-azido-2-deoxy-3-*O*-benzyl-4-*O*-(8-*N*-benzyl(benzyloxycarbonyl)-1-octyl)-6-*O*-tert-butyldiphenylsilyl-D-glucopyranosyl N-phenyltrifluoroacetimidate (33b)**

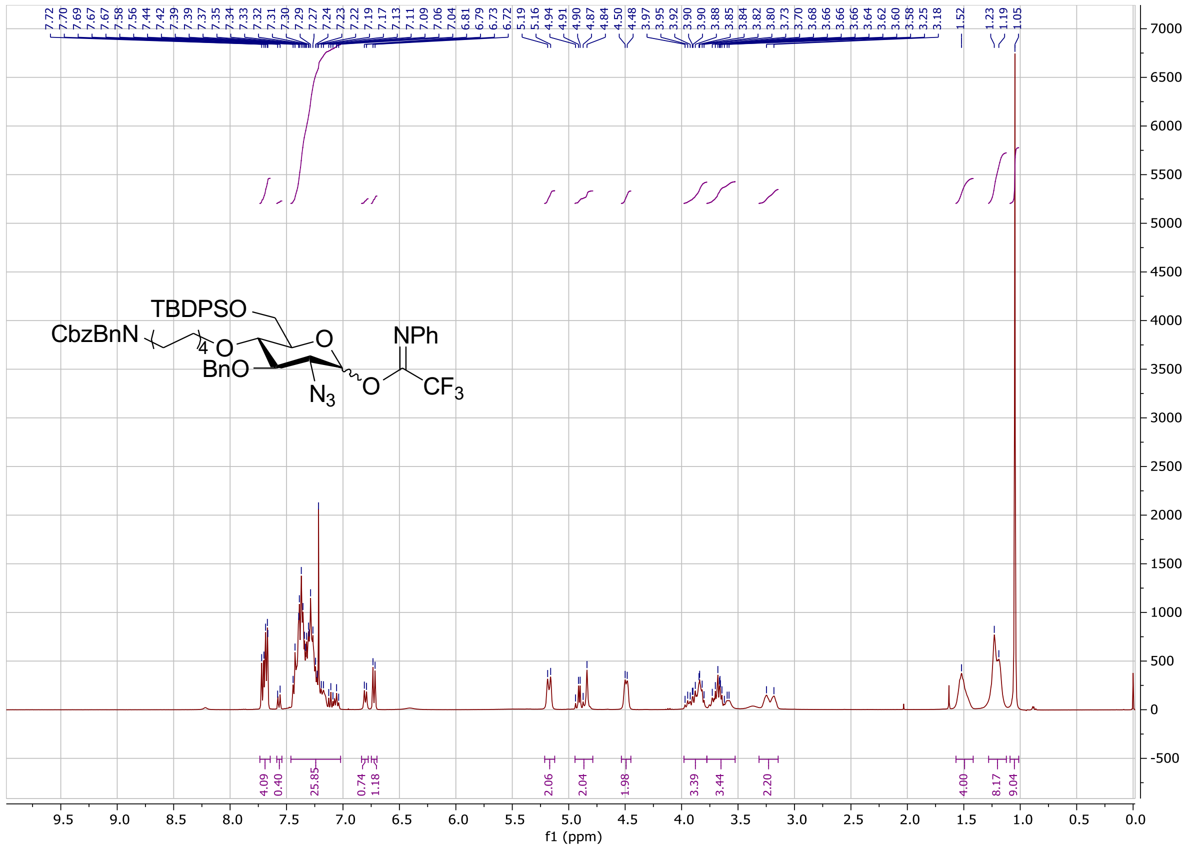

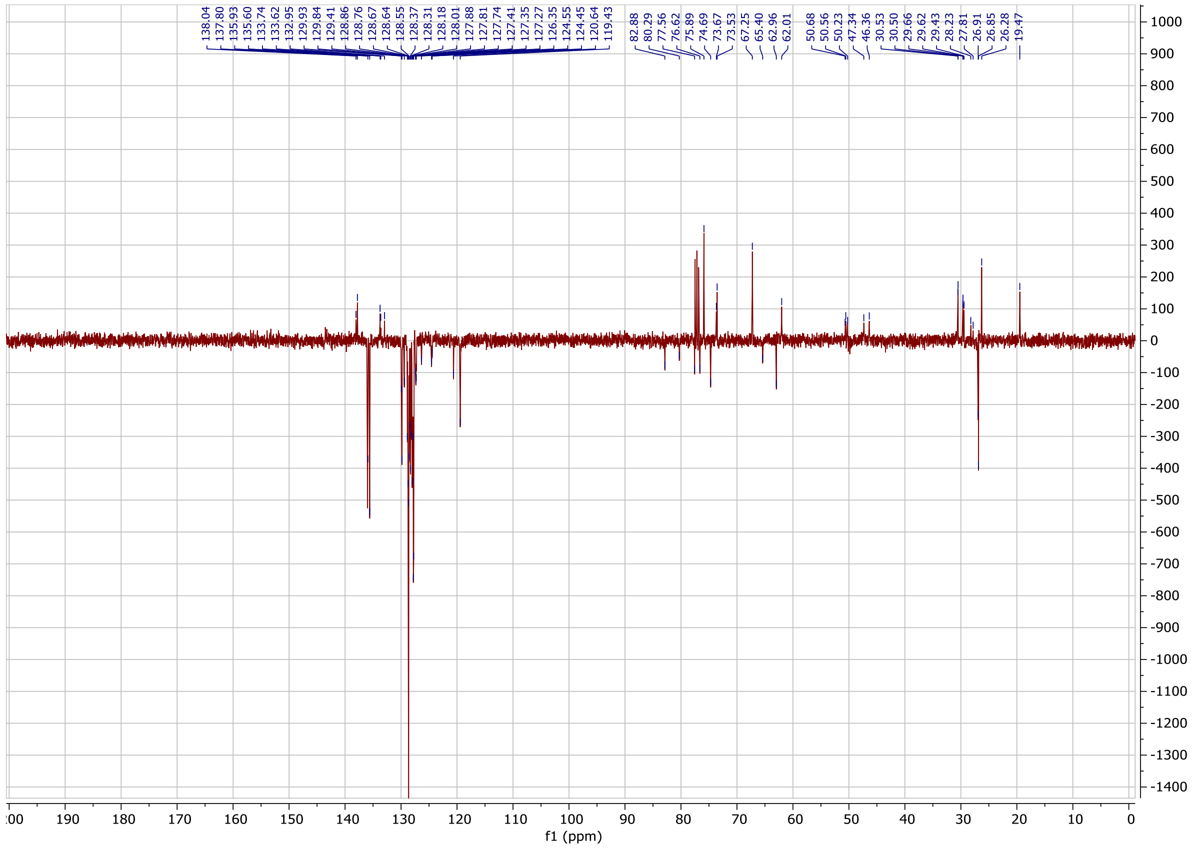

**2,3-di-*O*-benzyl-4-*O*-(2-azido-2-deoxy-3-*O*-benzyl-4-*O*-(8-*N*-benzyl(benzyloxycarbonyl)-1-octyl)6-*O*-tert-butyldiphenylsilyl-α-D-glucopyranosyl)-6-*O*-benzoyl-cyclophellitol alkene (34)**

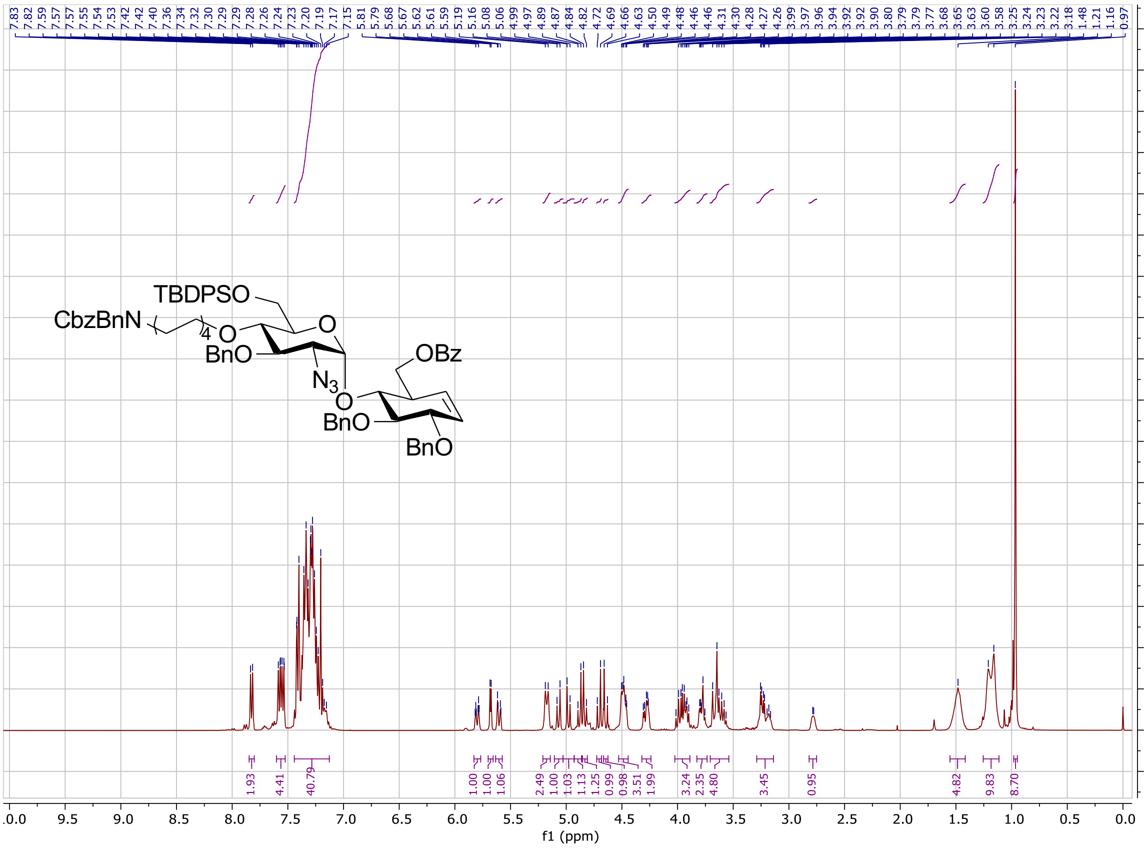


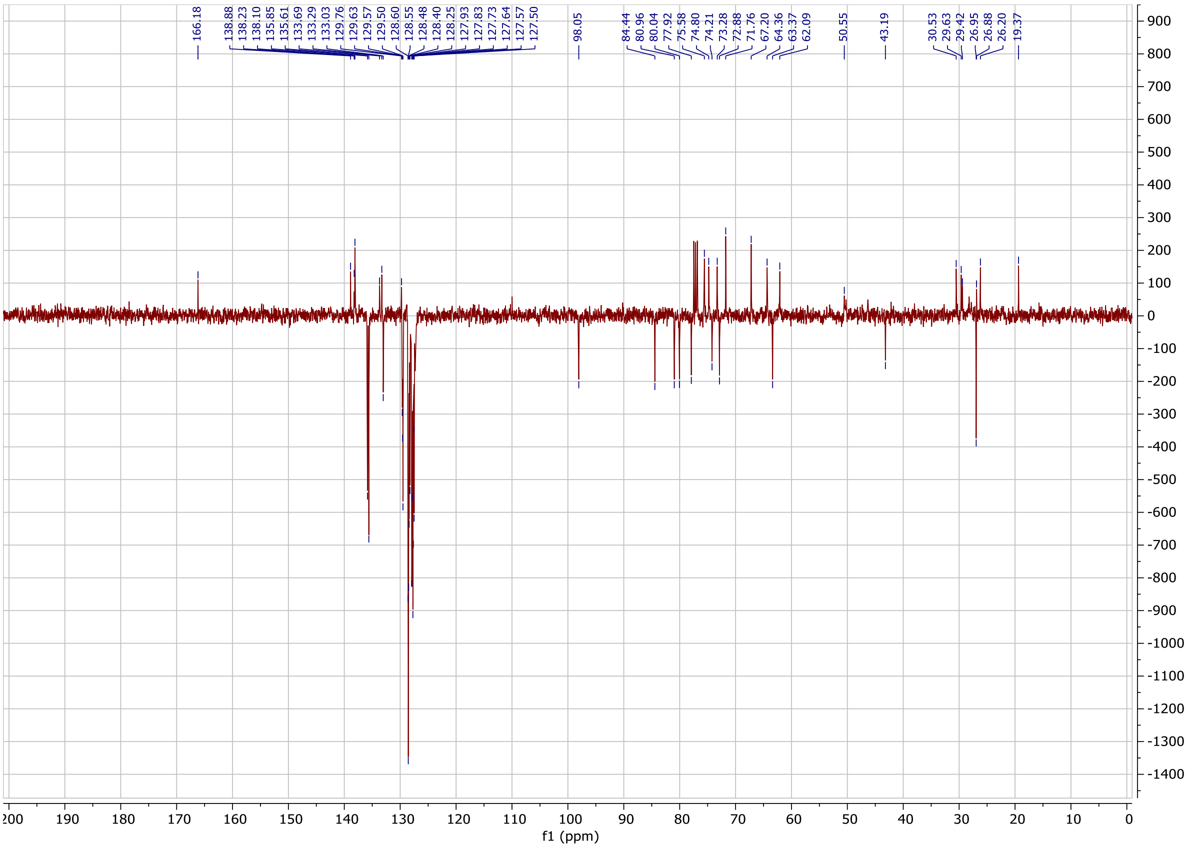

**2,3-di-*O*-benzyl-4-*O*-(2-*N*-acetyl-2-deoxy-3-*O*-benzyl-4-*O*-(8-*N*-benzyl(benzyloxycarbonyl)-1-octyl)-6-*O*-tert-butyldiphenylsilyl-α-D-glucopyranosyl)-6-*O*-benzoyl-cyclophellitol alkene (35a)**

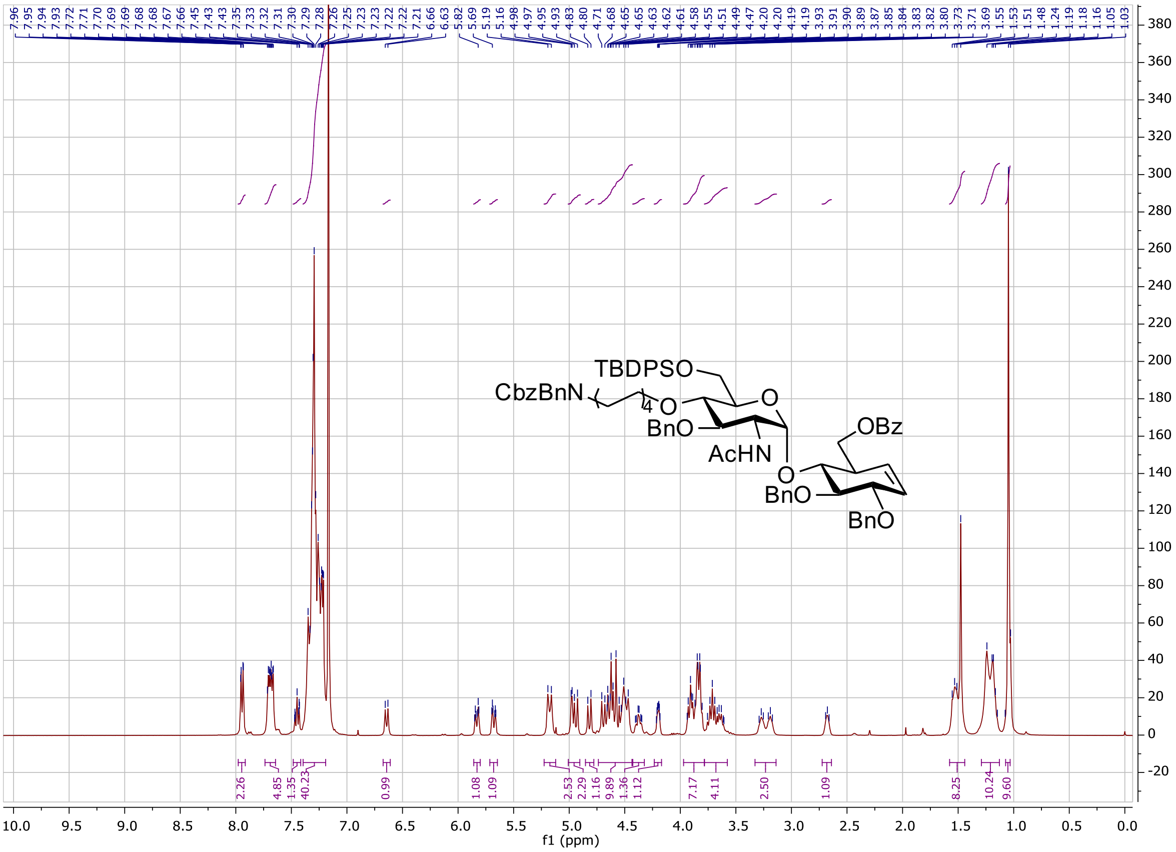


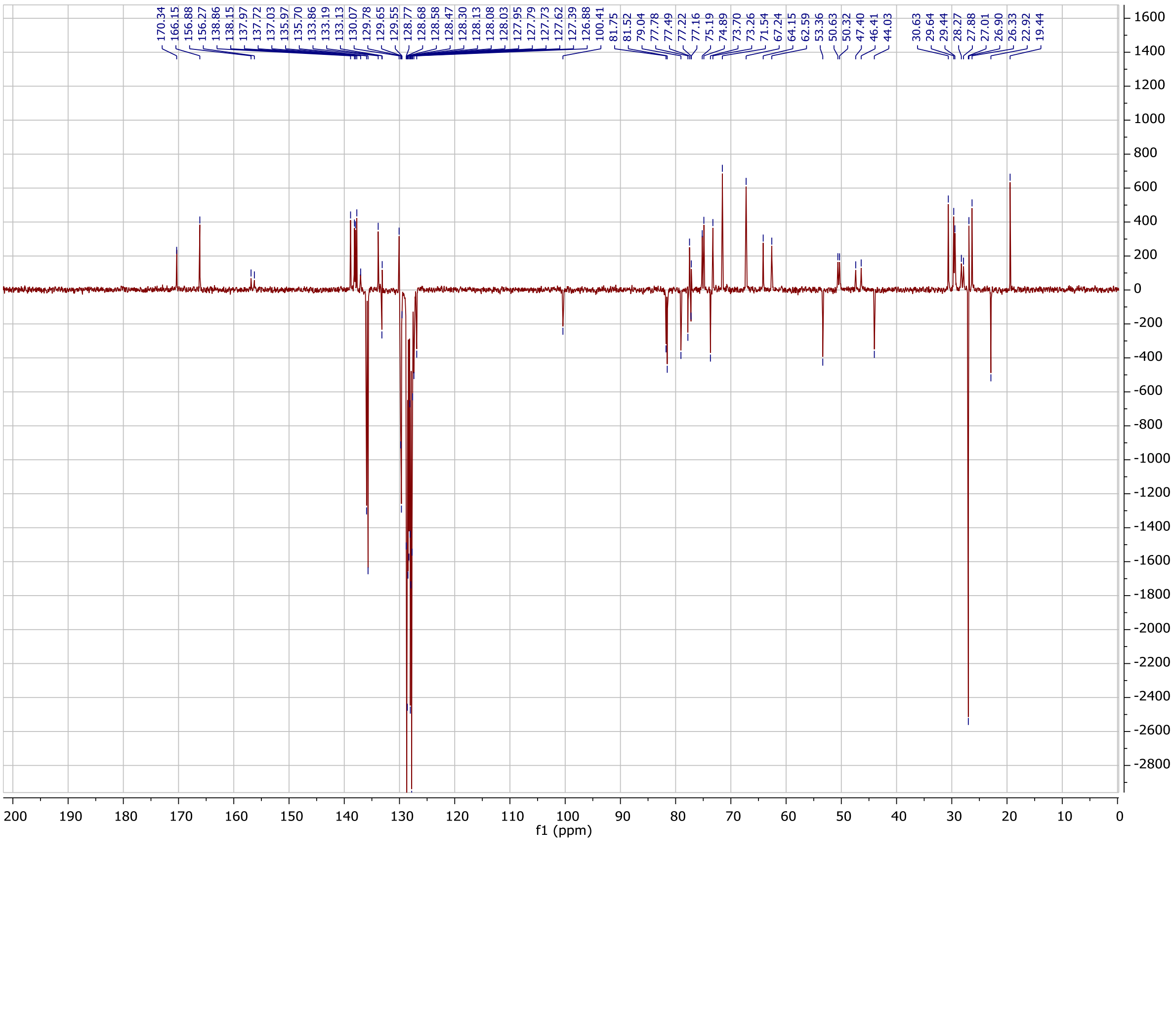

**2,3-di-*O*-benzyl-4-*O*-(2-*N*-acetyl-2-deoxy-3-*O*-benzyl-4-*O*-(8-*N*-benzyl-(benzyloxycarbonyl)-1-octyl)-6-*O*-tert-butyldiphenylsilyl-α-D-glucopyranosyl)-cyclophellitol alkene (35b)**

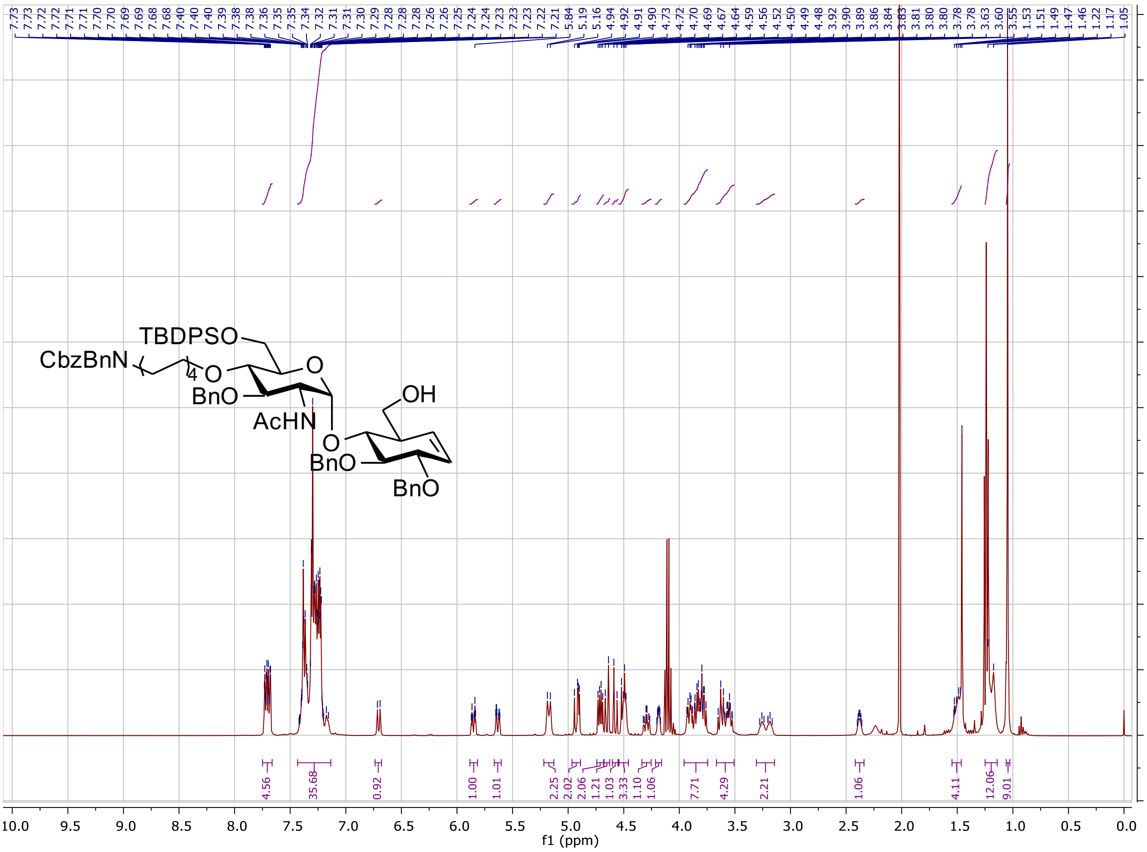


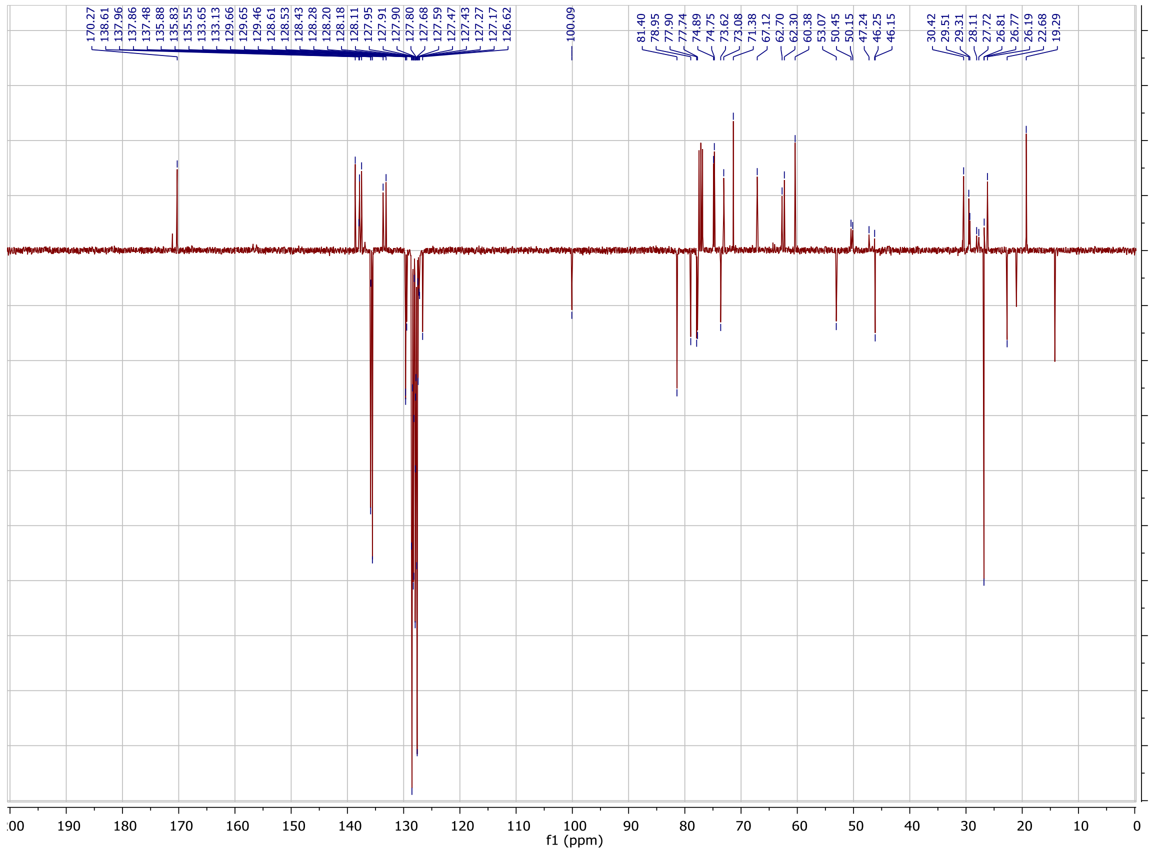

**2,3-di-*O*-benzyl-4-*O*-(2-*N*-acetyl-2-deoxy-3-*O*-benzyl-4-*O*-(8-*N*-benzyl-(benzyloxycarbonyl)-1-octyl)-6-*O*-tert-butyldiphenylsilyl-α-D-glucopyranosyl)-6-*O*-tert-butyloxycarbonyl-cyclophellitol alkene (35c)**

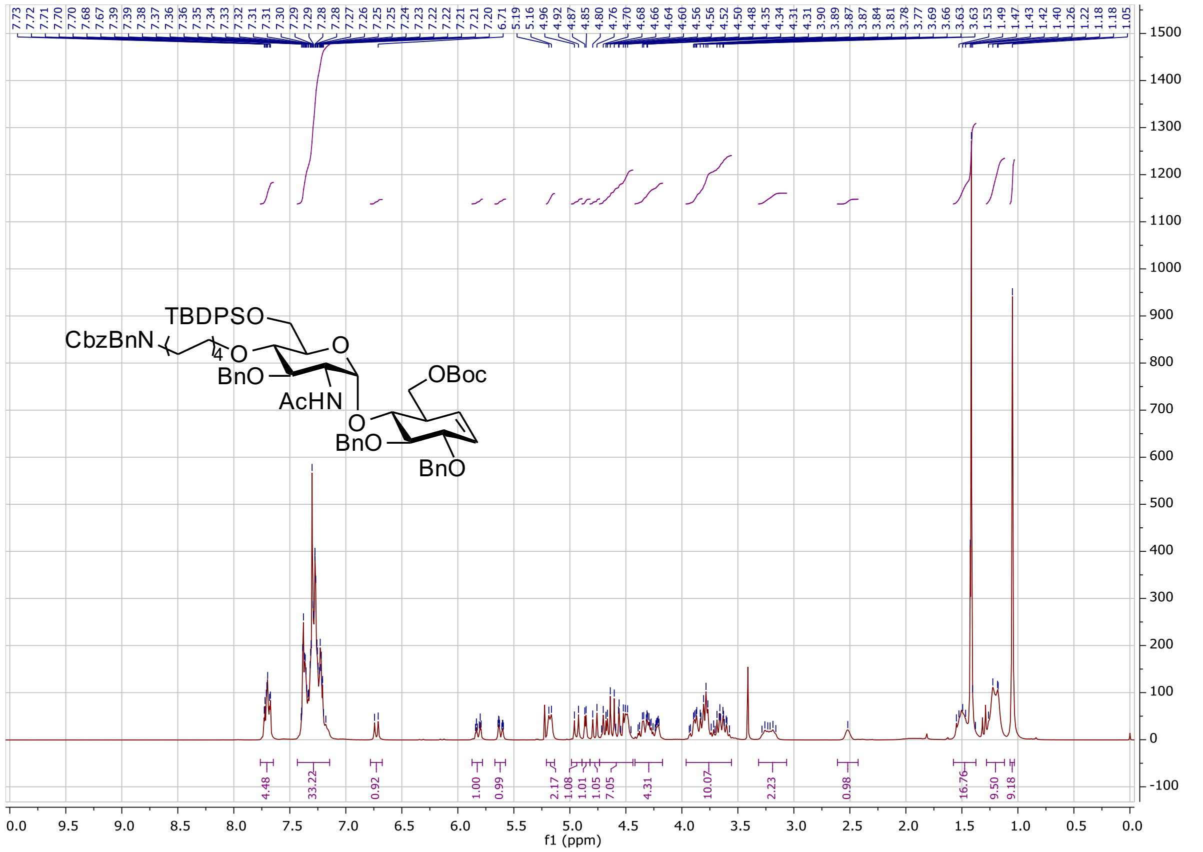


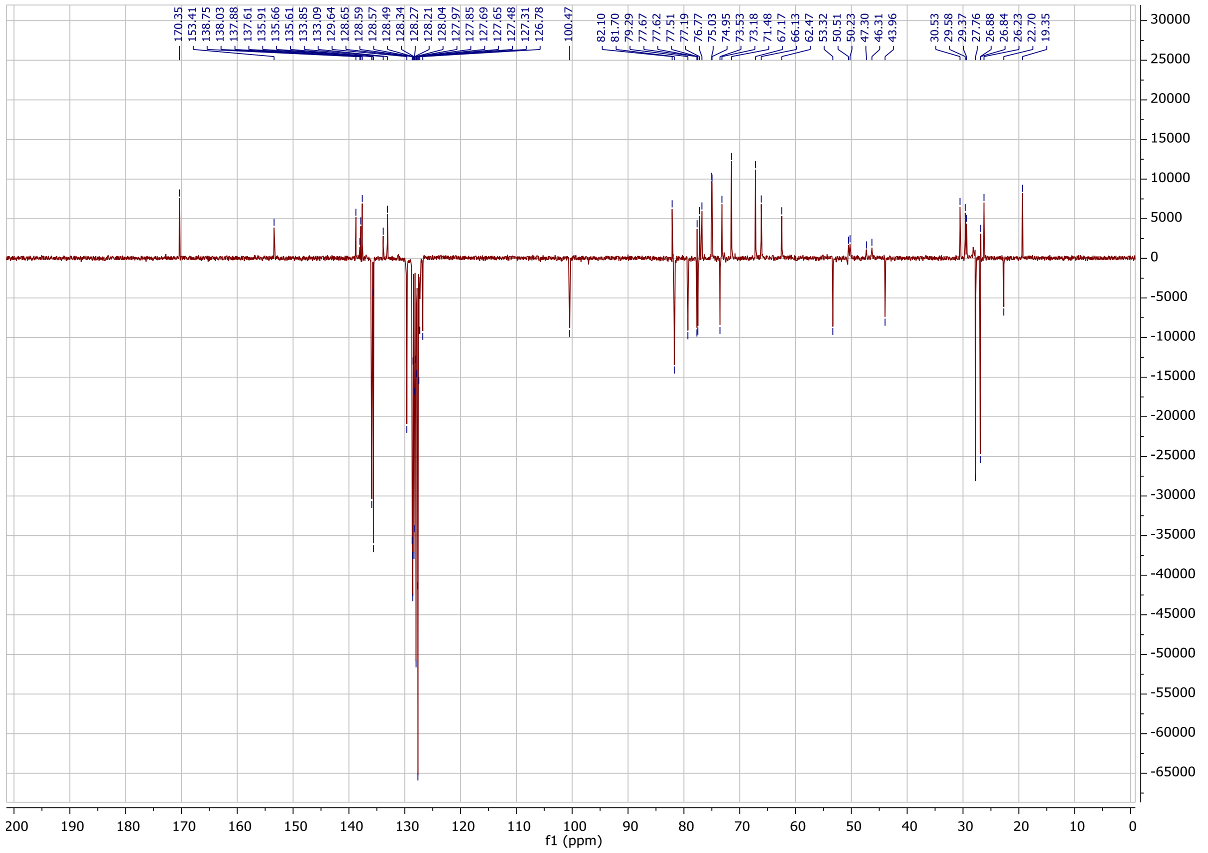

**1-iodo-2,3-di-*O*-benzyl-4-*O*-(2-*N*-acetyl-2-deoxy-3-*O*-benzyl-4-*O*-(8-*N*-benzyl-(benzyloxycarbonyl)-1-octyl)-6-*O*-tert-butyldiphenylsilyl-α-D-glucopyranosyl)-6,7-*O*-carbonyl-cyclophellitol alkane (35d)**

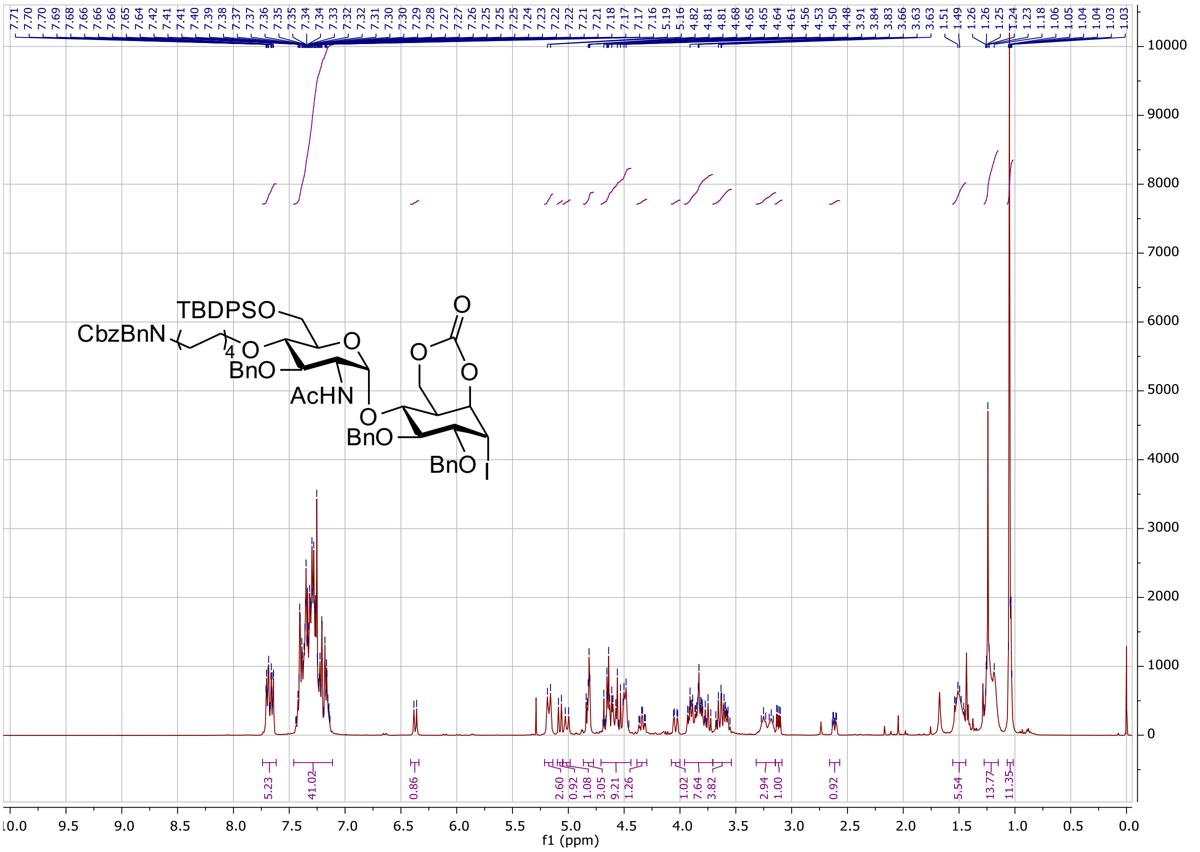


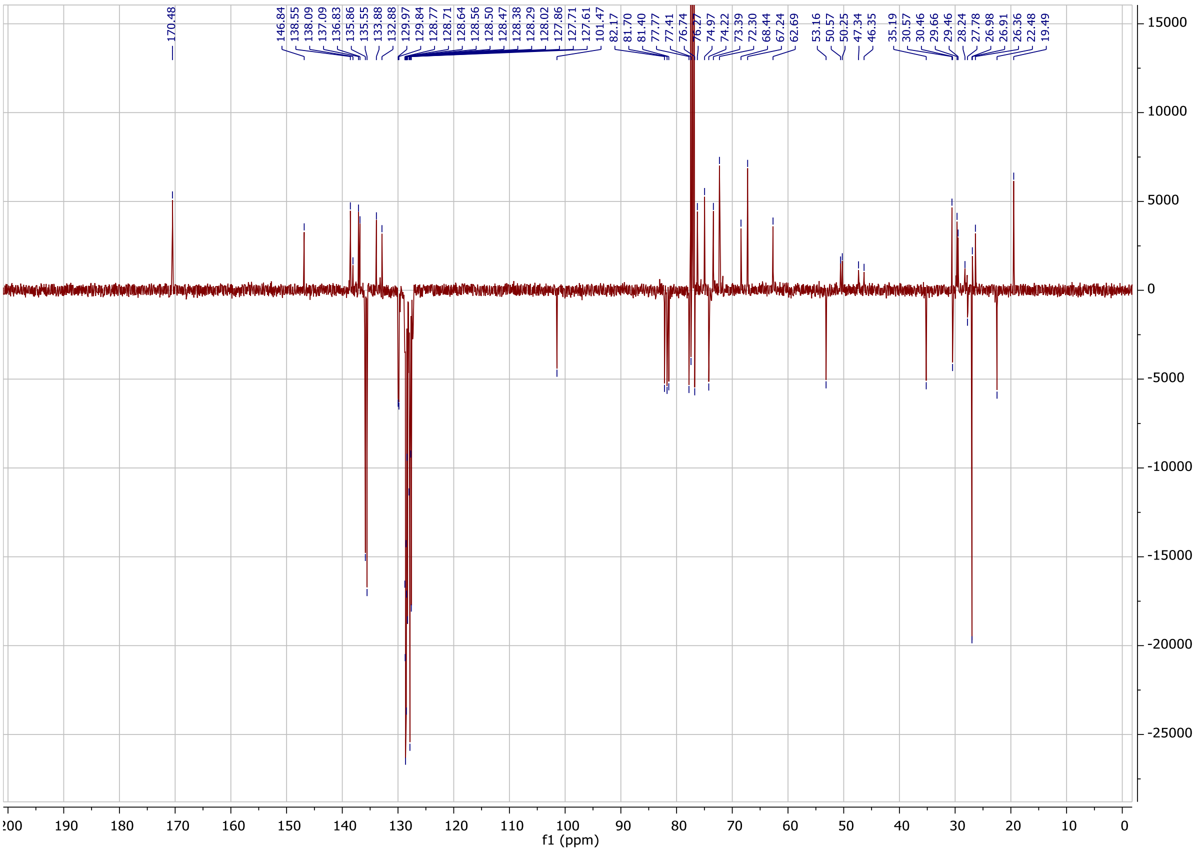

**2,3-di-*O*-benzyl-4-*O*-(2-*N*-acetyl-2-deoxy-3-*O*-benzyl-4-*O*-(8-*N*-benzyl-(benzyloxycarbonyl)-1-octyl)6-*O*-tert-butyldiphenylsilyl-α-D-glucopyranosyl)-cyclophellitol (36)**

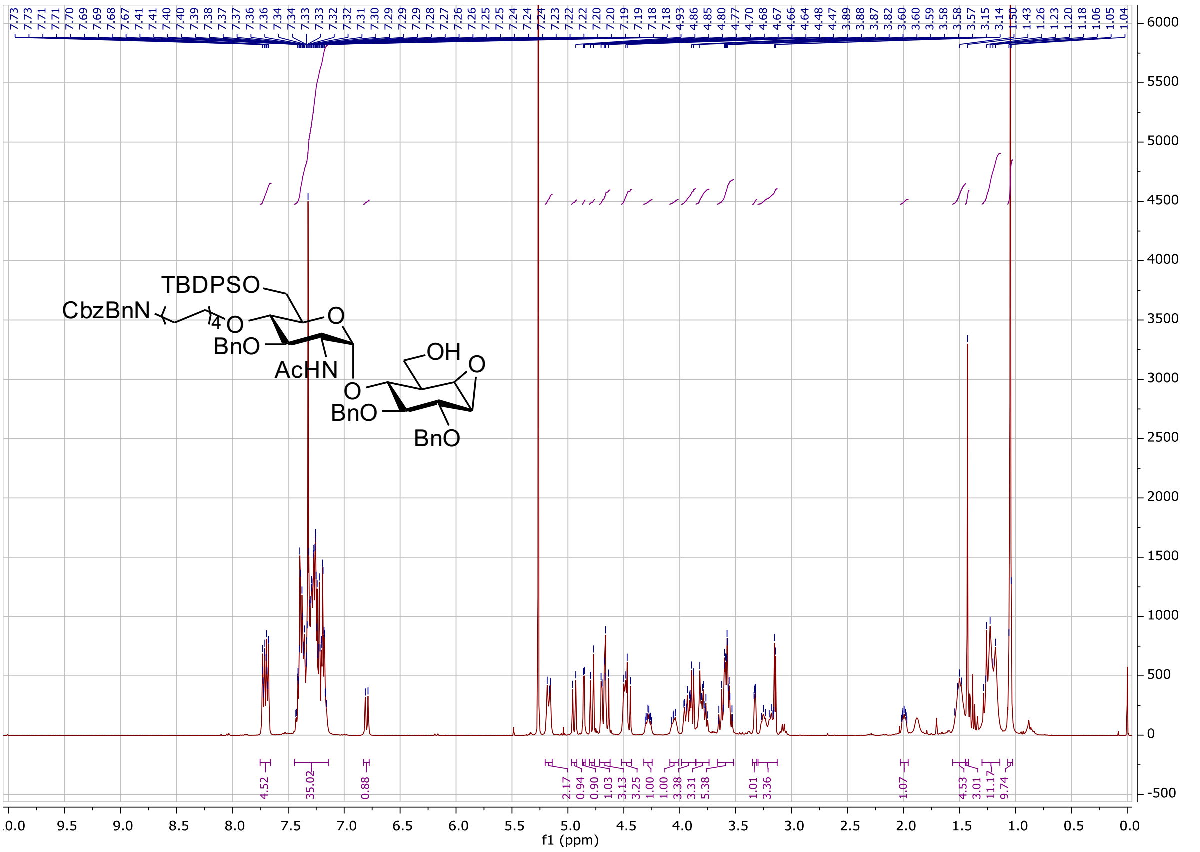

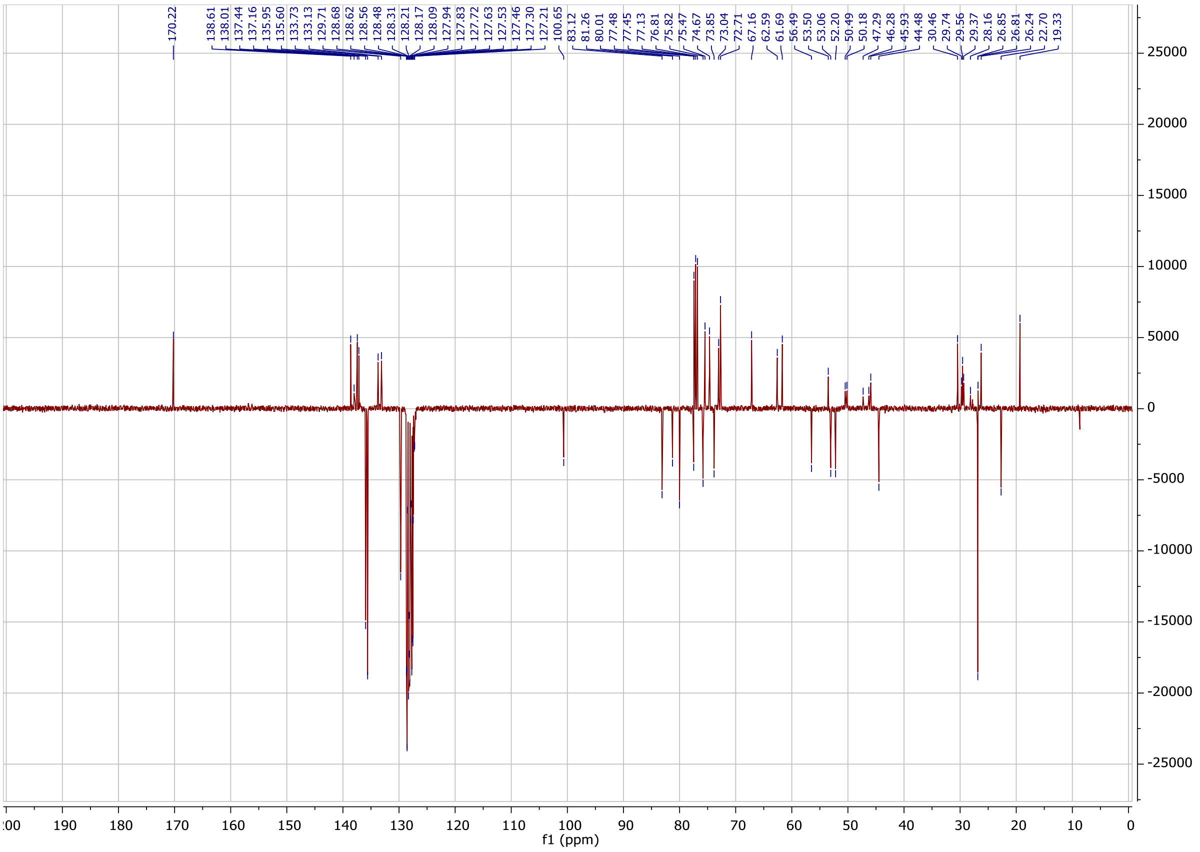

**2,3-di-*O*-benzyl-4-*O*-(2-*N*-acetyl-2-deoxy-3-*O*-benzyl-4-*O*-(8-*N*-benzyl-(benzyloxycarbonyl)-1-octyl)-6-*O*-tert-butyldiphenylsilyl-α-D-glucopyranosyl)-glucurono-cyclophellitol (37a)**

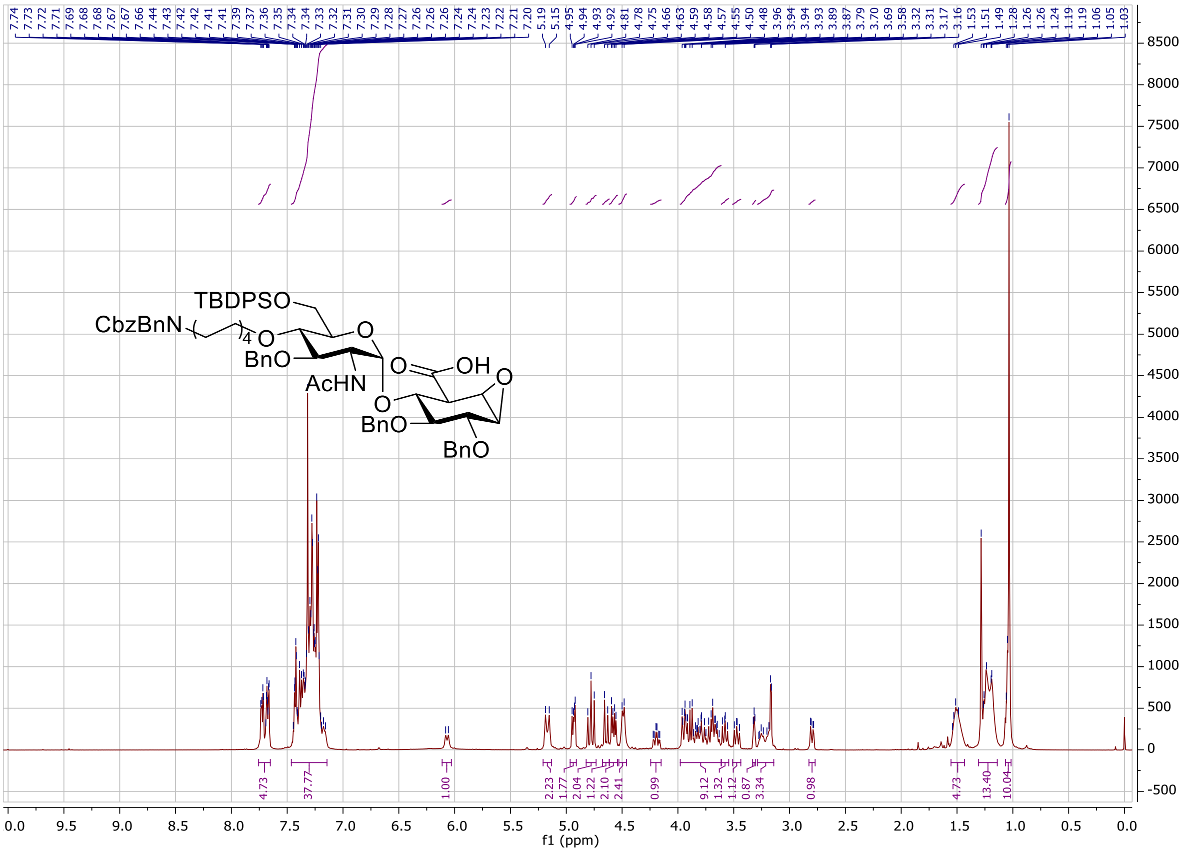


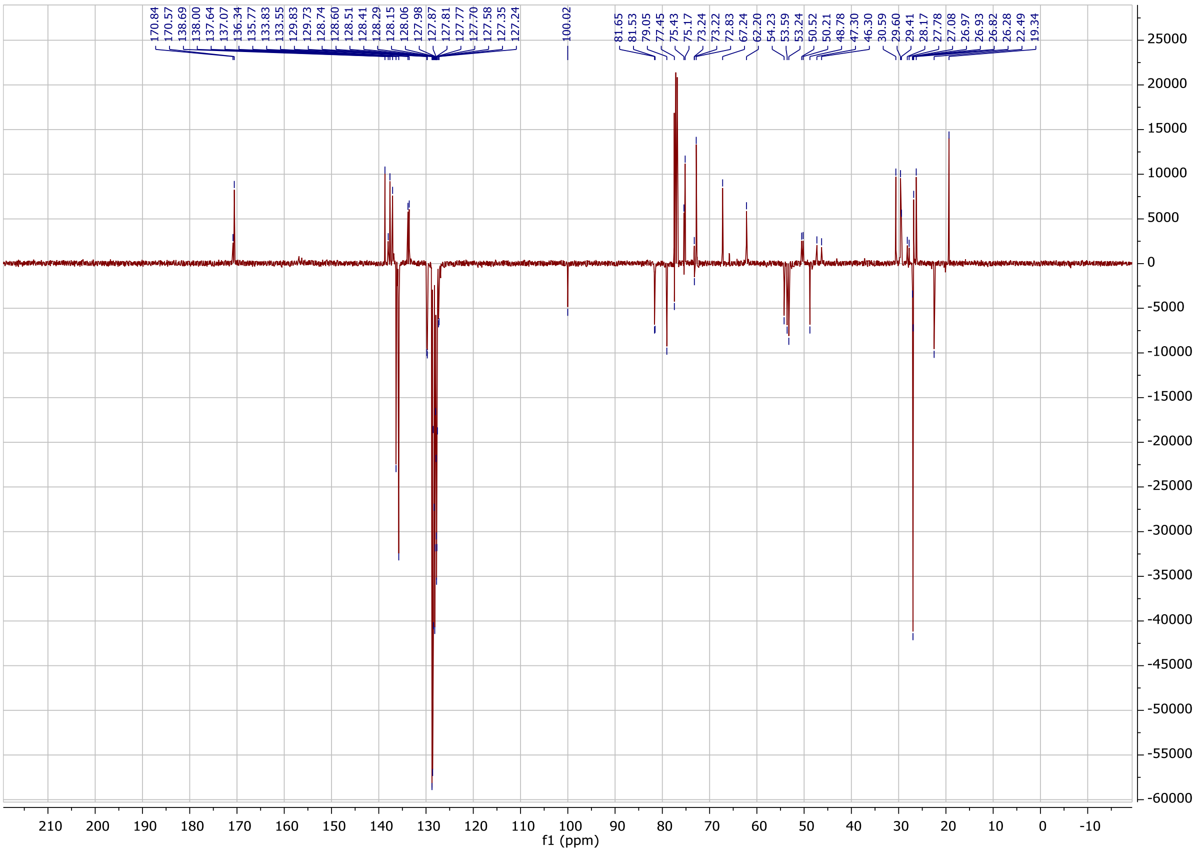

**2,3-di-*O*-benzyl-4-*O*-(2-*N*-acetyl-2-deoxy-3-*O*-benzyl-4-*O*-(8-*N*-benzyl(benzyloxycarbonyl)-1-octyl)-α-D-glucopyranosyl)-glucurono-cyclophellitol (37b)**

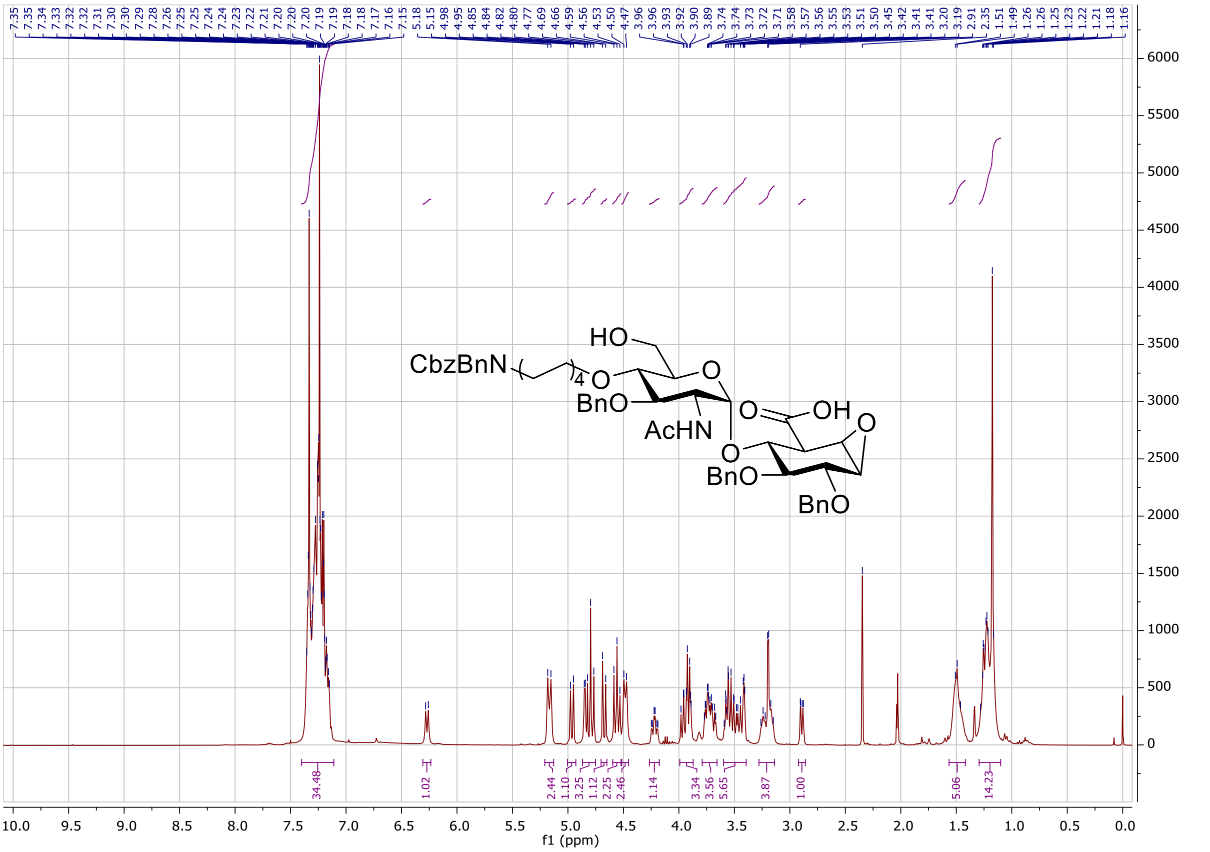


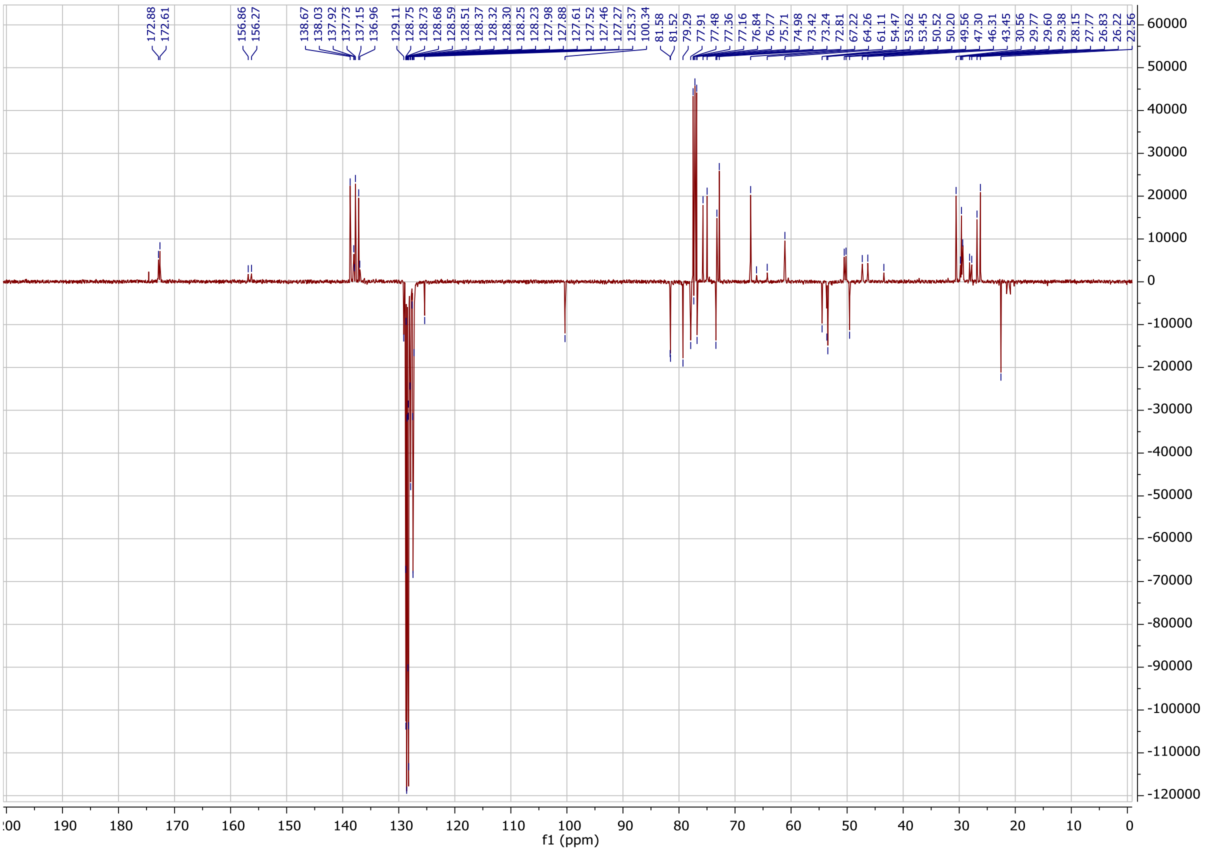

# 4-*O*-(2-*N*-acetyl-2-deoxy-4-*O*-(8-amino-1-octyl)-α-D-glucopyranosyl)-glucurono-cyclophellitol (38)

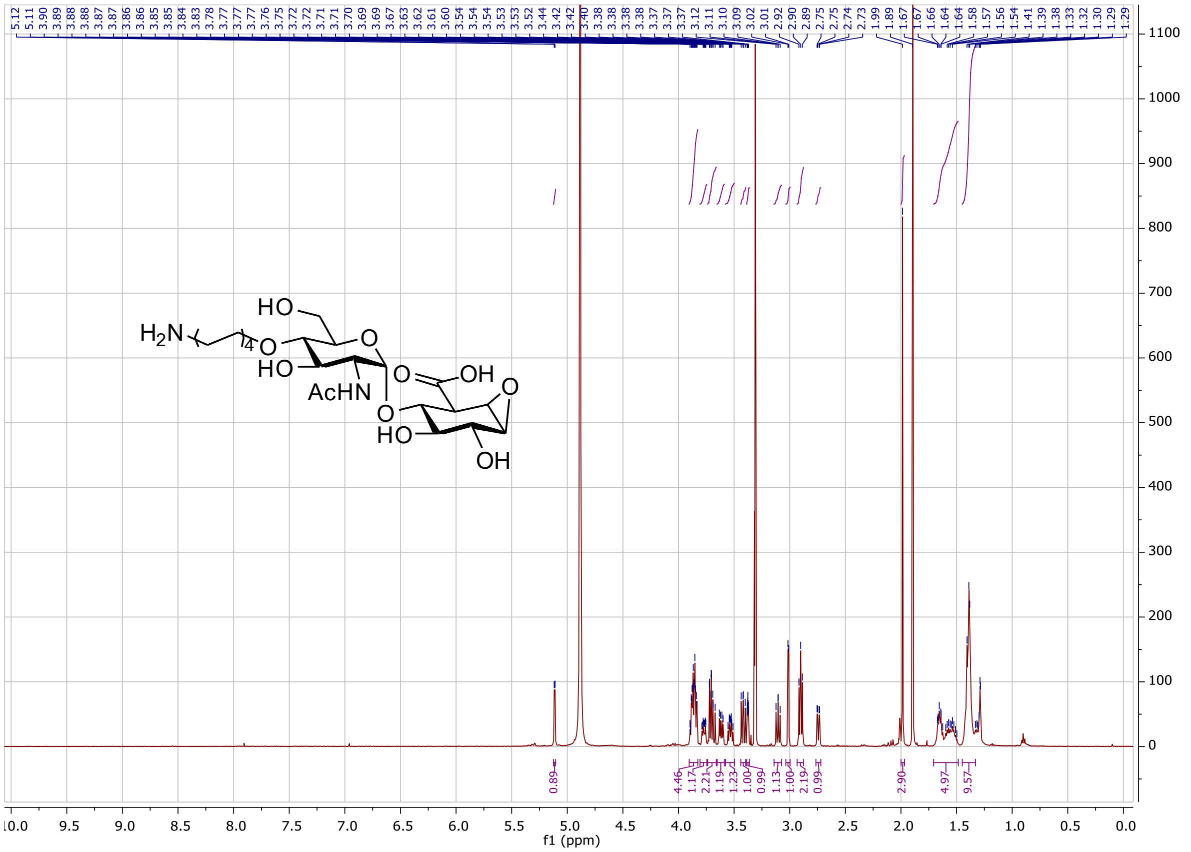

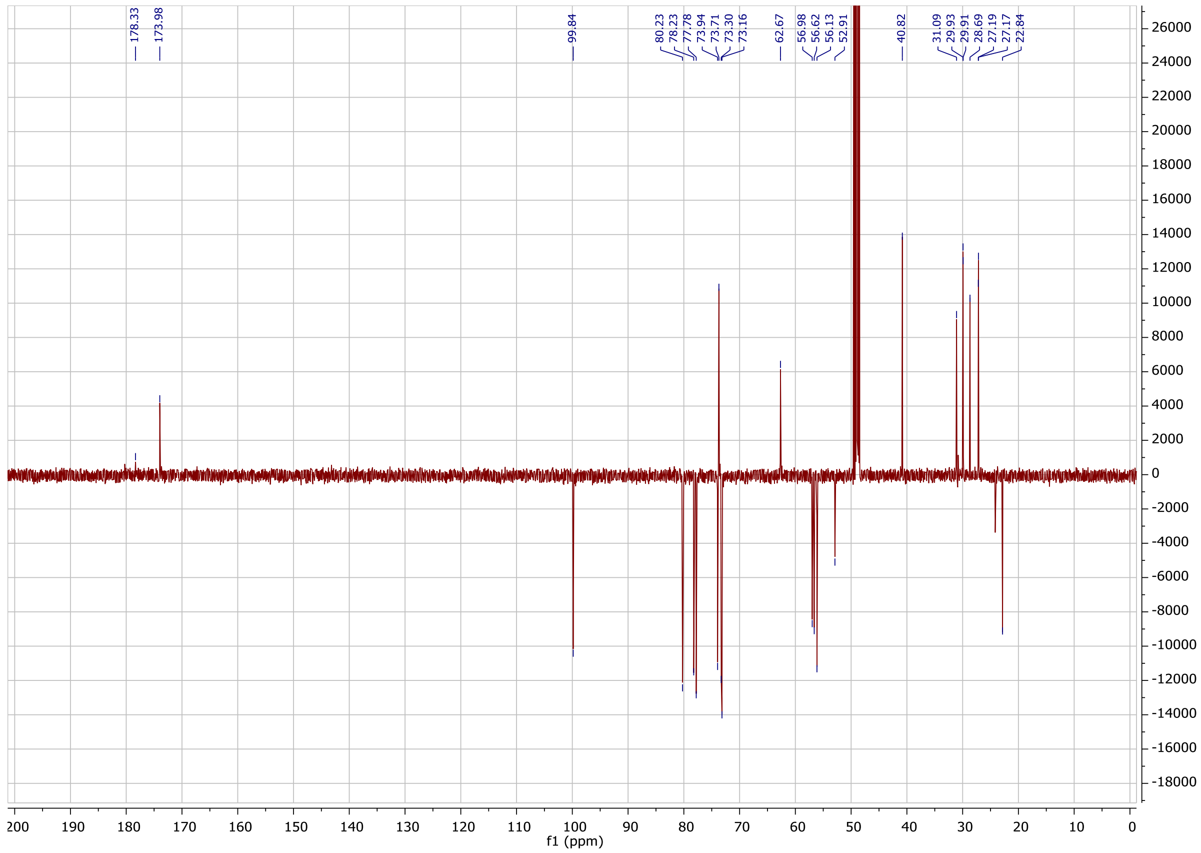

## 4-*O*-(2-*N*-acetyl-2-deoxy-4-*O*-(8-amino-Cy5-1-octyl)-α-D-glucopyranosyl)-glucurono-cyclophellitol (9)

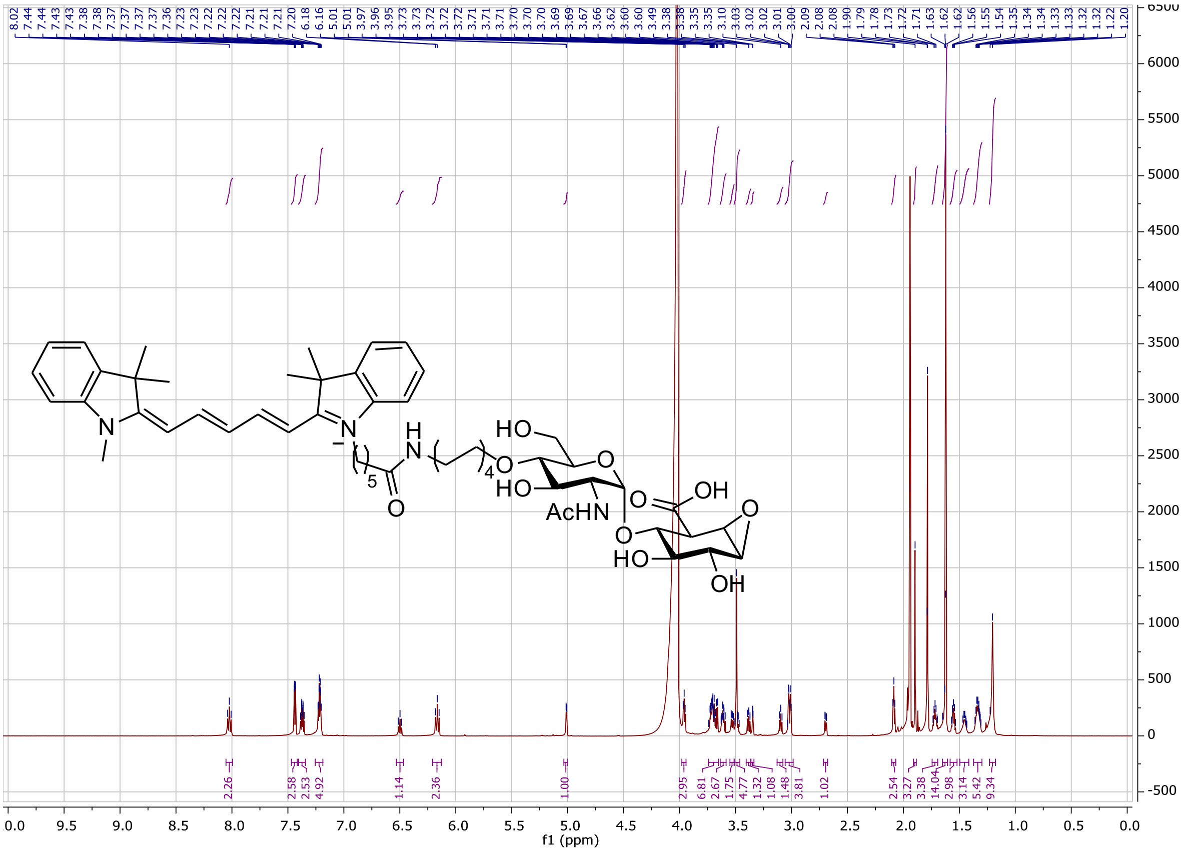


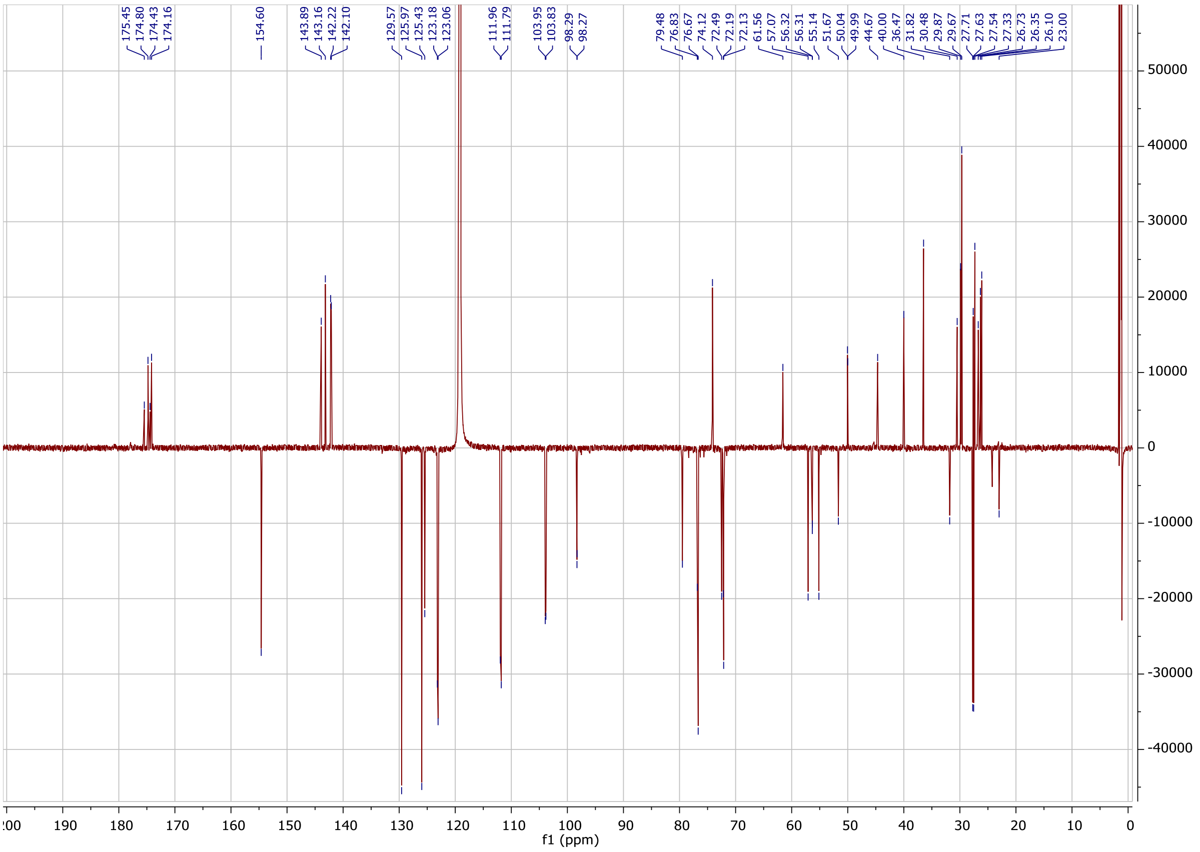

## Supporting Information References


1. B. L. Cantarel *et al.*, The Carbohydrate-Active EnZymes database (CAZy): an expert resource for Glycogenomics. *Nucleic Acids Res* **37**, D233-D238 (2009).
2. L. Wu, C. M. Viola, A. M. Brzozowski, G. J. Davies, Structural characterization of human heparanase reveals insights into substrate recognition. *Nat Struct Mol Biol* **22**, 1016-1022 (2015).
3. L. Wu *et al.*, Activity-based probes for functional interrogation of retaining beta-glucuronidases. *Nat Chem Biol* **13**, 867-873 (2017).
4. G. Winter, xia2: an expert system for macromolecular crystallography data reduction. *Journal of Applied Crystallography* **43**, 186-190 (2010).
5. A. J. McCoy *et al.*, Phaser crystallographic software. *J Appl Crystallogr* **40**, 658-674 (2007).
6. P. Emsley, B. Lohkamp, W. G. Scott, K. Cowtan, Features and development of Coot. *Acta Crystallogr D Biol Crystallogr* **66**, 486-501 (2010).
7. G. N. Murshudov *et al.*, REFMAC5 for the refinement of macromolecular crystal structures. *Acta Crystallogr D Biol Crystallogr* **67**, 355-367 (2011).
8. A. A. Lebedev *et al.*, JLigand: a graphical tool for the CCP4 template-restraint library. *Acta Crystallogr D Biol Crystallogr* **68**, 431-440 (2012).
9. E. Hammond, C. P. Li, V. Ferro, Development of a colorimetric assay for heparanase activity suitable for kinetic analysis and inhibitor screening. *Anal Biochem* **396**, 112-116 (2010).
10. I. Vlodavsky, Preparation of extracellular matrices produced by cultured corneal endothelial and PF-HR9 endodermal cells. *Curr Protoc Cell Biol* **Chapter 10**, Unit 10 14 (2001).
11. I. Vlodavsky *et al.*, Mammalian heparanase: gene cloning, expression and function in tumor progression and metastasis. *Nat Med* **5**, 793-802 (1999).
12. U. Barash *et al.*, Involvement of Heparanase in the Pathogenesis of Mesothelioma: Basic Aspects and Clinical Applications. *Journal of the National Cancer Institute* **110**, 1102-1114 (2018).
13. M. Weissmann *et al.*, Heparanase-neutralizing antibodies attenuate lymphoma tumor growth and metastasis. *Proc Natl Acad Sci* **113**, 704-709 (2016).
14. E. C. Lourenco, M. R. Ventura, Improvement of the stereoselectivity of the glycosylation reaction with 2-azido-2-deoxy-1-thioglucoside donors. *Carbohydr Res* **426**, 33-39 (2016).
